# Adolescent transport and unintentional injuries: a systematic analysis using the Global Burden of Disease Study 2019

**DOI:** 10.1016/S2468-2667(22)00134-7

**Published:** 2022-06-30

**Authors:** GBD 2019 Adolescent Transport and Unintentional Injuries Collaborators, Amy E Peden, Patricia Cullen, Kate Louise Francis, Holger Moeller, Margaret M Peden, Pengpeng Ye, Maoyi Tian, Zhiyong Zou, Susan M Sawyer, Amirali Aali, Zeinab Abbasi-Kangevari, Mohsen Abbasi-Kangevari, Michael Abdelmasseh, Meriem Abdoun, Rami Abd-Rabu, Deldar Morad Abdulah, Getachew Abebe, Ayele Mamo Abebe, Aidin Abedi, Hassan Abidi, Richard Gyan Aboagye, Hiwa Abubaker Ali, Eman Abu-Gharbieh, Denberu Eshetie Adane, Tigist Demssew Adane, Isaac Yeboah Addo, Ololade Grace Adewole, Sangeet Adhikari, Mohammad Adnan, Qorinah Estiningtyas Sakilah Adnani, Aanuoluwapo Adeyimika Bachelor Afolabi, Saira Afzal, Muhammad Sohail Afzal, Zahra Babaei Aghdam, Bright Opoku Ahinkorah, Araz Ramazan Ahmad, Tauseef Ahmad, Sajjad Ahmad, Ali Ahmadi, Haroon Ahmed, Muktar Beshir Ahmed, Ali Ahmed, Ayman Ahmed, Jivan Qasim Ahmed, Tarik Ahmed Rashid, Janardhana P Aithala, Budi Aji, Meisam Akhlaghdoust, Fares Alahdab, Fahad Mashhour Alanezi, Astawus Alemayehu, Hanadi Al Hamad, Syed Shujait Ali, Liaqat Ali, Yousef Alimohamadi, Vahid Alipour, Syed Mohamed Aljunid, Louay Almidani, Sami Almustanyir, Khalid A Altirkawi, Nelson J Alvis-Zakzuk, Edward Kwabena Ameyaw, Tarek Tawfik Amin, Mehrdad Amir-Behghadami, Sohrab Amiri, Hoda Amiri, Tadele Fentabil Anagaw, Tudorel Andrei, Catalina Liliana Andrei, Davood Anvari, Sumadi Lukman Anwar, Anayochukwu Edward Anyasodor, Jalal Arabloo, Morteza Arab-Zozani, Asrat Arja, Judie Arulappan, Ashokan Arumugam, Armin Aryannejad, Saeed Asgary, Tahira Ashraf, Seyyed Shamsadin Athari, Alok Atreya, Sameh Attia, Avinash Aujayeb, Atalel Fentahun Awedew, Sina Azadnajafabad, Mohammadreza Azangou-Khyavy, Samad Azari, Amirhossein Azari Jafari, Hosein Azizi, Ahmed Y Azzam, Ashish D Badiye, Nayereh Baghcheghi, Sara Bagherieh, Atif Amin Baig, Shankar M Bakkannavar, Asaminew Birhanu Balta, Maciej Banach, Palash Chandra Banik, Hansi Bansal, Mainak Bardhan, Francesco Barone-Adesi, Amadou Barrow, Azadeh Bashiri, Pritish Baskaran, Saurav Basu, Nebiyou Simegnew Bayileyegn, Abebe Ayalew Bekel, Alehegn Bekele Bekele, Salaheddine Bendak, Isabela M Bensenor, Alemshet Yirga Berhie, Devidas S Bhagat, Akshaya Srikanth Bhagavathula, Pankaj Bhardwaj, Nikha Bhardwaj, Sonu Bhaskar, Ajay Nagesh Bhat, Krittika Bhattacharyya, Zulfiqar A Bhutta, Sadia Bibi, Bagas Suryo Bintoro, Saeid Bitaraf, Belay Boda Abule Bodicha, Archith Boloor, Souad Bouaoud, Julie Brown, Katrin Burkart, Nadeem Shafique Butt, Muhammad Hammad Butt, Luis Alberto Cámera, Julio Cesar Campuzano Rincon, Chao Cao, Andre F Carvalho, Márcia Carvalho, Promit Ananyo Chakraborty, Eeshwar K Chandrasekar, Jung-Chen Chang, Periklis Charalampous, Jaykaran Charan, Vijay Kumar Chattu, Bitew Mekonnen Chekole, Abdulaal Chitheer, Daniel Youngwhan Cho, Hitesh Chopra, Devasahayam J Christopher, Isaac Sunday Chukwu, Natália Cruz-Martins, Omid Dadras, Saad M A Dahlawi, Xiaochen Dai, Giovanni Damiani, Gary L Darmstadt, Reza Darvishi Cheshmeh Soltani, Aso Mohammad Darwesh, Saswati Das, Anna Dastiridou, Sisay Abebe Debela, Amin Dehghan, Getnet Makasha Demeke, Andreas K Demetriades, Solomon Demissie, Fikadu Nugusu Dessalegn, Abebaw Alemayehu Desta, Mostafa Dianatinasab, Nancy Diao, Diana Dias da Silva, Daniel Diaz, Lankamo Ena Digesa, Mengistie Diress, Shirin Djalalinia, Linh Phuong Doan, Milad Dodangeh, Paul Narh Doku, Deepa Dongarwar, Haneil Larson Dsouza, Ebrahim Eini, Michael Ekholuenetale, Temitope Cyrus Ekundayo, Ahmed Elabbas Mustafa Elagali, Mostafa Ahmed Elbahnasawy, Hala Rashad Elhabashy, Muhammed Elhadi, Maysaa El Sayed Zaki, Daniel Berhanie Enyew, Ryenchindorj Erkhembayar, Sharareh Eskandarieh, Farshid Etaee, Adeniyi Francis Fagbamigbe, Pawan Sirwan Faris, Abbas Farmany, Andre Faro, Farshad Farzadfar, Ali Fatehizadeh, Seyed-Mohammad Fereshtehnejad, Abdullah Hamid Feroze, Getahun Fetensa, Bikila Regassa Feyisa, Irina Filip, Florian Fischer, Behzad Foroutan, Masoud Foroutan, Kayode Raphael Fowobaje, Richard Charles Franklin, Takeshi Fukumoto, Peter Andras Gaal, Muktar A Gadanya, Yaseen Galali, Nasrin Galehdar, Balasankar Ganesan, Tushar Garg, Mesfin Gebrehiwot Damtew Gebrehiwot, Yosef Haile Gebremariam, Yibeltal Yismaw Gela, Urge Gerema, Mansour Ghafourifard, Seyyed-Hadi Ghamari, Reza Ghanbari, Mohammad Ghasemi Nour, Maryam Gholamalizadeh, Ali Gholami, Ali Gholamrezanezhad, Sherief Ghozy, Syed Amir Gilani, Tiffany K Gill, Iago Giné-Vázquez, Zeleke Abate Girma, James C Glasbey, Franklin N Glozah, Mahaveer Golechha, Pouya Goleij, Michal Grivna, Habtamu Alganeh Guadie, Damitha Asanga Gunawardane, Yuming Guo, Veer Bala Gupta, Sapna Gupta, Bhawna Gupta, Vivek Kumar Gupta, Arvin Haj-Mirzaian, Rabih Halwani, Randah R Hamadeh, Sajid Hameed, Asif Hanif, Arief Hargono, Netanja I Harlianto, Mehdi Harorani, Ahmed I Hasaballah, S M Mahmudul Hasan, Amr Hassan, Soheil Hassanipour, Hadi Hassankhani, Rasmus J Havmoeller, Simon I Hay, Mohammad Heidari, Delia Hendrie, Demisu Zenbaba Heyi, Yuta Hiraike, Ramesh Holla, Nobuyuki Horita, Sheikh Jamal Hossain, Mohammad Bellal Hossain Hossain, Sedighe Hosseini Shabanan, Mehdi Hosseinzadeh, Sorin Hostiuc, Amir Human Hoveidaei, Alexander Kevin Hsiao, Salman Hussain, Amal Hussein, Segun Emmanuel Ibitoye, Olayinka Stephen Ilesanmi, Irena M Ilic, Milena D Ilic, Mustapha Immurana, Leeberk Raja Inbaraj, Sheikh Mohammed Shariful Islam, Rakibul M Islam, Mohammad Mainul Islam, Nahlah Elkudssiah Ismail, Linda Merin J, Haitham Jahrami, Mihajlo Jakovljevic, Manthan Dilipkumar Janodia, Tahereh Javaheri, Sathish Kumar Jayapal, Umesh Umesh Jayarajah, Sudha Jayaraman, Jayakumar Jeganathan, Bedru Jemal, Ravi Prakash Jha, Jost B Jonas, Tamas Joo, Nitin Joseph, Jacek Jerzy Jozwiak, Mikk Jürisson, Ali Kabir, Vidya Kadashetti, Dler Hussein Kadir, Laleh R Kalankesh, Leila R Kalankesh, Rohollah Kalhor, Vineet Kumar Kamal, Rajesh Kamath, Himal Kandel, Rami S Kantar, Neeti Kapoor, Hassan Karami, Ibraheem M Karaye, Samad Karkhah, Patrick DMC Katoto, Joonas H Kauppila, Gbenga A Kayode, Leila Keikavoosi-Arani, Cumali Keskin, Yousef Saleh Khader, Himanshu Khajuria, Mohammad Khammarnia, Ejaz Ahmad Khan, Md Nuruzzaman Khan, Maseer Khan, Yusra H Khan, Imteyaz A Khan, Abbas Khan, Moien AB Khan, Javad Khanali, Moawiah Mohammad Khatatbeh, Hamid Reza Khayat Kashani, Jagdish Khubchandani, Zemene Demelash Kifle, Jihee Kim, Yun Jin Kim, Sezer Kisa, Adnan Kisa, Cameron J Kneib, Farzad Kompani, Hamid Reza Koohestani, Parvaiz A Koul, Sindhura Lakshmi Koulmane Laxminarayana, Ai Koyanagi, Kewal Krishan, Vijay Krishnamoorthy, Burcu Kucuk Bicer, Nithin Kumar, Naveen Kumar, Narinder Kumar, Manasi Kumar, Om P Kurmi, Lucie Laflamme, Judit Lám, Iván Landires, Bagher Larijani, Savita Lasrado, Paolo Lauriola, Carlo La Vecchia, Shaun Wen Huey Lee, Yo Han Lee, Sang-woong Lee, Wei-Chen Lee, Samson Mideksa Legesse, Shanshan Li, Stephen S Lim, László Lorenzovici, Amana Ogeto Luke, Farzan Madadizadeh, Áurea M Madureira-Carvalho, Muhammed Magdy Abd El Razek, Soleiman Mahjoub, Ata Mahmoodpoor, Razzagh Mahmoudi, Marzieh Mahmoudimanesh, Azeem Majeed, Alaa Makki, Elaheh Malakan Rad, Mohammad-Reza Malekpour, Ahmad Azam Malik, Tauqeer Hussain Mallhi, Deborah Carvalho Malta, Borhan Mansouri, Mohammad Ali Mansournia, Elezebeth Mathews, Sazan Qadir Maulud, Dennis Mazingi, Entezar Mehrabi Nasab, Oliver Mendoza-Cano, Ritesh G Menezes, Dechasa Adare Mengistu, Alexios-Fotios A Mentis, Atte Meretoja, Mohamed Kamal Mesregah, Tomislav Mestrovic, Ana Carolina Micheletti Gomide Nogueira de Sá, Ted R Miller, Seyed Peyman Mirghaderi, Andreea Mirica, Seyyedmohammadsadeq Mirmoeeni, Erkin M Mirrakhimov, Moonis Mirza, Sanjeev Misra, Prasanna Mithra, Chaitanya Mittal, Madeline E Moberg, Mokhtar Mohammadi, Soheil Mohammadi, Esmaeil Mohammadi, Reza Mohammadpourhodki, Shafiu Mohammed, Teroj Abdulrahman Mohammed, Mohammad Mohseni, Ali H Mokdad, Sara Momtazmanesh, Lorenzo Monasta, Mohammad Ali Moni, Rafael Silveira Moreira, Shane Douglas Morrison, Ebrahim Mostafavi, Haleh Mousavi Isfahani, Sumaira Mubarik, Lorenzo Muccioli, Soumyadeep Mukherjee, Francesk Mulita, Ghulam Mustafa, Ahamarshan Jayaraman Nagarajan, Mukhammad David Naimzada, Vinay Nangia, Hasan Nassereldine, Zuhair S Natto, Biswa Prakash Nayak, Ionut Negoi, Seyed Aria Nejadghaderi, Samata Nepal, Sandhya Neupane Kandel, Nafise Noroozi, Virginia Nuñez-Samudio, Ogochukwu Janet Nzoputam, Chimezie Igwegbe Nzoputam, Chimedsuren Ochir, Julius Nyerere Odhiambo, Oluwakemi Ololade Odukoya, Hassan Okati-Aliabad, Osaretin Christabel Okonji, Andrew T Olagunju, Ahmed Omar Bali, Emad Omer, Adrian Otoiu, Stanislav S Otstavnov, Nikita Otstavnov, Bilcha Oumer, Mayowa O Owolabi, Mahesh P A, Alicia Padron-Monedero, Jagadish Rao Padubidri, Mohammad Taha Pahlevan Fallahy, Songhomitra Panda-Jonas, Seithikurippu R Pandi-Perumal, Shahina Pardhan, Eun-Kee Park, Sangram Kishor Patel, Aslam Ramjan Pathan, Siddhartha Pati, Uttam Paudel, Shrikant Pawar, Paolo Pedersini, Mario F P Peres, Ionela-Roxana Petcu, Michael R Phillips, Julian David Pillay, Zahra Zahid Piracha, Mohsen Poursadeqiyan, Naeimeh Pourtaheri, Ibrahim Qattea, Amir Radfar, Ata Rafiee, Pankaja Raghav Raghav, Fakher Rahim, Muhammad Aziz Rahman, Firman Suryadi Rahman, Mosiur Rahman, Amir Masoud Rahmani, Shayan Rahmani, Sheetal Raj Moolambally, Sheena Ramazanu, Kiana Ramezanzadeh, Juwel Rana, Chythra R Rao, Sowmya J Rao, Vahid Rashedi, Mohammad-Mahdi Rashidi, Prateek Rastogi, Azad Rasul, Salman Rawaf, David Laith Rawaf, Lal Rawal, Reza Rawassizadeh, Negar Rezaei, Nazila Rezaei, Mohsen Rezaeian, Aziz Rezapour, Abanoub Riad, Muhammad Riaz, Jennifer Rickard, Jefferson Antonio Buendia Rodriguez, Leonardo Roever, Luca Ronfani, Bedanta Roy, Manjula S, Aly M A Saad, Siamak Sabour, Leila Sabzmakan, Basema Saddik, Malihe Sadeghi, Mohammad Reza Saeb, Umar Saeed, Sahar Saeedi Moghaddam, Sher Zaman Safi, Biniyam Sahiledengle, Harihar Sahoo, Mohammad Ali Sahraian, Morteza Saki, Payman Salamati, Sana Salehi, Marwa Rashad Salem, Abdallah M Samy, Juan Sanabria, Milena M Santric-Milicevic, Muhammad Arif Nadeem Saqib, Yaser Sarikhani, Arash Sarveazad, Brijesh Sathian, Maheswar Satpathy, Ganesh Kumar Saya, Ione Jayce Ceola Schneider, David C Schwebel, Hamed Seddighi, Subramanian Senthilkumaran, Allen Seylani, Hosein Shabaninejad, Melika Shafeghat, Pritik A Shah, Saeed Shahabi, Ataollah Shahbandi, Fariba Shahraki-Sanavi, Masood Ali Shaikh, Elaheh Shaker, Mehran Shams-Beyranvand, Mohd Shanawaz, Mohammed Shannawaz, Mequannent Melaku Sharew Sharew, Neeraj Sharma, Bereket Beyene Shashamo, Maryam Shayan, Rahim Ali Sheikhi, Jiabin Shen, B Suresh Kumar Shetty, Pavanchand H Shetty, Jae Il Shin, Nebiyu Aniley Shitaye, K M Shivakumar, Parnian Shobeiri, Seyed Afshin Shorofi, Sunil Shrestha, Soraya Siabani, Negussie Boti Sidemo, Wudneh Simegn, Ehsan Sinaei, Paramdeep Singh, Robert Sinto, Md Shahjahan Siraj, Valentin Yurievich Skryabin, Anna Aleksandrovna Skryabina, David A Sleet, Chandan S N, Bogdan Socea, Marco Solmi, Yonatan Solomon, Yi Song, Raúl A R C Sousa, Ireneous N Soyiri, Mark A Stokes, Muhammad Suleman, Rizwan Suliankatchi Abdulkader, Jing Sun, Rafael Tabarés-Seisdedos, Seyyed Mohammad Tabatabaei, Mohammad Tabish, Ensiyeh Taheri, Moslem Taheri Soodejani, Mircea Tampa, Ker-Kan Tan, Ingan Ukur Tarigan, Md Tariqujjaman, Nathan Y Tat, Vivian Y Tat, Arash Tavakoli, Belay Negash Tefera, Yibekal Manaye Tefera, Gebremaryam Temesgen, Mohamad-Hani Temsah, Pugazhenthan Thangaraju, Rekha Thapar, Nikhil Kenny Thomas, Jansje Henny Vera Ticoalu, Marius Belmondo Tincho, Amir Tiyuri, Munkhsaikhan Togtmol, Marcos Roberto Tovani-Palone, Mai Thi Ngoc Tran, Sana Ullah, Saif Ullah, Irfan Ullah, Srikanth Umakanthan, Bhaskaran Unnikrishnan, Era Upadhyay, Sahel Valadan Tahbaz, Pascual R Valdez, Tommi Juhani Vasankari, Siavash Vaziri, Massimiliano Veroux, Dominique Vervoort, Francesco S Violante, Vasily Vlassov, Linh Gia Vu, Yasir Waheed, Yanzhong Wang, Yuan-Pang Wang, Cong Wang, Taweewat Wiangkham, Nuwan Darshana Wickramasinghe, Abay Tadesse Woday, Ai-Min Wu, Gahin Abdulraheem Tayib Yahya, Seyed Hossein Yahyazadeh Jabbari, Lin Yang, Sanni Yaya, Arzu Yigit, Vahit Yiğit, Eshetu Yisihak, Naohiro Yonemoto, Yuyi You, Mustafa Z Younis, Chuanhua Yu, Ismaeel Yunusa, Hossein Yusefi, Mazyar Zahir, Sojib Bin Zaman, Iman Zare, Kourosh Zarea, Mikhail Sergeevich Zastrozhin, Zhi-Jiang Zhang, Yunquan Zhang, Arash Ziapour, Sanjay Zodpey, Mohammad Zoladl, George C Patton, Rebecca Q Ivers

## Abstract

**Background:**

Globally, transport and unintentional injuries persist as leading preventable causes of mortality and morbidity for adolescents. We sought to report comprehensive trends in injury-related mortality and morbidity for adolescents aged 10–24 years during the past three decades.

**Methods:**

Using the Global Burden of Disease, Injuries, and Risk Factors 2019 Study, we analysed mortality and disability-adjusted life-years (DALYs) attributed to transport and unintentional injuries for adolescents in 204 countries. Burden is reported in absolute numbers and age-standardised rates per 100 000 population by sex, age group (10–14, 15–19, and 20–24 years), and sociodemographic index (SDI) with 95% uncertainty intervals (UIs). We report percentage changes in deaths and DALYs between 1990 and 2019.

**Findings:**

In 2019, 369 061 deaths (of which 214 337 [58%] were transport related) and 31·1 million DALYs (of which 16·2 million [52%] were transport related) among adolescents aged 10–24 years were caused by transport and unintentional injuries combined. If compared with other causes, transport and unintentional injuries combined accounted for 25% of deaths and 14% of DALYs in 2019, and showed little improvement from 1990 when such injuries accounted for 26% of adolescent deaths and 17% of adolescent DALYs. Throughout adolescence, transport and unintentional injury fatality rates increased by age group. The unintentional injury burden was higher among males than females for all injury types, except for injuries related to fire, heat, and hot substances, or to adverse effects of medical treatment. From 1990 to 2019, global mortality rates declined by 34·4% (from 17·5 to 11·5 per 100 000) for transport injuries, and by 47·7% (from 15·9 to 8·3 per 100 000) for unintentional injuries. However, in low-SDI nations the absolute number of deaths increased (by 80·5% to 42 774 for transport injuries and by 39·4% to 31 961 for unintentional injuries). In the high-SDI quintile in 2010–19, the rate per 100 000 of transport injury DALYs was reduced by 16·7%, from 838 in 2010 to 699 in 2019. This was a substantially slower pace of reduction compared with the 48·5% reduction between 1990 and 2010, from 1626 per 100 000 in 1990 to 838 per 100 000 in 2010. Between 2010 and 2019, the rate of unintentional injury DALYs per 100 000 also remained largely unchanged in high-SDI countries (555 in 2010 *vs* 554 in 2019; 0·2% reduction). The number and rate of adolescent deaths and DALYs owing to environmental heat and cold exposure increased for the high-SDI quintile during 2010–19.

**Interpretation:**

As other causes of mortality are addressed, inadequate progress in reducing transport and unintentional injury mortality as a proportion of adolescent deaths becomes apparent. The relative shift in the burden of injury from high-SDI countries to low and low–middle-SDI countries necessitates focused action, including global donor, government, and industry investment in injury prevention. The persisting burden of DALYs related to transport and unintentional injuries indicates a need to prioritise innovative measures for the primary prevention of adolescent injury.

**Funding:**

Bill & Melinda Gates Foundation.

## Introduction

Injuries are a substantial yet neglected cause of mortality, and the millions of injury-related deaths that occur each year reflect large disparities in terms of gender, race, and socioeconomic status.^1^, ^2^ Among adolescents (age 10–24 years), injuries are the leading cause of death, claiming more lives than communicable or non-communicable diseases, nutritional or maternal health causes, or self-harm.^3^ Injuries also cause substantial disability globally. In 2019, the leading causes of injury-related disability-adjusted life-years (DALYs) for all ages were road injuries (ranked 7th) and falls (ranked 21st), and for adolescents the leading causes were road-injuries (ranked 1st), with falls ranked 22nd and drowning ranked 23rd (compared with 46th for all ages; for definitions, see the appendix p 1).^4^

Despite the potential lifelong effect of injuries (including those to the brain) acquired in adolescence on future health and wellbeing, physical mobility, education, and employment, there has also been far less focus on disability attributed to injury in adolescents than in younger children and adults.^5^, ^6^ Patterns of injury in adolescents differ from those in younger age groups, and adolescence represents a developmental transition point for injury risk, owing to factors such as increased independence and elevated tendencies towards risk-taking.^3^, ^7^, ^8^ However, until now, no study has systematically examined patterns of injury in this specific age group.

**Research in context**

**Evidence before this study**

We searched PubMed and Embase on Dec 15, 2021 for studies published in English between Jan 1, 1990 and Dec 31, 2019, which explored transport injury, unintentional injury, or both, burden among adolescents from a global perspective. We used the search terms injur* AND (“transport” OR unintentional) AND (“death” OR “mortality” OR “morbidity” OR “disability”) AND (“adolescen*” or “young people”) AND (“global” OR “world” OR “international”). Despite injuries being the leading cause of death in adolescents globally, and a life phase where injury deaths and Disability Adjusted Life Years (DALYs) change markedly, there is a striking absence of focus on adolescent injury in the published literature to now. Although we found adolescent-specific global research exploring all-cause mortality and progress in health and wellbeing indicators, these did not disaggregate injury by specific mechanism or intent. We also found several publications from the Global Burden of Disease Study 2017 reporting injury morbidity and mortality across the life course and the impact of sociodemographic index (SDI) on all-age injury-related DALYs, but these did not focus on adolescents. Other identified studies included a location, injury mechanism, or age-group focus, but we did not identify any studies that explored transport and unintentional injury mortality and morbidity among adolescents globally.

**Added value of this study**

To the best of our knowledge, this is the first comprehensive global analysis of transport and unintentional injury-related morbidity and mortality among adolescents aged 10–24 years. This study provides insight into trends and pace of change in cause-specific injury mortality and morbidity by country, sex, and age band. As such, this study identifies both areas of success and challenges regarding the reduction of injury-related harms within this age group. We found reductions in transport and unintentional injury fatalities in most SDI quintiles, but there have been large increases in the absolute number of transport and unintentional injury fatalities in low-SDI countries, particularly in those aged 20–24 years. Reductions in unintentional injury DALY rates for adolescents all but ceased in high-SDI countries between 2010 and 2019 if compared with progress made between 1990 and 2010.

**Implications of all the available evidence**

Transport and unintentional injury-related harms constitute a substantial disease burden in adolescents. Although progress has been made in reducing the rate of transport and unintentional injury deaths and DALYs in adolescents, this progress conceals concerning trends. Increasing absolute numbers of injury-related deaths and DALYs in low-SDI nations indicate a growing population of adolescents at risk of injury. Stalled progress in reducing injury in adolescents in high-SDI nations, requires an increased commitment and new approaches. Globally, a focus on primary and secondary prevention of injury is required to reduce both deaths and disability, particularly for high-lethality injuries such as drowning. The irregular but devastating injury burden owing to forces of nature and increasing deaths and DALYs for injuries related to environmental heat and cold exposure in high-SDI nations in 2010–19 are further evidence of the need for investment in disaster risk reduction strategies and action on climate change. Injuries are highly preventable and yet there is a complete absence of investment in the prevention of injury-related harms for adolescents from global donors, particularly in the context of the shifting burden of injury in low–SDI and low–middle-SDI countries.

Adolescence is a neglected life stage, in terms of both injury prevention and other related aspects of health,^6^ such as the risk of physical and cognitive injury associated with adolescent undernutrition,^9^ rising rates of antimicrobial resistance affecting recovery from traumatic injury,^10^ and the poor viability of anti-venom therapies in adolescents.^11^ Injuries are preventable, and investment in the prevention of injury-related harms for adolescents will enhance health and wellbeing, improve socioeconomic growth and development, and contribute towards the achievement of the UN Sustainable Development Goals (SDGs).^12^ Beyond the health and wellbeing goal (SDG 3) and two specific road safety targets (SDG 3.6 and SDG 11.2), strategies to reduce injury risk could have substantial effects on the realisation of SDG targets, such as SDG 10 (reduced inequalities), SDG 5 (gender equality), and SDG 11 (sustainable cities and communities).^13^

Investments to prevent injury-related harms in adolescents require a detailed understanding of the type of injuries involved, and how the rates and numbers of fatal and non-fatal injuries vary over time and by geography and country-level income. The aim of this study was to describe the pattern of mortality and morbidity from transport injuries and unintentional injuries in adolescents and to report trends between 1990 and 2019 using the Global Burden of Diseases, Injuries and Risk Factors (GBD) Study 2019**.**^4^ This Article was produced as part of the GBD Collaborator Network and in accordance with the GBD protocol.

## Methods

### Participants, study design, and data sources

We defined adolescence as age 10–24 years to reflect adolescent growth and popular understandings of this life phase.^14^

The methods for the GBD study,^4^ including the burden of injuries,^15^ have been published elsewhere. In brief, the GBD study is a comprehensive assessment providing time trends for a mutually exclusive and collectively exhaustive list of diseases and injuries. GBD 2019 estimated the incidence, prevalence, mortality, years of life lost (YLLs), years lived with disability (YLDs), and DALYs that were caused by 369 diseases and injuries across 204 countries and territories.^4^ GBD input data have been extracted from a wide range of sources, including censuses and household surveys, civil registration and vital statistics, disease registries, data for health-service use, air-pollution monitors, satellite imaging, and disease notifications.^4^

Injuries are one of three broad categories of causes of death and injury used in the GBD hierarchy, the others being non-communicable diseases, and the group of communicable, maternal, neonatal, and nutritional diseases (appendix pp 1–4).^15^ Case definitions for injury, including the International Classification of Diseases (ICD) external cause codes used to identify injury, are in the appendix (pp 1–4).

GBD 2019 complies with the Guidelines for Accurate and Transparent Health Estimates Reporting (GATHER) statement.^16^

### Data inclusions for injury

Outcome measures were the absolute numbers and rates per 100 000 of deaths and DALYs. From the GBD Study hierarchy of injury cause codes, we downloaded the transport injuries group (C.1), which is further delineated into road injuries or other transport injuries, and the unintentional injury grouping (C.2), which is further delineated into the following mechanisms: falls; drowning; fire, heat, and hot substances; poisoning; exposure to mechanical forces; adverse effects of medical treatment; animal contact; foreign body; environmental heat and cold exposure; exposure to forces of nature; and other unintentional injuries (appendix pp 1–4). Where possible, further disaggregation to level 4 of the hierarchy is provided for road injuries and selected unintentional injury mechanisms (eg, animal contact is further delineated into venomous-animal contact and non-venomous-animal contact; appendix pp 1–4). Injuries caused by self-harm, interpersonal violence, conflict, terrorism, execution, and police conflict were excluded.

### Data variables

Our analysis was stratified by sex, 5-year age group (10–14 years, 15–19 years, and 20–24 years), country, and sociodemographic index (SDI; a summary indicator of social and economic conditions that are strongly correlated with health outcomes).^4^ The derivation of SDIs has been described in detail elsewhere.^17^ We used a country's 2019 SDI throughout this study (appendix pp 5–9). SDIs are grouped into quintiles for analysis: low, low–middle, middle, middle–high, and high.

### Data analysis

All the GBD 2019 data for this study were from http://ghdx.healthdata.org/gbd-results-tool and imported into Stata (version 16.1). Absolute numbers and age-standardised rates per 100 000 population for deaths and DALYs were downloaded by sex, age group, injury mechanism, and SDI, alongside 95% uncertainty intervals (UIs). Trends over time comprised the percentage change in absolute numbers and rates between 1990 and 2019. Additionally, we reported the pace of change across the two predefined GBD time periods: 1990–2010 and 2010–2019. Exposure to forces of nature has shown extreme variation between years (appendix p 48), so quantifying change as simply the differences between 1990 and 2010 and 2019, was not meaningful given the extreme spike of deaths and DALYs in 2010, therefore change on this individual cause has not been reported. The percentage-change graph for this mechanism is in the appendix (p 48) for completeness.

### Role of the funding source

The funders of this study had no role in study design, data collection, data analysis, data interpretation, writing of the manuscript, or decision to submit.

## Results

The GBD Study reported that in 1990, for adolescents globally, transport and unintentional injuries accounted for 518 005 (26·4%) of 1 963 681 deaths and 41 814 820 (17·1%) of 244 507 616 DALYs, whereas in 2019, they accounted for 369 061 (24·8%) of 1 490 988 deaths and 31 121 600 (13·6%) of 229 251 808 DALYs (table 1). Of these, in 1990, 271 772 (13·8%) of 1 963 681 deaths were caused by transport injuries, whereas in 2019, the absolute number of these had decreased to 214 337, although these deaths accounted for an increased proportion of all-cause mortality (14·4%). The proportion of global transport injury deaths occurring in low-SDI and low–middle-SDI countries combined increased from 74 713 (27·5%) of 271 772 deaths in 1990 to 100 102 (46·7%) of 214 337 deaths in 2019. From 1990 to 2019, transport injury DALYs declined globally, whereas increases were reported in low-SDI and low–middle-SDI countries (table 1).

**Table 1:** Number and proportion of adolescent deaths and disability-adjusted life-years by SDI in 1990 and 2019

		**All causes for adolescents**	**Transport and unintentional injuries combined in adolescents**		**Transport injuries in adolescents**			**Unintentional injuries in adolescents**		
		n	n	% all causes in adolescents	n	% all causes in adolescents	% transport injury by SDI quintile	n	% all causes in adolescents	% unintentional injury by SDI quintile
**Deaths**										
All										
	1990	1 963 681	518 005	26·4%	271 772	13·8%	100·0%	246 233	12·5%	100·0%
	2019	1 490 988	369 061	24·8%	214 337	14·4%	100·0%	154 725	10·4%	100·0%
High SDI										
	1990	113 745	54 913	48·3%	41 019	36·1%	15·1%	13 894	12·2%	5·6%
	2019	64 923	21 963	33·8%	16 611	25·6%	7·8%	5351	8·2%	3·5%
Middle–high SDI										
	1990	251 965	95 461	37·9%	52 976	21·0%	19·5%	42 485	16·9%	17·3%
	2019	120 481	44 071	36·6%	26 776	22·2%	12·5%	17 295	14·4%	11·2%
Middle SDI										
	1990	607 343	202 796	33·4%	102 886	16·9%	37·9%	99 910	16·5%	40·6%
	2019	375 556	116 146	30·9%	70 690	18·8%	33·0%	45 456	12·1%	29·4%
Low–middle SDI										
	1990	630 671	117 900	18·7%	51 012	8·1%	18·8%	66 888	10·6%	27·2%
	2019	500 856	111 876	22·3%	57 328	11·4%	26·7%	54 548	10·9%	35·3%
Low SDI										
	1990	358 885	46 629	13·0%	23 701	6·6%	8·7%	22 928	6·4%	9·3%
	2019	428 154	74 736	17·5%	42 774	10·0%	20·0%	31 961	7·5%	20·7%
**Disability-adjusted life-years**										
All										
	1990	244 507 616	41 814 820	17·1%	20 244 278	8·3%	100·0%	21 570 542	8·8%	100·0%
	2019	229 251 808	31 121 600	13·6%	16 233 631	7·1%	100·0%	14 887 970	6·5%	100·0%
High SDI										
	1990	21 375 098	4 645 680	21·7%	2 982 541	14·0%	14·7%	1 663 139	7·8%	7·7%
	2019	18 187 918	2 199 400	12·1%	1 226 415	6·7%	7·6%	972 985	5·3%	6·5%
Middle–high SDI										
	1990	36 678 524	7 918 213	21·6%	3 937 569	10·7%	19·5%	3 980 644	10·9%	18·5%
	2019	24 068 040	3 871 488	16·1%	2 014 784	8·4%	12·4%	1 856 704	7·7%	12·5%
Middle SDI										
	1990	77 226 048	15 901 807	20·6%	7 582 829	9·8%	37·5%	8 318 978	10·8%	38·6%
	2019	60 399 016	9 475 380	15·7%	5 252 382	8·7%	32·4%	4 222 999	7·0%	28·4%
Low–middle SDI										
	1990	70 408 216	9 519 718	13·5%	3 905 194	5·5%	19·3%	5 614 524	8·0%	26·0%
	2019	69 492 544	9 282 480	13·4%	4 433 812	6·4%	27·3%	4 848 669	7·0%	32·6%
Low SDI										
	1990	38 683 968	3 804 800	9·8%	1 823 110	4·7%	9·0%	1 981 690	5·1%	9·2%
	2019	56 954 928	6 269 259	11·0%	3 294 650	5·8%	20·3%	2 974 610	5·2%	20·0%

Data are n or %. SDI=socio-demographic index.

In 2019, transport injuries represented a fatality rate of 11·5 (95% UI 10·4 to 12·6) per 100 000 and transport injury death rates have declined by –34·4% (–41·4 to –27·9) from 17·5 to 11·5 per 100 000 since 1990. DALYs owing to transport injuries amounted to 16·2 million (95% UI 14·7 million to 17·8 million), a rate of 871·9 per 100 000 (789·9 to 965·5). Transport injury DALY rates per 100 000 have declined globally by 33·3% (95% UI –39·9 to –27·4) since 1990 (table 2).

**Table 2:** Injury-related mortality and disability-adjusted life-years in adolescents by mechanism of injury, 1990–2019

		**Absolute number of adolescent injury-related burden in 2019 (95% UI)**	**Percentage change in number of adolescents, 1990–2019 (95% UI)**	**Number of adolescents per 100 000 population, 2019 (95% UI)**	**Percentage change in number of adolescents per 100 000 population, 1990–2019 (95% UI)**
**Deaths**					
Transport injuries		214 337 (192 798 to 235 285)	−21·1% (−29·5 to −13·3)	11·5 (10·4 to 12·6)	−34·4% (−41·4 to −27·9)
	Road injuries	200 113 (180 274 to 220 461)	−21·4% (−29·8 to −13·5)	10·7 (9·7 to 11·8)	−34·6% (−41·6 to −28·1)
	Other transport injuries	14 224 (11 910 to 16 203)	−17·6% (−28·0 to −5·4)	0·8 (0·6 to 0·9)	−31·4% (−40·1 to −21·3)
Unintentional injuries		154 725 (136 607 to 172 673)	−37·2% (−42·7 to −30·2)	8·3 (7·3 to 9·3)	−47·7% (−52·3 to −41·9)
	Adverse effects of medical treatment	5341 (4160 to 6583)	−23·5% (−33·6 to −7·6)	0·3 (0·2 to 0·4)	−36·4% (−44·8 to −23·1)
	Animal contact	13 552 (8975 to 17 062)	−16·0% (−32·7 to 24·7)	0·7 (0·5 to 0·9)	−30·2% (−44·0 to 3·7)
	Drowning	45 391 (41 069 to 50 616)	−50·4% (−55·6 to −44·2)	2·4 (2·2 to 2·7)	−58·7% (−63·1 to −53·6)
	Environmental heat and cold exposure	2123 (1261 to 2628)	−47·0% (−54·9 to −39·1)	0·1 (0·1 to 0·1)	−43·9% (−54·8 to −34·0)
	Exposure to forces of nature	1565 (1421 to 1722)	−87·8% (−87·8 to −87·8)	0·1 (0·1 to 0·1)	−89·9% (−89·9 to −89·9)
	Exposure to mechanical forces	16 721 (13 470 to 19 568)	−28·8% (−41·4 to −14·6)	0·9 (0·7 to 1·1)	−40·8% (−51·3 to −29·0)
	Falls	23 774 (20 315 to 27 097)	−16·0% (−28·9 to −0·8)	1·3 (1·1 to 1·5)	−30·1% (−40·8 to −17·5)
	Fire, heat, and hot substances	12 527 (8641 to 16 754)	−24·0% (−42·4 to 10·1)	0·7 (0·5 to 0·9)	−36·8% (−52·1 to −8·4)
	Foreign body	6737 (6088 to 7517)	−2·8% (−13·0 to 71)	0·4 (0·3 to 0·4)	−19·1% (−27·7 to −10·9)
	Poisonings	8755 (7293 to 9865)	−30·3% (−41·5 to −20·5)	0·5 (0·4 to 0·5)	−42·0% (−51·3 to −33·9)
	Other unintentional injuries	18 238 (14 110 to 20 692)	−32·6% (−45·7 to −20·7)	1·0 (0·8 to 1·1)	−55·9% (−62·4 to −49·4)
**Disability-adjusted life-years**					
Transport injuries		16 233 631 (14 706 076 to 17 809 534)	−19·8% (−27·8 to −12·7)	871·9 (789·9 to 956·5)	−33·3% (−39·9 to −27·4)
	Road injuries	15 110 183 (13 667 679 to 16 591 872)	−20·1% (−28·3 to −12·9)	811·6 (734·1 to 891·1)	−33·5% (−40·3 to −27·5)
	Other transport injuries	1 123 448 (959 314 to 1 278 037)	−16·4% (−25·9 to −5·4)	60·3 (51·5 to 68·6)	−30·5% (−38·4 to −21·3)
Unintentional injuries		14 887 970 (12 988 833 to 16 873 351)	−31·0% (−36·2 to −24·9)	799·6 (697·6 to 906·3)	−42·6% (−46·9 to −37·5)
	Adverse effects of medical treatment	393 423 (311 539 to 479 087)	−22·5% (−32·8 to −7·2)	21·1 (16·7 to 25·7)	−35·5% (−44·1 to −22·8)
	Animal contact	1 112 804 (787 384 to 1 385 295)	−15·4% (−30·4 to 19·9)	59·8 (42·3 to 74·4)	−29·6% (−42·1 to −0·2)
	Drowning	3 271 033 (2 959 200 to 3 645 410)	−50·7% (−55·9 to −44·7)	175·7 (158·9 to 195·8)	−59·0% (−63·3 to −54·0)
	Environmental heat and cold exposure	311 482 (240 031 to 396 449)	−29·9% (−36·0 to −23·3)	16·7 (12·9 to 21·3)	−41·7% (−46·8 to −36·2)
	Exposure to forces of nature	233 717 (187 540 to 301 272)	−76·3% (−79·9 to −70·7)	12·6 (10·1 to 16·2)	−80·3% (−83·3 to −75·6)
	Exposure to mechanical forces	1 811 505 (1 482 152 to 2 188 792)	−22·2% (−32·5 to −12·5)	97·3 (79·6 to 117·6)	−35·3% (−43·9 to −27·2)
	Falls	3 303 744 (2 701 281 to 4 029 961)	−8·4% (−16·9 to 0·4)	177·4 (145·1 to 216·4)	−23·8% (−–30·8 to −16·5)
	Fire, heat, and hot substances	1 226 401 (914 069 to 1 581 956)	−22·9% (−37·9 to 1·0)	65·9 (49·1 to 85·0)	−35·8% (−48·4 to −16·0)
	Foreign body	765 234 (644 476 to 922 981)	0·1% (−6·8 to 6·6)	41·1 (34·6 to 49·6)	−16·7% (−22·5 to −11·3)
	Poisonings	696 767 (597 906 to 779 864)	−28·8% (−39·1 to −19·8)	37·4 (32·1 to 41·9)	−40·8% (−49·3 to −33·3)
	Other unintentional injuries	1 761 860 (1 434 188 to 2 068 144)	−27·1% (−38·2 to −17·6)	94·6 (77·0 to 111·1)	−39·4% (−48·6 to −31·4)

Data are n (95% UI) or % (95% UI). UI=uncertainty interval.

If assessed against SDI, the absolute number of transport injuries has declined in the middle and upper two SDI quintiles, but has increased in the low–middle-SDI and low-SDI quintiles; with an increase of 80·5% (95% UI 53·4 to 113·3), from 23 701 deaths to 42 774 deaths in low-SDI countries. These increases in fatal transport injuries in low–middle-SDI countries were driven largely by increased absolute numbers of road injuries (13·9% [95% UI –1·0 to 30·0]). In low-SDI countries, there were large increases in the absolute numbers of both road injuries (81·6% [95% UI 53·1 to 114·6) and other transport injuries (66·8% [95% UI 33·9 to 103·9]; appendix pp 10–11). Absolute numbers of transport injury DALYs have also increased in low–middle-SDI and low-SDI countries (appendix pp 12–13). The breakdown of road injury deaths into their component parts (ie, pedestrian, cyclist, motorcyclist, motor vehicle, and other) are shown in the appendix (pp 14–15).

Rates per 100 000 of both deaths and DALYs owing to transport injuries are substantially higher among males (17·9 deaths per 100 000 in males *vs* 4·8 in females) than females (1335 DALYs per 100 000 in males *vs* 386 in females). The reduction in the rate of transport injuries between 1990 and 2019 was higher among adolescent females than for males for both deaths and DALYs (appendix pp 16–17).

For adolescents, the greatest reduction in the rate of transport-related injury deaths between 1990 and 2019 was in 10–14-year-olds (–44·5% [95% UI –51·0 to –37·5]; from 7·8 to 4·3 per 100 000), with reductions being less marked in those aged 15–19 years and 20–24 years (appendix pp 18–19). For transport injuries in low-SDI and low–middle-SDI countries, the fatality rate declined, and injury-related mortality in absolute numbers increased (appendix pp 11–12). These increases were most pronounced among both males (113·9% [95% UI 78·6 to 159·9]) and females (62·1% [29·2 to 100·5]) who were aged 20–24 years and lived in low-SDI countries (appendix pp 20–23).

**Figure 1:**
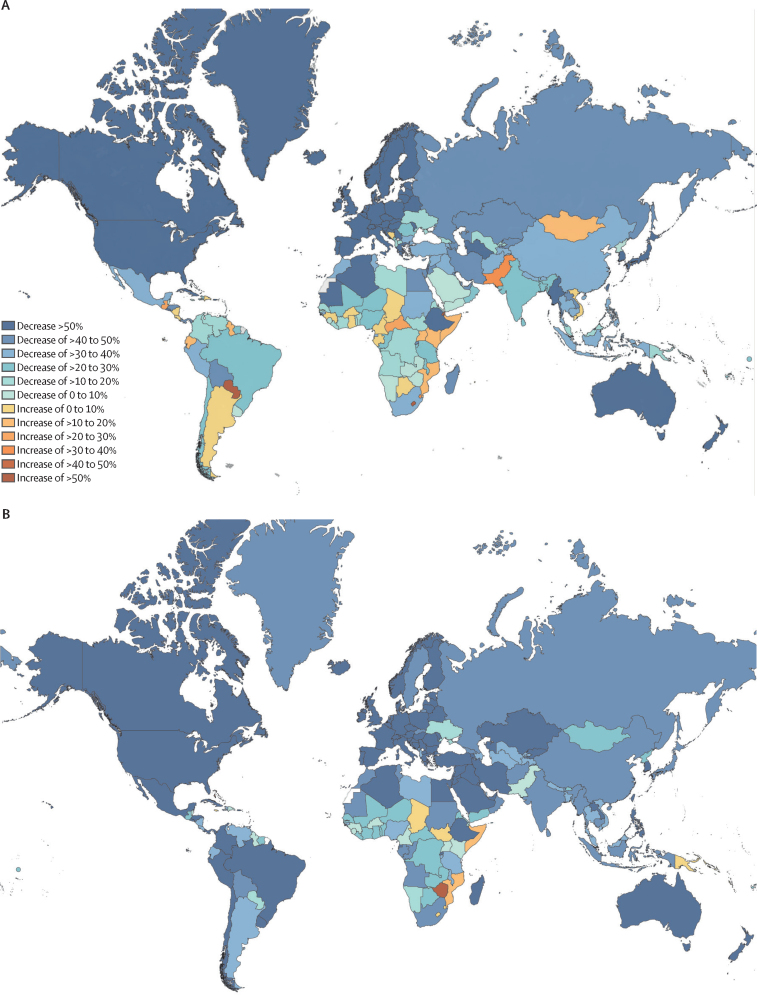
Percentage change in transport injury deaths (A) and unintentional injury deaths (B) per 100 000 population in adolescents aged 10–24 years from 1990 to 2019

Among adolescents, percentage changes between 1990 and 2019 for transport injury deaths by country are shown in figure 1A and these changes for transport injury DALYs are shown in the appendix (p 30). The greatest reductions (>80%) in death rates and DALYs were in Estonia and Portugal. Paraguay recorded the largest increases in both transport injury deaths (96·7% [95% UI 32·6–175·5]) and DALYs (86·8% [95% UI 29·0–157·4]). Substantial increases in transport injury deaths and DALYs among adolescents were also recorded in Djibouti, Lesotho, and the Seychelles.

Globally, transport injury death rates declined more rapidly in 2010–19 than in 1990–2010, and for all SDI quintiles other than in high-SDI countries in which the pace of change had slowed substantially in 2010–19 (–17·4% [95% UI –13·5 to –22·0], equivalent to –1·7% per annum) compared with 1990–2010 (–48·8% [95% UI –47·3 to –50·2], equivalent to –2·4% per annum; appendix p 31). The largest temporal change in transport injury DALYs occurred in low–middle-SDI countries, in which DALYs declined by 18·2% (95% UI –24·6 to –11·6; equivalent to –1·8% per annum) between 2010 and 2019, compared with a 3·8% reduction (95% UI –14·7 to 4·5; equivalent to –0·2% per annum) between 1990 and 2010 (appendix p 32).

For males, the pace of the decline in transport injury deaths and DALYs in high-SDI countries slowed dramatically in 2010–19 compared with 1990–2010 (deaths –49·5% [95% UI –51·0 to –47·8] in 1990–2010 *vs* –18·7% [95% UI –24·1 to –14·2] in 2010–19; DALYs –49·2% [95% UI –50·7 to –47·6] in 1990–2010 *vs* –18·4% [95% UI –23·5 to –14·1] in 2010–19; appendix pp 31–32). However, for all other SDI quintiles, higher percentage reductions were reported in 2010–19 than in 1990–2010. For females, the reduction in transport injury deaths and DALY rates plateaued in 2010–19 globally and for all SDI quintiles, except for declining death rates in middle–high and middle-SDI countries (appendix pp 31–32).

**Figure 2:**
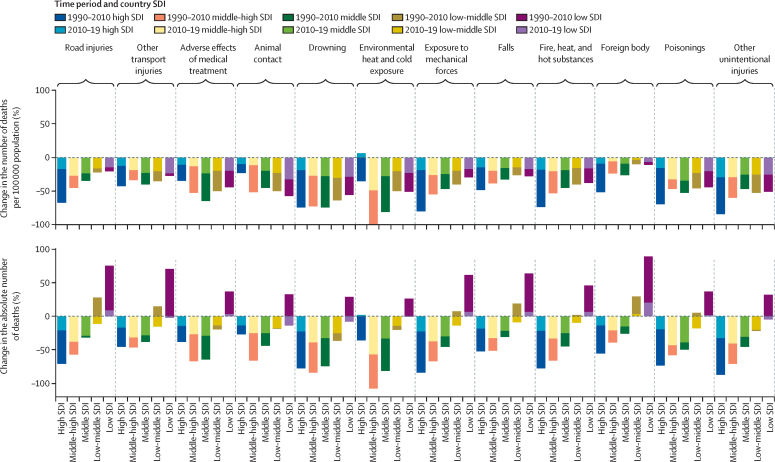
Percentage change in deaths per 100 000 population in adolescents aged 10–24 years from 1990 to 2010 and from 2010 to 2019, by injury mechanism and SDI quintile Deaths caused by exposure to forces of nature have not been included because of they extreme fluctuations over time. SDI=sociodemographic index.

Figure 2 shows the percentage change in the absolute numbers of deaths and mortality rate by SDI and level-3 cause of injury 1990–2019, and the appendix (p 33) depicts the same for DALYs. Improvements in the rates of road injuries and other transport injuries were recorded in the death and DALY rates per 100 000 population; however, the absolute numbers of deaths and DALYs increased in low-SDI and low–middle-SDI countries.

**Figure 3:**
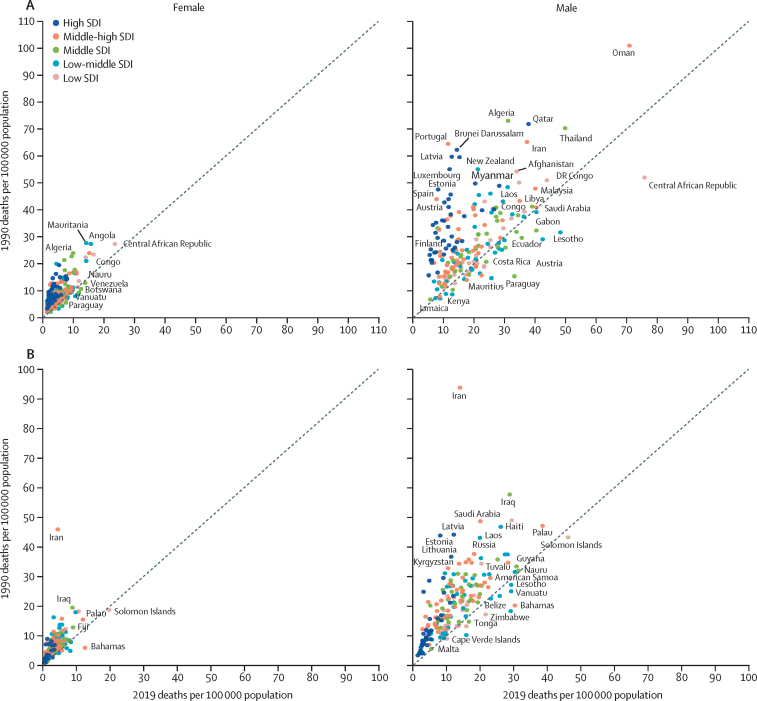
Injury death rates in 1990 versus 2019 in adolescents aged 10–24 years, by sex and country Transport injury death rates (A) and unintentional injury death rates (B). Countries shown above the diagonal dashed line had a lower rate in 2019 than in 1990. SDI=sociodemographic index.

Figure 3A shows the rate of transport-injury deaths, and the appendix (p 34) transport-injury DALYs, by sex, comparing 1990 and 2019 rates by country. Although many countries have made progress in reducing transport injury death rates and DALY rates for adolescent females, some countries had higher rates in 2019 (eg, Nauru, Venezuela, Botswana, Paraguay, and Vanuatu). For adolescent males, transport injury death rates and DALYs have increased in low and low–middle-SDI countries (eg, Central African Republic, Lesotho, Gabon, and Eswatini). The appendix (pp 35–38) shows transport injury deaths and DALY rates and percentage change between 1990 and 2019 by country.

For unintentional injuries in 1990, the GBD Study reported 246 233 (95% UI 225 434–263 748) adolescent deaths globally owing to unintentional injury, which accounted for 12·5% of all-cause adolescent mortality. By 2019, this proportion had reduced to 10·4%, with 154 725 (95% UI 136 607–172 673) deaths. Low-SDI and low–middle-SDI countries accounted for more than half of all unintentional injury deaths in adolescents globally in 2019 (86 509 [55·9%] of 154 725), compared with in 1990 (89 816 [36·5%] of 246 233). Unintentional injury-related DALYs for adolescents have declined, from 8·8% of all-cause DALYs in 1990 to 6·5% in 2019 (table 1).

The unintentional injury fatality rate for adolescents in 2019 was 8·3 (95% UI 7·3 to 9·3) per 100 000 population, a decline of –47·7% (95% UI –52·3% to –41·9) since 1990 (a fatality rate of 15·9 per 100 000). Drowning was the leading cause of unintentional injury-related deaths among adolescents in 2019 resulting in 45 400 deaths (95% UI 41 100 to 50 600), followed by falls (23 800 [95% UI 20 300 to 27 100]; table 2)

DALYs owing to unintentional injuries totalled 14·9 million (95% UI 13·0–16·9 million), a rate of 799·6 per 100 000 (95% UI 697·6–906·3). Globally, falls attracted the greatest DALY burden (3·30 million [95% UI 2·7 million–4·0 million]), followed by drowning (3·27 million [95% UI 3·0 million–3·6 million]; table 2).

The absolute number of adolescent deaths owing to unintentional injuries increased between 1990 and 2019 in low-SDI countries for all level-3 causes. Drowning was the leading cause of adolescent death in low-SDI countries, resulting in 6586 deaths (95% UI 5528–8044) followed by animal contact (3903 deaths [2544–5349]) and exposure to mechanical forces (3820 deaths [2932–5239]). Foreign body deaths (eg, asphyxia owing to choking on regurgitated food) increased by 30·1% (95% UI 9·5–49·5) in low–middle-SDI and by 103·2% (64·9–145·1) in low-SDI countries. Low-SDI countries showed high vulnerability to exposure to forces of nature, with a 389·5% increase in deaths associated with this mechanism among adolescents in 2019, compared with 1990 (appendix pp 10–11). Changes over time in deaths by disaggregation of select unintentional injury mechanisms are reported by SDI in the appendix (pp 24–26). Falls accounted for the largest absolute numbers of unintentional injury-related DALYs among adolescents in high-SDI, middle–high-SDI, and low-SDI countries. Drowning caused the highest absolute number of DALYs in middle and low–middle-SDI countries. Of concern are the increasing absolute numbers of DALYs caused by exposure to forces of nature and falls in low–middle SDI and increases in all level-3 causes of unintentional injury-related DALYs in low-SDI countries between 1990 and 2019 (appendix pp 12–13). Changes over time in DALYs by disaggregation of select unintentional injury mechanisms are reported by SDI in the appendix (pp 27–29). Absolute numbers and rates of deaths and DALYs were higher among males than females for all injury types, with the exceptions of fire, heat, and hot substances and adverse effects of medical treatment (appendix pp 16–17).

For 1990–2019 globally, the greatest reduction in the absolute number and rate of unintentional injury-related fatalities were recorded adolescents aged 10–14 years. Increases were seen in the absolute number of unintentional injury-related fatalities in low-SDI countries for all adolescent age groups (appendix pp 18–19).

Reductions in rates of unintentional injury-related fatalities among adolescents slowed in 2010–19 in all SDI quintiles other than low-SDI countries, which showed a marked reversal in the rate of unintentional injury fatalities, from a 1990–2010 increase of 146·0% (95% UI 115·3 to 189·2; equivalent to 7·3% per annum) to a 2010–19 reduction of –75·0% (–78·5% to –71·0%; equivalent to 7·5% per annum; appendix p 31), probably because of deaths from forces of nature 1990–2010 (appendix p 48). Globally, adolescent unintentional injury DALY rates have declined faster in 2010–19 (–35·8% [95% UI –39·9 to –31·7]) than in 1990–2010, largely driven by a reversal in the rate of adolescent unintentional injury DALYs in low-SDI countries, a decline of 69·8% (95% UI –73·4 to –65·8) for 2010–2019 compared with an increase of 119·3% (95% UI 94·7 to 152·3) for 1990–2010. This change is likely to be a function of a substantial number of injury events in the low-SDI quintile caused by forces of nature in 2010 (appendix p 48). However, in high-SDI countries, almost no progress has been made in reducing the rate of unintentional-injury-related DALYs among adolescents in 2010–19 (–0·2% [95% UI –4·5 to 3·6]; appendix p 32).

For males and females, progress in reducing unintentional injury death and DALY rates had slowed in 2010–19 in all SDI quintiles except for low SDI. Unintentional-injury DALY rates among females in high-SDI quintile countries increased for 2010–19 (8·7% [95% UI 4·6 to 11·5), despite significant reductions between 1990 and 2010 (–32·1% [95% UI –35·2 to –29·5] equivalent to –1·6% per annum; appendix p 32).

The percentage changes between 1990 and 2019 by country in unintentional injury deaths in adolescents are shown in figure 1B, and in unintentional injury DALY rates in adolescents in the appendix (p 39). The largest reductions in adolescent unintentional injury death rates were seen in South Korea (–86·8% [95% UI –89·6 to –79·2]) and Iran (–86·5% [–88·2% to –85·0%]), whereas the largest increases occurred in the Bahamas (63·2% [36·5% to 98·4%]) and Zimbabwe (59·6% [119·8% to 21·5%]). The appendix (pp 40–43) indicates increases in unintentional injury death rates for the Bahamas are being driven by large increases in animal contact fatalities (+232% in 2019, compared with 1990), whereas in Zimbabwe, unintentional injury death rates have increased since 1990 for all unintentional injury mechanisms. The appendix (pp 44–47) illustrates unintentional injury DALYs by unintentional injury mechanism and country. Large fluctuations are seen in the absolute number and rate of deaths and DALYs caused by exposure to forces of nature, coincident with large-scale disaster events, the occurrence of which is increasing (appendix p 48). Increases in the absolute number and rate of DALYs associated with animal contact, environmental heat and cold exposure, and falls among adolescents increased in the most recent decade (2010–19), particularly in high-SDI countries (appendix p 32).

The rate of unintentional injury deaths in shown in figure 3B and the rate of unintentional injury DALYs by sex is shown in the appendix (p 49), both with comparisons between 1990 and 2019, by country. For both females and males, unintentional injury death rates have declined dramatically in countries such as Iran and Iraq. Rates have increased for females in countries such as the Solomon Islands and The Bahamas, and for males in Zimbabwe and the Solomon Islands.

## Discussion

Adolescence is a sensitive point in the life course in which interventions can improve health and wellbeing during adolescence, into adulthood, and for the next generation.^7^ Most global and country-level programmes focused on adolescent health as it relates to mental health, communicable diseases, and reproductive health, despite the high incidence of unintentional and intentional injury among this age group.^18^ Recognition that road traffic injuries are the leading cause of death for adolescents aged 15–19 years,^19^ and the cost–benefit of implementing road safety interventions^20^ has led to the inclusion of unintentional injuries and road safety in the adolescent wellbeing framework^21^ published in 2020, with adolescents themselves joining the call for action through the Adolescents 2030 movement^22^ ahead of the UN Global Summit for Adolescent Wellbeing planned for 2023.

This study tracked trends in transport and unintentional injury mortality and morbidity in adolescents between 1990 and 2019. Our findings suggest that transport and unintentional injuries continue to be substantial causes of harm that have remained largely unchanged as a proportion of all-cause deaths and DALYs for adolescents since 1990. Although transport and unintentional injury mortality rates are declining globally, this result conceals variability and inequities by injury mechanism, time, SDI quintile, and outcome. In high-income countries, progress in reducing injury in adolescents has stalled, whereas injury is emerging as a growing cause of death and health-related disability in low–income and middle–income countries.^3^ This challenge necessitates global donor, government, and industry investment, which is largely inadequate for countries in the low-SDI quintile.^23^

Deaths and DALYs caused by transport injuries and most unintentional injury mechanisms are substantially higher among male than female adolescents. Similarly, between 1990 and 2019, reductions in the rate of transport injuries causing deaths and DALYs were both greater among adolescent females than males. However, our study has identified an increasing absolute number of transport and unintentional injuries among females in the high-SDI quintile and higher mortality and morbidity among females for injuries caused by fire, heat, and hot substances and the adverse effects of medical treatment, compared with males. Such findings indicate the importance of considering sex differences when developing injury prevention interventions for adolescents, including country-specific cultural and gender norms that might influence risk. Transport injuries accounted for the greatest proportion of deaths and DALYs among adolescents in this study. Preventing road injury in adolescence through the adoption of effective interventions that improve safety behaviours will have an effect that extends into adulthood, because road injuries are also the leading cause of DALYs among adults aged 25–49 years.^4^ Among adolescents aged 11–19 years, there is strong evidence that a suite of policies and practices reduce road traffic injuries.^13^ Graduated driver licensing schemes, enforcement of minimum drinking-age laws, lower blood alcohol content levels for novice drivers, wearing motorcycle and bicycle helmets, laws about seat-belt and helmet use, and reducing speed limits near to schools, residential areas, and play areas are interventions that have been effective in reducing injury-related harms.^24^ Although such interventions should be considered for integration into policy to reduce road traffic injuries, an absence of evidence of effectiveness in low–income and middle–income countries could be a barrier to implementation.^25^ Interventions applicable to all age groups, such as the enforcement of speed-limits and drink-driving bans, have been found to be effective in reducing road traffic deaths,^26^ and should also be prioritised.

Establishing public transport and active-transport infrastructure that prioritises safe travel is also crucial, and provides additional benefits for both adolescents (eg, physical activity) and the wider community (eg, cleaner air).^27^, ^28^, ^29^ As vulnerable road users, adolescents must be safeguarded as urbanisation and development increase motorised transport.^30^ Streets should be designed with children and adolescents in mind, and balance their need for safety on the roads with their need for accessibility and enjoyment, to promote their independent mobility.^31^ Cities such as Amsterdam, Addis Ababa, Bogota, Hanoi, and others have prioritised youth in their urban development and improvement without compromising children's safety or increasing road-traffic collisions.^32^

This study has identified drowning as the leading cause of unintentional injury-related deaths among adolescents, with a fatality rate almost double that of the next closest mechanism of injury (2·4 per 100 000 for drowning *vs* 1·3 per 100 000 for falls). WHO recognition and guidance in the prevention of drowning^33^, ^34^ has probably contributed to reductions between 1990 and 2019 in rates for death (–58·7%) and DALYs (–59·0%). However, these reductions must be considered with caution, as they exclude drownings related to boating and disasters.^35^ There is every expectation that the 2021 UN Resolution on Global Drowning Prevention^36^ will catalyse further action. However, it will be important to ensure that investment in interventions to prevent drowning in adolescents do not continue to lag behind those for very young children.^37^

Drowning is an injury linked strongly to the planetary driver of a changing climate.^38^ Findings from the current study start to identify additional ways in which the climate influences the risk of injury in adolescents. The devastating and increasing effects of injuries caused by forces of nature is noteworthy, particularly given the dramatic increase in the number of deaths and DALYs in low-SDI countries in the past 30 years. Similarly, we report increasing injury-related mortality and morbidity owing to environmental heat and cold exposure in high-SDI countries in 2010–19. Such findings add to the growing body of evidence regarding the need for global action on climate change,^39^ and the importance of tailoring disaster risk-reduction strategies to adolescents in the context of increasingly frequent and severe disasters.^40^

Globally, we have identified a slowing in the pace of reductions in transport and unintentional injury deaths and DALY rates in high-SDI countries in 2010–19, for both male and female adolescents. In most cases, reductions recorded between 2010 and 2019 are less than half of the achieved 1990–2010. Of concern, high-SDI countries also recorded an increase in DALYs owing to unintentional injury among females in 2010–19, which raises questions about further prioritisation of primary prevention while considering the benefit of adopting new approaches. The economic effects of adolescent injury and conversely, the cost–benefit associated with effective interventions, require further research, as these can be effective in catalysing policy change to further accelerate injury reduction.^41^

This study identifies increases in the relative burden of absolute numbers of transport and unintentional injury deaths and DALYS in adolescents from a high-income context to lower-SDI countries. Population growth is a contributor,^42^ but other factors also affect injury risk and outcomes in the low-SDI quintile, including low capacity for the tertiary care of injuries.^43^ The rise in the number of injuries is becoming increasingly visible as mortality and morbidity from other causes (eg, maternal and infectious diseases) declines.^3^ The absence of global development assistance for the prevention of adolescent injury in lower-income countries is a cause for concern in the context of the findings of this study.^23^

Increasing urbanisation has led to an increase in the number of vehicles on unsafe roads without adequate protection for vulnerable road users such as children and adolescents.^44^ Further investment in regulatory frameworks is needed to prevent transport-related injuries in lower-SDI countries, in which regulatory and societal responses to the prevention of injury often lag behind the pace of development or are poorly implemented.^2^, ^26^ A review of global non-motorised transportation plans found that although most countries had at least one relevant policy, these policies had not yet been adequately implemented or assessed for effectiveness.^45^

Tanzania is a low-SDI country in which investment in school-area interventions aimed at slowing traffic speed and separating child pedestrians from vehicles, alongside the provision of site-specific road safety education, has reduced the number of road-traffic injuries among primary school children (age 7–14 years).^46^ Other similar city-based initiatives to protect adolescents, which are focused on speed reductions around schools, infrastructure modifications, and awareness raising, are being undertaken in six low–income and middle–income countries.^47^ The feasibility of implementing such interventions should be also explored for older adolescents (aged 15–24 years). By contrast, Zimbabwe is a country in which the rate of fatal unintentional injury has increased by 60% since 1990. Increases in all mechanisms of unintentional injury have been seen; however, the greatest increases were for deaths caused by exposure to mechanical forces (73%) or a foreign body (51%). The country faces a range of challenges, including economic issues, reduced life expectancy, increases in communicable and non-communicable diseases, and a shortage of health workers,^48^ that could affect injuries in adolescents in Zimbabwe. The persistent burden of injury-related disability in many SDI quintiles is concerning. Although this finding could represent improvements in the health system response to the treatment of injury-related harms, given residual injury rates, it is also a call to action to enhance investment in interventions aimed at primary prevention, including systems-level approaches.^49^ Such action is crucial, because beyond fatal effects, injury-related morbidity in adolescents can have devasting effects on individuals and their families, owing to the effects on health, physical mobility, and brain function that influence future education, employment, and income, and social stigma, partnering, and parenting.^20^, ^50^

By presenting transport and unintentional injuries together in this study, we highlighted the persistent burden of such injuries in the adolescent age group. The responsibility for the prevention of transport-related and unintentional injuries is likely to extend across different government agencies and sectors, therefore countries should map responsibility to relevant ministries and portfolios.

Our study is subject to the limitations of the GBD study methods, such as sparsity of data in low-income and middle-income countries.^4^, ^42^ Also, there are further challenges associated with the estimation of injuries within the GBD Study framework that have been reported previously.^15^ In brief, these include data limitations around quantifying non-fatal injury and full disability associated with multiple injuries, misclassification of injuries in regard to intent, and a paucity of data on the nature of injury measurement.^15^, ^51^ National estimates mask subnational disparities. In addition, data for the adolescent age group are of poorer quality than for other groups, such as children younger than 5 years and women in their childbearing years.^52^ There is a need to improve data collection entirely for adolescents, and to give priority to ensuring consistent use of injury and trauma coding in hospitals and medical facilities that is age-specific and meets a global standard, strengthening national surveillance systems for injuries, and the development of an improved global standard paediatric and adolescent-specific Injury Severity Score.^53^, ^54^ Our study explored level-3 causes of injury and provided the available level-4 data for unintentional injuries, although this was not disaggregated by sex or age. Finally, 2019 was the last timepoint in the GBD Study data before the COVID-19 pandemic. Future studies should explore the impact of COVID-19-related suppression strategies, such as lockdowns, on the rate of transport and unintentional injury in adolescents.

Transport and unintentional injuries among adolescents represent substantial causes of health burden, in terms of both the young lives lost and the lifelong impacts of disability. Despite reductions in mortality rates for injury, such injuries continued to account for a quarter of all adolescent deaths in 2019, just like they did in 1990. In high-SDI countries, progress has stalled in the past decade, necessitating a commitment to injury prevention for adolescents and the adoption of innovative approaches to primary prevention. Increasingly, the relative burden of adolescent injuries is higher in low-SDI countries because of demographic change, rapid urbanisation, and vulnerability to disasters, alongside other effects of climate change. The global donor community must prioritise investment in injury prevention interventions in low-SDI countries. Global commitment to mitigating the climate crisis is required, as is investment in disaster risk reduction strategies for adolescents in the context of increasingly frequent and severe disasters. For all countries, a better understanding of the economic effects of adolescent injury and the economic benefit of effective interventions is crucial to encourage investment and policy change.

## Data sharing

All GBD 2019 data are publicly available and can be downloaded via the Global Burden of Disease Results Tool (http://ghdx.healthdata.org/gbd-results-tool). The statistical code used for the GBD estimation is publicly available online (http://ghdx.healthdata.org/gbd-2019/code).
