# Correction to *Lancet Public Health* 2022; 7: e657–69

**DOI:** 10.1016/S2468-2667(22)00294-8

**Published:** 2022-11-30

*GBD 2019 Adolescent Transport and Unintentional Injuries Collaborators. Adolescent transport and unintentional injuries: a systematic analysis using the Global Burden of Disease Study 2019.* Lancet Public Health *2022; **7:** e657–69*—In this Article, in table 2, the deaths from foreign bodies percentage change in number of adolescents, 1990–2019 (95% UI) should have read –2·8% (–13·0 to 7·1), the Contributors and Declaration of interests have been corrected, and the appendix (author list and affiliation details) has been corrected. These corrections have been made as of Nov 30, 2022.

