# Supplementary material for: Adolescent transport and unintentional injuries: a systematic analysis using the Global Burden of Disease Study 2019
Source: Lancet Public Health. 2022 Jun 30;7(8):e657–69. doi: 10.1016/S2468-2667(22)00134-7 (PMC9329128; doi:10.1016/S2468-2667(22)00134-7)
Supplement: Supplementary appendix [file mmc1.pdf]

THE LANCET
Public Health

Supplementary appendix

This appendix formed part of the original submission and has been peer reviewed. We post it as supplied by the authors.

Supplement to: GBD 2019 Adolescent Transport and Unintentional Injuries Collaborators. Adolescent transport and unintentional injuries: a systematic analysis using the Global Burden of Disease Study 2019. *Lancet Public Health* 2022; published online June 29. https://doi.org/10.1016/S2468-2667(22)00134-7.

APPENDIX

Table of Contents

Table S1: Injury mechanism codes and case definitions within GBD Study Cause code hierarchy 1

Table S2: List of the 204 country/territories and their Socio Demographic Index (SDI) classification in 2019 5

Table S3: Injury-related mortality in adolescents aged 10-24 years. Absolute number of deaths in 1990 and 2019 and percentage change in absolute number between 1990 and 2019 by mechanism of transport and unintentional injuries 10

Table S4: Injury-related Disability Adjusted Life Years (DALYs) in adolescents aged 10-24 years. Absolute number of DALYs in 1990 and 2019 and percentage change in absolute number between 1990 and 2019 by mechanism of transport and unintentional injuries 12

Table S5: Level 4 breakdown of road injuries category, deaths absolute numbers and rates per 100,000 population 1990 and 2019, and percentage change between 1990 and 2019 by Socio Demographic Index (SDI) 14

Table S6: Tier 4 breakdown of road injuries category, DALYs absolute numbers and rates per 100,000 population 1990 and 2019, and percentage change between 1990 and 2019 by SDI 15

Table S7: Injury-related deaths and DALYs absolute numbers and rates per 100,000 population for males aged 10-24 years by mechanism of injury, transport injuries and unintentional injuries 2019 and percentage change 1990-2019 16

Table S8: Injury-related deaths and DALYs absolute numbers and rates per 100,000 population for females aged 10-24 years by mechanism of injury, transport injuries and unintentional injuries 2019 and percentage change 1990-2019 17

Table S9: Adolescent transport and unintentional injury-related mortality in absolute numbers and rates per 100,000 population overall and by age group, by socio-demographic index (SDI), and percentage change from 1990 to 2019. 18

Table S10: Injury-related mortality in absolute numbers and rates per 100,000 population for **males** and by age group, by socio-demographic index, transport injury and unintentional injury and trends over time 1990-2019 20

Table S11: Injury-related mortality in absolute numbers and rates per 100,000 population for **females** overall and by age group, by socio-demographic index, transport injury and unintentional injury and trends over time 1990-2019 22

Table S12: Level 4 breakdown of select unintentional injury mechanisms for adolescents 10-24 years, total deaths and deaths per 100,000 population, 1990 and 2019 and percentage change by SDI 24

Table S13: Level 4 breakdown of select unintentional injury mechanisms for adolescents 10-24 years, total DALYs and DALYs per 100,000 population, 1990 and 2019 and percentage change by SDI 27

Figure S1: Percentage change in transport injury DALYs per 100,000 population in adolescents from 1990 to 2019 30

Figure S2: Percentage change in transport injury and unintentional injury fatality rates in adolescents per 100,000 population overall and by sex and SDI, between 1990-2010 and 2010-2019 31

Figure S3: Percentage change in transport injury and unintentional injury disability adjusted life year (DALY) rates per 100,000 population overall and by sex and SDI, between 1990-2010 and 2010-2019 32

Figure S4: The percentage change in DALYs per 100,000 population from 1990 to 2019 for each injury mechanism by SDI group 33

Figure S5: Transport injury DALY rates per 100,000 population, 1990 vs 2019 by sex and country (coloured according to SDI), adolescents 10-24 years 34

Figure S6: Heatmap of transport injury DALYs and deaths rates and percentage change 1990-2019 by country, adolescents 10-24 years 35

Figure S7: Percentage change in unintentional injury DALYs per 100,000 population from 1990 to 2019 39

Figure S8: Heatmap of unintentional injury mechanism death rates and percentage change 1990-2019 by country, adolescents 10-24 years 40

Figure S9: Heatmap of unintentional injury mechanism DALY rates and percentage change 1990-2019 by country, adolescents 10-24 years 44

Figure S10: Exposure to forces of nature absolute number, and rate per 100,000 population of DALYs and deaths, annual trends and percentage change, 1990-2019 48

Figure S11: Unintentional injury DALY rates per 100,000 population, 1990 vs 2019 by sex and country (coloured according to SDI), adolescents 10-24 years 49

GATHER Checklist 50

GBD 2019 Adolescent Transport and Unintentional Injuries Collaborators – Authors and Affiliations 51

Authors' Contributions 66

Author Acknowledgements and Declarations of Interest 71

Table S1: Injury mechanism codes and case definitions within GBD Study Cause code hierarchy

Hierarchy	Cause	ICD 10 codes	Case definition (fatal)	Case definition (non-fatal)
A	Communicable, maternal, neonatal, and nutritional diseases			
B	Non-communicable diseases			
**C**	**Injuries**			
**C.1**	**Transport injuries**			
C1.1	Road injuries	V01-V04.99, V06-V80.929, V82-V82.9, V87.2-V87.3	Interaction with an automobile, motorcycle, pedal cycle or other vehicles resulting in death.	Interaction with an automobile, motorcycle, pedal cycle or other vehicles resulting in bodily damage.
C.1.1.1	*Pedestrian road injuries*	V01-V04.99, V06-V09.9	Interaction, as a pedestrian on the road, with an automobile, motorcycle, pedal cycle or other vehicles resulting in death.	Interaction, as a pedestrian on the road, with an automobile, motorcycle, pedal cycle or other vehicles resulting in bodily damage.
C.1.1.2	*Cyclist road injuries*	V10-V19.9	Accident, as a cyclist or passenger on a pedal cycle, resulting in death.	Accident, as a cyclist or passenger on a pedal cycle, resulting in bodily damage.
C.1.1.3	*Motorcyclist road injuries*	V20-V29.9	Accident, as a rider on a motorcycle, resulting in death.	Accident, as a rider on a motorcycle, resulting in bodily damage.
C.1.1.4	*Motor vehicle road injuries*	V30-V79.9, V87.2-V87.3	Accident, as a driver or passenger in a motor vehicle, resulting in death.	Accident, as a driver or passenger in a motor vehicle, resulting in bodily damage.
C.1.1.5	*Other road injuries*	V80-V80.929, V82-V82.9	Death resulting from being a driver or passenger of a vehicle not including automobiles, motorcycles, bicycles (ie, streetcar).	Bodily damage resulting from being a driver or passenger of a vehicle not including automobiles, motorcycles, bicycles (ie, streetcar).
C1.2	Other transport injuries	V00-V00.898, V05-V05.99, V81-V81.9, V83-V86.99, V88.2-V88.3, V90-V98.8	Interaction with a means of transport other than automobile, motorcycle, pedal cycle or other road vehicles resulting in death.	Interaction with a means of transport other than automobile, motorcycle, pedal cycle or other road vehicles resulting in bodily damage.
**C.2**	**Unintentional injuries**			
C.2.1	Falls	W00-W19.9	A sudden movement downwards due to slipping, tripping or other accidental movement which results in a person coming to rest	A sudden movement downward due to slipping, tripping or other accidental movement which results in a person coming to rest inadvertently on the

			inadvertently on the ground, floor or other lower level, resulting in death.	ground, floor or other lower level, resulting in tissue damage.
C.2.2	Drowning	W65-W70.9, W73-W74.9	Death that occurs as a result of immersion in water or another fluid.	Non-fatal immersion or submersion in water or another fluid, regardless of whether tissue damage has occurred. The subject can be resuscitated and has not suffered brain death.
C.2.3	Fire, heat, and hot substances	X00-X06.9, X08-X19.9	Death due to unintentional exposure to substances of high temperature sufficient to cause tissue damage on exposure, including bodily contact with hot liquid, solid or gas such as cooking stoves, smoke, steam, drinks, machinery, appliances, tools, radiators and objects radiating heat energy.	Unintentional exposure to substances of high temperature sufficient to cause tissue damage on exposure, including bodily contact with hot liquid, solid or gas such as cooking stoves, smoke, steam, drinks, machinery, appliances, tools, radiators and objects radiating heat energy.
C.2.4	Poisonings	E86.02-E86.99, J70.5, X40-X44.9, X46-X49.9, Y10-Y14.9, Y16-Y19.9	Death resulting from accidental exposure to a non-infectious substance which contacts the body or enters into the body via inhalation, ingestion, injection or absorption and causes deranged physiological function of body and/or cellular injury/death.	Unintentional exposure to a non-infectious substance which contacts the body or enters into the body via inhalation, ingestion, injection or absorption and causes deranged physiological function of body and/or cellular injury/death.
C.2.4.1	*Poisoning by carbon monoxide*	E86.2-E86.29, E86.8-E86.89, J70.5, X47-X47.9	Death from exposure to carbon monoxide (CO) as identified based on carboxyhemoglobin levels (specified based on smoking status and age) or proximity to a confirmed CO poisoning case.	Non-fatal exposure to CO as identified based on carboxyhemoglobin levels (specified based on smoking status and age) or proximity to a confirmed CO poisoning case.
C.2.4.2	*Poisoning by other means*	E86.02-E86.19, E86.3-E86.7, E86.9-E86.99, X40-X44.9, X46-X46.9, X48-X49.9, Y10-Y14.9, Y16-Y19.9	Death resulting from accidental exposure to a non-infectious substance (other than CO) which contacts the body or enters into the body via inhalation, ingestion, injection or absorption and causes	Accidental exposure to a non-infectious substance (other than CO) which contacts the body or enters into the body via inhalation, ingestion, injection or absorption and causes deranged

			deranged physiological function of body and/or cellular injury/death.	physiological function of body and/or cellular injury/death.
C.2.5	Exposure to mechanical forces	W20-W38.9, W40-W43.9, W45.0-W45.2, W46-W46.2, W49-W52	Unintentional death resulting from contact with or threat of an (in)animate object, human or plant.	Unintentional bodily damage resulting from contact with or threat of an (in)animate object, human or plant.
C.2.5.1	*Unintentional firearm injuries*	W32-W34.9	Unintentional death resulting from contact with a firearm.	Unintentional bodily damage resulting from contact with a firearm.
C.2.5.2	*Other exposure to mechanical forces*	W20-W31.9, W35-W38.9, W40-W43.9, W45.0-W45.2, W46-W46.2, W49-W52	Unintentional death resulting from contact with or threat of an (in)animate object (not including a firearm), human or plant.	Unintentional bodily damage resulting from contact with or threat of an (in)animate object (not including a firearm), human or plant.
C.2.6	Adverse effects of medical treatment	D69.5-D69.59, D70.1-D70.2, D78-D78.89, D89.81-D89.813, E03.2, E06.4, E09-E09.9, E16.0, E23.1, E24.2, E27.3, E36-E36.8, E66.1, E87.0-E87.99, E89-E89.9, G21.0-G21.19, G24.0-G24.09, G25.1, G25.4, G25.6-G25.79, G62.0, G72.0, G93.7, G96.0, G96.11, G97-G97.9, H05.33-H05.339, H05.42-H05.53, H59-H59.89, H91.0-H91.09, H95-H95.9, I95.2-I95.81, I97-I97.9, J70-J70.4, J95-J95.9, K08.5-K08.59, K43-K43.9, K52.0, K62.7, K68.11, K91-K91.9, K94-K95.89, L23.3, L27.0-L27.1, L56.0-L56.1, L64.0, L76-L76.82, M10.2-M10.29, M87.1-M87.19, M96-M96.9, N14-N14.4, N30.4-N30.41, N46.021, N46.121, N52.2-N52.39, N65-N65.1, N99-N99.9, P93-P93.8, P96.2, P96.5, R50.2-R50.83, Y40-Y84.9, Y88-Y88.3, Z21.0, Z42-Z43.0, Z43.8-Z43.9, Z48-Z48.9, Z51-Z51.9, Z88-Z88.9, Z92-Z94.0, Z94.6, Z94.8-Z94.9, Z96-Z96.49, Z96.6-Z97.2, Z97.8-Z99.12, Z99.3-Z99.9	Death sustained as the result of undergoing a procedure, treatment, or other exposure to the health-care system. This exposure can occur in inpatient admission, outpatient facilities, emergency care, or during home treatment.	Short-term or long-term disability sustained as the result of undergoing a procedure, treatment, or other exposure to the health-care system. This exposure can occur in inpatient admission, outpatient facilities, emergency care, or during home treatment.
C.2.7	Animal contact	W52.0-W62.9, W64-W64.9, X20-X29.9	Death resulting from unintentionally being attacked, struck, impaled, bitten, stung, crushed, exposed to or stepped on by a non-human animal.	Bodily damage resulting from unintentionally being attacked, butted, impaled, bitten, stung, crushed, exposed to or stepped on by a non-human animal.

C.2.7.1	*Venomous animal contact*		Death resulting from unintentionally being bitten by, stung by, or exposed to a non-human venomous animal.	Bodily damage resulting from unintentionally being bitten by, stung by or exposed to a non-human venomous or poisonous animal.
C.2.7.2	*Non-venomous animal contact*	W52.0-W62.9, W64-W64.9, X20-X29.9	Death resulting from unintentionally being attacked, struck, impaled, crushed, exposed to or stepped on by a non-human animal.	Bodily damage resulting from unintentionally being attacked, struck, impaled, crushed, exposed to or stepped on by a non-human animal.
C.2.8	Foreign body	H02.81-H02.819, H44.6-H44.799, M60.2-M60.28, W44-W45, W45.3-W45.9, W75-W76.9, W78-W80.9, W83-W84.9	Unintentional death from an extraneous material or substance being within the body, including the airway, lungs, nose, and eyes.	Unintentional bodily damage from an extraneous material or substance being within the body, including the airway, lungs, nose, and eyes.
C.2.8.1	*Pulmonary aspiration and foreign body in airway*	W75-W76.9, W78-W80.9, W83-W84.9	Unintentional death from inhaling, swallowing or aspirating extraneous materials or substance that enters the airway or lungs.	Unintentional bodily damage from inhaling, swallowing or aspirating extraneous materials or substance that enters the airway or lungs.
C.2.8.2	*Foreign body in eyes*	H02.81-H02.819, H44.6-H44.799	NA	Unintentional damage from extraneous materials or substance in the orbital structure or eye.
C.2.8.3	*Foreign body in other body part*	M60.2-M60.28, W44-W45, W45.3-W45.9	Unintentional death from an extraneous material or substance being within the body, not including the airway, lungs or eyes.	Unintentional bodily damage from an extraneous material or substance being within the body, not including the airway, lungs or eyes.
C.2.9	Environmental heat and cold exposure	L55-L55.9, L58-L58.9, W88-W94.9, W97.9, W99-W99.9, X30-X32.9, X39-X39.9	Deaths resulting from exposure to high or low temperatures in the environment, not including contact with fire, heat, or hot substances.	Disability resulting from exposure to high or low temperatures in the environment, not including contact with fire, heat, or hot substances.
C.2.10	Exposure to forces of nature	X33-X38.9	Death resulting from an unforeseen and often sudden natural event such as a hurricane, earthquake, tsunami or tornado.	Bodily damage resulting from an unforeseen and often sudden natural event such as a hurricane, earthquake, tsunami or tornado.
C.2.11	Other unintentional injuries	W39-W39.9, W77-W77.9, W81-W81.9, W85-W87.9, X50-X58.9	Death resulting from unintentional injuries not included in transport injuries or elsewhere in unintentional injuries.	Disability resulting from unintentional injuries not included in transport injuries or elsewhere in unintentional injuries.

Table S2: List of the 204 country/territories and their Socio Demographic Index (SDI) classification in 2019

Country/Territory	SDI classification
Afghanistan	Low SDI
Benin	Low SDI
Bhutan	Low SDI
Burkina Faso	Low SDI
Burundi	Low SDI
Central African Republic	Low SDI
Chad	Low SDI
Comoros	Low SDI
Côte d'Ivoire	Low SDI
Democratic Republic of the Congo	Low SDI
Eritrea	Low SDI
Ethiopia	Low SDI
Gambia	Low SDI
Guinea	Low SDI
Guinea-Bissau	Low SDI
Haiti	Low SDI
Kiribati	Low SDI
Liberia	Low SDI
Madagascar	Low SDI
Malawi	Low SDI
Mali	Low SDI
Mozambique	Low SDI
Nepal	Low SDI
Niger	Low SDI
Papua New Guinea	Low SDI
Rwanda	Low SDI
Senegal	Low SDI
Sierra Leone	Low SDI
Solomon Islands	Low SDI
Somalia	Low SDI
South Sudan	Low SDI
Togo	Low SDI
Uganda	Low SDI
United Republic of Tanzania	Low SDI
Yemen	Low SDI
Angola	Low-middle SDI
Bangladesh	Low-middle SDI
Belize	Low-middle SDI
Bolivia (Plurinational State of)	Low-middle SDI
Cabo Verde	Low-middle SDI
Cambodia	Low-middle SDI
Cameroon	Low-middle SDI
Congo	Low-middle SDI

Democratic People's Republic of Korea	Low-middle SDI
Djibouti	Low-middle SDI
Dominican Republic	Low-middle SDI
El Salvador	Low-middle SDI
Eswatini	Low-middle SDI
Ghana	Low-middle SDI
Guatemala	Low-middle SDI
Honduras	Low-middle SDI
India	Low-middle SDI
Kenya	Low-middle SDI
Kyrgyzstan	Low-middle SDI
Lao People's Democratic Republic	Low-middle SDI
Lesotho	Low-middle SDI
Maldives	Low-middle SDI
Marshall Islands	Low-middle SDI
Mauritania	Low-middle SDI
Micronesia (Federated States of)	Low-middle SDI
Morocco	Low-middle SDI
Myanmar	Low-middle SDI
Nicaragua	Low-middle SDI
Nigeria	Low-middle SDI
Pakistan	Low-middle SDI
Philippines	Low-middle SDI
Samoa	Low-middle SDI
Sao Tome and Principe	Low-middle SDI
Sudan	Low-middle SDI
Tajikistan	Low-middle SDI
Timor-Leste	Low-middle SDI
Tuvalu	Low-middle SDI
Vanuatu	Low-middle SDI
Zambia	Low-middle SDI
Zimbabwe	Low-middle SDI
Albania	Middle SDI
Algeria	Middle SDI
Botswana	Middle SDI
Brazil	Middle SDI
Colombia	Middle SDI
Costa Rica	Middle SDI
Cuba	Middle SDI
Ecuador	Middle SDI
Egypt	Middle SDI
Equatorial Guinea	Middle SDI
Fiji	Middle SDI
Gabon	Middle SDI
Grenada	Middle SDI
Guyana	Middle SDI
Indonesia	Middle SDI
Iraq	Middle SDI

Jamaica	Middle SDI
Mexico	Middle SDI
Mongolia	Middle SDI
Namibia	Middle SDI
Nauru	Middle SDI
Palestine	Middle SDI
Panama	Middle SDI
Paraguay	Middle SDI
Peru	Middle SDI
Saint Lucia	Middle SDI
Saint Vincent and the Grenadines	Middle SDI
South Africa	Middle SDI
Sri Lanka	Middle SDI
Suriname	Middle SDI
Syrian Arab Republic	Middle SDI
Thailand	Middle SDI
Tokelau	Middle SDI
Tonga	Middle SDI
Tunisia	Middle SDI
Uzbekistan	Middle SDI
Venezuela (Bolivarian Republic of)	Middle SDI
Viet Nam	Middle SDI
American Samoa	High-middle SDI
Antigua and Barbuda	High-middle SDI
Argentina	High-middle SDI
Armenia	High-middle SDI
Azerbaijan	High-middle SDI
Bahamas	High-middle SDI
Bahrain	High-middle SDI
Barbados	High-middle SDI
Belarus	High-middle SDI
Bosnia and Herzegovina	High-middle SDI
Bulgaria	High-middle SDI
Chile	High-middle SDI
China	High-middle SDI
Cook Islands	High-middle SDI
Croatia	High-middle SDI
Dominica	High-middle SDI
Georgia	High-middle SDI
Greece	High-middle SDI
Greenland	High-middle SDI
Hungary	High-middle SDI
Iran (Islamic Republic of)	High-middle SDI
Italy	High-middle SDI
Jordan	High-middle SDI
Kazakhstan	High-middle SDI
Lebanon	High-middle SDI
Libya	High-middle SDI

Malaysia	High-middle SDI
Malta	High-middle SDI
Mauritius	High-middle SDI
Montenegro	High-middle SDI
Niue	High-middle SDI
North Macedonia	High-middle SDI
Northern Mariana Islands	High-middle SDI
Oman	High-middle SDI
Palau	High-middle SDI
Poland	High-middle SDI
Portugal	High-middle SDI
Republic of Moldova	High-middle SDI
Romania	High-middle SDI
Russian Federation	High-middle SDI
Saint Kitts and Nevis	High-middle SDI
Saudi Arabia	High-middle SDI
Serbia	High-middle SDI
Seychelles	High-middle SDI
Spain	High-middle SDI
Trinidad and Tobago	High-middle SDI
Turkey	High-middle SDI
Turkmenistan	High-middle SDI
Ukraine	High-middle SDI
Uruguay	High-middle SDI
Andorra	High SDI
Australia	High SDI
Austria	High SDI
Belgium	High SDI
Bermuda	High SDI
Brunei Darussalam	High SDI
Canada	High SDI
Cyprus	High SDI
Czechia	High SDI
Denmark	High SDI
Estonia	High SDI
Finland	High SDI
France	High SDI
Germany	High SDI
Guam	High SDI
Iceland	High SDI
Ireland	High SDI
Israel	High SDI
Japan	High SDI
Kuwait	High SDI
Latvia	High SDI
Lithuania	High SDI
Luxembourg	High SDI
Monaco	High SDI

Netherlands	High SDI
New Zealand	High SDI
Norway	High SDI
Puerto Rico	High SDI
Qatar	High SDI
Republic of Korea	High SDI
San Marino	High SDI
Singapore	High SDI
Slovakia	High SDI
Slovenia	High SDI
Sweden	High SDI
Switzerland	High SDI
Taiwan (Province of China)	High SDI
United Arab Emirates	High SDI
United Kingdom	High SDI
United States of America	High SDI
United States Virgin Islands	High SDI

Table S3: Injury-related mortality in adolescents aged 10-24 years. Absolute number of deaths in 1990 and 2019 and percentage change in absolute number between 1990 and 2019 by mechanism of transport and unintentional injuries

		1990 Deaths (Uncertainty Interval)	2019 Deaths (Uncertainty Interval)	Percentage change 1990 to 2019 (Uncertainty Interval)
High SDI	**Transport injuries**	41,019 (40,382 - 42,031)	16,611 (15,633 - 17,562)	-59.5% (-61.7 - -57.4)
	Road injuries	39,397 (38,777 - 40,378)	15,656 (14,699 - 16,586)	-60.3% (-62.5 - -58.2)
	Other transport injuries	1,622 (1,563 - 1,693)	956 (903 - 1,012)	-41.1% (-44.7 - -36.8)
	**Unintentional injuries**	13,894 (12,943 - 14,575)	5,351 (5,050 - 5,711)	-61.5% (-64.0 - -58.0)
	Adverse effects of medical treatment	265 (223 - 311)	172 (151 - 191)	-35.0% (-45.5 - -23.3)
	Animal contact	38 (35 - 41)	28 (26 - 30)	-25.1% (-31.6 - -17.1)
	Drowning	3,960 (3,632 - 4,137)	1,381 (1,304 - 1,472)	-65.1% (-67.5 - -61.9)
	Environmental heat and cold exposure	172 (162 - 203)	113 (108 - 119)	-34.4% (-41.8 - -29.7)
	Exposure to forces of nature	158 (144 - 174)	29 (26 - 32)	-81.7% (-81.7 - -81.7)
	Exposure to mechanical forces	2,414 (2,302 - 2,604)	723 (648 - 814)	-70.0% (-74.0 - -65.9)
	Falls	2,271 (2,067 - 2,420)	1,228 (1,110 - 1,376)	-45.9% (-52.1 - -38.9)
	Fire, heat, and hot substances	1,178 (1,099 - 1,246)	411 (365 - 467)	-65.1% (-68.2 - -60.3)
	Foreign body	788 (755 - 812)	395 (378 - 415)	-49.9% (-52.5 - -46.0)
	Other unintentional injuries	1,760 (1,412 - 2,222)	540 (406 - 703)	-69.4% (-79.9 - -56.5)
	Poisonings	889 (794 - 951)	332 (310 - 354)	-62.6% (-66.1 - -58.0)
High-middle SDI	**Transport injuries**	52,976 (50,168 - 58,948)	26,776 (24,606 - 28,689)	-49.5% (-55.6 - -44.7)
	Road injuries	49,823 (47,178 - 55,558)	24,939 (22,921 - 26,754)	-49.9% (-56.0 - -45.3)
	Other transport injuries	3,154 (2,767 - 3,537)	1,837 (1,576 - 2,059)	-41.7% (-50.5 - -33.3)
	**Unintentional injuries**	42,485 (40,071 - 45,321)	17,295 (15,937 - 18,543)	-59.3% (-63.4 - -55.2)
	Adverse effects of medical treatment	729 (563 - 849)	321 (283 - 358)	-56.0% (-64.0 - -46.1)
	Animal contact	496 (389 - 579)	220 (181 - 284)	-55.5% (-65.8 - -37.1)
	Drowning	18,295 (16,822 - 19,950)	6,125 (5,589 - 6,691)	-66.5% (-70.3 - -62.6)
	Environmental heat and cold exposure	1,626 (1,459 - 1,736)	347 (311 - 383)	-78.7% (-80.9 - -76.1)
	Exposure to forces of nature	236 (214 - 259)	36 (33 - 40)	-84.8% (-84.8 - -84.8)
	Exposure to mechanical forces	4,197 (3,770 - 5,130)	1,850 (1,528 - 2,080)	-55.9% (-66.2 - -46.8)
	Falls	5,835 (5,338 - 6,803)	3,200 (2,827 - 3,537)	-45.2% (-54.6 - -37.1)
	Fire, heat, and hot substances	2,351 (1,990 - 2,758)	1,057 (790 - 1,351)	-55.0% (-63.4 - -40.6)
	Foreign body	1,471 (1,419 - 1,538)	949 (880 - 1,015)	-35.5% (-40.5 - -30.6)
	Other unintentional injuries	4,545 (4,224 - 5,210)	1,880 (1,666 - 2,075)	-58.6% (-65.9 - -52.7)
	Poisonings	2,704 (2,483 - 3,016)	1,310 (1,141 - 1,437)	-51.6% (-61.3 - -45.0)
Middle SDI	**Transport injuries**	102,886 (96,121 - 117,485)	70,690 (64,019 - 77,832)	-31.3% (-40.1 - -23.6)
	Road injuries	96,915 (90,591 - 110,860)	66,840 (60,183 - 73,725)	-31.0% (-39.8 - -23.2)
	Other transport injuries	5,972 (5,033 - 6,943)	3,850 (3,170 - 4,484)	-35.5% (-45.4 - -22.4)
	**Unintentional injuries**	99,910 (91,337 - 107,126)	45,456 (39,152 - 50,411)	-54.5% (-59.4 - -49.3)
	Adverse effects of medical treatment	2,160 (1,484 - 2,592)	986 (816 - 1,138)	-54.4% (-63.1 - -39.4)
	Animal contact	2,593 (1,942 - 3,005)	1,578 (1,282 - 1,982)	-39.2% (-51.0 - -12.2)
	Drowning	40,351 (34,526 - 44,801)	15,811 (13,955 - 17,426)	-60.8% (-65.8 - -55.3)
	Environmental heat and cold exposure	917 (458 - 1,173)	315 (168 - 382)	-65.7% (-71.2 - -58.7)
	Exposure to forces of nature	11,548 (10,485 - 12,706)	249 (226 - 274)	-97.8% (-97.8 - -97.8)
	Exposure to mechanical forces	9,349 (8,030 - 10,916)	5,517 (4,036 - 6,591)	-41.0% (-57.0 - -25.6)
	Falls	10,507 (9,214 - 12,276)	7,440 (6,104 - 8,433)	-29.2% (-42.3 - -16.6)
	Fire, heat, and hot substances	5,844 (3,963 - 7,822)	3,524 (2,138 - 5,225)	-39.7% (-55.3 - -8.0)
	Foreign body	2,472 (2,319 - 2,659)	1,869 (1,676 - 2,101)	-24.4% (-33.2 - -15.8)
	Other unintentional injuries	9,693 (8,137 - 11,472)	5,736 (4,260 - 6,629)	-40.8% (-54.2 - -28.3)
	Poisonings	4,477 (3,938 - 5,133)	2,432 (1,811 - 2,739)	-45.7% (-61.8 - -35.4)
Low - Middle SDI	**Transport injuries**	51,012 (45,971 - 57,281)	57,328 (49,856 - 64,825)	12.4% (-2.1 - 28.6)
	Road injuries	46,259 (41,758 - 52,312)	52,679 (45,611 - 59,504)	13.9% (-1.0 - 30.0)
	Other transport injuries	4,754 (3,959 - 5,570)	4,649 (3,645 - 5,433)	-2.2% (-19.2 - 18.3)
	**Unintentional injuries**	66,888 (57,626 - 74,954)	54,548 (46,828 - 61,976)	-18.4% (-27.2 - -6.6)
	Adverse effects of medical treatment	2,502 (1,717 - 3,112)	2,032 (1,568 - 2,348)	-18.8% (-34.2 - 2.0)
	Animal contact	9,605 (5,875 - 11,697)	7,820 (4,677 - 10,036)	-18.6% (-35.4 - 24.0)
	Drowning	23,231 (20,016 - 27,736)	15,450 (13,360 - 18,270)	-33.5% (-43.1 - -21.8)
	Environmental heat and cold exposure	622 (269 - 823)	501 (214 - 672)	-19.4% (-33.6 - 1.4)
	Exposure to forces of nature	738 (670 - 812)	492 (447 - 542)	-33.3% (-33.3 - -33.3)
	Exposure to mechanical forces	5,197 (4,167 - 6,251)	4,793 (3,908 - 5,727)	-7.8% (-22.4 - 12.4)
	Falls	7,406 (6,285 - 9,103)	8,076 (6,842 - 9,386)	9.0% (-10.5 - 34.9)
	Fire, heat, and hot substances	5,314 (2,833 - 7,608)	4,873 (3,300 - 6,567)	-8.3% (-36.5 - 61.2)
	Foreign body	1,289 (1,168 - 1,423)	1,676 (1,468 - 1,918)	30.1% (9.5 - 49.5)
	Other unintentional injuries	8,079 (5,011 - 10,225)	6,332 (4,365 - 7,572)	-21.6% (-41.1 - -2.0)
	Poisonings	2,906 (2,348 - 3,584)	2,502 (2,075 - 2,915)	-13.9% (-33.0 - 3.7)
Low SDI	**Transport injuries**	23,701 (19,658 - 27,429)	42,774 (35,369 - 50,565)	80.5% (53.4 - 113.3)
	Road injuries	21,950 (18,206 - 25,397)	39,853 (32,786 - 47,034)	81.6% (53.1 - 114.6)
	Other transport injuries	1,751 (1,405 - 2,363)	2,921 (2,331 - 4,023)	66.8% (33.9 - 103.9)

**Unintentional injuries**	22,928 (19,032 - 26,561)	31,961 (26,987 - 37,994)	39.4% (20.0 - 65.3)
Adverse effects of medical treatment	1,323 (857 - 2,109)	1,825 (1,286 - 2,870)	37.9% (16.1 - 76.2)
Animal contact	3,408 (1,987 - 4,537)	3,903 (2,544 - 5,349)	14.5% (-18.1 - 82.2)
Drowning	5,557 (4,489 - 6,702)	6,586 (5,528 - 8,044)	18.5% (-1.4 - 48.6)
Environmental heat and cold exposure	671 (362 - 861)	846 (439 - 1,210)	26.0% (-2.9 - 65.7)
Exposure to forces of nature	155 (141 - 170)	758 (688 - 834)	389.5% (389.5 - 389.5)
Exposure to mechanical forces	2,317 (1,868 - 2,925)	3,820 (2,932 - 5,239)	64.9% (33.7 - 134.1)
Falls	2,269 (1,733 - 2,783)	3,818 (2,996 - 4,760)	68.3% (23.1 - 133.8)
Fire, heat, and hot substances	1,781 (1,076 - 2,481)	2,652 (1,875 - 3,553)	48.9% (2.8 - 151.6)
Foreign body	907 (772 - 1,046)	1,843 (1,553 - 2,188)	103.2% (64.9 - 145.1)
Other unintentional injuries	2,965 (2,136 - 4,072)	3,737 (3,097 - 4,495)	26.0% (-5.4 - 69.8)
Poisonings	1,574 (1,232 - 1,951)	2,174 (1,790 - 2,717)	38.1% (13.2 - 81.9)

Note: Uncertainty intervals percentage of change 1990-2019 for exposure to forces of nature are the same as the percentage value.

Table S4: Injury-related Disability Adjusted Life Years (DALYs) in adolescents aged 10-24 years. Absolute number of DALYs in 1990 and 2019 and percentage change in absolute number between 1990 and 2019 by mechanism of transport and unintentional injuries

		1990 DALYs (thousands) (Uncertainty Interval)	2019 DALYs (thousands) (Uncertainty Interval)	Percentage change1990 to 2019 (Uncertainty Interval)
High SDI	**Transport injuries**	2,982.5 (2,914.7 - 3,065.0)	1,226.4 (1,156.8 - 1,292.3)	-58.9% (-61.0 - -56.9)
	Road injuries	2,851.9 (2,789.3 - 2,928.4)	1,146.1 (1,075.3 - 1,209.2)	-59.8% (-62.0 - -57.8)
	Other transport injuries	130.7 (123.5 - 139.4)	80.3 (74.3 - 87.3)	-38.5% (-41.9 - -34.8)
	**Unintentional injuries**	1,663.1 (1,428.9 - 1,985.7)	973.0 (764.6 - 1,239.6)	-41.5% (-46.5 - -36.8)
	Adverse effects of medical treatment	22.3 (19.0 - 26.1)	16.0 (13.7 - 19.0)	-28.3% (-38.4 - -18.3)
	Animal contact	14.9 (10.2 - 20.6)	13.2 (8.9 - 18.4)	-11.6% (-14.1 - -9.7)
	Drowning	282.7 (260.0 - 295.6)	98.8 (93.4 - 105.0)	-65.0% (-67.4 - -61.9)
	Environmental heat and cold exposure	22.0 (18.2 - 27.2)	16.1 (13.1 - 20.0)	-26.8% (-32.1 - -23.3)
	Exposure to forces of nature	11.8 (10.7 - 13.0)	2.6 (2.3 - 3.3)	-77.5% (-79.1 - -74.6)
	Exposure to mechanical forces	331.9 (268.4 - 423.7)	177.3 (129.5 - 250.8)	-46.6% (-52.6 - -40.5)
	Falls	451.9 (354.0 - 582.6)	374.1 (278.4 - 502.4)	-17.2% (-23.0 - -13.0)
	Fire, heat, and hot substances	129.6 (110.5 - 155.8)	61.9 (47.1 - 83.1)	-52.3% (-58.1 - -46.4)
	Foreign body	83.1 (73.0 - 98.0)	52.8 (43.6 - 66.6)	-36.5% (-40.8 - -31.5)
	Other unintentional injuries	226.2 (179.7 - 289.5)	117.7 (85.8 - 163.8)	-48.0% (-58.2 - -38.3)
	Poisonings	86.8 (76.9 - 98.8)	42.5 (35.2 - 51.4)	-51.1% (-56.0 - -45.1)
High-middle SDI	**Transport injuries**	3,937.6 (3,712.9 - 4,351.6)	2,014.8 (1,858.5 - 2,152.1)	-48.8% (-54.6 - -44.4)
	Road injuries	3,694.0 (3,485.0 - 4,093.9)	1,872.6 (1,724.3 - 1,999.7)	-49.3% (-55.1 - -44.9)
	Other transport injuries	243.6 (217.1 - 273.6)	142.1 (123.2 - 158.5)	-41.6% (-49.5 - -34.0)
	**Unintentional injuries**	3,980.6 (3,628.8 - 4,390.9)	1,856.7 (1,628.7 - 2,160.2)	-53.4% (-57.3 - -49.5)
	Adverse effects of medical treatment	54.2 (43.0 - 63.1)	25.0 (22.1 - 28.1)	-54.0% (-61.9 - -44.3)
	Animal contact	56.9 (46.2 - 68.4)	29.1 (23.5 - 36.2)	-48.9% (-56.5 - -37.6)
	Drowning	1,318.8 (1,214.5 - 1,438.1)	437.3 (399.3 - 476.7)	-66.8% (-70.5 - -63.0)
	Environmental heat and cold exposure	150.6 (134.3 - 168.3)	46.2 (38.3 - 57.1)	-69.3% (-72.8 - -65.4)
	Exposure to forces of nature	19.5 (17.5 - 21.9)	6.4 (5.0 - 8.5)	-67.0% (-72.7 - -58.6)
	Exposure to mechanical forces	508.2 (423.2 - 622.9)	261.0 (207.7 - 335.1)	-48.6% (-56.4 - -43.1)
	Falls	804.1 (675.3 - 966.8)	517.6 (409.7 - 647.4)	-35.6% (-42.5 - -30.7)
	Fire, heat, and hot substances	246.2 (206.2 - 291.9)	117.1 (90.9 - 151.6)	-52.4% (-59.1 - -42.4)
	Foreign body	153.8 (134.4 - 182.0)	96.8 (83.7 - 115.3)	-37.0% (-40.6 - -33.9)
	Other unintentional injuries	452.1 (398.1 - 529.1)	212.8 (178.8 - 260.0)	-52.9% (-59.3 - -48.2)
	Poisonings	216.3 (197.0 - 244.4)	107.4 (95.9 - 118.0)	-50.3% (-58.7 - -44.5)
Middle SDI	**Transport injuries**	7,582.8 (7,099.9 - 8,549.4)	5,252.4 (4,767.3 - 5,759.3)	-30.7% (-39.0 - -23.5)
	Road injuries	7,115.7 (6,672.4 - 8,057.5)	4,949.0 (4,478.0 - 5,433.5)	-30.5% (-38.9 - -23.2)
	Other transport injuries	467.1 (400.2 - 539.8)	303.4 (253.4 - 352.3)	-35.0% (-44.0 - -23.3)
	**Unintentional injuries**	8,319.0 (7,597.7 - 9,007.8)	4,223.0 (3,688.5 - 4,738.7)	-49.2% (-53.7 - -44.3)
	Adverse effects of medical treatment	157.1 (109.4 - 187.3)	73.9 (62.3 - 84.9)	-53.0% (-61.6 - -38.0)
	Animal contact	229.7 (181.7 - 264.1)	145.5 (120.7 - 176.5)	-36.7% (-46.8 - -15.8)
	Drowning	2,933.2 (2,515.3 - 3,252.0)	1,138.8 (1,004.8 - 1,255.1)	-61.2% (-66.0 - -55.8)
	Environmental heat and cold exposure	120.2 (83.9 - 153.1)	68.0 (50.3 - 90.0)	-43.5% (-51.8 - -32.4)
	Exposure to forces of nature	872.0 (791.5 - 959.8)	45.7 (36.0 - 59.4)	-94.8% (-95.7 - -93.3)
	Exposure to mechanical forces	817.6 (706.2 - 953.1)	543.5 (423.0 - 654.7)	-33.5% (-48.1 - -21.2)
	Falls	1,220.1 (1,051.2 - 1,412.5)	950.3 (780.0 - 1,148.8)	-22.1% (-31.9 - -13.6)
	Fire, heat, and hot substances	553.3 (404.4 - 707.4)	342.6 (235.6 - 475.8)	-38.1% (-51.5 - -15.1)
	Foreign body	269.2 (232.4 - 324.4)	202.4 (171.0 - 245.1)	-24.8% (-30.6 - -19.0)
	Other unintentional injuries	808.8 (692.4 - 951.3)	522.5 (409.1 - 606.5)	-35.4% (-47.5 - -24.4)
	Poisonings	337.6 (299.3 - 387.8)	189.8 (148.7 - 212.1)	-43.8% (-58.6 - -34.1)
Low-middle SDI	**Transport injuries**	3,905.2 (3,532.5 - 4,346.3)	4,433.8 (3,878.5 - 4,948.9)	13.5% (0.1 - 28.2)
	Road injuries	3,540.2 (3,196.4 - 3,977.3)	4,070.9 (3,559.8 - 4,547.7)	15.0% (1.4 - 29.6)
	Other transport injuries	365.0 (308.3 - 425.7)	362.9 (293.3 - 420.3)	-0.6% (-16.4 - 18.5)
	**Unintentional injuries**	5,614.5 (4,934.5 - 6,267.4)	4,848.7 (4,221.2 - 5,478.9)	-13.6% (-21.9 - -3.0)
	Adverse effects of medical treatment	178.8 (123.6 - 222.5)	146.3 (114.3 - 168.5)	-18.2% (-33.2 - 2.4)
	Animal contact	744.4 (478.4 - 899.5)	606.9 (381.7 - 766.1)	-18.5% (-34.1 - 20.2)

	Drowning	1,686.4 (1,451.1 - 2,013.0)	1,114.1 (963.8 - 1,317.3)	-33.9% (-43.4 - -22.5)
	Environmental heat and cold exposure	87.0 (61.1 - 110.5)	88.0 (63.9 - 112.5)	1.1% (-10.2 - 13.1)
	Exposure to forces of nature	68.4 (59.3 - 79.6)	79.7 (63.0 - 103.6)	16.5% (1.6 - 36.8)
	Exposure to mechanical forces	460.3 (388.6 - 545.4)	463.2 (388.3 - 548.0)	0.6% (-13.3 - 15.8)
	Falls	858.7 (731.5 - 1,016.7)	958.4 (797.6 - 1,139.3)	11.6% (-2.0 - 27.1)
	Fire, heat, and hot substances	477.4 (297.8 - 643.4)	431.1 (317.2 - 556.3)	-9.7% (-33.0 - 36.8)
	Foreign body	154.3 (131.0 - 185.4)	199.0 (164.7 - 243.1)	28.9% (16.1 - 40.5)
	Other unintentional injuries	679.0 (453.2 - 841.0)	569.1 (420.2 - 672.2)	-16.2% (-33.5 - 1.1)
	Poisonings	219.8 (180.0 - 267.9)	193.0 (161.9 - 223.0)	-12.2% (-31.1 - 4.2)
Low SDI	**Transport injuries**	1,823.1 (1,537.5 - 2,085.0)	3,294.6 (2,753.8 - 3,848.2)	80.7% (55.8 - 110.4)
	Road injuries	1,685.6 (1,417.3 - 1,926.9)	3,060.8 (2,551.3 - 3,584.1)	81.6% (55.3 - 111.4)
	Other transport injuries	137.5 (113.4 - 179.1)	233.9 (193.5 - 314.9)	70.1% (40.3 - 103.8)
	**Unintentional injuries**	1,981.7 (1,674.9 - 2,262.5)	2,974.6 (2,544.8 - 3,518.9)	50.1% (31.5 - 72.6)
	Adverse effects of medical treatment	95.1 (62.1 - 150.9)	132.0 (93.3 - 205.2)	38.8% (16.9 - 76.9)
	Animal contact	268.4 (163.8 - 348.0)	317.8 (219.2 - 423.7)	18.4% (-12.9 - 80.2)
	Drowning	405.8 (327.8 - 488.0)	479.4 (402.2 - 585.9)	18.1% (-1.5 - 48.0)
	Environmental heat and cold exposure	64.2 (41.5 - 79.2)	93.0 (62.4 - 124.4)	44.9% (19.6 - 77.8)
	Exposure to forces of nature	14.7 (12.7 - 17.2)	98.0 (80.0 - 122.1)	567.0% (514.4 - 636.3)
	Exposure to mechanical forces	209.3 (172.9 - 258.0)	364.7 (290.5 - 464.7)	74.2% (48.0 - 128.0)
	Falls	269.9 (219.7 - 325.8)	501.6 (402.3 - 616.3)	85.9% (53.3 - 124.7)
	Fire, heat, and hot substances	182.4 (125.7 - 235.4)	272.3 (211.5 - 346.3)	49.3% (14.5 - 114.3)
	Foreign body	103.6 (88.4 - 122.5)	213.7 (175.9 - 256.4)	106.2% (81.1 - 131.5)
	Other unintentional injuries	250.6 (189.3 - 331.0)	338.4 (284.3 - 400.6)	35.1% (6.1 - 72.1)
	Poisonings	117.8 (93.6 - 144.9)	163.8 (137.0 - 203.9)	39.1% (14.5 - 82.1)

Table S5: Level 4 breakdown of road injuries category, deaths absolute numbers and rates per 100,000 population 1990 and 2019, and percentage change between 1990 and 2019 by Socio Demographic Index (SDI)

		Deaths in 1990 (UI)	Deaths in 2019 (UI)	Deaths % change (UI)	Deaths/100,000 in 1990 (UI)	Deaths/100,000 in 2019 (UI)	Deaths per 100,000 % change (UI)
Global	**Road injuries**	254,510.0 (240,521.1 to 280,349.0)	200,112.9 (180,273.8 to 220,460.9)	-21.4% (-29.8 to -13.5)	16.4 (15.5 to 18.1)	10.7 (9.7 to 11.8)	-34.6% (-41.6 to -28.1)
	Pedestrian road injuries	89,417.9 (75,694.2 to 104,826.3)	55,392.6 (46,538.8 to 62,816.8)	-38.1% (-49.0 to -23.9)	5.8 (4.9 to 6.8)	3.0 (2.5 to 3.4)	-48.5% (-57.6 to -36.7)
	Cyclist road injuries	9,359.3 (7,749.5 to 11,966.9)	7,889.8 (6,811.7 to 8,834.1)	-15.7% (-40.4 to 5.0)	0.6 (0.5 to 0.8)	0.4 (0.4 to 0.5)	-29.9% (-50.4 to -12.7)
	Motorcyclist road injuries	53,083.8 (45,430.8 to 64,461.1)	51,354.0 (44,220.7 to 57,749.9)	-3.3% (-20.2 to 15.8)	3.4 (2.9 to 4.2)	2.8 (2.4 to 3.1)	-19.5% (-33.6 to -3.7)
	Motor vehicle road injuries	100,284.8 (90,270.4 to 112,699.6)	83,372.5 (74,249.3 to 94,239.1)	-16.9% (-26.6 to -6.3)	6.5 (5.8 to 7.3)	4.5 (4.0 to 5.1)	-30.8% (-38.9 to -22.0)
	Other road injuries	2,364.2 (2,001.6 to 2,851.9)	2,104.0 (1,807.7 to 2,432.7)	-11.0% (-30.4 to 14.0)	0.2 (0.1 to 0.2)	0.1 (0.1 to 0.1)	-26.0% (-42.1 to -5.2)
Low SDI	**Road injuries**	21,949.8 (18,206.4 to 25,397.0)	39,853.2 (32,786.1 to 47,034.4)	81.6% (53.1 to 114.6)	13.4 (11.1 to 15.5)	10.7 (8.8 to 12.7)	-19.9% (-32.5 to -5.4)
	Pedestrian road injuries	8,480.3 (6,114.8 to 11,097.3)	13,455.2 (10,175.6 to 16,661.4)	58.7% (25.3 to 108.8)	5.2 (3.7 to 6.8)	3.6 (2.7 to 4.5)	-30.0% (-44.7 to -7.9)
	Cyclist road injuries	790.1 (608.9 to 1,162.2)	1,283.1 (979.5 to 1,681.3)	62.4% (2.2 to 114.6)	0.5 (0.4 to 0.7)	0.3 (0.3 to 0.5)	-28.4% (-54.9 to -5.4)
	Motorcyclist road injuries	2,573.4 (2,027.6 to 3,734.1)	5,383.1 (4,116.5 to 7,206.2)	109.2% (55.1 to 172.3)	1.6 (1.2 to 2.3)	1.4 (1.1 to 1.9)	-7.7% (-31.6 to 20.1)
	Motor vehicle road injuries	9,934.4 (7,336.7 to 12,281.7)	19,408.1 (15,371.9 to 23,666.2)	95.4% (61.4 to 151.4)	6.1 (4.5 to 7.5)	5.2 (4.1 to 6.4)	-13.8% (-28.8 to 10.9)
	Other road injuries	171.5 (129.4 to 244.5)	323.7 (236.2 to 439.8)	88.7% (36.5 to 162.8)	0.1 (0.1 to 0.1)	0.1 (0.1 to 0.1)	-16.8% (-39.8 to 15.9)
Low-middle SDI	**Road injuries**	46,258.9 (41,757.9 to 52,312.2)	52,679.0 (45,610.7 to 59,504.3)	13.9% (-1.0 to 30.0)	13.1 (11.8 to 14.8)	10.3 (8.9 to 11.7)	-21.0% (-31.4 to -9.9)
	Pedestrian road injuries	17,326.6 (14,258.7 to 21,049.1)	15,183.5 (12,423.4 to 17,538.1)	-12.4% (-31.3 to 10.7)	4.9 (4.0 to 5.9)	3.0 (2.4 to 3.4)	-39.2% (-52.4 to -23.2)
	Cyclist road injuries	2,019.3 (1,582.5 to 2,866.2)	2,348.3 (1,898.7 to 2,793.3)	16.3% (-25.7 to 51.3)	0.6 (0.4 to 0.8)	0.5 (0.4 to 0.5)	-19.4% (-48.5 to 4.9)
	Motorcyclist road injuries	11,366.1 (9,236.4 to 13,871.6)	15,821.4 (12,568.5 to 18,358.2)	39.2% (8.9 to 71.5)	3.2 (2.6 to 3.9)	3.1 (2.5 to 3.6)	-3.5% (-24.5 to 18.9)
	Motor vehicle road injuries	14,969.1 (12,326.5 to 18,747.1)	18,643.0 (15,886.6 to 22,075.4)	24.5% (4.1 to 52.5)	4.2 (3.5 to 5.3)	3.7 (3.1 to 4.3)	-13.7% (-27.8 to 5.7)
	Other road injuries	577.8 (469.1 to 730.6)	682.8 (533.8 to 835.8)	18.2% (-15.8 to 62.4)	0.2 (0.1 to 0.2)	0.1 (0.1 to 0.2)	-18.1% (-41.6 to 12.6)
Middle SDI	**Road injuries**	96,914.6 (90,591.0 to 110,860.3)	66,839.8 (60,183.4 to 73,725.4)	-31.0% (-39.8 to -23.2)	17.8 (16.6 to 20.4)	12.2 (11.0 to 13.4)	-31.7% (-40.4 to -23.9)
	Pedestrian road injuries	38,921.3 (32,222.2 to 46,752.9)	18,596.9 (15,342.0 to 21,403.2)	-52.2% (-63.4 to -37.2)	7.1 (5.9 to 8.6)	3.4 (2.8 to 3.9)	-52.7% (-63.7 to -37.7)
	Cyclist road injuries	3,319.8 (2,576.1 to 4,398.0)	2,668.7 (2,191.3 to 3,076.2)	-19.6% (-48.9 to 11.0)	0.6 (0.5 to 0.8)	0.5 (0.4 to 0.6)	-20.4% (-49.4 to 10.0)
	Motorcyclist road injuries	23,084.1 (18,633.8 to 29,554.6)	21,295.0 (18,203.7 to 24,107.8)	-7.8% (-28.5 to 17.8)	4.2 (3.4 to 5.4)	3.9 (3.3 to 4.4)	-8.6% (-29.2 to 16.7)
	Motor vehicle road injuries	30,682.2 (26,720.7 to 38,156.6)	23,525.0 (20,673.9 to 28,012.2)	-23.3% (-36.6 to -10.4)	5.6 (4.9 to 7.0)	4.3 (3.8 to 5.1)	-24.0% (-37.2 to -11.3)
	Other road injuries	907.1 (759.7 to 1,106.0)	754.2 (646.8 to 869.5)	-16.9% (-36.8 to 5.3)	0.2 (0.1 to 0.2)	0.1 (0.1 to 0.2)	-17.6% (-37.4 to 4.4)
High-middle SDI	**Road injuries**	49,822.6 (47,177.7 to 55,558.1)	24,938.8 (22,921.4 to 26,754.4)	-49.9% (-56.0 to -45.3)	16.5 (15.6 to 18.4)	9.8 (9.0 to 10.5)	-40.3% (-47.5 to -34.8)
	Pedestrian road injuries	19,034.3 (16,661.3 to 22,592.0)	6,085.7 (5,323.5 to 7,029.5)	-68.0% (-74.2 to -59.2)	6.3 (5.5 to 7.5)	2.4 (2.1 to 2.8)	-61.9% (-69.2 to -51.4)
	Cyclist road injuries	1,824.9 (1,416.4 to 2,381.0)	1,119.9 (955.4 to 1,268.7)	-38.6% (-57.0 to -17.9)	0.6 (0.5 to 0.8)	0.4 (0.4 to 0.5)	-26.8% (-48.8 to -2.1)
	Motorcyclist road injuries	9,446.0 (8,080.6 to 11,067.6)	6,469.0 (5,510.4 to 7,270.3)	-31.5% (-46.7 to -15.1)	3.1 (2.7 to 3.7)	2.5 (2.2 to 2.9)	-18.3% (-36.5 to 1.2)
	Motor vehicle road injuries	18,934.4 (17,679.7 to 20,751.1)	11,025.2 (10,155.3 to 11,897.5)	-41.8% (-48.4 to -36.1)	6.3 (5.8 to 6.9)	4.3 (4.0 to 4.7)	-30.6% (-38.4 to -23.8)
	Other road injuries	582.9 (455.3 to 745.9)	239.0 (201.7 to 291.9)	-59.0% (-69.2 to -42.6)	0.2 (0.2 to 0.2)	0.1 (0.1 to 0.1)	-51.1% (-63.3 to -31.5)
High SDI	**Road injuries**	39,396.6 (38,777.5 to 40,378.4)	15,655.6 (14,698.6 to 16,585.9)	-60.3% (-62.5 to -58.2)	21.5 (21.1 to 22.0)	8.9 (8.4 to 9.4)	-58.5% (-60.9 to -56.3)
	Pedestrian road injuries	5,614.4 (5,303.5 to 6,013.1)	2,040.9 (1,849.7 to 2,262.1)	-63.6% (-68.3 to -58.8)	3.1 (2.9 to 3.3)	1.2 (1.1 to 1.3)	-62.0% (-66.8 to -57.0)
	Cyclist road injuries	1,394.7 (1,325.5 to 1,469.9)	464.6 (435.4 to 494.9)	-66.7% (-69.6 to -64.0)	0.8 (0.7 to 0.8)	0.3 (0.2 to 0.3)	-65.2% (-68.3 to -62.4)
	Motorcyclist road injuries	6,579.7 (6,271.1 to 7,095.3)	2,350.2 (2,163.3 to 2,593.1)	-64.3% (-67.7 to -61.1)	3.6 (3.4 to 3.9)	1.3 (1.2 to 1.5)	-62.7% (-66.2 to -59.4)
	Motor vehicle road injuries	25,684.9 (25,135.1 to 26,323.4)	10,697.3 (10,002.2 to 11,365.9)	-58.4% (-61.0 to -55.8)	14.0 (13.7 to 14.4)	6.1 (5.7 to 6.5)	-56.5% (-59.3 to -53.9)
	Other road injuries	123.0 (111.1 to 138.7)	102.5 (91.4 to 116.7)	-16.6% (-30.6 to -2.0)	0.1 (0.1 to 0.1)	0.1 (0.1 to 0.1)	-12.9% (-27.6 to 2.4)

Table S6: Tier 4 breakdown of road injuries category, DALYs absolute numbers and rates per 100,000 population 1990 and 2019, and percentage change between 1990 and 2019 by SDI

		DALYs (in thousands) in 1990	DALYs (in thousands) in 2019	DALYs % change	DALYs/100,000 in 1990	DALYs/100,000 in 2019	DALYs per 100,000 % change
Global	**Road injuries**	18,899.8 (17,915.5 to 20,746.8)	15,110.2 (13,667.7 to 16,591.9)	-20.1% (-28.3 to -12.9)	1,220.2 (1,156.6 to 1,339.4)	811.6 (734.1 to 891.1)	-33.5% (-40.3 to -27.5)
	Pedestrian road injuries	6,600.0 (5,607.7 to 7,706.5)	4,156.9 (3,573.0 to 4,686.8)	-37.0% (-47.5 to -23.7)	426.1 (362.0 to 497.5)	223.3 (191.9 to 251.7)	-47.6% (-56.3 to -36.5)
	Cyclist road injuries	994.5 (824.8 to 1,195.2)	940.7 (786.1 to 1,105.9)	-5.4% (-25.3 to 9.4)	64.2 (53.2 to 77.2)	50.5 (42.2 to 59.4)	-21.3% (-37.8 to -9.0)
	Motorcyclist road injuries	3,899.7 (3,373.1 to 4,682.9)	3,817.4 (3,320.6 to 4,237.0)	-2.1% (-18.0 to 15.8)	251.8 (217.8 to 302.3)	205.0 (178.3 to 227.6)	-18.6% (-31.8 to -3.7)
	Motor vehicle road injuries	7,217.0 (6,508.4 to 8,085.5)	6,022.9 (5,395.6 to 6,817.4)	-16.5% (-25.9 to -6.3)	465.9 (420.2 to 522.0)	323.5 (289.8 to 366.2)	-30.6% (-38.4 to -22.1)
	Other road injuries	188.5 (161.4 to 222.4)	172.3 (147.4 to 196.2)	-8.6% (-26.8 to 13.1)	12.2 (10.4 to 14.4)	9.3 (7.9 to 10.5)	-24.0% (-39.1 to -5.9)
Low SDI	**Road injuries**	1,685.6 (1,417.3 to 1,926.9)	3,060.8 (2,551.3 to 3,584.1)	81.6% (55.3 to 111.4)	1,028.7 (865.0 to 1,176.0)	823.9 (686.8 to 964.8)	-19.9% (-31.5 to -6.7)
	Pedestrian road injuries	646.3 (481.0 to 831.6)	1,026.6 (798.2 to 1,255.6)	58.8% (27.2 to 104.6)	394.5 (293.6 to 507.6)	276.3 (214.9 to 338.0)	-29.9% (-43.9 to -9.7)
	Cyclist road injuries	103.3 (83.6 to 132.1)	182.8 (146.7 to 224.4)	77.0% (36.3 to 104.7)	63.0 (51.0 to 80.6)	49.2 (39.5 to 60.4)	-21.9% (-39.9 to -9.7)
	Motorcyclist road injuries	199.8 (160.5 to 280.2)	417.5 (327.7 to 544.4)	109.0% (60.6 to 165.1)	121.9 (98.0 to 171.0)	112.4 (88.2 to 146.5)	-7.8% (-29.2 to 16.9)
	Motor vehicle road injuries	722.4 (542.2 to 890.5)	1,407.4 (1,121.2 to 1,708.6)	94.8% (61.7 to 149.0)	440.9 (330.9 to 543.5)	378.8 (301.8 to 459.9)	-14.1% (-28.7 to 9.8)
	Other road injuries	13.8 (10.7 to 18.9)	26.5 (20.2 to 34.8)	92.3% (44.9 to 156.4)	8.4 (6.5 to 11.5)	7.1 (5.4 to 9.4)	-15.2% (-36.1 to 13.1)
Low-middle SDI	**Road injuries**	3,540.2 (3,196.4 to 3,977.3)	4,070.9 (3,559.8 to 4,547.7)	15.0% (1.4 to 29.6)	1,000.4 (903.2 to 1,123.9)	797.6 (697.4 to 891.0)	-20.3% (-29.7 to -10.1)
	Pedestrian road injuries	1,301.8 (1,078.9 to 1,559.5)	1,156.9 (968.2 to 1,331.0)	-11.1% (-28.9 to 10.7)	367.9 (304.9 to 440.7)	226.6 (189.7 to 260.8)	-38.4% (-50.7 to -23.3)
	Cyclist road injuries	261.1 (210.8 to 326.9)	319.8 (258.8 to 385.7)	22.4% (-5.4 to 41.1)	73.8 (59.6 to 92.4)	62.6 (50.7 to 75.6)	-15.1% (-34.4 to -2.2)
	Motorcyclist road injuries	847.3 (697.2 to 1,025.4)	1,183.9 (965.5 to 1,364.4)	39.7% (11.4 to 69.6)	239.4 (197.0 to 289.7)	231.9 (189.1 to 267.3)	-3.1% (-22.8 to 17.6)
	Motor vehicle road injuries	1,084.8 (896.3 to 1,335.0)	1,355.7 (1,167.0 to 1,587.9)	25.0% (4.9 to 51.8)	306.5 (253.3 to 377.2)	265.6 (228.6 to 311.1)	-13.3% (-27.3 to 5.3)
	Other road injuries	45.2 (37.3 to 55.9)	54.6 (43.7 to 66.1)	20.8% (-11.7 to 60.8)	12.8 (10.5 to 15.8)	10.7 (8.6 to 12.9)	-16.3% (-38.8 to 11.5)
Middle SDI	**Road injuries**	7,115.7 (6,672.4 to 8,057.5)	4,949.0 (4,478.0 to 5,433.5)	-30.5% (-38.9 to -23.2)	1,307.0 (1,225.6 to 1,480.0)	900.6 (814.9 to 988.7)	-31.1% (-39.5 to -23.9)
	Pedestrian road injuries	2,838.0 (2,349.3 to 3,393.2)	1,365.8 (1,133.7 to 1,563.6)	-51.9% (-62.5 to -37.6)	521.3 (431.5 to 623.3)	248.5 (206.3 to 284.5)	-52.3% (-62.8 to -38.1)
	Cyclist road injuries	313.8 (250.1 to 391.7)	270.0 (226.6 to 314.9)	-14.0% (-38.3 to 8.8)	57.6 (45.9 to 72.0)	49.1 (41.2 to 57.3)	-14.8% (-38.9 to 7.7)
	Motorcyclist road injuries	1,686.4 (1,380.0 to 2,128.6)	1,563.2 (1,347.1 to 1,748.9)	-7.3% (-27.1 to 16.6)	309.8 (253.5 to 391.0)	284.5 (245.1 to 318.2)	-8.2% (-27.8 to 15.5)
	Motor vehicle road injuries	2,204.9 (1,920.5 to 2,713.9)	1,688.5 (1,485.4 to 2,003.7)	-23.4% (-36.3 to -10.8)	405.0 (352.8 to 498.5)	307.3 (270.3 to 364.6)	-24.1% (-36.9 to -11.6)
	Other road injuries	72.6 (61.7 to 86.0)	61.5 (52.8 to 71.0)	-15.3% (-33.5 to 3.9)	13.3 (11.3 to 15.8)	11.2 (9.6 to 12.9)	-16.1% (-34.2 to 2.9)
High-middle SDI	**Road injuries**	3,694.0 (3,485.0 to 4,093.9)	1,872.6 (1,724.3 to 1,999.7)	-49.3% (-55.1 to -44.9)	1,220.7 (1,151.7 to 1,352.9)	738.0 (679.5 to 788.1)	-39.5% (-46.4 to -34.3)
	Pedestrian road injuries	1,397.0 (1,232.1 to 1,648.8)	453.5 (401.7 to 519.3)	-67.5% (-73.5 to -59.2)	461.7 (407.2 to 544.9)	178.7 (158.3 to 204.6)	-61.3% (-68.4 to -51.3)
	Cyclist road injuries	187.9 (150.1 to 229.8)	119.2 (100.9 to 139.5)	-36.6% (-50.7 to -22.6)	62.1 (49.6 to 76.0)	47.0 (39.8 to 55.0)	-24.3% (-41.2 to -7.8)
	Motorcyclist road injuries	689.8 (592.3 to 802.9)	478.2 (412.3 to 533.2)	-30.7% (-45.0 to -15.3)	228.0 (195.7 to 265.3)	188.5 (162.5 to 210.1)	-17.3% (-34.5 to 1.0)
	Motor vehicle road injuries	1,373.4 (1,282.6 to 1,498.4)	801.2 (739.5 to 867.0)	-41.7% (-48.0 to -36.2)	453.9 (423.8 to 495.2)	315.7 (291.4 to 341.7)	-30.4% (-38.0 to -24.0)
	Other road injuries	45.9 (37.1 to 57.2)	20.5 (17.5 to 24.5)	-55.3% (-65.6 to -39.7)	15.2 (12.3 to 18.9)	8.1 (6.9 to 9.7)	-46.7% (-59.0 to -28.1)
High SDI	**Road injuries**	2,851.9 (2,789.3 to 2,928.4)	1,146.1 (1,075.3 to 1,209.2)	-59.8% (-62.0 to -57.8)	1,555.3 (1,521.2 to 1,597.1)	652.9 (612.6 to 688.9)	-58.0% (-60.3 to -55.9)
	Pedestrian road injuries	413.8 (391.1 to 442.5)	151.9 (138.3 to 167.4)	-63.3% (-67.5 to -58.8)	225.7 (213.3 to 241.3)	86.5 (78.8 to 95.4)	-61.7% (-66.0 to -57.0)
	Cyclist road injuries	127.5 (117.2 to 139.8)	48.5 (42.8 to 54.9)	-62.0% (-64.9 to -59.3)	69.5 (63.9 to 76.2)	27.6 (24.4 to 31.3)	-60.3% (-63.3 to -57.5)
	Motorcyclist road injuries	473.9 (453.3 to 508.9)	172.0 (158.6 to 188.6)	-63.7% (-67.0 to -60.7)	258.5 (247.2 to 277.5)	98.0 (90.4 to 107.4)	-62.1% (-65.5 to -58.9)
	Motor vehicle road injuries	1,825.8 (1,784.1 to 1,872.5)	764.8 (712.7 to 810.2)	-58.1% (-60.7 to -55.7)	995.8 (973.0 to 1,021.2)	435.7 (406.0 to 461.6)	-56.2% (-59.0 to -53.8)
	Other road injuries	10.9 (9.7 to 12.2)	9.0 (7.9 to 10.1)	-17.5% (-28.6 to -6.2)	5.9 (5.3 to 6.7)	5.1 (4.5 to 5.8)	-13.8% (-25.4 to -2.0)

Table S7: Injury-related deaths and DALYs absolute numbers and rates per 100,000 population for males aged 10-24 years by mechanism of injury, transport injuries and unintentional injuries 2019 and percentage change 1990-2019

	Males					
	Deaths (thousands)	Deaths per 100,000	% change 1990-2019 Deaths per 100 000 (95% uncertainty interval [UI])	DALYs (thousands)	DALYs per 100,000	% change 1990-2019 DALYs per 100,000 (95% uncertainty interval [UI])
**Transport injuries**	171.1 (151.7 - 189.2)	17.9 (15.9 - 19.8)	-32.8 (-41.7 to -25.3)	12,725.3 (11,263.5 - 14,020.7)	1,334.6 (1,181.3 - 1,470.5)	-31.9 (-40.3 to -24.6)
Road injuries	159.5 (141.4 - 176.6)	16.7 (14.8 - 18.5)	-33.0 (-41.9 to -25.5)	11,838.5 (10,549.5 - 13,075.4)	1,241.6 (1,106.4 - 1,371.3)	-32.1 (-40.7 to -24.9)
Other transport injuries	11.6 (9.3 - 13.4)	1.2 (1.0 - 1.4)	-29.7 (-40.0 to -17.2)	886.8 (726.1 - 1,011.5)	93.0 (76.2 - 106.1)	-28.9 (-38.4 to -17.0)
**Unintentional injuries**	110.7 (98.2 - 121.7)	11.6 (10.3 - 12.8)	-48.9 (-53.5 to -43.3)	9,982.8 (8,873.5 - 11,176.7)	1,047.0 (930.6 - 1,172.2)	-45.0 (-49.3 to -40.5)
Adverse effects of medical treatment	2.6 (1.9 - 3.4)	0.3 (0.2 - 0.4)	-34.9 (-45.5 to -16.7)	190.9 (142.9 - 248.8)	20.0 (15.0 - 26.1)	-34.0 (-44.1 to -16.4)
Animal contact	7.5 (5.3 - 9.7)	0.8 (0.6 - 1.0)	-37.3 (-50.7 to -6.0)	615.8 (455.7 - 766.8)	64.6 (47.8 - 80.4)	-36.4 (-48.6 to -8.9)
Drowning	35.9 (31.8 - 40.4)	3.8 (3.3 - 4.2)	-57.8 (-63.1 to -50.9)	2,570.7 (2,282.3 - 2,895.6)	269.6 (239.4 - 303.7)	-58.2 (-63.4 to -51.3)
Environmental heat and cold exposure	1.6 (1.0 - 2.1)	0.2 (0.1 - 0.2)	-59.4 (-65.4 to -53.0)	203.3 (153.7 - 258.5)	21.3 (16.1 - 27.1)	-47.9 (-53.3 to -41.8)
Exposure to forces of nature	1.0 (0.9 - 1.1)	0.1 (0.1 - 0.1)	-89.9 (-89.9 to -89.9)	144.5 (117.0 - 181.2)	15.2 (12.3 - 19.0)	-81.5 (-84.2 to -77.4)
Exposure to mechanical forces	13.5 (10.7 - 16.1)	1.4 (1.1 - 1.7)	-43.6 (-54.4 to -31.3)	1,354.3 (1,106.2 - 1,625.9)	142.0 (116.0 - 170.5)	-39.1 (-48.1 to -29.9)
Falls	18.1 (15.3 - 20.9)	1.9 (1.6 - 2.2)	-32.0 (-42.9 to -18.4)	2,139.0 (1,767.9 - 2,558.6)	224.3 (185.4 - 268.3)	-27.7 (-35.7 to -19.1)
Fire, heat, and hot substances	5.1 (3.5 - 6.3)	0.5 (0.4 - 0.7)	-43.0 (-50.2 to -30.0)	520.8 (402.2 - 637.8)	54.6 (42.2 - 66.9)	-40.6 (-46.0 to -31.2)
Foreign body	4.6 (4.1 - 5.2)	0.5 (0.4 - 0.5)	-22.3 (-31.0 to -13.8)	498.3 (418.9 - 602.8)	52.3 (43.9 - 63.2)	-19.9 (-25.8 to -14.2)
Other unintentional injuries	15.2 (10.8 - 17.4)	1.6 (1.1 - 1.8)	-42.1 (-56.5 to -30.6)	1,316.4 (996.4 - 1,517.8)	138.1 (104.5 - 159.2)	-39.2 (-52.3 to -29.6)
Poisonings	5.5 (4.2 - 6.4)	0.6 (0.4 - 0.7)	-43.1 (-54.2 to -33.6)	428.7 (342.5 - 488.4)	45.0 (35.9 - 51.2)	-42.1 (-52.4 to -33.3)

Table S8: Injury-related deaths and DALYs absolute numbers and rates per 100,000 population for females aged 10-24 years by mechanism of injury, transport injuries and unintentional injuries 2019 and percentage change 1990-2019

	Females					
	Deaths (thousands)	Deaths per 100,000	% change 1990-2019 Deaths per 100 000 (95% uncertainty interval [UI])	DALYs (thousands)	DALYs per 100,000	% change 1990-2019 DALYs per 100,000 (95% uncertainty interval [UI])
**Transport injuries**	43.2 (39.1 - 47.3)	4.8 (4.3 - 5.2)	-41.2 (-47.0 to -35.4)	3,508.4 (3,178.3 - 3,844.8)	386.2 (349.9 - 423.3)	-39.1 (-44.5 to -34.0)
Road injuries	40.6 (36.6 - 44.5)	4.5 (4.0 - 4.9)	-41.3 (-47.1 to -35.5)	3,271.7 (2,960.0 - 3,582.8)	360.2 (325.9 - 394.4)	-39.2 (-44.8 to -34.0)
Other transport injuries	2.6 (2.3 - 3.0)	0.3 (0.3 - 0.3)	-39.5 (-47.6 to -27.5)	236.6 (206.5 - 268.7)	26.1 (22.7 - 29.6)	-37.1 (-44.3 to -27.3)
**Unintentional injuries**	44.1 (36.4 - 53.1)	4.8 (4.0 - 5.8)	-45.1 (-53.8 to -32.0)	4,905.2 (4,071.7 - 5,812.7)	540.0 (448.2 - 639.9)	-37.4 (-44.4 to -27.5)
Adverse effects of medical treatment	2.8 (2.1 - 3.4)	0.3 (0.2 - 0.4)	-37.6 (-48.7 to -22.9)	202.5 (152.4 - 247.0)	22.3 (16.8 - 27.2)	-36.9 (-47.8 to -22.5)
Animal contact	6.0 (3.2 - 8.5)	0.7 (0.4 - 0.9)	-18.8 (-43.0 to 55.7)	497.0 (296.6 - 682.8)	54.7 (32.7 - 75.2)	-19.0 (-40.7 to 41.4)
Drowning	9.5 (8.4 - 10.7)	1.0 (0.9 - 1.2)	-62.3 (-67.1 to -56.6)	700.4 (618.4 - 785.0)	77.1 (68.1 - 86.4)	-62.2 (-66.9 to -56.6)
Environmental heat and cold exposure	0.5 (0.2 - 0.7)	0.1 (0.0 - 0.1)	-40.9 (-57.5 to -26.0)	108.2 (77.5 - 142.6)	11.9 (8.5 - 15.7)	-25.8 (-33.1 to -19.6)
Exposure to forces of nature	0.5 (0.5 - 0.6)	0.1 (0.1 - 0.1)	-89.8 (-89.8 to -89.8)	89.2 (70.1 - 118.3)	9.8 (7.7 - 13.0)	-78.1 (-81.9 to -72.3)
Exposure to mechanical forces	3.2 (2.3 - 3.8)	0.4 (0.3 - 0.4)	-27.5 (-45.1 to -9.7)	457.2 (359.2 - 587.5)	50.3 (39.5 - 64.7)	-22.2 (-31.7 to -13.0)
Falls	5.7 (4.4 - 6.7)	0.6 (0.5 - 0.7)	-24.8 (-45.6 to -4.1)	1,164.8 (879.6 - 1,502.8)	128.2 (96.8 - 165.4)	-16.0 (-26.6 to -8.0)
Fire, heat, and hot substances	7.4 (4.5 - 11.3)	0.8 (0.5 - 1.2)	-31.5 (-55.6 to 40.3)	705.6 (490.7 - 986.2)	77.7 (54.0 - 108.6)	-31.7 (-51.3 to 10.9)
Foreign body	2.1 (1.9 - 2.4)	0.2 (0.2 - 0.3)	-12.2 (-23.9 to -0.1)	266.9 (222.9 - 320.7)	29.4 (24.5 - 35.3)	-10.8 (-18.1 to -4.0)
Other unintentional injuries	3.1 (2.6 - 3.7)	0.3 (0.3 - 0.4)	-52.6 (-63.1 to -36.0)	445.4 (348.5 - 571.5)	49.0 (38.4 - 62.9)	-40.7 (-50.2 to -29.7)
Poisonings	3.2 (2.8 - 3.6)	0.4 (0.3 - 0.4)	-40.3 (-49.8 to -30.8)	268.1 (234.7 - 302.8)	29.5 (25.8 - 33.3)	-38.9 (-46.9 to -30.5)

Table S9: Adolescent transport and unintentional injury-related mortality in absolute numbers and rates per 100,000 population overall and by age group, by socio-demographic index (SDI), and percentage change from 1990 to 2019.

		10-14 years		15-19 years		20-24 years		10-24 years	
**Transport injury**									
		Number (thousands) (95% UI)	Rate (95% UI)	Number (thousands) (95% UI)	Rate (95% UI)	Number (thousands) (95% UI)	Rate (95% UI)	Number (thousands) (95% UI)	Rate (95% UI)
Global	1990	41.6 (38.1-45.7)	7.8 (7.1-8.5)	102.9 (96.4-113.9)	19.8 (18.6-21.9)	127.3 (120.5-141.3)	25.8 (24.5-28.7)	271.8 (256.8 - 297.9)	17.5 (16.6 – 19.2)
	2010	35.1 (30.9 - 38.4)	5.8 (5.1 - 6.3)	92.8 (83.2 - 99.0)	15.1 (13.5 - 16.1)	140.4 (124.8 - 147.8)	22.7 (20.2 - 23.9)	268.3 (239.5 - 283.9)	14.6 (13.0 - 15.4)
	2019	27.6 (24.0-31.5)	4.3 (3.7-4.9)	75.4 (66.7-83.6)	12.2 (10.8-13.5)	111.4 (100.3-122.0)	18.6 (16.7-20.3)	214.3 (192.8 - 235.3)	11.5 (10.4 - 12.6)
	% change	-33.6 (-41.4--25.2)	-44.5 (-51.0--37.5)	-26.8 (-35.0--18.9)	-38.6 (-45.5--32.0)	-12.5 (-22.2--3.6)	-28.2 (-36.1--20.9)	-21.1 (-29.5 - -13.3)	-34.4 (-41.4 - -27.9)
Low SDI	1990	5.2 (4.1-6.2)	7.9 (6.3-9.5)	9.0 (7.4-10.6)	17.0 (13.9-19.8)	9.4 (7.9-10.9)	21.1 (17.6-24.4)	23.7 (19.7 - 27.4)	14.5 (12.0 - 16.7)
	2010	7.8 (6.4 - 9.3)	6.8 (5.6 - 8.2)	14.5 (11.8 - 16.9)	15.3 (12.4 - 17.8)	17.1 (13.9 - 19.5)	21.6 (17.5 - 24.6)	39.4 (32.5 - 45.4)	13.7 (11.3 - 15.7)
	2019	8.0 (6.4-9.9)	5.5 (4.5-6.9)	15.7 (12.6-18.9)	12.7 (10.2-15.3)	19.1 (15.6-22.5)	18.4 (15.0-21.8)	42.8 (35.4 - 50.6)	11.5 (9.5 - 13.6)
	% change	52.4 (23.7-91.4)	-30.4 (-43.5--12.5)	73.9 (44.8-110.0)	-25.2 (-37.7--9.7)	102.3 (70.2-140.3)	-12.5 (-26.4-3.9)	80.5 (53.4 - 113.3)	-20.4 (-32.3 - -5.9)
Low-middle SDI	1990	8.6 (7.4-10.0)	6.5 (5.6-7.6)	19.0 (16.9-21.7)	16.2 (14.4-18.5)	23.3 (21.1-26.5)	22.5 (20.3-25.5)	51.0 (46.0 - 57.3)	14.4 (13.0 - 16.2)
	2010	10.0 (8.3 - 11.4)	5.8 (4.8 - 6.6)	22.1 (19.1 - 24.3)	13.8 (11.9 - 15.2)	32.5 (27.5 - 35.2)	22.5 (19.0 - 24.4)	64.6 (55.4 - 70.0)	13.6 (11.6 - 14.7)
	2019	7.7 (6.4-9.2)	4.4 (3.6-5.2)	19.6 (16.8-22.4)	11.4 (9.8-13.0)	30.0 (25.8-34.1)	18.6 (15.9-21.0)	57.3 (49.9 - 64.8)	11.2 (9.8 - 12.7)
	% change	-10.5 (-23.9-4.5)	-32.8 (-42.8--21.5)	2.8 (-11.0-18.2)	-29.9 (-39.3--19.4)	28.7 (10.6-49.3)	-17.4 (-29.0--4.1)	12.4 (-2.1 - 28.6)	-22.1 (-32.1 - -10.8)
Middle SDI	1990	17.6 (16.2-19.4)	9.6 (8.8-10.6)	38.2 (35.2-43.9)	20.6 (18.9-23.7)	47.1 (43.7-54.9)	26.7 (24.8-31.1)	102.9 (96.1 - 117.5)	18.9 (17.7 - 21.6)
	2010	12.3 (11.2 - 13.2)	6.7 (6.1 - 7.2)	35.0 (31.7 - 37.2)	17.5 (15.9 - 18.6)	52.6 (47.8 - 56.0)	25.3 (23.0 - 27.0)	99.9 (90.6 - 105.7)	16.9 (15.3 - 17.9)
	2019	8.4 (7.4-9.4)	4.5 (4.0-5.1)	24.9 (22.3-27.6)	13.6 (12.2-15.1)	37.4 (33.7-41.1)	20.5 (18.5-22.5)	70.7 (64.0 - 77.8)	12.9 (11.6 - 14.2)
	% change	-52.5 (-57.9--46.3)	-52.9 (-58.2--46.8)	-34.7 (-43.2--26.8)	-33.8 (-42.4--25.8)	-20.6 (-32.1--11.0)	-23.3 (-34.4--14.0)	-31.3 (-40.1 - -23.6)	-31.9 (-40.6 - -24.3)
High-middle SDI	1990	6.6 (6.2-7.2)	6.7 (6.3-7.3)	19.5 (18.3-21.8)	19.2 (18.0-21.5)	26.9 (25.3-30.1)	26.2 (24.6-29.3)	53.0 (50.2 - 58.9)	17.5 (16.6 - 19.5)
	2010	3.5 (3.2 - 3.7)	4.3 (3.9 - 4.5)	13.3 (12.1 - 13.8)	13.5 (12.3 - 14.1)	26.4 (24.1 - 27.6)	21.7 (19.9 - 22.7)	43.2 (39.5 - 44.9)	14.3 (13.1 - 14.9)
	2019	2.4 (2.2-2.6)	3.0 (2.7-3.2)	8.9 (8.2-9.6)	10.8 (9.9-11.6)	15.4 (14.1-16.6)	17.1 (15.6-18.4)	26.8 (24.6 - 28.7)	10.6 (9.7 - 11.3)
	% change	-63.3 (-67.2--59.4)	-55.4 (-60.1--50.6)	-54.2 (-60.0--49.3)	-43.7 (-50.9--37.6)	-42.7 (-50.0--36.9)	-34.7 (-43.1--28.2)	-49.5 (-55.6 - -44.7)	-39.7 (-47.0 - -34.1)
High SDI	1990	3.6 (3.5-3.7)	6.2 (6.0-6.5)	17.1 (16.8-17.6)	27.8 (27.3-28.7)	20.3 (20.0-20.8)	31.4 (30.9-32.1)	41.0 (40.4 - 42.0)	22.4 (22.0 - 22.9)
	2010	1.4 (1.4 - 1.5)	2.5 (2.4 - 2.6)	7.8 (7.6 - 8.0)	12.7 (12.4 - 13.1)	11.8 (11.4 - 12.3)	18.1 (17.5 - 18.8)	21.0 (20.5 - 21.7)	11.5 (11.2 - 11.8)
	2019	1.1 (1.1-1.2)	2.0 (1.9-2.1)	6.2 (5.8-6.4)	10.7 (10.0-11.2)	9.3 (8.7-10.0)	15.1 (14.1-16.2)	16.6 (15.6 - 17.6)	9.5 (8.9 - 10.0)
	% change	-68.0 (-69.7--66.2)	-67.5 (-69.2--65.7)	-64.0 (-66.0--62.4)	-61.5 (-63.7--59.9)	-54.2 (-57.2--51.0)	-52.0 (-55.1--48.7)	-59.5 (-61.7 - -57.4)	-57.7 (-60.0 - -55.5)
**Unintentional injury**									
		10-14 years		15-19 years		20-24 years		10-24 years	
		Number (thousands)	Rate	Number (thousands)	Rate	Number (thousands)	Rate	Number (thousands)	Rate
Global	1990	76.6 (68.9-83.7)	14.3 (12.8-15.6)	82.0 (74.6-88.4)	15.8 (14.4-17.0)	87.6 (80.4-94.0)	17.8 (16.3-19.1)	246.2 (225.4 - 263.7)	15.9 (14.6 - 17.0)
	2010	80.8 (73.3 - 87.2)	13.3 (12.0 - 14.3)	85.6 (78.1 - 91.2)	13.9 (12.7 - 14.8)	102.8 (94.1 - 108.7)	16.6 (15.2 - 17.6)	269.2 (247.0 - 286.3)	14.6 (13.4 - 15.5)
	2019	42.1 (36.2-48.1)	6.6 (5.6-7.5)	49.0 (43.0-54.8)	7.9 (6.9-8.9)	63.6 (56.3-70.4)	10.6 (9.4-11.7)	154.7 (136.6 - 172.7)	8.3 (7.3 - 9.3)
	% change	-45.1 (-50.7--37.6)	-54.1 (-58.8--47.8)	-40.2 (-45.6--33.3)	-49.9 (-54.4--44.1)	-27.4 (-33.7--19.9)	-40.4 (-45.6--34.3)	-37.2 (-42.7 - -30.2)	-47.7 (-52.3 - -41.9)
Low SDI	1990	8.2 (6.6-9.7)	12.4 (10.0-14.8)	7.3 (5.9-8.5)	13.6 (11.2-16.0)	7.5 (6.2-8.7)	16.8 (13.8-19.4)	22.9 (19.0 - 26.6)	14.0 (11.6 - 16.2)
	2010	33.7 (30.8 - 36.7)	29.6 (27.0 - 32.2)	32.5 (30.0 - 35.4)	34.2 (31.5 - 37.2)	33.0 (30.4 - 35.9)	41.7 (38.4 - 45.3)	99.3 (91.4 - 107.7)	34.4 (31.7 - 37.4)
	2019	10.7 (8.7-13.1)	7.4 (6.0-9.1)	9.9 (8.3-11.9)	8.0 (6.7-9.6)	11.3 (9.6-13.3)	10.9 (9.2-12.9)	32.0 (27.0 - 38.0)	8.6 (7.3 - 10.2)
	% change	31.1 (8.0-66.0)	-40.1 (-50.6--24.2)	36.9 (17.3-62.8)	-41.1 (-49.6--30.0)	50.8 (29.7-77.8)	-34.8 (-43.9--23.2)	39.4 (20.0 - 65.3)	-38.5 (-47.1 - -27.1)
Low-middle SDI	1990	22.0 (18.4-25.7)	16.6 (13.9-19.3)	22.1 (19.1-24.8)	18.9 (16.3-21.2)	22.7 (19.9-25.3)	21.8 (19.2-24.3)	66.9 (57.6 - 75.0)	18.9 (16.3 - 21.2)
	2010	21.7 (18.3 - 24.7)	12.6 (10.6 - 14.4)	20.8 (17.9 - 23.1)	13.0 (11.2 - 14.4)	24.0 (21.0 - 26.0)	16.6 (14.6 - 18.0)	66.5 (57.8 - 73.1)	14.0 (12.1 - 15.3)
	2019	15.8 (13.0-18.6)	8.9 (7.4-10.5)	17.2 (14.7-19.7)	10.0 (8.6-11.4)	21.6 (18.7-24.4)	13.3 (11.6-15.1)	54.5 (46.8 - 62.0)	10.7 (9.2 - 12.1)
	% change	-28.4 (-37.9--15.9)	-46.2 (-53.4--36.8)	-22.5 (-31.5--10.4)	-47.1 (-53.3--38.9)	-4.9 (-15.8-8.9)	-38.9 (-45.9--30.1)	-18.4 (-27.2 - -6.6)	-43.5 (-49.5 - -35.2)
Middle SDI	1990	32.5 (29.8-34.8)	17.8 (16.3-19.1)	33.3 (29.9-36.1)	17.9 (16.1-19.5)	34.2 (31.2-37.0)	19.4 (17.7-21.0)	99.9 (91.3 - 107.1)	18.4 (16.8 - 19.7)
	2010	17.9 (16.0 - 19.2)	9.7 (8.7 - 10.4)	21.0 (18.2 - 22.5)	10.5 (9.1 - 11.3)	26.8 (23.3 - 28.9)	12.9 (11.2 - 13.9)	65.7 (57.4 - 70.1)	11.1 (9.7 - 11.8)

	2019	11.2 (9.9-12.4)	6.1 (5.4-6.7)	14.8 (12.3-16.6)	8.1 (6.7-9.1)	19.4 (16.5-21.7)	10.7 (9.0-11.9)	45.5 (39.2 - 50.4)	8.3 (7.1 - 9.2)
	% change	-65.5 (-68.6--61.6)	-65.8 (-68.9--61.9)	-55.5 (-60.7--49.6)	-54.9 (-60.1--48.9)	-43.1 (-49.9--36.2)	-45.0 (-51.5--38.3)	-54.5 (-59.4 - -49.3)	-54.9 (-59.8 - -49.8)
High-middle SDI	1990	11.2 (10.6-12.0)	11.4 (10.8-12.2)	14.5 (13.6-15.7)	14.3 (13.3-15.4)	16.8 (15.7-18.0)	16.3 (15.2-17.5)	42.5 (40.1 - 45.3)	14.0 (13.2 - 15.0)
	2010	5.5 (5.2 - 5.8)	6.7 (6.3 - 7.1)	8.4 (7.9 - 8.8)	8.6 (8.0 - 9.0)	14.4 (13.4 - 15.1)	11.9 (11.0 - 12.5)	28.3 (26.5 - 29.6)	9.4 (8.8 - 9.8)
	2019	3.5 (3.3-3.8)	4.4 (4.1-4.7)	5.5 (5.0-5.9)	6.6 (6.1-7.2)	8.3 (7.5-9.0)	9.2 (8.3-10.0)	17.3 (15.9 - 18.5)	6.8 (6.3 - 7.3)
	% change	-68.5 (-71.4--65.4)	-61.7 (-65.2--58.0)	-62.4 (-66.6--58.1)	-53.7 (-58.9--48.5)	-50.5 (-56.3--44.9)	-43.7 (-50.3--37.4)	-59.3 (-63.4 - -55.2)	-51.5 (-56.4 - -46.6)
High SDI	1990	2.7 (2.6-2.9)	4.8 (4.5-5.0)	4.8 (4.5-5.1)	7.9 (7.2-8.3)	6.3 (5.9-6.7)	9.8 (9.2-10.3)	13.9 (12.9 - 14.6)	7.6 (7.1 - 7.9)
	2010	1.1 (1.0 - 1.1)	1.9 (1.8 - 1.9)	2.1 (2.0 - 2.2)	3.4 (3.3 - 3.6)	3.7 (3.5 - 3.9)	5.6 (5.3 - 6.0)	6.9 (6.6 - 7.2)	3.7 (3.6 - 3.9)
	2019	0.8 (0.8-0.9)	1.5 (1.4-1.6)	1.6 (1.5-1.7)	2.8 (2.7-3.0)	2.9 (2.7-3.2)	4.7 (4.3-5.1)	5.4 (5.0 - 5.7)	3.0 (2.9 - 3.3)
	% change	-69.2 (-71.3--66.9)	-68.7 (-70.8--66.4)	-66.1 (-68.4--63.4)	-63.8 (-66.2--60.9)	-54.7 (-59.0--49.5)	-52.5 (-57.0--47.0)	-61.5 (-64.0 - -58.0)	-59.8 (-62.4 - -56.1)

Table S10: Injury-related mortality in absolute numbers and rates per 100,000 population for **males** and by age group, by socio-demographic index, transport injury and unintentional injury and trends over time 1990-2019

Transport injury

		10-14 years		15-19 years		20-24 years		10-24 years	
		Number (thousands)	Rate	Number (thousands)	Rate	Number (thousands)	Rate	Number (thousands)	Rate
Global	1990	28.1 (25.5 - 31.3)	10.2 (9.3 - 11.4)	78.8 (73.4 - 89.6)	29.9 (27.8 - 33.9)	103.2 (97.0 - 116.8)	41.5 (39.0 - 47.0)	210.1 (197.3 - 236.6)	26.7 (25.1 - 30.1)
	2010	24.3 (20.1 - 26.9)	7.7 (6.4 - 8.5)	72.7 (63.3 - 78.1)	23.1 (20.1 - 24.8)	115.8 (100.4 - 122.3)	37.1 (32.2 - 39.2)	212.7 (184.2 - 225.9)	22.6 (19.6 - 24.0)
	2019	18.7 (15.4 - 21.8)	5.6 (4.7 - 6.6)	59.3 (51.8 - 66.3)	18.7 (16.3 - 20.9)	93.1 (82.5 - 102.4)	30.6 (27.1 - 33.7)	171.1 (151.7 - 189.2)	17.9 (15.9 - 19.8)
	% change	-33.6 (-42.7 - -23.7)	-44.9 (-52.5 - -36.7)	-24.7 (-35.4 - -15.6)	-37.5 (-46.3 - -29.8)	-9.7 (-21.9 - 0.4)	-26.4 (-36.3 - -18.1)	-18.6 (-29.4 - -9.5)	-32.8 (-41.7 - -25.3)
Low SDI	1990	3.4 (2.6 - 4.2)	10.2 (7.7 - 12.4)	6.8 (5.4 - 8.1)	25.3 (19.9 - 30.0)	7.3 (6.0 - 8.6)	33.1 (27.0 - 38.9)	17.6 (14.0 - 20.4)	21.3 (16.9 - 24.6)
	2010	5.2 (4.0 - 6.2)	8.8 (6.9 - 10.6)	11.3 (8.8 - 13.3)	23.5 (18.3 - 27.6)	14.0 (10.8 - 16.0)	35.8 (27.7 - 40.9)	30.4 (24.0 - 34.8)	20.9 (16.5 - 23.9)
	2019	5.1 (3.9 - 6.4)	7.0 (5.4 - 8.8)	12.2 (9.7 - 14.9)	19.5 (15.5 - 23.8)	15.7 (12.3 - 18.6)	30.5 (23.9 - 36.3)	33.0 (26.3 - 39.2)	17.7 (14.0 - 20.9)
	% change	50.1 (18.0 - 97.2)	-30.9 (-45.7 - -9.3)	78.4 (46.1 - 121.5)	-23.1 (-37.0 - -4.5)	113.9 (78.6 - 159.9)	-7.9 (-23.1 - 11.9)	87.7 (58.5 - 125.2)	-17.0 (-29.9 - -0.4)
Low-middle SDI	1990	5.9 (5.0 - 7.0)	8.7 (7.3 - 10.4)	14.4 (12.6 - 16.9)	24.3 (21.2 - 28.5)	18.8 (16.8 - 22.0)	35.9 (32.2 - 42.1)	39.2 (34.9 - 45.0)	21.8 (19.4 - 25.1)
	2010	7.2 (5.7 - 8.4)	8.2 (6.4 - 9.4)	17.5 (14.4 - 19.4)	21.3 (17.6 - 23.7)	27.3 (22.4 - 29.8)	37.6 (30.8 - 41.0)	52.0 (42.8 - 56.7)	21.4 (17.6 - 23.3)
	2019	5.4 (4.2 - 6.5)	5.9 (4.6 - 7.2)	15.5 (12.9 - 18.1)	17.7 (14.7 - 20.7)	25.3 (21.2 - 29.1)	31.1 (26.1 - 35.8)	46.1 (38.8 - 52.8)	17.8 (15.0 - 20.3)
	% change	-9.3 (-25.4 - 8.8)	-32.1 (-44.1 - -18.5)	7.2 (-10.5 - 27.1)	-27.2 (-39.2 - -13.7)	34.6 (11.8 - 58.1)	-13.5 (-28.1 - 1.6)	17.8 (-1.7 - 36.5)	-18.4 (-32.0 - -5.5)
Middle SDI	1990	12.0 (10.8 - 13.5)	12.8 (11.6 - 14.5)	29.5 (27.0 - 35.1)	31.3 (28.6 - 37.2)	38.0 (34.8 - 45.8)	42.9 (39.2 - 51.7)	79.5 (73.4 - 93.5)	28.8 (26.6 - 33.9)
	2010	8.6 (7.4 - 9.3)	8.9 (7.7 - 9.7)	27.9 (24.5 - 30.0)	27.3 (23.9 - 29.3)	43.4 (38.2 - 46.5)	41.7 (36.7 - 44.6)	79.9 (70.6 - 85.3)	26.4 (23.3 - 28.2)
	2019	5.8 (5.0 - 6.7)	6.1 (5.1 - 7.0)	20.3 (17.9 - 22.6)	21.4 (18.9 - 23.8)	31.7 (28.2 - 35.1)	34.2 (30.4 - 37.9)	57.8 (51.5 - 63.9)	20.4 (18.2 - 22.6)
	% change	-51.2 (-57.5 - -43.6)	-52.6 (-58.8 - -45.3)	-31.3 (-42.7 - -21.5)	-31.7 (-43.0 - -21.9)	-16.6 (-31.1 - -5.1)	-20.2 (-34.1 - -9.3)	-27.3 (-38.8 - -17.7)	-29.2 (-40.4 - -19.9)
High-middle SDI	1990	4.5 (4.2 - 5.1)	9.0 (8.3 - 10.1)	15.2 (14.1 - 17.4)	29.3 (27.2 - 33.7)	22.7 (21.1 - 25.7)	43.6 (40.6 - 49.4)	42.4 (39.7 - 48.4)	27.5 (25.8 - 31.4)
	2010	2.4 (2.1 - 2.5)	5.5 (4.9 - 5.9)	10.2 (9.1 - 10.8)	20.2 (17.9 - 21.3)	21.6 (19.4 - 22.9)	35.0 (31.3 - 36.9)	34.2 (30.7 - 35.9)	22.0 (19.8 - 23.1)
	2019	1.6 (1.4 - 1.8)	3.8 (3.4 - 4.2)	7.0 (6.3 - 7.6)	16.2 (14.5 - 17.7)	13.0 (11.7 - 14.1)	27.8 (25.0 - 30.1)	21.6 (19.5 - 23.4)	16.4 (14.8 - 17.7)
	% change	-64.2 (-69.0 - -59.4)	-57.3 (-63.1 - -51.7)	-53.9 (-61.5 - -48.0)	-44.5 (-53.7 - -37.4)	-42.6 (-51.3 - -36.2)	-36.3 (-46.0 - -29.2)	-48.9 (-56.7 - -43.3)	-40.4 (-49.4 - -33.8)
High SDI	1990	2.3 (2.2 - 2.4)	7.9 (7.6 - 8.3)	12.8 (12.5 - 13.3)	40.7 (39.7 - 42.3)	16.3 (15.9 - 16.7)	49.2 (48.2 - 50.4)	31.4 (30.8 - 32.4)	33.4 (32.8 - 34.5)
	2010	0.9 (0.9 - 1.0)	3.1 (2.9 - 3.3)	5.7 (5.5 - 5.9)	18.0 (17.5 - 18.6)	9.4 (9.1 - 9.8)	27.8 (26.8 - 29.1)	16.0 (15.5 - 16.6)	16.9 (16.4 - 17.5)
	2019	0.7 (0.7 - 0.8)	2.5 (2.3 - 2.6)	4.4 (4.0 - 4.6)	14.7 (13.6 - 15.5)	7.4 (6.8 - 7.9)	23.0 (21.3 - 24.9)	12.4 (11.6 - 13.3)	13.7 (12.8 - 14.7)
	% change	-69.0 (-71.1 - -66.8)	-68.5 (-70.6 - -66.3)	-66.0 (-68.2 - -64.4)	-64.0 (-66.2 - -62.2)	-54.8 (-58.0 - -51.3)	-53.2 (-56.5 - -49.6)	-60.4 (-62.9 - -58.1)	-58.9 (-61.5 - -56.6)

Unintentional injury

		10-14 years		15-19 years		20-24 years		10-24 years	
		Number (thousands)	Rate	Number (thousands)	Rate	Number (thousands)	Rate	Number (thousands)	Rate
Global	1990	52.3 (46.4 - 58.1)	19.0 (16.9 - 21.1)	60.2 (53.9 - 66.7)	22.8 (20.4 - 25.3)	66.5 (60.4 - 73.2)	26.8 (24.3 - 29.5)	178.9 (161.6 - 196.4)	22.7 (20.5 - 25.0)
	2010	53.8 (48.5 - 58.3)	17.1 (15.4 - 18.5)	60.4 (54.6 - 64.1)	19.2 (17.3 - 20.4)	75.8 (69.5 - 80.0)	24.3 (22.3 - 25.7)	190.0 (173.7 - 201.6)	20.2 (18.4 - 21.4)
	2019	28.0 (24.0 - 32.1)	8.5 (7.2 - 9.7)	34.9 (30.7 - 38.8)	11.0 (9.7 - 12.2)	47.7 (42.6 - 52.2)	15.7 (14.0 - 17.1)	110.7 (98.2 - 121.7)	11.6 (10.3 - 12.8)
	% change	-46.3 (-52.3 - -39.6)	-55.5 (-60.4 - -49.9)	-42.0 (-47.5 - -35.3)	-51.8 (-56.4 - -46.2)	-28.2 (-35.5 - -20.4)	-41.4 (-47.4 - -35.1)	-38.1 (-43.6 - -31.4)	-48.9 (-53.5 - -43.3)
Low SDI	1990	5.3 (4.0 - 6.4)	15.7 (11.9 - 19.1)	4.9 (3.8 - 6.2)	18.2 (14.1 - 22.8)	5.2 (4.1 - 6.4)	23.4 (18.4 - 28.9)	15.4 (12.0 - 18.7)	18.6 (14.5 - 22.6)
	2010	20.9 (18.9 - 22.9)	35.8 (32.4 - 39.3)	22.1 (20.3 - 24.2)	45.9 (42.3 - 50.3)	23.0 (21.2 - 25.0)	58.7 (54.1 - 64.1)	65.9 (60.6 - 71.8)	45.3 (41.6 - 49.3)
	2019	6.9 (5.6 - 8.8)	9.5 (7.6 - 12.1)	6.9 (5.7 - 8.4)	11.1 (9.1 - 13.4)	8.3 (7.0 - 9.8)	16.1 (13.6 - 19.0)	22.1 (18.5 - 26.7)	11.8 (9.9 - 14.3)
	% change	31.8 (4.7 - 69.7)	-39.4 (-51.8 - -21.9)	40.8 (16.0 - 75.8)	-39.3 (-50.0 - -24.2)	59.9 (32.7 - 97.6)	-31.1 (-42.9 - -14.9)	44.1 (21.3 - 77.2)	-36.2 (-46.4 - -21.6)
Low-middle SDI	1990	14.4 (11.8 - 17.3)	21.2 (17.3 - 25.5)	14.5 (12.1 - 17.0)	24.4 (20.4 - 28.6)	15.5 (13.2 - 18.5)	29.6 (25.3 - 35.4)	44.4 (38.2 - 52.0)	24.7 (21.3 - 28.9)
	2010	15.0 (12.6 - 17.1)	16.9 (14.3 - 19.3)	13.7 (11.7 - 15.3)	16.6 (14.2 - 18.6)	16.6 (14.6 - 18.1)	22.9 (20.2 - 24.9)	45.3 (39.5 - 49.6)	18.6 (16.2 - 20.4)
	2019	10.2 (8.4 - 12.1)	11.3 (9.2 - 13.3)	11.2 (9.5 - 12.8)	12.8 (10.8 - 14.6)	14.9 (12.9 - 16.8)	18.3 (15.9 - 20.7)	36.3 (31.3 - 41.0)	14.0 (12.1 - 15.8)
	% change	-29.1 (-39.5 - -16.0)	-46.9 (-54.7 - -37.1)	-23.1 (-34.0 - -10.2)	-47.8 (-55.2 - -39.0)	-3.9 (-20.8 - 11.5)	-38.2 (-49.1 - -28.3)	-18.3 (-29.3 - -5.9)	-43.5 (-51.1 - -34.9)
Middle SDI	1990	22.2 (19.8 - 24.0)	23.7 (21.2 - 25.7)	25.0 (21.8 - 27.6)	26.5 (23.2 - 29.4)	26.3 (23.7 - 28.9)	29.7 (26.7 - 32.7)	73.4 (65.4 - 79.8)	26.6 (23.7 - 28.9)
	2010	12.7 (11.0 - 13.6)	13.2 (11.5 - 14.2)	15.8 (13.5 - 17.0)	15.4 (13.2 - 16.5)	20.8 (18.2 - 22.4)	20.0 (17.4 - 21.5)	49.3 (42.9 - 52.5)	16.3 (14.2 - 17.4)

	2019	7.7 (6.8 - 8.5)	8.0 (7.1 - 8.9)	11.2 (9.4 - 12.6)	11.8 (9.9 - 13.3)	15.3 (13.1 - 17.0)	16.5 (14.1 - 18.4)	34.2 (29.5 - 37.8)	12.1 (10.4 - 13.3)
	% change	-65.2 (-68.9 - -60.9)	-66.2 (-69.8 - -62.1)	-55.1 (-61.0 - -48.6)	-55.4 (-61.2 - -48.9)	-42.0 (-49.5 - -34.6)	-44.5 (-51.7 - -37.4)	-53.4 (-59.0 - -47.9)	-54.7 (-60.0 - -49.2)
High-middle SDI	1990	8.4 (7.8 - 9.1)	16.6 (15.6 - 18.0)	11.8 (10.9 - 12.8)	22.7 (21.0 - 24.8)	14.1 (13.2 - 15.3)	27.2 (25.3 - 29.3)	34.3 (32.1 - 37.0)	22.2 (20.8 - 24.0)
	2010	4.0 (3.8 - 4.3)	9.5 (8.8 - 10.0)	6.6 (6.2 - 7.0)	13.0 (12.2 - 13.7)	11.8 (10.9 - 12.4)	19.1 (17.7 - 20.1)	22.4 (21.0 - 23.5)	14.4 (13.5 - 15.2)
	2019	2.5 (2.3 - 2.8)	6.0 (5.5 - 6.5)	4.3 (3.9 - 4.7)	10.0 (9.1 - 10.9)	6.9 (6.2 - 7.5)	14.7 (13.3 - 16.0)	13.7 (12.6 - 14.8)	10.4 (9.5 - 11.2)
	% change	-69.5 (-72.6 - -66.2)	-63.7 (-67.4 - -59.7)	-63.5 (-67.7 - -58.6)	-56.1 (-61.2 - -50.2)	-51.4 (-57.2 - -45.1)	-46.0 (-52.5 - -39.0)	-60.0 (-64.2 - -55.4)	-53.3 (-58.2 - -47.9)
High SDI	1990	2.0 (1.9 - 2.1)	6.8 (6.3 - 7.2)	4.0 (3.6 - 4.3)	12.7 (11.6 - 13.5)	5.3 (5.0 - 5.6)	16.1 (15.0 - 17.1)	11.3 (10.5 - 12.0)	12.0 (11.1 - 12.7)
	2010	0.8 (0.7 - 0.8)	2.6 (2.4 - 2.7)	1.7 (1.6 - 1.8)	5.4 (5.1 - 5.6)	3.0 (2.9 - 3.3)	9.0 (8.5 - 9.7)	5.5 (5.2 - 5.8)	5.8 (5.5 - 6.1)
	2019	0.6 (0.5 - 0.6)	2.0 (1.9 - 2.1)	1.3 (1.2 - 1.4)	4.4 (4.1 - 4.7)	2.4 (2.2 - 2.6)	7.4 (6.8 - 8.2)	4.2 (4.0 - 4.6)	4.7 (4.4 - 5.0)
	% change	-71.0 (-73.2 - -68.6)	-70.6 (-72.8 - -68.1)	-67.4 (-69.9 - -64.5)	-65.4 (-68.1 - -62.4)	-55.5 (-60.1 - -49.6)	-53.9 (-58.7 - -47.8)	-62.4 (-65.3 - -58.7)	-61.0 (-64.0 - -57.1)

Table S11: Injury-related mortality in absolute numbers and rates per 100,000 population for **females** overall and by age group, by socio-demographic index, transport injury and unintentional injury and trends over time 1990-2019

Transport injury

		10-14 years		15-19 years		20-24 years		10-24 years	
		Number (thousands)	Rate	Number (thousands)	Rate	Number (thousands)	Rate	Number (thousands)	Rate
Global	1990	13.5 (12.2 - 14.8)	5.1 (4.7 - 5.6)	24.1 (22.5 - 25.7)	9.4 (8.8 - 10.0)	24.1 (22.5 - 25.9)	9.9 (9.2 - 10.6)	61.6 (57.7 - 65.9)	8.1 (7.6 - 8.6)
	2010	10.8 (9.7 - 11.9)	3.7 (3.3 - 4.0)	20.1 (18.8 - 21.5)	6.7 (6.3 - 7.2)	24.6 (23.1 - 26.1)	8.0 (7.5 - 8.5)	55.5 (52.0 - 59.1)	6.2 (5.8 - 6.6)
	2019	8.9 (7.8 - 10.2)	2.9 (2.5 - 3.3)	16.0 (14.4 - 17.6)	5.3 (4.8 - 5.8)	18.3 (16.5 - 20.1)	6.2 (5.6 - 6.8)	43.2 (39.1 - 47.3)	4.8 (4.3 - 5.2)
	% change	-33.8 (-41.5 - -25.5)	-44.2 (-50.7 - -37.2)	-33.4 (-40.1 - -26.7)	-43.6 (-49.3 - -37.9)	-24.2 (-32.4 - -16.1)	-37.4 (-44.2 - -30.7)	-29.9 (-36.8 - -23.0)	-41.2 (-47.0 - -35.4)
Low SDI	1990	1.8 (1.4 - 2.2)	5.6 (4.4 - 6.9)	2.2 (1.8 - 2.7)	8.4 (6.7 - 10.3)	2.1 (1.7 - 2.6)	9.3 (7.6 - 11.3)	6.1 (5.1 - 7.3)	7.5 (6.2 - 9.0)
	2010	2.6 (2.1 - 3.3)	4.7 (3.8 - 5.8)	3.2 (2.7 - 4.0)	6.8 (5.7 - 8.5)	3.1 (2.6 - 3.8)	7.8 (6.5 - 9.5)	8.9 (7.6 - 11.3)	6.3 (5.3 - 7.9)
	2019	2.8 (2.3 - 3.7)	4.0 (3.2 - 5.2)	3.5 (2.8 - 4.6)	5.7 (4.6 - 7.5)	3.4 (2.8 - 4.3)	6.5 (5.3 - 8.3)	9.8 (8.0 - 12.6)	5.3 (4.3 - 6.8)
	% change	56.7 (25.1 - 97.3)	-28.9 (-43.2 - -10.5)	60.0 (26.4 - 105.6)	-31.4 (-45.8 - -11.9)	62.1 (29.2 - 100.5)	-29.7 (-43.9 - -13.0)	59.7 (31.2 - 92.3)	-29.8 (-42.3 - -15.4)
Low-middle SDI	1990	2.7 (2.3 - 3.2)	4.2 (3.5 - 4.9)	4.6 (4.0 - 5.2)	7.9 (7.0 - 9.0)	4.6 (4.0 - 5.1)	8.8 (7.8 - 9.9)	11.9 (10.5 - 13.2)	6.8 (6.0 - 7.6)
	2010	2.8 (2.4 - 3.2)	3.3 (2.9 - 3.8)	4.6 (4.1 - 5.2)	5.9 (5.3 - 6.6)	5.2 (4.7 - 5.7)	7.2 (6.5 - 7.9)	12.6 (11.3 - 13.8)	5.4 (4.9 - 5.9)
	2019	2.4 (2.0 - 2.8)	2.7 (2.3 - 3.2)	4.1 (3.5 - 4.7)	4.8 (4.2 - 5.5)	4.8 (4.1 - 5.5)	5.9 (5.0 - 6.8)	11.2 (9.8 - 12.6)	4.5 (3.9 - 5.0)
	% change	-13.1 (-27.4 - 3.2)	-34.6 (-45.4 - -22.3)	-11.1 (-26.2 - 4.4)	-39.1 (-49.4 - -28.4)	4.2 (-13.0 - 23.9)	-33.1 (-44.2 - -20.5)	-5.7 (-19.0 - 8.4)	-34.5 (-43.8 - -24.7)
Middle SDI	1990	5.6 (5.1 - 6.2)	6.3 (5.7 - 6.9)	8.7 (7.9 - 9.5)	9.5 (8.7 - 10.4)	9.1 (8.3 - 10.1)	10.4 (9.4 - 11.5)	23.4 (21.6 - 25.3)	8.7 (8.1 - 9.4)
	2010	3.8 (3.5 - 4.0)	4.3 (4.0 - 4.6)	7.1 (6.6 - 7.6)	7.3 (6.8 - 7.8)	9.2 (8.4 - 10.0)	8.9 (8.1 - 9.7)	20.0 (18.7 - 21.4)	6.9 (6.5 - 7.4)
	2019	2.5 (2.2 - 2.8)	2.9 (2.6 - 3.2)	4.7 (4.2 - 5.2)	5.3 (4.8 - 5.8)	5.7 (5.0 - 6.4)	6.4 (5.6 - 7.1)	12.9 (11.6 - 14.3)	4.8 (4.4 - 5.4)
	% change	-55.2 (-61.2 - -49.1)	-54.6 (-60.7 - -48.4)	-46.2 (-53.3 - -38.6)	-44.3 (-51.6 - -36.5)	-37.4 (-46.5 - -27.6)	-38.8 (-47.7 - -29.2)	-44.9 (-51.7 - -37.9)	-44.5 (-51.3 - -37.4)
High-middle SDI	1990	2.1 (1.9 - 2.2)	4.3 (4.0 - 4.6)	4.3 (4.0 - 4.6)	8.6 (8.1 - 9.3)	4.2 (3.9 - 4.6)	8.4 (7.7 - 9.2)	10.6 (10.0 - 11.4)	7.1 (6.7 - 7.7)
	2010	1.2 (1.1 - 1.2)	3.0 (2.8 - 3.1)	3.1 (2.9 - 3.2)	6.4 (6.2 - 6.7)	4.7 (4.5 - 5.0)	7.9 (7.5 - 8.4)	8.9 (8.6 - 9.3)	6.1 (5.9 - 6.4)
	2019	0.8 (0.7 - 0.9)	2.1 (1.9 - 2.2)	1.9 (1.8 - 2.1)	4.9 (4.5 - 5.3)	2.4 (2.2 - 2.7)	5.6 (5.1 - 6.1)	5.1 (4.8 - 5.5)	4.2 (3.9 - 4.6)
	% change	-61.4 (-65.3 - -57.1)	-52.1 (-56.9 - -46.7)	-55.2 (-59.3 - -51.0)	-43.7 (-48.8 - -38.4)	-42.9 (-49.8 - -35.0)	-33.3 (-41.4 - -24.1)	-51.5 (-56.1 - -46.7)	-40.8 (-46.4 - -34.9)
High SDI	1990	1.3 (1.2 - 1.3)	4.5 (4.4 - 4.7)	4.3 (4.2 - 4.4)	14.2 (13.9 - 14.6)	4.1 (4.0 - 4.2)	12.8 (12.5 - 13.2)	9.6 (9.4 - 9.8)	10.7 (10.5 - 10.9)
	2010	0.5 (0.5 - 0.5)	1.9 (1.8 - 1.9)	2.1 (2.0 - 2.2)	7.1 (6.9 - 7.3)	2.4 (2.3 - 2.5)	7.6 (7.4 - 8.0)	5.0 (4.9 - 5.2)	5.6 (5.5 - 5.8)
	2019	0.4 (0.4 - 0.5)	1.6 (1.5 - 1.7)	1.8 (1.7 - 1.9)	6.5 (6.1 - 6.8)	1.9 (1.8 - 2.1)	6.5 (6.2 - 7.0)	4.2 (4.0 - 4.4)	4.9 (4.7 - 5.1)
	% change	-66.2 (-68.3 - -63.9)	-65.6 (-67.8 - -63.2)	-57.8 (-60.4 - -55.7)	-54.6 (-57.4 - -52.3)	-52.1 (-55.1 - -48.7)	-49.1 (-52.3 - -45.5)	-56.5 (-58.6 - -54.3)	-54.2 (-56.4 - -51.9)

Unintentional injury

		10-14 years		15-19 years		20-24 years		10-24 years	
		Number (thousands)	Rate	Number (thousands)	Rate	Number (thousands)	Rate	Number (thousands)	Rate
Global	1990	24.4 (21.5 - 27.3)	9.3 (8.2 - 10.4)	21.8 (19.6 - 24.3)	8.5 (7.7 - 9.5)	21.1 (18.7 - 24.2)	8.6 (7.6 - 9.9)	67.3 (60.5 - 75.4)	8.8 (7.9 - 9.9)
	2010	27.0 (24.3 - 29.3)	9.2 (8.3 - 9.9)	25.2 (22.7 - 27.4)	8.4 (7.6 - 9.1)	27.0 (24.1 - 29.5)	8.8 (7.9 - 9.6)	79.2 (71.6 - 85.7)	8.8 (8.0 - 9.5)
	2019	14.0 (11.7 - 16.6)	4.5 (3.8 - 5.3)	14.1 (11.6 - 16.8)	4.7 (3.8 - 5.6)	15.9 (12.8 - 19.7)	5.4 (4.3 - 6.7)	44.1 (36.4 - 53.1)	4.8 (4.0 - 5.8)
	% change	-42.4 (-50.5 - -29.7)	-51.5 (-58.3 - -40.8)	-35.4 (-45.3 - -19.8)	-45.3 (-53.7 - -32.1)	-24.6 (-39.2 - -2.3)	-37.7 (-49.8 - -19.3)	-34.6 (-44.9 - -18.9)	-45.1 (-53.8 - -32.0)
Low SDI	1990	2.9 (2.2 - 3.7)	9.0 (7.0 - 11.4)	2.3 (1.9 - 2.8)	8.9 (7.2 - 10.7)	2.3 (1.9 - 2.8)	10.3 (8.5 - 12.6)	7.6 (6.2 - 9.1)	9.3 (7.6 - 11.2)
	2010	12.9 (11.7 - 14.0)	23.1 (21.0 - 25.1)	10.4 (9.6 - 11.3)	22.2 (20.4 - 24.1)	10.1 (9.3 - 11.0)	25.1 (23.1 - 27.4)	33.4 (30.6 - 36.3)	23.4 (21.4 - 25.4)
	2019	3.7 (3.0 - 4.6)	5.3 (4.2 - 6.5)	3.0 (2.4 - 3.8)	4.9 (3.9 - 6.1)	3.1 (2.4 - 3.9)	5.9 (4.6 - 7.6)	9.8 (7.9 - 12.3)	5.3 (4.3 - 6.6)
	% change	29.8 (3.3 - 73.6)	-41.1 (-53.1 - -21.2)	28.8 (1.9 - 73.8)	-44.8 (-56.3 - -25.5)	30.8 (0.5 - 81.2)	-43.2 (-56.4 - -21.4)	29.8 (3.0 - 73.9)	-42.9 (-54.7 - -23.5)
Low-middle SDI	1990	7.6 (6.1 - 9.1)	11.7 (9.5 - 14.1)	7.6 (6.3 - 9.0)	13.2 (11.0 - 15.5)	7.2 (6.0 - 8.7)	14.0 (11.6 - 16.9)	22.4 (18.8 - 26.5)	12.9 (10.8 - 15.2)
	2010	6.7 (5.4 - 7.9)	8.1 (6.4 - 9.5)	7.1 (6.0 - 8.1)	9.1 (7.7 - 10.4)	7.4 (6.1 - 8.5)	10.3 (8.5 - 11.8)	21.3 (17.8 - 24.1)	9.1 (7.6 - 10.3)
	2019	5.5 (4.3 - 6.8)	6.4 (5.0 - 8.0)	6.0 (4.7 - 7.3)	7.1 (5.6 - 8.7)	6.7 (5.3 - 8.6)	8.4 (6.5 - 10.7)	18.2 (14.5 - 22.4)	7.3 (5.8 - 8.9)
	% change	-27.2 (-40.7 - -4.5)	-45.1 (-55.4 - -28.1)	-21.3 (-36.3 - 4.2)	-46.1 (-56.3 - -28.6)	-7.0 (-29.5 - 30.4)	-40.4 (-54.7 - -16.3)	-18.7 (-34.6 - 8.1)	-43.5 (-54.6 - -24.9)
Middle SDI	1990	10.3 (9.4 - 11.3)	11.6 (10.5 - 12.7)	8.3 (7.4 - 9.2)	9.1 (8.1 - 10.1)	7.9 (6.9 - 9.1)	9.0 (7.9 - 10.3)	26.5 (24.0 - 29.4)	9.9 (8.9 - 10.9)
	2010	5.3 (4.8 - 5.7)	6.0 (5.4 - 6.5)	5.2 (4.4 - 5.8)	5.3 (4.5 - 6.0)	6.0 (5.0 - 6.9)	5.8 (4.8 - 6.6)	16.4 (14.2 - 18.2)	5.7 (4.9 - 6.3)
	2019	3.5 (3.0 - 4.0)	4.0 (3.4 - 4.6)	3.6 (2.9 - 4.4)	4.1 (3.3 - 5.0)	4.2 (3.2 - 5.4)	4.7 (3.6 - 6.0)	11.3 (9.2 - 13.7)	4.2 (3.5 - 5.1)

	% change	-66.0 (-70.4 - -59.8)	-65.6 (-70.0 - -59.3)	-56.7 (-63.9 - -46.0)	-55.2 (-62.7 - -44.1)	-46.9 (-58.2 - -28.5)	-48.1 (-59.1 - -30.1)	-57.4 (-64.4 - -46.4)	-57.1 (-64.1 - -45.9)
High-middle SDI	1990	2.8 (2.6 - 3.0)	5.9 (5.5 - 6.3)	2.8 (2.6 - 3.0)	5.5 (5.1 - 6.0)	2.6 (2.4 - 2.9)	5.2 (4.7 - 5.8)	8.2 (7.6 - 8.9)	5.5 (5.2 - 6.0)
	2010	1.5 (1.4 - 1.6)	3.7 (3.5 - 4.0)	1.8 (1.7 - 2.0)	3.8 (3.5 - 4.1)	2.6 (2.3 - 2.8)	4.4 (3.9 - 4.8)	5.9 (5.4 - 6.3)	4.0 (3.7 - 4.3)
	2019	1.0 (0.9 - 1.1)	2.5 (2.3 - 2.8)	1.2 (1.0 - 1.3)	2.9 (2.6 - 3.3)	1.4 (1.2 - 1.6)	3.3 (2.7 - 3.8)	3.6 (3.1 - 4.0)	2.9 (2.6 - 3.3)
	% change	-65.3 (-69.2 - -61.3)	-56.9 (-61.7 - -51.9)	-57.6 (-64.2 - -51.4)	-46.7 (-55.0 - -38.9)	-45.8 (-56.0 - -34.2)	-36.7 (-48.6 - -23.1)	-56.5 (-63.2 - -49.7)	-46.9 (-55.1 - -38.7)
High SDI	1990	0.7 (0.7 - 0.8)	2.6 (2.4 - 2.8)	0.8 (0.8 - 0.9)	2.8 (2.6 - 3.0)	1.0 (1.0 - 1.1)	3.2 (3.0 - 3.5)	2.6 (2.4 - 2.7)	2.9 (2.7 - 3.1)
	2010	0.3 (0.3 - 0.3)	1.1 (1.1 - 1.2)	0.4 (0.4 - 0.4)	1.4 (1.3 - 1.5)	0.6 (0.6 - 0.7)	2.0 (1.9 - 2.1)	1.4 (1.3 - 1.4)	1.5 (1.5 - 1.6)
	2019	0.3 (0.2 - 0.3)	0.9 (0.9 - 1.0)	0.3 (0.3 - 0.4)	1.2 (1.1 - 1.3)	0.5 (0.5 - 0.6)	1.7 (1.6 - 1.9)	1.1 (1.0 - 1.2)	1.3 (1.2 - 1.4)
	% change	-64.1 (-66.9 - -61.1)	-63.5 (-66.3 - -60.4)	-59.7 (-63.5 - -55.8)	-56.6 (-60.7 - -52.4)	-50.6 (-55.7 - -44.5)	-47.5 (-52.9 - -41.0)	-57.3 (-60.5 - -53.5)	-55.1 (-58.5 - -51.0)

Table S12: Level 4 breakdown of select unintentional injury mechanisms for adolescents 10-24 years, total deaths and deaths per 100,000 population, 1990 and 2019 and percentage change by SDI

		Deaths in 1990	Deaths in 2019	Deaths % change	Deaths/100,000 in 1990	Deaths/100,000 in 2019	Deaths per 100,000 % change
Global	**Poisonings**	12,555.3 (11,108.7 to 14,295.3)	8,755.4 (7,293.2 to 9,864.6)	-30.3% (-41.5 to -20.5)	0.8 (0.7 to 0.9)	0.5 (0.4 to 0.5)	-42.0% (-51.3 to -33.9)
	Poisoning by carbon monoxide	6,591.9 (5,440.2 to 7,825.3)	4,807.4 (3,699.0 to 5,549.1)	-27.1% (-42.8 to -14.4)	0.4 (0.4 to 0.5)	0.3 (0.2 to 0.3)	-39.3% (-52.4 to -28.8)
	Poisoning by other means	5,963.4 (4,965.5 to 7,050.9)	3,948.1 (3,274.1 to 4,865.6)	-33.8% (-43.3 to -22.4)	0.4 (0.3 to 0.5)	0.2 (0.2 to 0.3)	-44.9% (-52.8 to -35.5)
	**Exposure to mechanical forces**	23,488.9 (20,892.6 to 26,960.0)	16,721.1 (13,469.8 to 19,568.2)	-28.8% (-41.4 to -14.6)	1.5 (1.3 to 1.7)	0.9 (0.7 to 1.1)	-40.8% (-51.3 to -29.0)
	Unintentional firearm injuries	6,882.6 (5,425.7 to 8,218.4)	4,270.5 (3,367.7 to 5,564.4)	-38.0% (-49.8 to -17.2)	0.4 (0.4 to 0.5)	0.2 (0.2 to 0.3)	-48.4% (-58.3 to -31.1)
	Other exposure to mechanical forces	16,606.4 (14,398.0 to 19,657.5)	12,450.6 (9,498.9 to 14,541.6)	-25.0% (-42.0 to -8.0)	1.1 (0.9 to 1.3)	0.7 (0.5 to 0.8)	-37.6% (-51.7 to -23.5)
	**Animal contact**	16,142.0 (10,347.2 to 19,377.9)	13,552.1 (8,975.3 to 17,062.2)	-16.0% (-32.7 to 24.7)	1.0 (0.7 to 1.3)	0.7 (0.5 to 0.9)	-30.2% (-44.0 to 3.7)
	Venomous animal contact	14,381.2 (9,088.2 to 17,484.2)	11,957.0 (7,646.8 to 15,364.5)	-16.9% (-34.2 to 25.9)	0.9 (0.6 to 1.1)	0.6 (0.4 to 0.8)	-30.8% (-45.3 to 4.7)
	Non-venomous animal contact	1,760.8 (1,266.9 to 2,183.2)	1,595.1 (1,252.7 to 2,115.0)	-9.4% (-27.1 to 26.0)	0.1 (0.1 to 0.1)	0.1 (0.1 to 0.1)	-24.6% (-39.4 to 4.8)
	**Foreign body**	6,932.0 (6,557.4 to 7,371.0)	6,737.5 (6,088.3 to 7,516.5)	-2.8% (-13.0 to 7.1)	0.4 (0.4 to 0.5)	0.4 (0.3 to 0.4)	-19.1% (-27.7 to -10.9)
	Pulmonary aspiration and foreign body in airway	6,298.2 (5,956.9 to 6,704.1)	6,099.4 (5,510.1 to 6,800.3)	-3.2% (-12.6 to 6.0)	0.4 (0.4 to 0.4)	0.3 (0.3 to 0.4)	-19.4% (-27.3 to -11.9)
	Foreign body in eyes	-	-	-	-	-	-
	Foreign body in other body part	633.8 (486.6 to 735.9)	638.1 (494.1 to 813.4)	0.7% (-26.0 to 31.4)	0.0 (0.0 to 0.0)	0.0 (0.0 to 0.0)	-16.2% (-38.5 to 9.3)
Low SDI	**Poisonings**	1,574.1 (1,232.3 to 1,951.0)	2,174.2 (1,789.6 to 2,717.3)	38.1% (13.2 to 81.9)	1.0 (0.8 to 1.2)	0.6 (0.5 to 0.7)	-39.1% (-50.1 to -19.8)
	Poisoning by carbon monoxide	472.8 (304.0 to 704.0)	934.5 (692.4 to 1,318.3)	97.6% (40.6 to 185.8)	0.3 (0.2 to 0.4)	0.3 (0.2 to 0.4)	-12.8% (-38.0 to 26.0)
	Poisoning by other means	1,101.3 (780.0 to 1,364.8)	1,239.7 (960.5 to 1,579.8)	12.6% (-11.6 to 56.5)	0.7 (0.5 to 0.8)	0.3 (0.3 to 0.4)	-50.3% (-61.0 to -31.0)
	**Exposure to mechanical forces**	2,316.9 (1,868.4 to 2,925.1)	3,820.0 (2,932.2 to 5,238.6)	64.9% (33.7 to 134.1)	1.4 (1.1 to 1.8)	1.0 (0.8 to 1.4)	-27.3% (-41.0 to 3.3)
	Unintentional firearm injuries	879.2 (667.0 to 1,175.2)	1,262.0 (872.3 to 1,954.2)	43.5% (-0.2 to 123.4)	0.5 (0.4 to 0.7)	0.3 (0.2 to 0.5)	-36.7% (-56.0 to -1.4)
	Other exposure to mechanical forces	1,437.7 (1,041.9 to 1,990.1)	2,558.1 (1,937.9 to 3,372.1)	77.9% (38.2 to 140.7)	0.9 (0.6 to 1.2)	0.7 (0.5 to 0.9)	-21.5% (-39.1 to 6.1)
	**Animal contact**	3,408.5 (1,986.5 to 4,536.5)	3,903.2 (2,543.9 to 5,349.3)	14.5% (-18.1 to 82.2)	2.1 (1.2 to 2.8)	1.1 (0.7 to 1.4)	-49.5% (-63.9 to -19.6)
	Venomous animal contact	2,919.9 (1,578.7 to 3,998.7)	3,061.3 (1,864.1 to 4,273.0)	4.8% (-27.8 to 72.7)	1.8 (1.0 to 2.4)	0.8 (0.5 to 1.2)	-53.8% (-68.2 to -23.8)
	Non-venomous animal contact	488.5 (315.6 to 669.5)	841.9 (619.1 to 1,233.4)	72.3% (21.1 to 170.1)	0.3 (0.2 to 0.4)	0.2 (0.2 to 0.3)	-24.0% (-46.6 to 19.1)
	**Foreign body**	907.0 (771.6 to 1,045.7)	1,843.0 (1,553.4 to 2,187.6)	103.2% (64.9 to 145.1)	0.6 (0.5 to 0.6)	0.5 (0.4 to 0.6)	-10.4% (-27.3 to 8.1)
	Pulmonary aspiration and foreign body in airway	730.3 (611.1 to 859.1)	1,515.7 (1,254.6 to 1,813.9)	107.5% (71.9 to 150.8)	0.4 (0.4 to 0.5)	0.4 (0.3 to 0.5)	-8.5% (-24.2 to 10.6)
	Foreign body in eyes	-	-	-	-	-	-
	Foreign body in other body part	176.7 (125.2 to 231.5)	327.3 (225.1 to 455.6)	85.3% (26.5 to 153.6)	0.1 (0.1 to 0.1)	0.1 (0.1 to 0.1)	-18.3% (-44.2 to 11.9)
Low-middle	**Poisonings**	2,906.0 (2,348.4 to 3,583.9)	2,502.4 (2,075.0 to 2,914.9)	-13.9% (-33.0 to 3.7)	0.8 (0.7 to 1.0)	0.5 (0.4 to 0.6)	-40.3% (-53.6 to -28.1)
	Poisoning by carbon monoxide	1,150.1 (772.7 to 1,575.5)	1,235.6 (853.0 to 1,542.1)	7.4% (-23.2 to 44.3)	0.3 (0.2 to 0.4)	0.2 (0.2 to 0.3)	-25.5% (-46.7 to 0.1)

	Poisoning by other means	1,755.9 (1,395.9 to 2,163.2)	1,266.9 (1,042.7 to 1,628.8)	-27.9% (-42.4 to -8.6)	0.5 (0.4 to 0.6)	0.2 (0.2 to 0.3)	-50.0% (-60.1 to -36.6)
	**Exposure to mechanical forces**	5,196.5 (4,166.9 to 6,251.0)	4,793.1 (3,908.4 to 5,727.1)	-7.8% (-22.4 to 12.4)	1.5 (1.2 to 1.8)	0.9 (0.8 to 1.1)	-36.1% (-46.2 to -22.0)
	Unintentional firearm injuries	1,345.8 (986.0 to 1,742.0)	1,196.0 (934.3 to 1,566.4)	-11.1% (-33.2 to 29.2)	0.4 (0.3 to 0.5)	0.2 (0.2 to 0.3)	-38.4% (-53.7 to -10.4)
	Other exposure to mechanical forces	3,850.7 (2,982.7 to 4,772.6)	3,597.1 (2,801.0 to 4,313.3)	-6.6% (-23.7 to 15.6)	1.1 (0.8 to 1.3)	0.7 (0.5 to 0.8)	-35.2% (-47.1 to -19.9)
	**Animal contact**	9,604.7 (5,875.4 to 11,697.3)	7,819.7 (4,677.1 to 10,035.8)	-18.6% (-35.4 to 24.0)	2.7 (1.7 to 3.3)	1.5 (0.9 to 2.0)	-43.6% (-55.2 to -14.0)
	Venomous animal contact	9,055.2 (5,421.9 to 11,099.1)	7,330.7 (4,257.3 to 9,442.1)	-19.0% (-36.3 to 24.5)	2.6 (1.5 to 3.1)	1.4 (0.8 to 1.8)	-43.9% (-55.9 to -13.7)
	Non-venomous animal contact	549.5 (364.6 to 744.2)	489.0 (377.1 to 664.8)	-11.0% (-33.7 to 36.4)	0.2 (0.1 to 0.2)	0.1 (0.1 to 0.1)	-38.3% (-54.0 to -5.5)
	**Foreign body**	1,288.6 (1,167.9 to 1,423.2)	1,676.4 (1,468.0 to 1,918.1)	30.1% (9.5 to 49.5)	0.4 (0.3 to 0.4)	0.3 (0.3 to 0.4)	-9.8% (-24.1 to 3.7)
	Pulmonary aspiration and foreign body in airway	1,172.9 (1,065.9 to 1,299.7)	1,499.9 (1,305.1 to 1,737.4)	27.9% (9.0 to 47.2)	0.3 (0.3 to 0.4)	0.3 (0.3 to 0.3)	-11.3% (-24.4 to 2.0)
	Foreign body in eyes	-	-	-	-	-	-
	Foreign body in other body part	115.7 (85.3 to 140.0)	176.6 (135.3 to 229.4)	52.7% (7.9 to 108.9)	0.0 (0.0 to 0.0)	0.0 (0.0 to 0.0)	5.8% (-25.2 to 44.8)
Middle SDI	**Poisonings**	4,477.0 (3,937.7 to 5,133.0)	2,431.7 (1,810.9 to 2,739.4)	-45.7% (-61.8 to -35.4)	0.8 (0.7 to 0.9)	0.4 (0.3 to 0.5)	-46.2% (-62.1 to -36.0)
	Poisoning by carbon monoxide	2,408.3 (1,978.6 to 2,969.5)	1,372.4 (979.7 to 1,599.2)	-43.0% (-61.2 to -29.0)	0.4 (0.4 to 0.5)	0.2 (0.2 to 0.3)	-43.5% (-61.6 to -29.7)
	Poisoning by other means	2,068.7 (1,677.1 to 2,454.9)	1,059.3 (743.0 to 1,367.6)	-48.8% (-63.0 to -35.3)	0.4 (0.3 to 0.5)	0.2 (0.1 to 0.2)	-49.3% (-63.4 to -35.9)
	**Exposure to mechanical forces**	9,348.8 (8,029.6 to 10,916.1)	5,517.4 (4,036.4 to 6,590.7)	-41.0% (-57.0 to -25.6)	1.7 (1.5 to 2.0)	1.0 (0.7 to 1.2)	-41.5% (-57.4 to -26.3)
	Unintentional firearm injuries	2,521.7 (1,863.4 to 3,061.6)	1,042.1 (811.3 to 1,448.0)	-58.7% (-70.0 to -36.3)	0.5 (0.3 to 0.6)	0.2 (0.1 to 0.3)	-59.1% (-70.3 to -36.9)
	Other exposure to mechanical forces	6,827.1 (5,687.2 to 8,282.3)	4,475.3 (3,076.4 to 5,422.3)	-34.4% (-56.6 to -13.3)	1.3 (1.0 to 1.5)	0.8 (0.6 to 1.0)	-35.1% (-57.0 to -14.2)
	**Animal contact**	2,592.9 (1,941.8 to 3,005.0)	1,577.7 (1,281.7 to 1,982.2)	-39.2% (-51.0 to -12.2)	0.5 (0.4 to 0.6)	0.3 (0.2 to 0.4)	-39.7% (-51.5 to -13.0)
	Venomous animal contact	2,044.8 (1,569.0 to 2,412.0)	1,369.5 (1,091.5 to 1,759.5)	-33.0% (-47.5 to -0.6)	0.4 (0.3 to 0.4)	0.2 (0.2 to 0.3)	-33.6% (-47.9 to -1.5)
	Non-venomous animal contact	548.1 (335.1 to 651.6)	208.1 (174.5 to 241.2)	-62.0% (-70.1 to -43.1)	0.1 (0.1 to 0.1)	0.0 (0.0 to 0.0)	-62.4% (-70.3 to -43.7)
	**Foreign body**	2,471.8 (2,319.4 to 2,659.3)	1,868.6 (1,676.2 to 2,101.4)	-24.4% (-33.2 to -15.8)	0.5 (0.4 to 0.5)	0.3 (0.3 to 0.4)	-25.1% (-33.8 to -16.6)
	Pulmonary aspiration and foreign body in airway	2,246.7 (2,112.0 to 2,425.6)	1,785.1 (1,604.4 to 2,010.8)	-20.5% (-29.5 to -11.5)	0.4 (0.4 to 0.4)	0.3 (0.3 to 0.4)	-21.3% (-30.2 to -12.3)
	Foreign body in eyes	-	-	-	-	-	-
	Foreign body in other body part	225.1 (162.3 to 269.0)	83.5 (68.0 to 109.1)	-62.9% (-72.9 to -40.4)	0.0 (0.0 to 0.0)	0.0 (0.0 to 0.0)	-63.2% (-73.1 to -41.0)
High-middle SDI	**Poisonings**	2,703.9 (2,483.2 to 3,015.6)	1,310.1 (1,140.5 to 1,436.8)	-51.6% (-61.3 to -45.0)	0.9 (0.8 to 1.0)	0.5 (0.4 to 0.6)	-42.2% (-53.8 to -34.4)
	Poisoning by carbon monoxide	1,971.3 (1,791.8 to 2,201.0)	1,030.2 (868.6 to 1,137.0)	-47.7% (-58.1 to -40.3)	0.7 (0.6 to 0.7)	0.4 (0.3 to 0.4)	-37.7% (-50.0 to -28.8)
	Poisoning by other means	732.6 (634.0 to 909.2)	279.9 (230.1 to 388.1)	-61.8% (-69.7 to -49.8)	0.2 (0.2 to 0.3)	0.1 (0.1 to 0.2)	-54.4% (-63.8 to -40.2)
	**Exposure to mechanical forces**	4,196.7 (3,770.5 to 5,130.2)	1,850.0 (1,527.6 to 2,080.4)	-55.9% (-66.2 to -46.8)	1.4 (1.2 to 1.7)	0.7 (0.6 to 0.8)	-47.4% (-59.7 to -36.6)
	Unintentional firearm injuries	1,199.7 (949.8 to 1,463.6)	494.3 (348.9 to 595.2)	-58.8% (-68.3 to -48.1)	0.4 (0.3 to 0.5)	0.2 (0.1 to 0.2)	-50.9% (-62.2 to -38.1)
	Other exposure to mechanical forces	2,997.1 (2,631.8 to 3,839.1)	1,355.6 (1,120.2 to 1,524.1)	-54.8% (-67.0 to -44.3)	1.0 (0.9 to 1.3)	0.5 (0.4 to 0.6)	-46.1% (-60.6 to -33.6)
	**Animal contact**	495.7 (388.9 to 579.4)	220.5 (181.0 to 284.4)	-55.5% (-65.8 to -37.1)	0.2 (0.1 to 0.2)	0.1 (0.1 to 0.1)	-47.0% (-59.2 to -24.9)

	Venomous animal contact	341.9 (263.1 to 415.1)	180.3 (142.2 to 237.0)	-47.3% (-61.3 to -23.6)	0.1 (0.1 to 0.1)	0.1 (0.1 to 0.1)	-37.1% (-53.9 to -8.9)
	Non-venomous animal contact	153.9 (109.0 to 183.3)	40.2 (35.7 to 46.8)	-73.9% (-79.2 to -60.2)	0.1 (0.0 to 0.1)	0.0 (0.0 to 0.0)	-68.9% (-75.2 to -52.6)
	**Foreign body**	1,470.6 (1,418.5 to 1,537.6)	949.0 (880.1 to 1,015.1)	-35.5% (-40.5 to -30.6)	0.5 (0.5 to 0.5)	0.4 (0.3 to 0.4)	-23.0% (-29.0 to -17.2)
	Pulmonary aspiration and foreign body in airway	1,377.8 (1,325.7 to 1,442.0)	915.8 (849.9 to 978.5)	-33.5% (-38.6 to -28.7)	0.5 (0.4 to 0.5)	0.4 (0.3 to 0.4)	-20.7% (-26.7 to -14.9)
	Foreign body in eyes	-	-	-	-	-	-
	Foreign body in other body part	92.8 (71.9 to 110.0)	33.2 (28.3 to 42.3)	-64.2% (-72.8 to -46.4)	0.0 (0.0 to 0.0)	0.0 (0.0 to 0.0)	-57.3% (-67.6 to -36.1)
High SDI	**Poisonings**	888.8 (793.6 to 950.8)	332.1 (310.0 to 354.1)	-62.6% (-66.1 to -58.0)	0.5 (0.4 to 0.5)	0.2 (0.2 to 0.2)	-61.0% (-64.6 to -56.2)
	Poisoning by carbon monoxide	587.8 (496.7 to 652.0)	233.0 (216.2 to 249.9)	-60.4% (-65.2 to -53.8)	0.3 (0.3 to 0.4)	0.1 (0.1 to 0.1)	-58.6% (-63.7 to -51.7)
	Poisoning by other means	301.0 (273.9 to 375.6)	99.1 (91.4 to 106.8)	-67.1% (-73.0 to -63.0)	0.2 (0.1 to 0.2)	0.1 (0.1 to 0.1)	-65.6% (-71.8 to -61.4)
	**Exposure to mechanical forces**	2,414.4 (2,302.0 to 2,603.5)	723.1 (647.9 to 813.6)	-70.0% (-74.0 to -65.9)	1.3 (1.3 to 1.4)	0.4 (0.4 to 0.5)	-68.7% (-72.8 to -64.4)
	Unintentional firearm injuries	928.4 (721.7 to 1,041.4)	266.7 (231.4 to 331.3)	-71.3% (-76.1 to -63.4)	0.5 (0.4 to 0.6)	0.2 (0.1 to 0.2)	-70.0% (-75.0 to -61.8)
	Other exposure to mechanical forces	1,486.0 (1,383.1 to 1,690.0)	456.5 (389.7 to 529.9)	-69.3% (-74.3 to -63.5)	0.8 (0.8 to 0.9)	0.3 (0.2 to 0.3)	-67.9% (-73.1 to -61.8)
	**Animal contact**	37.6 (35.0 to 40.6)	28.2 (26.3 to 30.1)	-25.1% (-31.6 to -17.1)	0.0 (0.0 to 0.0)	0.0 (0.0 to 0.0)	-21.8% (-28.6 to -13.4)
	Venomous animal contact	17.7 (15.7 to 20.1)	13.1 (11.8 to 14.6)	-25.7% (-36.2 to -12.3)	0.0 (0.0 to 0.0)	0.0 (0.0 to 0.0)	-22.4% (-33.4 to -8.4)
	Non-venomous animal contact	19.9 (19.0 to 20.9)	15.0 (14.3 to 15.7)	-24.6% (-29.4 to -19.5)	0.0 (0.0 to 0.0)	0.0 (0.0 to 0.0)	-21.2% (-26.2 to -15.9)
	**Foreign body**	788.3 (754.7 to 811.9)	394.8 (377.5 to 415.3)	-49.9% (-52.5 to -46.0)	0.4 (0.4 to 0.4)	0.2 (0.2 to 0.2)	-47.7% (-50.3 to -43.6)
	Pulmonary aspiration and foreign body in airway	765.0 (734.4 to 787.4)	377.7 (361.1 to 397.3)	-50.6% (-53.1 to -46.8)	0.4 (0.4 to 0.4)	0.2 (0.2 to 0.2)	-48.4% (-51.0 to -44.4)
	Foreign body in eyes	-	-	-	-	-	-
	Foreign body in other body part	23.3 (20.5 to 25.5)	17.1 (15.8 to 18.5)	-26.4% (-34.8 to -13.3)	0.0 (0.0 to 0.0)	0.0 (0.0 to 0.0)	-23.1% (-31.9 to -9.4)

Table S13: Level 4 breakdown of select unintentional injury mechanisms for adolescents 10-24 years, total DALYs and DALYs per 100,000 population, 1990 and 2019 and percentage change by SDI

		DALYs (in thousands) 1990	DALYs (in thousands) 2019	DALYs % change	DALYs/100,000 in 1990	DALYs/100,000 in 2019	DALYs per 100,000 % change
Global	**Poisonings**	978.7 (873.9 to 1,109.4)	696.8 (597.9 to 779.9)	-28.8% (-39.1 to -19.8)	63.2 (56.4 to 71.6)	37.4 (32.1 to 41.9)	-40.8% (-49.3 to -33.3)
	Poisoning by carbon monoxide	481.2 (401.6 to 565.8)	355.5 (275.6 to 412.2)	-26.1% (-42.0 to -13.9)	31.1 (25.9 to 36.5)	19.1 (14.8 to 22.1)	-38.5% (-51.7 to -28.4)
	Poisoning by other means	497.5 (425.0 to 580.8)	341.3 (291.8 to 411.2)	-31.4% (-39.8 to -21.5)	32.1 (27.4 to 37.5)	18.3 (15.7 to 22.1)	-42.9% (-49.9 to -34.7)
	**Exposure to mechanical forces**	2,328.7 (2,010.3 to 2,733.4)	1,811.5 (1,482.2 to 2,188.8)	-22.2% (-32.5 to -12.5)	150.3 (129.8 to 176.5)	97.3 (79.6 to 117.6)	-35.3% (-43.9 to -27.2)
	Unintentional firearm injuries	520.5 (419.7 to 610.0)	334.0 (270.8 to 425.1)	-35.8% (-47.2 to -17.0)	33.6 (27.1 to 39.4)	17.9 (14.5 to 22.8)	-46.6% (-56.0 to -30.9)
	Other exposure to mechanical forces	1,808.3 (1,525.6 to 2,188.6)	1,477.5 (1,182.1 to 1,848.3)	-18.3% (-30.6 to -7.6)	116.7 (98.5 to 141.3)	79.4 (63.5 to 99.3)	-32.0% (-42.3 to -23.2)
	**Animal contact**	1,314.7 (893.6 to 1,560.2)	1,112.8 (787.4 to 1,385.3)	-15.4% (-30.4 to 19.9)	84.9 (57.7 to 100.7)	59.8 (42.3 to 74.4)	-29.6% (-42.1 to -0.2)
	Venomous animal contact	1,118.7 (736.3 to 1,350.6)	930.8 (620.7 to 1,174.4)	-16.8% (-33.1 to 22.4)	72.2 (47.5 to 87.2)	50.0 (33.3 to 63.1)	-30.8% (-44.4 to 1.8)
	Non-venomous animal contact	196.0 (149.8 to 245.6)	182.0 (145.1 to 231.0)	-7.1% (-19.6 to 16.2)	12.7 (9.7 to 15.9)	9.8 (7.8 to 12.4)	-22.8% (-33.1 to -3.4)
	**Foreign body**	764.6 (665.4 to 909.3)	765.2 (644.5 to 923.0)	0.1% (-6.8 to 6.6)	49.4 (43.0 to 58.7)	41.1 (34.6 to 49.6)	-16.7% (-22.5 to -11.3)
	Pulmonary aspiration and foreign body in airway	484.9 (458.7 to 518.3)	461.2 (420.3 to 510.4)	-4.9% (-13.7 to 3.5)	31.3 (29.6 to 33.5)	24.8 (22.6 to 27.4)	-20.9% (-28.2 to -13.9)
	Foreign body in eyes	93.8 (33.0 to 201.1)	98.0 (34.8 to 208.6)	4.5% (-3.2 to 16.6)	6.1 (2.1 to 13.0)	5.3 (1.9 to 11.2)	-13.1% (-19.5 to -3.0)
	Foreign body in other body part	185.8 (142.7 to 241.2)	206.0 (156.7 to 268.7)	10.9% (3.1 to 18.6)	12.0 (9.2 to 15.6)	11.1 (8.4 to 14.4)	-7.8% (-14.2 to -1.4)
Low SDI	**Poisonings**	117.8 (93.6 to 144.9)	163.8 (137.0 to 203.9)	39.1% (14.5 to 82.1)	71.9 (57.1 to 88.5)	44.1 (36.9 to 54.9)	-38.7% (-49.5 to -19.7)
	Poisoning by carbon monoxide	34.5 (22.5 to 51.4)	68.4 (51.3 to 95.3)	98.1% (42.7 to 183.4)	21.1 (13.7 to 31.4)	18.4 (13.8 to 25.6)	-12.6% (-37.1 to 25.0)
	Poisoning by other means	83.2 (60.8 to 102.1)	95.4 (75.6 to 120.3)	14.6% (-8.7 to 56.3)	50.8 (37.1 to 62.3)	25.7 (20.3 to 32.4)	-49.4% (-59.7 to -31.0)
	**Exposure to mechanical forces**	209.3 (172.9 to 258.0)	364.7 (290.5 to 464.7)	74.2% (48.0 to 128.0)	127.7 (105.5 to 157.5)	98.2 (78.2 to 125.1)	-23.1% (-34.7 to 0.5)
	Unintentional firearm injuries	65.7 (50.6 to 86.5)	95.9 (68.0 to 145.0)	45.9% (4.1 to 121.1)	40.1 (30.9 to 52.8)	25.8 (18.3 to 39.0)	-35.6% (-54.1 to -2.5)
	Other exposure to mechanical forces	143.6 (111.9 to 186.8)	268.8 (207.8 to 340.6)	87.2% (57.0 to 130.8)	87.6 (68.3 to 114.0)	72.4 (55.9 to 91.7)	-17.4% (-30.8 to 1.8)
	**Animal contact**	268.4 (163.8 to 348.0)	317.8 (219.2 to 423.7)	18.4% (-12.9 to 80.2)	163.8 (100.0 to 212.4)	85.6 (59.0 to 114.1)	-47.8% (-61.6 to -20.5)
	Venomous animal contact	222.3 (124.4 to 299.6)	238.3 (153.5 to 328.6)	7.2% (-24.6 to 71.9)	135.7 (75.9 to 182.9)	64.2 (41.3 to 88.5)	-52.7% (-66.7 to -24.2)
	Non-venomous animal contact	46.1 (32.0 to 60.6)	79.5 (60.8 to 107.5)	72.5% (30.6 to 143.4)	28.1 (19.5 to 37.0)	21.4 (16.4 to 28.9)	-23.9% (-42.4 to 7.4)
	**Foreign body**	103.6 (88.4 to 122.5)	213.7 (175.9 to 256.4)	106.2% (81.1 to 131.5)	63.2 (53.9 to 74.8)	57.5 (47.4 to 69.0)	-9.0% (-20.1 to 2.1)
	Pulmonary aspiration and foreign body in airway	55.9 (47.6 to 65.5)	113.6 (95.6 to 134.3)	103.3% (70.0 to 143.4)	34.1 (29.1 to 40.0)	30.6 (25.7 to 36.2)	-10.3% (-25.0 to 7.3)
	Foreign body in eyes	8.2 (3.0 to 17.0)	18.3 (6.6 to 38.1)	123.9% (121.9 to 126.2)	5.0 (1.8 to 10.4)	4.9 (1.8 to 10.2)	-1.3% (-2.1 to -0.2)
	Foreign body in other body part	39.6 (30.8 to 49.9)	81.8 (62.6 to 105.3)	106.7% (85.3 to 128.2)	24.1 (18.8 to 30.5)	22.0 (16.8 to 28.4)	-8.8% (-18.3 to 0.6)
Low-middle SDI	**Poisonings**	219.8 (180.0 to 267.9)	193.0 (161.9 to 223.0)	-12.2% (-31.1 to 4.2)	62.1 (50.9 to 75.7)	37.8 (31.7 to 43.7)	-39.1% (-52.2 to -27.7)
	Poisoning by carbon monoxide	84.5 (57.7 to 114.3)	91.6 (63.9 to 114.0)	8.5% (-21.5 to 44.7)	23.9 (16.3 to 32.3)	18.0 (12.5 to 22.3)	-24.8% (-45.6 to 0.3)
	Poisoning by other means	135.4 (109.2 to 165.6)	101.3 (84.9 to 126.7)	-25.2% (-38.9 to -7.0)	38.3 (30.9 to 46.8)	19.9 (16.6 to 24.8)	-48.1% (-57.6 to -35.5)
	**Exposure to mechanical forces**	460.3 (388.6 to 545.4)	463.2 (388.3 to 548.0)	0.6% (-13.3 to 15.8)	130.1 (109.8 to 154.1)	90.7 (76.1 to 107.4)	-30.2% (-39.9 to -19.7)
	Unintentional firearm injuries	102.7 (78.1 to 129.8)	93.0 (74.2 to 119.1)	-9.5% (-30.3 to 26.5)	29.0 (22.1 to 36.7)	18.2 (14.5 to 23.3)	-37.3% (-51.6 to -12.3)
	Other exposure to mechanical forces	357.5 (293.7 to 433.6)	370.2 (303.9 to 442.4)	3.5% (-11.2 to 20.3)	101.0 (83.0 to 122.5)	72.5 (59.5 to 86.7)	-28.2% (-38.5 to -16.6)
	**Animal contact**	744.4 (478.4 to 899.5)	606.9 (381.7 to 766.1)	-18.5% (-34.1 to 20.2)	210.4 (135.2 to 254.2)	118.9 (74.8 to 150.1)	-43.5% (-54.3 to -16.7)
	Venomous animal contact	683.2 (426.2 to 833.0)	550.6 (327.7 to 701.1)	-19.4% (-36.1 to 21.3)	193.1 (120.4 to 235.4)	107.9 (64.2 to 137.4)	-44.1% (-55.7 to -15.9)

	Non-venomous animal contact	61.1 (44.2 to 78.2)	56.2 (44.2 to 71.8)	-8.0% (-23.9 to 21.9)	17.3 (12.5 to 22.1)	11.0 (8.7 to 14.1)	-36.2% (-47.3 to -15.5)
	**Foreign body**	154.3 (131.0 to 185.4)	199.0 (164.7 to 243.1)	28.9% (16.1 to 40.5)	43.6 (37.0 to 52.4)	39.0 (32.3 to 47.6)	-10.6% (-19.5 to -2.6)
	Pulmonary aspiration and foreign body in airway	95.0 (86.6 to 104.5)	116.7 (102.8 to 133.6)	22.8% (6.2 to 39.5)	26.8 (24.5 to 29.5)	22.9 (20.1 to 26.2)	-14.8% (-26.4 to -3.3)
	Foreign body in eyes	20.1 (7.0 to 42.8)	28.3 (9.9 to 60.5)	40.7% (35.0 to 47.4)	5.7 (2.0 to 12.1)	5.5 (1.9 to 11.9)	-2.4% (-6.4 to 2.2)
	Foreign body in other body part	39.2 (29.8 to 51.2)	54.0 (41.7 to 70.5)	37.7% (26.7 to 49.2)	11.1 (8.4 to 14.5)	10.6 (8.2 to 13.8)	-4.5% (-12.2 to 3.4)
Middle SDI	**Poisonings**	337.6 (299.3 to 387.8)	189.8 (148.7 to 212.1)	-43.8% (-58.6 to -34.1)	62.0 (55.0 to 71.2)	34.5 (27.1 to 38.6)	-44.3% (-59.0 to -34.7)
	Poisoning by carbon monoxide	175.1 (145.4 to 213.4)	101.4 (74.0 to 117.3)	-42.1% (-60.3 to -28.8)	32.2 (26.7 to 39.2)	18.4 (13.5 to 21.3)	-42.7% (-60.6 to -29.4)
	Poisoning by other means	162.5 (136.1 to 192.3)	88.4 (68.4 to 109.9)	-45.6% (-57.9 to -33.4)	29.8 (25.0 to 35.3)	16.1 (12.4 to 20.0)	-46.1% (-58.3 to -34.0)
	**Exposure to mechanical forces**	817.6 (706.2 to 953.1)	543.5 (423.0 to 654.7)	-33.5% (-48.1 to -21.2)	150.2 (129.7 to 175.1)	98.9 (77.0 to 119.1)	-34.1% (-48.6 to -22.0)
	Unintentional firearm injuries	190.9 (145.4 to 227.9)	83.9 (66.6 to 113.3)	-56.0% (-66.8 to -35.6)	35.1 (26.7 to 41.9)	15.3 (12.1 to 20.6)	-56.4% (-67.1 to -36.2)
	Other exposure to mechanical forces	626.7 (528.9 to 752.7)	459.6 (343.8 to 561.1)	-26.7% (-45.6 to -10.9)	115.1 (97.2 to 138.3)	83.6 (62.6 to 102.1)	-27.3% (-46.1 to -11.7)
	**Animal contact**	229.7 (181.7 to 264.1)	145.5 (120.7 to 176.5)	-36.7% (-46.8 to -15.8)	42.2 (33.4 to 48.5)	26.5 (22.0 to 32.1)	-37.3% (-47.3 to -16.6)
	Venomous animal contact	169.8 (133.6 to 199.3)	115.3 (95.5 to 141.8)	-32.1% (-44.6 to -4.6)	31.2 (24.5 to 36.6)	21.0 (17.4 to 25.8)	-32.7% (-45.1 to -5.5)
	Non-venomous animal contact	60.0 (44.1 to 74.6)	30.2 (23.5 to 39.9)	-49.6% (-58.1 to -35.4)	11.0 (8.1 to 13.7)	5.5 (4.3 to 7.3)	-50.1% (-58.5 to -36.0)
	**Foreign body**	269.2 (232.4 to 324.4)	202.4 (171.0 to 245.1)	-24.8% (-30.6 to -19.0)	49.5 (42.7 to 59.6)	36.8 (31.1 to 44.6)	-25.5% (-31.3 to -19.7)
	Pulmonary aspiration and foreign body in airway	170.6 (160.7 to 184.0)	132.9 (119.6 to 148.6)	-22.1% (-30.6 to -13.5)	31.3 (29.5 to 33.8)	24.2 (21.8 to 27.0)	-22.8% (-31.2 to -14.3)
	Foreign body in eyes	37.5 (12.5 to 82.3)	30.0 (10.6 to 63.6)	-20.0% (-27.6 to -5.5)	6.9 (2.3 to 15.1)	5.5 (1.9 to 11.6)	-20.7% (-28.3 to -6.3)
	Foreign body in other body part	61.2 (46.7 to 78.3)	39.6 (29.4 to 53.4)	-35.3% (-41.5 to -27.5)	11.2 (8.6 to 14.4)	7.2 (5.4 to 9.7)	-35.9% (-42.0 to -28.1)
High-middle SDI	**Poisonings**	216.3 (197.0 to 244.4)	107.4 (95.9 to 118.0)	-50.3% (-58.7 to -44.5)	71.5 (65.1 to 80.8)	42.3 (37.8 to 46.5)	-40.8% (-50.8 to -33.8)
	Poisoning by carbon monoxide	143.0 (130.7 to 158.7)	75.4 (64.6 to 82.9)	-47.3% (-57.3 to -40.2)	47.3 (43.2 to 52.4)	29.7 (25.5 to 32.7)	-37.1% (-49.0 to -28.7)
	Poisoning by other means	73.3 (62.2 to 89.6)	32.0 (26.0 to 40.5)	-56.3% (-62.7 to -48.0)	24.2 (20.5 to 29.6)	12.6 (10.3 to 16.0)	-47.9% (-55.5 to -37.9)
	**Exposure to mechanical forces**	508.2 (423.2 to 622.9)	261.0 (207.7 to 335.1)	-48.6% (-56.4 to -43.1)	167.9 (139.8 to 205.8)	102.9 (81.9 to 132.1)	-38.8% (-48.0 to -32.2)
	Unintentional firearm injuries	91.3 (74.2 to 109.2)	39.2 (28.5 to 46.4)	-57.1% (-66.5 to -47.3)	30.2 (24.5 to 36.1)	15.4 (11.2 to 18.3)	-48.8% (-60.1 to -37.2)
	Other exposure to mechanical forces	416.9 (332.6 to 527.1)	221.8 (172.7 to 295.0)	-46.8% (-55.4 to -41.2)	137.8 (109.9 to 174.2)	87.4 (68.1 to 116.2)	-36.6% (-46.8 to -29.8)
	**Animal contact**	56.9 (46.2 to 68.4)	29.1 (23.5 to 36.2)	-48.9% (-56.5 to -37.6)	18.8 (15.3 to 22.6)	11.5 (9.3 to 14.3)	-39.0% (-48.1 to -25.5)
	Venomous animal contact	36.1 (28.7 to 43.9)	20.1 (16.0 to 24.8)	-44.2% (-54.0 to -28.7)	11.9 (9.5 to 14.5)	7.9 (6.3 to 9.8)	-33.5% (-45.2 to -15.0)
	Non-venomous animal contact	20.9 (16.1 to 27.3)	9.0 (6.3 to 12.8)	-56.9% (-64.4 to -47.6)	6.9 (5.3 to 9.0)	3.5 (2.5 to 5.1)	-48.6% (-57.6 to -37.5)
	**Foreign body**	153.8 (134.4 to 182.0)	96.8 (83.7 to 115.3)	-37.0% (-40.6 to -33.9)	50.8 (44.4 to 60.1)	38.1 (33.0 to 45.4)	-24.9% (-29.2 to -21.1)
	Pulmonary aspiration and foreign body in airway	105.4 (100.8 to 111.4)	68.3 (63.1 to 73.1)	-35.2% (-39.9 to -30.7)	34.8 (33.3 to 36.8)	26.9 (24.9 to 28.8)	-22.7% (-28.3 to -17.4)
	Foreign body in eyes	19.0 (6.4 to 41.7)	13.0 (4.5 to 28.0)	-31.8% (-36.0 to -25.1)	6.3 (2.1 to 13.8)	5.1 (1.8 to 11.0)	-18.7% (-23.6 to -10.7)
	Foreign body in other body part	29.3 (21.9 to 39.0)	15.5 (11.4 to 21.3)	-47.0% (-51.4 to -41.9)	9.7 (7.3 to 12.9)	6.1 (4.5 to 8.4)	-36.8% (-42.1 to -30.7)
High SDI	**Poisonings**	86.8 (76.9 to 98.8)	42.5 (35.2 to 51.4)	-51.1% (-56.0 to -45.1)	47.3 (41.9 to 53.9)	24.2 (20.1 to 29.3)	-48.9% (-54.0 to -42.7)
	Poisoning by carbon monoxide	44.0 (37.0 to 48.9)	18.6 (16.9 to 20.6)	-57.7% (-63.1 to -51.0)	24.0 (20.2 to 26.6)	10.6 (9.6 to 11.7)	-55.8% (-61.4 to -48.8)
	Poisoning by other means	42.8 (33.5 to 53.7)	23.9 (17.0 to 32.4)	-44.2% (-51.9 to -38.7)	23.3 (18.3 to 29.3)	13.6 (9.7 to 18.5)	-41.7% (-49.8 to -36.0)
	**Exposure to mechanical forces**	331.9 (268.4 to 423.7)	177.3 (129.5 to 250.8)	-46.6% (-52.6 to -40.5)	181.0 (146.4 to 231.1)	101.0 (73.8 to 142.9)	-44.2% (-50.5 to -37.8)
	Unintentional firearm injuries	69.3 (54.3 to 77.0)	21.3 (18.5 to 25.8)	-69.2% (-73.9 to -61.5)	37.8 (29.6 to 42.0)	12.1 (10.6 to 14.7)	-67.9% (-72.8 to -59.8)
	Other exposure to mechanical forces	262.6 (201.7 to 356.2)	156.0 (109.0 to 228.3)	-40.6% (-47.0 to -34.5)	143.2 (110.0 to 194.3)	88.9 (62.1 to 130.0)	-37.9% (-44.7 to -31.6)
	**Animal contact**	14.9 (10.2 to 20.6)	13.2 (8.9 to 18.4)	-11.6% (-14.1 to -9.7)	8.1 (5.6 to 11.2)	7.5 (5.1 to 10.5)	-7.7% (-10.2 to -5.7)
	Venomous animal contact	7.1 (4.9 to 9.8)	6.3 (4.3 to 8.6)	-12.1% (-15.7 to -8.9)	3.9 (2.7 to 5.3)	3.6 (2.4 to 4.9)	-8.2% (-11.9 to -4.8)

Non-venomous animal contact	7.8 (5.1 to 12.1)	6.9 (4.4 to 10.8)	-11.2% (-13.7 to -9.3)	4.2 (2.8 to 6.6)	3.9 (2.5 to 6.2)	-7.3% (-9.9 to -5.2)
**Foreign body**	83.1 (73.0 to 98.0)	52.8 (43.6 to 66.6)	-36.5% (-40.8 to -31.5)	45.3 (39.8 to 53.4)	30.1 (24.8 to 37.9)	-33.6% (-38.2 to -28.5)
Pulmonary aspiration and foreign body in airway	57.7 (55.4 to 59.7)	29.4 (27.8 to 31.0)	-49.1% (-51.5 to -45.4)	31.5 (30.2 to 32.5)	16.7 (15.9 to 17.7)	-46.8% (-49.4 to -43.0)
Foreign body in eyes	9.0 (3.3 to 19.1)	8.4 (3.1 to 17.8)	-6.4% (-8.5 to -4.1)	4.9 (1.8 to 10.4)	4.8 (1.8 to 10.2)	-2.2% (-4.5 to 0.1)
Foreign body in other body part	16.4 (11.7 to 23.0)	15.0 (10.5 to 21.3)	-8.5% (-10.7 to -6.4)	8.9 (6.4 to 12.6)	8.6 (6.0 to 12.1)	-4.4% (-6.7 to -2.2)

Figure S1: Percentage change in transport injury DALYs per 100,000 population in adolescents from 1990 to 2019

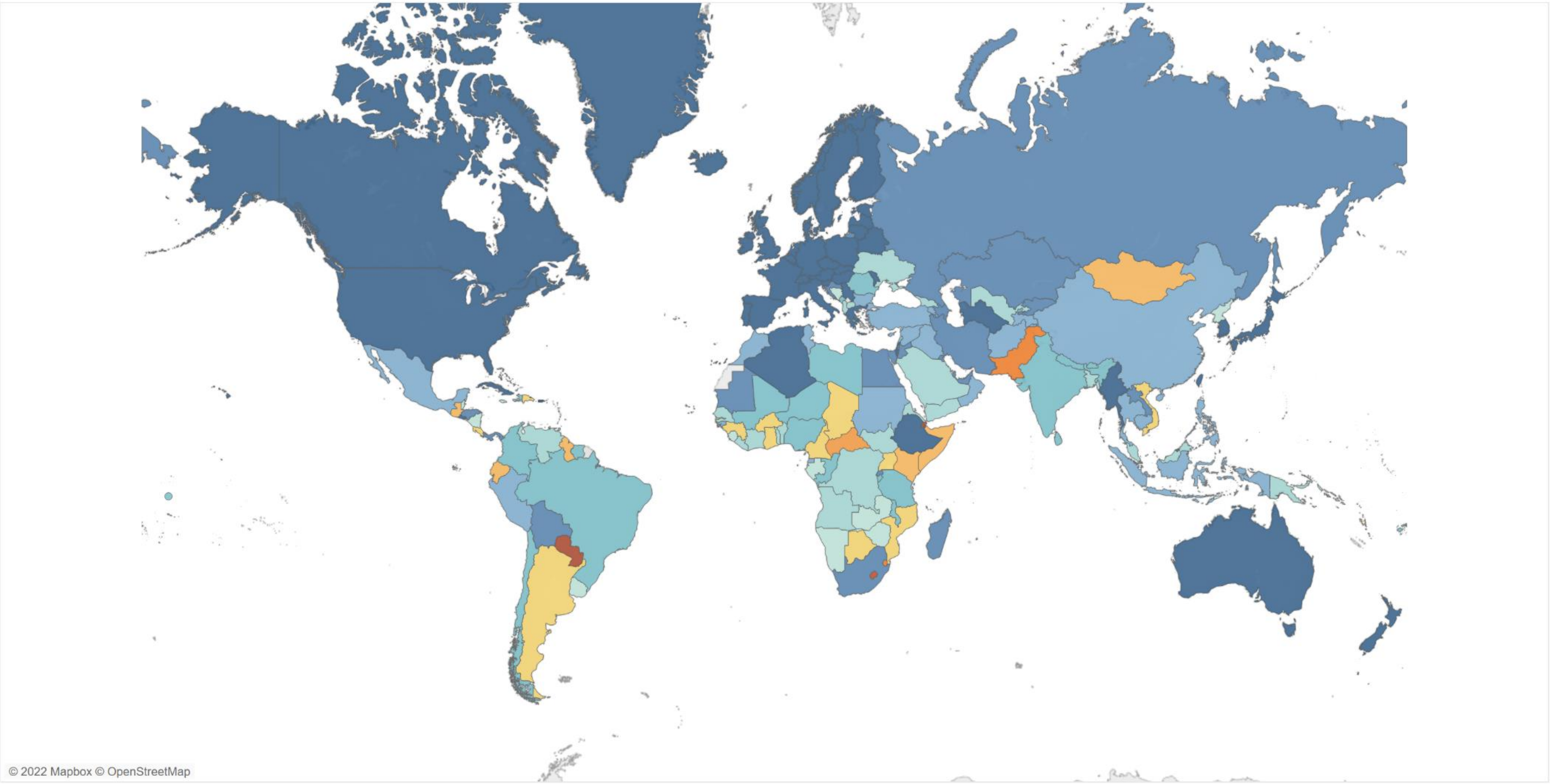

decrease >=50%	decrease of 20-30%	increase of 0-10%	increase of 30-40%
decrease of 40-50%	decrease of 10-20%	increase of 10-20%	increase of 40-50%
decrease of 30-40%	decrease of 0-10%	increase of 20-30%	increase of >=50%

Figure S2: Percentage change in transport injury and unintentional injury fatality rates in adolescents per 100,000 population overall and by sex and SDI, between 1990-2010 and 2010-2019

		Both		Female		Male	
		1990 to 2010	2010 to 2019	1990 to 2010	2010 to 2019	1990 to 2010	2010 to 2019
Transport injuries	Global	-17.0 (-26.0 to -11.9)	-20.9 (-26.4 to -15.7)	-23.8 (-28.4 to -19.0)	-22.9 (-28.7 to -17.4)	-15.4 (-27.1 to -9.5)	-20.5 (-26.3 to -14.8)
	High SDI	-48.8 (-50.2 to -47.3)	-17.4 (-22.0 to -13.5)	-47.3 (-48.8 to -45.7)	-13.1 (-17.4 to -9.0)	-49.5 (-51.0 to -47.8)	-18.7 (-24.1 to -14.2)
	High-middle SDI	-18.2 (-28.7 to -12.2)	-26.3 (-32.1 to -20.7)	-14.4 (-21.3 to -7.5)	-30.9 (-36.4 to -24.9)	-19.8 (-32.5 to -12.5)	-25.7 (-32.5 to -19.2)
	Middle SDI	-10.6 (-22.0 to -3.4)	-23.8 (-30.3 to -17.5)	-20.6 (-27.6 to -12.3)	-30.0 (-36.9 to -23.3)	-8.2 (-22.9 to 0.7)	-22.8 (-30.0 to -15.9)
	Low-middle SDI	-6.0 (-17.8 to 3.0)	-17.2 (-24.5 to -9.4)	-20.8 (-29.4 to -12.9)	-17.2 (-26.2 to -8.2)	-1.9 (-17.3 to 8.5)	-16.9 (-25.0 to -8.0)
	Low SDI	-5.6 (-15.3 to 6.8)	-15.7 (-24.0 to -6.4)	-16.9 (-27.9 to -4.1)	-15.5 (-26.0 to -5.3)	-1.6 (-12.2 to 12.7)	-15.6 (-24.4 to -5.8)
Unintentional injuries	Global	-8.1 (-13.2 to -2.2)	-43.1 (-47.2 to -38.3)	-0.5 (-9.3 to 11.8)	-44.9 (-51.3 to -36.6)	-11.3 (-17.2 to -5.3)	-42.5 (-46.7 to -37.8)
	High SDI	-50.7 (-53.9 to -47.1)	-18.3 (-22.8 to -13.3)	-46.8 (-50.1 to -43.2)	-15.6 (-20.6 to -9.7)	-51.9 (-55.5 to -47.8)	-19.0 (-23.8 to -13.7)
	High-middle SDI	-33.0 (-38.9 to -27.9)	-27.5 (-32.6 to -22.4)	-26.9 (-35.1 to -19.4)	-27.4 (-33.8 to -20.2)	-35.0 (-40.8 to -29.5)	-28.1 (-33.9 to -21.9)
	Middle SDI	-39.4 (-44.7 to -34.5)	-25.6 (-31.0 to -19.8)	-42.4 (-49.3 to -34.6)	-25.5 (-33.8 to -15.3)	-38.7 (-44.2 to -33.0)	-26.0 (-32.5 to -19.5)
	Low-middle SDI	-26.1 (-31.9 to -18.4)	-23.5 (-29.5 to -16.4)	-29.2 (-38.5 to -14.9)	-20.2 (-30.9 to -7.6)	-24.8 (-32.9 to -17.0)	-24.8 (-32.2 to -17.5)
	Low SDI	146.0 (115.3 to 189.2)	-75.0 (-78.5 to -71.0)	150.2 (107.7 to 203.9)	-77.2 (-81.1 to -72.0)	143.9 (106.0 to 201.7)	-73.9 (-77.6 to -69.7)

decrease >=20% | decrease of 10-20% | decrease of 0-10% | increase of 0-10% | increase of >=20%

Figure S3: Percentage change in transport injury and unintentional injury disability adjusted life year (DALY) rates per 100,000 population overall and by sex and SDI, between 1990-2010 and 2010-2019

		Both		Female		Male	
		1990 to 2010	2010 to 2019	1990 to 2010	2010 to 2019	1990 to 2010	2010 to 2019
Transport injuries	Global	-15.9 (-24.3 to -11.2)	-20.7 (-25.7 to -15.9)	-22.1 (-26.5 to -17.8)	-21.7 (-26.8 to -16.9)	-14.3 (-25.6 to -8.7)	-20.4 (-25.8 to -15.1)
	High SDI	-48.5 (-49.7 to -47.1)	-16.7 (-20.9 to -12.9)	-46.7 (-48.2 to -45.1)	-11.5 (-15.4 to -7.7)	-49.2 (-50.7 to -47.6)	-18.4 (-23.5 to -14.1)
	High-middle SDI	-18.2 (-28.1 to -12.6)	-25.4 (-30.8 to -20.1)	-15.0 (-21.1 to -9.0)	-28.7 (-33.7 to -23.5)	-19.8 (-31.9 to -12.8)	-25.0 (-31.5 to -18.9)
	Middle SDI	-10.2 (-21.1 to -3.4)	-23.6 (-29.7 to -17.5)	-19.7 (-26.2 to -12.3)	-28.6 (-34.7 to -22.6)	-7.8 (-21.8 to 0.6)	-22.7 (-29.5 to -16.1)
	Low-middle SDI	-3.8 (-14.7 to 4.5)	-18.2 (-24.6 to -11.6)	-17.3 (-25.0 to -10.1)	-18.1 (-25.4 to -10.7)	0.2 (-14.0 to 10.0)	-18.0 (-25.2 to -10.3)
	Low SDI	-5.5 (-14.5 to 5.7)	-15.7 (-23.2 to -7.1)	-16.0 (-25.9 to -4.7)	-14.9 (-24.2 to -6.0)	-1.6 (-11.5 to 11.8)	-15.8 (-23.9 to -6.7)
Unintentional injuries	Global	-10.5 (-14.8 to -6.2)	-35.8 (-39.9 to -31.7)	-5.7 (-12.0 to 2.7)	-33.6 (-39.6 to -27.2)	-12.9 (-18.0 to -7.8)	-36.9 (-41.1 to -32.9)
	High SDI	-38.8 (-42.3 to -35.5)	-0.2 (-4.5 to 3.6)	-32.1 (-35.2 to -29.5)	8.7 (4.6 to 11.5)	-41.6 (-45.3 to -38.1)	-4.1 (-8.6 to -0.1)
	High-middle SDI	-31.2 (-35.9 to -27.2)	-19.2 (-23.7 to -14.8)	-25.1 (-30.6 to -20.5)	-13.1 (-18.3 to -8.1)	-33.6 (-38.4 to -29.0)	-21.8 (-26.7 to -16.7)
	Middle SDI	-36.8 (-41.3 to -32.6)	-20.4 (-25.2 to -15.6)	-37.3 (-43.0 to -30.8)	-17.0 (-23.7 to -9.7)	-36.9 (-42.1 to -31.9)	-22.2 (-27.7 to -16.5)
	Low-middle SDI	-25.0 (-30.0 to -18.4)	-20.1 (-25.6 to -14.3)	-27.6 (-35.2 to -16.2)	-15.5 (-23.9 to -5.8)	-23.8 (-30.9 to -16.9)	-22.4 (-28.8 to -15.9)
	Low SDI	119.3 (94.7 to 152.3)	-69.8 (-73.4 to -65.8)	112.7 (83.3 to 147.9)	-69.6 (-73.8 to -64.5)	123.0 (92.6 to 170.3)	-69.9 (-73.6 to -65.8)

■ decrease >=20% ■ decrease of 10-20% ■ decrease of 0-10% ■ increase of 0-10% ■ increase of >=20%

Figure S4: The percentage change in DALYs per 100,000 population from 1990 to 2019 for each injury mechanism by SDI group

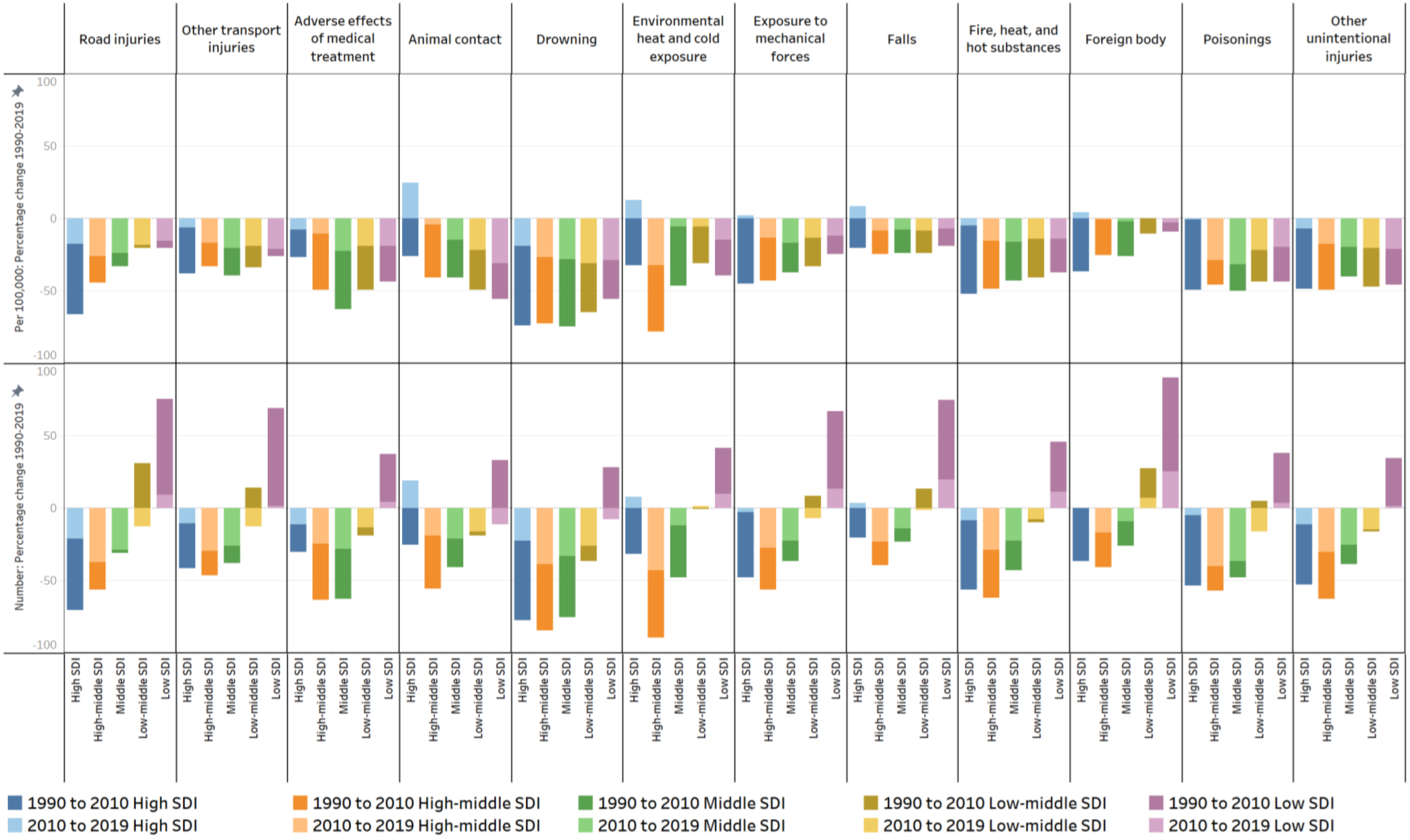

* Excludes exposure to forces of nature due to extreme fluctuations

Figure S5: Transport injury DALY rates per 100,000 population, 1990 vs 2019 by sex and country (coloured according to SDI), adolescents 10-24 years

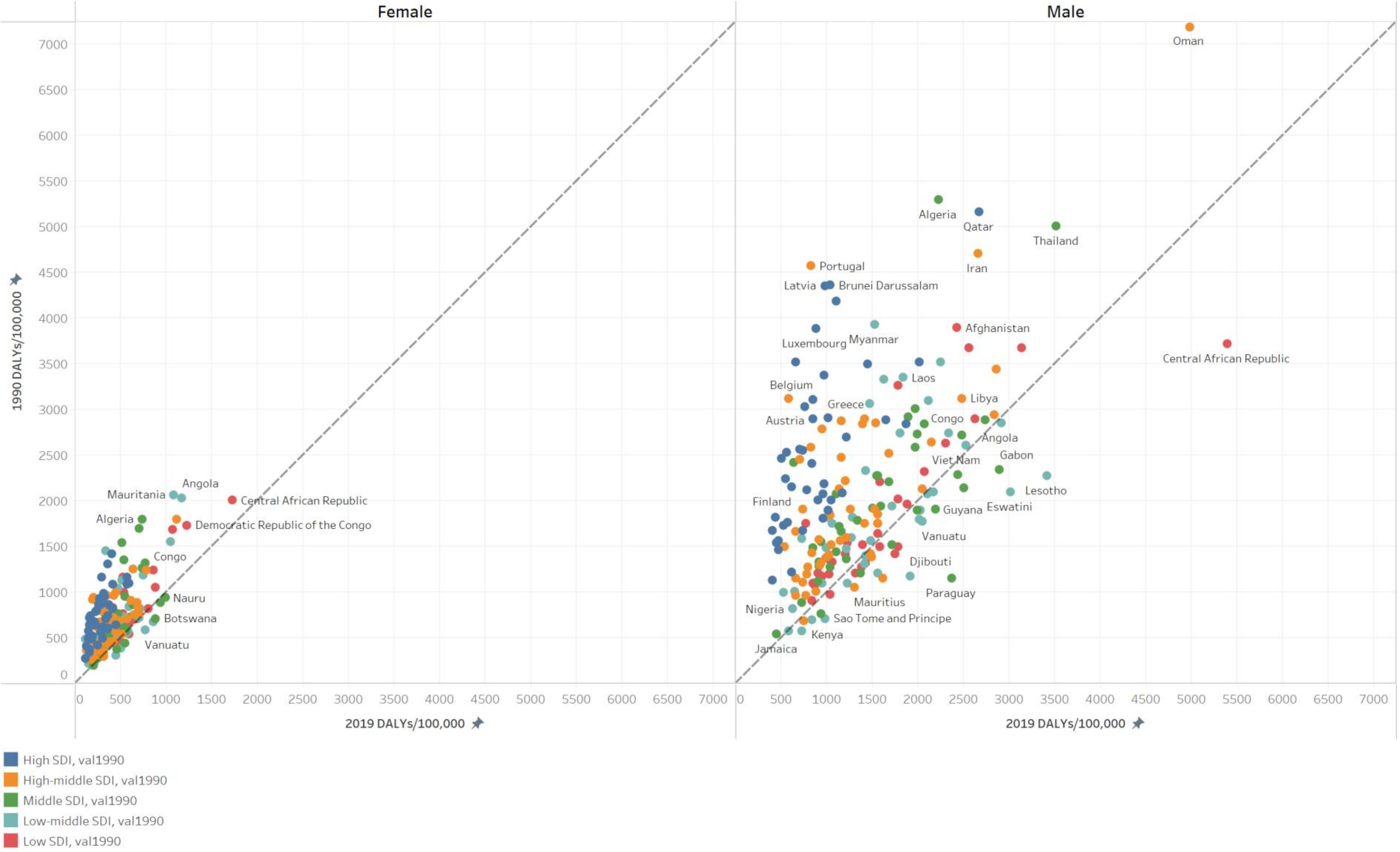

Figure S6: Heatmap of transport injury DALYs and deaths rates and percentage change 1990-2019 by country, adolescents 10-24 years

	DALYs (Disability-Adjusted Life Years)					Deaths				
	Transport injuries			Road injuries	Other transport injuries	Transport injuries			Road injuries	Other transport injuries
	DALYs/100,000 1990	DALYs/100,000 2019	% change, 1990 to 2019	% change, 1990 to 2019	% change, 1990 to 2019	Deaths/100,000 1990	Deaths/100,000 2019	% change, 1990 to 2019	% change, 1990 to 2019	% change, 1990 to 2019
Afghanistan	2,451.26	1,505.78	-39%	-38%	-56%	33.81	20.89	-38%	-37%	-59%
Albania	879.10	767.97	-13%	-15%	18%	10.87	9.57	-12%	-15%	29%
Algeria	3,573.54	1,501.54	-58%	-58%	-32%	48.79	20.69	-58%	-58%	-34%
American Samoa	819.25	578.97	-29%	-28%	-41%	11.24	7.91	-30%	-28%	-42%
Andorra	1,335.70	721.40	-46%	-46%	-39%	18.39	9.79	-47%	-47%	-41%
Angola	2,439.19	2,025.65	-17%	-15%	-47%	33.17	27.79	-16%	-15%	-48%
Antigua and Barbuda	660.53	512.10	-22%	-26%	57%	8.68	6.72	-23%	-26%	76%
Argentina	924.41	965.28	4%	9%	-36%	12.33	13.17	7%	12%	-36%
Armenia	665.53	406.06	-39%	-41%	29%	8.58	5.16	-40%	-42%	45%
Australia	1,752.16	517.39	-70%	-71%	-68%	24.42	6.95	-72%	-72%	-71%
Austria	1,937.64	505.41	-74%	-75%	-39%	27.01	6.72	-75%	-76%	-40%
Azerbaijan	707.78	424.31	-40%	-39%	-53%	9.13	5.35	-41%	-40%	-63%
Bahamas	1,444.82	1,374.49	-5%	-37%	495%	19.68	18.91	-4%	-38%	541%
Bahrain	1,767.17	1,037.20	-41%	-39%	-76%	23.96	14.23	-41%	-38%	-80%
Bangladesh	689.24	560.78	-19%	-17%	-29%	8.23	6.29	-24%	-22%	-31%
Barbados	949.99	592.27	-38%	-38%	-5%	12.66	7.76	-39%	-39%	-2%
Belarus	1,779.05	661.36	-63%	-63%	-13%	23.50	8.30	-65%	-65%	-13%
Belgium	2,041.94	567.35	-72%	-74%	8%	28.55	7.67	-73%	-74%	9%
Belize	1,293.05	1,254.97	-3%	-7%	91%	17.19	17.17	-0%	-5%	113%
Benin	1,019.73	838.47	-18%	-16%	-33%	13.21	10.78	-18%	-17%	-34%
Bermuda	1,272.76	563.28	-56%	-58%	8%	17.22	7.20	-58%	-60%	11%
Bhutan	887.72	634.54	-29%	-28%	-39%	10.97	7.48	-32%	-31%	-42%
Bolivia	1,259.47	707.61	-44%	-45%	-15%	16.90	9.61	-43%	-45%	-12%
Bosnia and Herzegovina	484.02	476.49	-2%	-2%	5%	5.06	5.11	1%	-0%	14%
Botswana	1,672.53	1,686.14	1%	1%	-7%	23.01	23.17	1%	1%	-6%
Brazil	1,666.23	1,232.92	-26%	-25%	-47%	22.83	17.11	-25%	-24%	-46%
Brunei Darussalam	2,651.70	747.61	-72%	-72%	-71%	37.43	10.30	-72%	-73%	-72%
Bulgaria	1,324.98	857.19	-35%	-34%	-49%	16.79	10.62	-37%	-35%	-50%
Burkina Faso	1,096.85	1,127.93	3%	4%	-8%	14.46	14.85	3%	4%	-7%
Burundi	1,465.76	1,035.71	-29%	-29%	-28%	19.00	13.36	-30%	-30%	-29%
Cabo Verde	392.21	364.92	-7%	-6%	-12%	4.85	4.52	-7%	-6%	-12%
Cambodia	2,052.83	1,311.57	-36%	-36%	-30%	28.22	18.15	-36%	-36%	-31%
Cameroon	1,270.51	1,276.93	1%	1%	-6%	16.69	16.87	1%	2%	-5%
Canada	1,647.64	613.50	-63%	-66%	-14%	22.74	8.23	-64%	-67%	-15%
Central African Republic	2,853.00	3,533.05	24%	23%	33%	39.51	49.23	25%	24%	36%
Chad	821.16	894.74	9%	9%	3%	10.61	11.51	8%	9%	4%
Chile	829.56	636.09	-23%	-22%	-39%	11.15	8.56	-23%	-22%	-40%
China	1,010.46	706.77	-30%	-27%	-63%	13.99	9.62	-31%	-29%	-67%
Colombia	1,246.14	966.92	-22%	-23%	16%	15.81	12.85	-19%	-20%	25%
Comoros	1,156.44	1,088.14	-6%	-6%	1%	14.21	13.57	-5%	-5%	4%
Congo	2,136.57	1,687.27	-21%	-20%	-41%	29.46	23.22	-21%	-20%	-43%
Cook Islands	1,930.57	899.91	-53%	-55%	-18%	26.95	12.63	-53%	-54%	-18%
Costa Rica	978.33	1,036.43	6%	2%	79%	12.83	14.10	10%	6%	90%
Côte d'Ivoire	893.34	744.08	-17%	-17%	-19%	11.49	9.43	-18%	-18%	-22%
Croatia	1,508.08	778.53	-48%	-50%	-23%	18.93	8.99	-52%	-54%	-24%
Cuba	1,686.96	478.05	-72%	-72%	-53%	22.85	6.03	-74%	-74%	-65%
Cyprus	1,765.80	1,140.19	-35%	-51%	290%	24.73	16.01	-35%	-52%	315%
Czechia	1,372.32	629.29	-54%	-55%	-45%	16.86	7.20	-57%	-59%	-46%
Denmark	1,218.11	399.52	-67%	-68%	-57%	16.54	5.22	-68%	-69%	-61%
DPR of Korea	1,655.55	1,585.48	-4%	-4%	-7%	23.45	22.57	-4%	-4%	-5%
DR of the Congo	2,705.50	2,193.56	-19%	-20%	9%	37.14	30.31	-18%	-19%	11%

	DALYs (Disability-Adjusted Life Years)					Deaths				
	Transport injuries			Road injuries	Other transport injuries	Transport injuries			Road injuries	Other transport injuries
	DALYs/100,000 1990	DALYs/100,000 2019	% change, 1990 to 2019	% change, 1990 to 2019	% change, 1990 to 2019	Deaths/100,000 1990	Deaths/100,000 2019	% change, 1990 to 2019	% change, 1990 to 2019	% change, 1990 to 2019
Djibouti	819.59	1,287.07	57%	-6%	974%	10.08	17.06	69%	-5%	1142%
Dominica	1,388.26	1,124.97	-19%	-20%	24%	18.87	15.08	-20%	-21%	28%
Dominican Republic	1,290.21	1,318.64	2%	6%	-63%	17.29	17.92	4%	8%	-68%
Ecuador	1,351.43	1,546.13	14%	13%	62%	18.36	21.76	18%	17%	74%
Egypt	2,206.61	1,276.51	-42%	-42%	-49%	29.61	17.45	-41%	-41%	-49%
El Salvador	1,861.32	902.15	-52%	-52%	-50%	24.80	12.12	-51%	-51%	-49%
Equatorial Guinea	2,291.35	1,399.12	-39%	-39%	-35%	31.29	19.17	-39%	-39%	-36%
Eritrea	1,116.47	988.32	-11%	-12%	-7%	14.41	12.83	-11%	-11%	-6%
Estonia	2,378.29	484.02	-80%	-80%	-71%	31.55	5.79	-82%	-82%	-73%
Eswatini	1,377.80	1,872.56	36%	36%	32%	18.84	26.11	39%	38%	43%
Ethiopia	1,211.81	510.59	-58%	-58%	-56%	15.57	6.39	-59%	-59%	-60%
Fiji	925.54	662.03	-28%	-29%	-15%	12.85	9.04	-30%	-30%	-16%
Finland	1,430.75	422.32	-70%	-71%	-68%	19.42	5.41	-72%	-72%	-69%
France	1,898.90	564.71	-70%	-72%	12%	26.62	7.59	-71%	-73%	12%
Gabon	1,793.77	1,773.30	-1%	-1%	6%	24.30	24.47	1%	0%	10%
Gambia	633.01	641.53	1%	1%	1%	8.04	8.31	3%	3%	3%
Georgia	1,108.73	986.08	-11%	-14%	73%	14.90	13.33	-11%	-14%	90%
Germany	1,702.96	488.02	-71%	-73%	-38%	23.67	6.50	-73%	-74%	-40%
Ghana	1,259.37	1,273.63	1%	2%	-13%	16.62	16.77	1%	2%	-13%
Greece	1,829.73	909.38	-50%	-52%	46%	25.62	12.55	-51%	-52%	50%
Greenland	1,194.65	470.53	-61%	-58%	-65%	16.15	6.16	-62%	-59%	-65%
Grenada	834.01	622.14	-25%	-30%	49%	11.12	8.19	-26%	-31%	62%
Guam	1,198.23	599.26	-50%	-50%	-51%	16.61	8.10	-51%	-51%	-57%
Guatemala	790.85	946.18	20%	13%	289%	10.37	13.03	26%	18%	424%
Guinea	947.00	967.39	2%	3%	-5%	12.17	12.48	3%	3%	-3%
Guinea-Bissau	2,660.16	1,808.56	-32%	-32%	-39%	35.85	24.32	-32%	-32%	-39%
Guyana	1,245.75	1,379.33	11%	6%	86%	16.78	18.98	13%	7%	104%
Haiti	1,052.17	745.38	-29%	-31%	12%	13.81	9.70	-30%	-32%	19%
Honduras	1,068.82	602.23	-44%	-44%	-45%	13.96	7.79	-44%	-44%	-47%
Hungary	1,642.07	501.87	-69%	-71%	-53%	21.23	5.76	-73%	-74%	-55%
Iceland	1,395.68	380.31	-73%	-74%	-66%	19.31	4.96	-74%	-76%	-66%
India	991.47	776.95	-22%	-20%	-34%	12.47	9.65	-23%	-21%	-35%
Indonesia	1,753.66	1,196.76	-32%	-32%	-29%	23.62	16.23	-31%	-32%	-25%
Iran	3,008.68	1,675.86	-44%	-46%	20%	41.22	23.28	-44%	-45%	26%
Iraq	1,201.36	755.76	-37%	-39%	13%	16.04	10.29	-36%	-38%	19%
Ireland	1,239.88	303.55	-76%	-76%	-55%	17.02	3.86	-77%	-78%	-60%
Israel	805.44	393.70	-51%	-51%	-61%	10.82	5.14	-53%	-52%	-66%
Italy	1,621.75	544.67	-66%	-68%	27%	22.58	7.25	-68%	-69%	33%
Jamaica	361.98	333.46	-8%	-9%	15%	4.43	4.17	-6%	-8%	44%
Japan	1,053.35	272.65	-74%	-75%	-39%	14.43	3.51	-76%	-76%	-48%
Jordan	1,256.95	677.14	-46%	-47%	-41%	16.85	9.07	-46%	-47%	-41%
Kazakhstan	1,412.74	797.40	-44%	-43%	-52%	18.91	10.43	-45%	-44%	-57%
Kenya	477.54	526.21	10%	11%	1%	5.84	6.63	14%	14%	4%
Kiribati	1,269.61	1,091.03	-14%	-12%	-35%	17.91	15.41	-14%	-12%	-38%
Kuwait	1,719.50	941.28	-45%	-45%	-52%	23.34	12.73	-45%	-45%	-54%
Kyrgyzstan	1,164.77	686.33	-41%	-42%	-16%	15.56	9.05	-42%	-43%	-17%
Lao PDR	2,209.96	1,182.82	-46%	-47%	-43%	30.01	16.09	-46%	-47%	-43%
Latvia	2,865.85	680.91	-76%	-77%	-68%	38.38	8.60	-78%	-78%	-69%
Lebanon	874.44	625.81	-28%	-29%	0%	11.81	8.52	-28%	-29%	12%
Lesotho	1,456.86	2,148.93	48%	48%	29%	19.99	30.06	50%	51%	41%
Liberia	766.41	620.29	-19%	-20%	-12%	9.72	7.96	-18%	-19%	-12%

	DALYs (Disability-Adjusted Life Years)					Deaths				
	Transport injuries			Road injuries	Other transport injuries	Transport injuries			Road injuries	Other transport injuries
	DALYs/100,000 1990	DALYs/100,000 2019	% change, 1990 to 2019	% change, 1990 to 2019	% change, 1990 to 2019	Deaths/100,000 1990	Deaths/100,000 2019	% change, 1990 to 2019	% change, 1990 to 2019	% change, 1990 to 2019
Libya	2,028.61	1,607.65	-21%	-30%	432%	27.69	22.42	-19%	-29%	523%
Lithuania	2,201.60	652.00	-70%	-71%	-49%	29.02	7.93	-73%	-74%	-51%
Luxembourg	2,398.12	591.67	-75%	-76%	-47%	33.62	7.96	-76%	-77%	-50%
Madagascar	1,341.35	725.85	-46%	-46%	-43%	17.20	9.13	-47%	-47%	-46%
Malawi	964.81	729.05	-24%	-25%	-23%	12.30	9.38	-24%	-24%	-25%
Malaysia	2,071.55	1,707.63	-18%	-17%	-21%	28.51	23.62	-17%	-17%	-21%
Maldives	739.46	342.19	-54%	-55%	-30%	9.81	4.45	-55%	-56%	-32%
Mali	848.73	618.91	-27%	-27%	-31%	10.86	7.79	-28%	-28%	-34%
Malta	712.57	470.34	-34%	-45%	132%	9.71	6.27	-35%	-47%	142%
Marshall Islands	1,465.60	1,017.01	-31%	-31%	-27%	20.92	14.26	-32%	-32%	-29%
Mauritania	2,691.65	1,353.60	-50%	-48%	-72%	36.38	17.86	-51%	-49%	-75%
Mauritius	670.47	762.10	14%	10%	186%	8.71	10.18	17%	13%	458%
Mexico	1,432.07	981.75	-31%	-32%	-8%	19.02	13.22	-31%	-32%	-3%
Micronesia	1,387.88	1,231.52	-11%	-12%	-4%	19.35	17.45	-10%	-10%	-2%
Monaco	1,130.36	615.94	-46%	-47%	-13%	15.49	8.19	-47%	-49%	-14%
Mongolia	796.40	922.44	16%	30%	-64%	10.27	12.15	18%	34%	-67%
Montenegro	889.03	661.95	-26%	-25%	-38%	10.96	7.88	-28%	-27%	-40%
Morocco	2,121.88	1,354.96	-36%	-37%	-12%	28.87	18.54	-36%	-36%	-10%
Mozambique	967.30	1,032.12	7%	7%	2%	11.94	13.20	11%	11%	7%
Myanmar	2,691.88	932.32	-65%	-65%	-68%	37.49	12.82	-66%	-65%	-70%
Namibia	1,240.74	1,230.30	-1%	-1%	-0%	16.90	16.83	-0%	-0%	2%
Nauru	1,919.30	1,889.43	-2%	-2%	9%	27.15	26.80	-1%	-2%	10%
Nepal	715.45	512.37	-28%	-27%	-39%	9.10	6.33	-30%	-29%	-42%
Netherlands	1,107.44	332.20	-70%	-71%	-42%	14.96	4.23	-72%	-72%	-46%
New Zealand	2,824.01	762.39	-73%	-74%	-62%	39.69	10.35	-74%	-75%	-63%
Nicaragua	759.56	739.70	-3%	-4%	32%	9.50	9.82	3%	1%	41%
Niger	919.73	727.36	-21%	-24%	2%	11.86	9.39	-21%	-24%	6%
Nigeria	542.60	411.55	-24%	-24%	-25%	6.83	5.04	-26%	-26%	-29%
Niue	1,190.35	992.63	-17%	-17%	-9%	16.64	13.78	-17%	-18%	-9%
North Macedonia	689.50	595.61	-14%	-20%	107%	7.93	6.95	-12%	-20%	140%
Northern Mariana Islands	1,258.20	688.48	-45%	-43%	-65%	17.62	9.45	-46%	-44%	-69%
Norway	991.62	312.43	-68%	-66%	-79%	13.51	4.02	-70%	-68%	-80%
Oman	4,798.17	3,337.90	-30%	-30%	-21%	66.70	47.13	-29%	-29%	-23%
Pakistan	443.94	590.72	33%	42%	-21%	5.34	7.05	32%	41%	-24%
Palau	1,298.84	1,106.69	-15%	-16%	-7%	18.30	15.65	-14%	-16%	-6%
Palestine	892.32	508.01	-43%	-44%	-22%	11.94	6.75	-43%	-44%	-26%
Panama	1,368.70	726.03	-47%	-48%	37%	18.34	9.60	-48%	-49%	57%
Papua New Guinea	1,667.31	1,466.38	-12%	-13%	-8%	23.16	20.39	-12%	-13%	-7%
Paraguay	792.85	1,481.35	87%	88%	55%	10.47	20.61	97%	97%	75%
Peru	1,029.32	662.37	-36%	-32%	-61%	14.00	9.07	-35%	-31%	-62%
Philippines	1,002.16	635.28	-37%	-36%	-41%	13.60	8.56	-37%	-36%	-43%
Poland	1,581.10	783.32	-50%	-50%	-56%	20.19	9.66	-52%	-52%	-57%
Portugal	2,780.57	521.99	-81%	-82%	-50%	38.94	7.17	-82%	-83%	-51%
Puerto Rico	1,327.63	736.32	-45%	-46%	34%	17.78	9.87	-44%	-46%	55%
Qatar	3,567.36	2,071.86	-42%	-42%	-33%	49.20	29.21	-41%	-41%	-32%
Republic of Korea	1,658.04	377.08	-77%	-77%	-81%	22.75	4.94	-78%	-78%	-85%
Republic of Moldova	1,846.21	739.33	-60%	-61%	-49%	24.40	9.32	-62%	-62%	-51%
Romania	1,102.13	784.73	-29%	-27%	-39%	13.88	9.98	-28%	-26%	-40%
Russian Federation	1,894.23	1,088.68	-43%	-45%	-9%	24.43	14.05	-42%	-45%	-8%
Rwanda	2,187.21	1,145.92	-48%	-48%	-46%	28.56	14.81	-48%	-48%	-47%
Saint Kitts and Nevis	921.12	611.16	-34%	-37%	41%	12.16	7.80	-36%	-40%	56%

	DALYs (Disability-Adjusted Life Years)					Deaths				
	Transport injuries			Road injuries	Other transport injuries	Transport injuries			Road injuries	Other transport injuries
	DALYs/100,000 1990	DALYs/100,000 2019	% change, 1990 to 2019	% change, 1990 to 2019	% change, 1990 to 2019	Deaths/100,000 1990	Deaths/100,000 2019	% change, 1990 to 2019	% change, 1990 to 2019	% change, 1990 to 2019
Saint Lucia	1,199.52	877.09	-27%	-31%	78%	16.15	11.81	-27%	-31%	97%
Samoa	1,109.34	938.02	-15%	-16%	-6%	15.38	13.10	-15%	-15%	-4%
San Marino	1,188.84	664.08	-44%	-46%	50%	16.09	8.89	-45%	-46%	64%
Sao Tome and Principe	470.90	598.78	27%	29%	15%	5.62	7.70	37%	39%	25%
Saudi Arabia	2,157.69	1,923.01	-11%	-11%	5%	29.74	27.30	-8%	-9%	11%
Senegal	796.63	686.17	-14%	-14%	-17%	10.17	8.78	-14%	-13%	-18%
Serbia	1,378.38	520.89	-62%	-62%	-66%	17.15	6.02	-65%	-64%	-69%
Seychelles	717.91	987.87	38%	33%	185%	9.63	13.56	41%	36%	351%
Sierra Leone	1,004.98	986.76	-2%	-1%	-8%	13.05	12.88	-1%	-1%	-7%
Singapore	714.49	262.64	-63%	-64%	-38%	9.73	3.26	-66%	-67%	-46%
Slovakia	1,414.19	670.75	-53%	-54%	-42%	17.97	8.04	-55%	-57%	-42%
Slovenia	1,947.02	687.38	-65%	-66%	-20%	25.00	7.94	-68%	-70%	-21%
Solomon Islands	1,583.45	1,458.35	-8%	-8%	-8%	22.51	20.80	-8%	-8%	-8%
Somalia	1,000.70	1,149.17	15%	14%	20%	12.97	15.08	16%	16%	23%
South Africa	1,864.23	1,106.78	-41%	-41%	-37%	25.99	15.68	-40%	-40%	-42%
South Sudan	663.69	585.06	-12%	-12%	-9%	7.83	6.95	-11%	-11%	-11%
Spain	2,042.00	393.09	-81%	-83%	20%	28.46	5.12	-82%	-84%	22%
Sri Lanka	908.12	661.60	-27%	-26%	-39%	12.50	9.14	-27%	-26%	-43%
St Vincent & the Grenadines	691.01	523.56	-24%	-27%	52%	9.02	6.81	-25%	-28%	71%
Sudan	1,188.38	812.84	-32%	-29%	-57%	16.16	11.03	-32%	-29%	-61%
Suriname	1,423.16	1,126.19	-21%	-23%	48%	19.19	15.07	-21%	-23%	59%
Sweden	1,069.61	304.72	-72%	-72%	-63%	14.55	3.89	-73%	-74%	-66%
Switzerland	1,615.94	341.74	-79%	-80%	-72%	22.27	4.31	-81%	-81%	-74%
Syrian Arab Republic	1,051.10	705.99	-33%	-34%	14%	14.04	9.44	-33%	-34%	41%
Taiwan (Province of China)	2,326.22	956.54	-59%	-59%	-42%	32.75	13.35	-59%	-59%	-47%
Tajikistan	631.81	408.79	-35%	-38%	20%	8.05	5.23	-35%	-38%	26%
Thailand	3,187.37	2,170.18	-32%	-32%	-36%	44.37	30.46	-31%	-31%	-36%
Timor-Leste	977.58	910.63	-7%	-8%	16%	13.04	12.33	-5%	-7%	21%
Togo	1,598.97	1,388.73	-13%	-13%	-9%	21.13	18.45	-13%	-13%	-8%
Tokelau	1,035.99	776.96	-25%	-25%	-21%	14.38	10.83	-25%	-25%	-20%
Tonga	523.73	608.44	16%	17%	10%	7.06	8.35	18%	18%	18%
Trinidad and Tobago	1,032.41	787.50	-24%	-25%	18%	13.71	10.56	-23%	-24%	31%
Tunisia	1,624.47	1,066.61	-34%	-34%	-35%	21.75	14.56	-33%	-33%	-37%
Turkey	842.44	525.34	-38%	-37%	-42%	11.08	6.93	-37%	-37%	-43%
Turkmenistan	922.36	364.62	-60%	-61%	-29%	12.19	4.59	-62%	-63%	-37%
Tuvalu	1,968.73	1,308.09	-34%	-34%	-26%	27.75	18.57	-33%	-34%	-26%
Uganda	792.18	870.64	10%	12%	-5%	9.97	11.15	12%	14%	-4%
Ukraine	1,709.03	1,420.99	-17%	-20%	344%	22.68	18.93	-17%	-20%	597%
United Arab Emirates	2,460.20	1,327.02	-46%	-47%	-4%	33.71	18.30	-46%	-46%	-1%
United Kingdom	1,116.31	346.41	-69%	-70%	-47%	15.29	4.56	-70%	-71%	-51%
United Republic of Tanzania	774.62	599.80	-23%	-24%	-2%	9.65	7.37	-24%	-25%	-2%
United States of America	1,914.94	909.75	-52%	-54%	-32%	26.26	12.39	-53%	-54%	-32%
United States Virgin Islands	1,366.64	542.84	-60%	-62%	-34%	18.06	6.97	-61%	-63%	-35%
Uruguay	993.96	974.19	-2%	-4%	46%	13.34	13.33	-0%	-3%	53%
Uzbekistan	783.51	682.57	-13%	-14%	10%	10.29	8.86	-14%	-15%	17%
Vanuatu	1,191.58	1,412.60	19%	18%	27%	16.64	19.93	20%	19%	33%
Venezuela	1,865.46	1,507.69	-19%	-23%	63%	24.78	20.53	-17%	-21%	73%
Viet Nam	1,439.95	1,507.97	5%	4%	17%	19.73	20.73	5%	4%	21%
Yemen	2,008.04	1,777.68	-11%	-12%	27%	26.89	24.36	-9%	-10%	36%
Zambia	949.68	861.62	-9%	-8%	-21%	12.26	11.37	-7%	-5%	-21%
Zimbabwe	974.63	909.16	-7%	-10%	19%	13.34	12.42	-7%	-10%	23%

Figure S7: Percentage change in unintentional injury DALYs per 100,000 population from 1990 to 2019

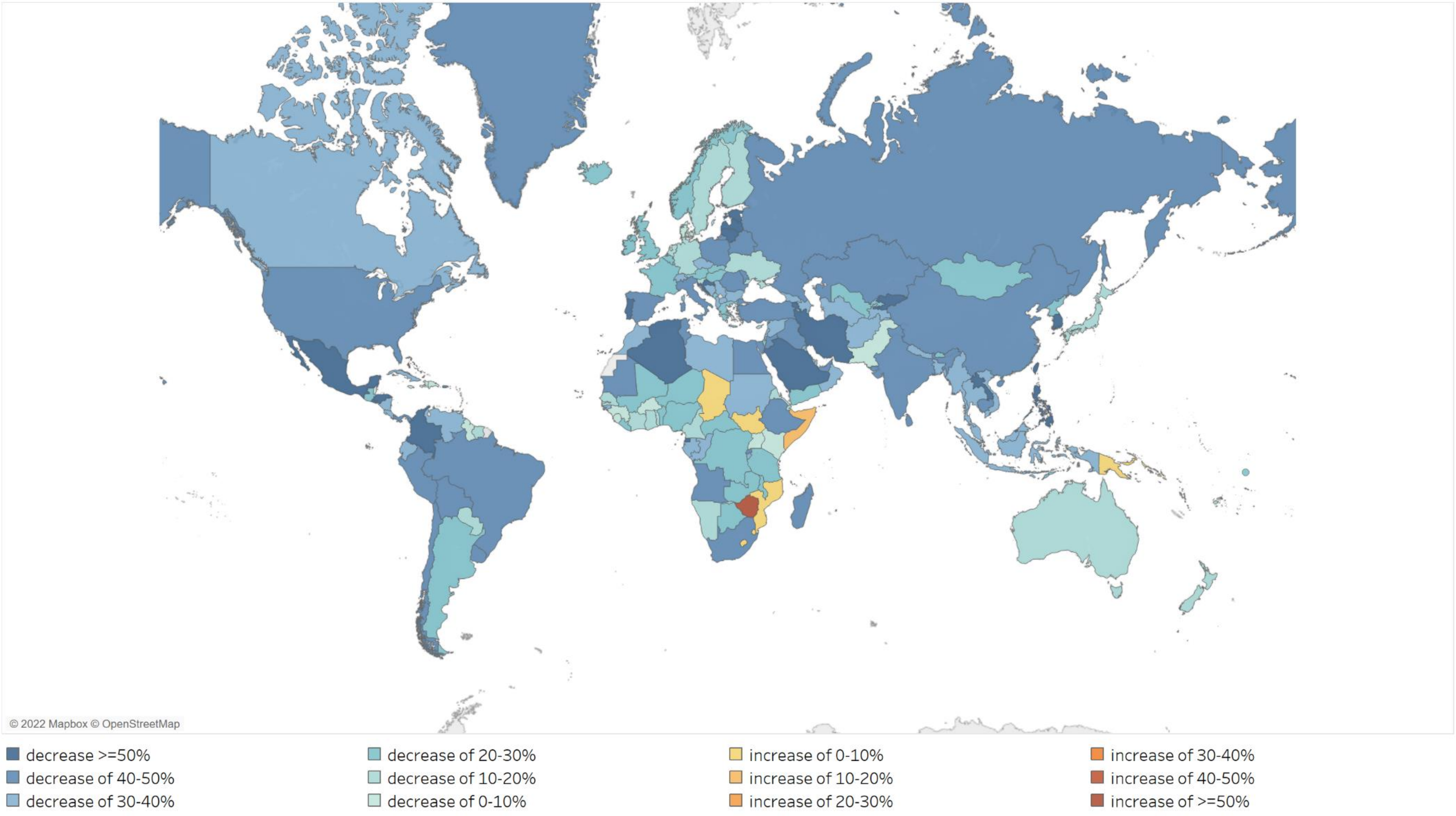

Figure S8: Heatmap of unintentional injury mechanism death rates and percentage change 1990-2019 by country, adolescents 10-24 years

	Unintentional injuries Deaths/100,000 1990	Unintentional injuries Deaths/100,000 2019	Unintentional injuries % change, 1990 to 2019	Percentage change in deaths/ 100,000, 1990 to 2019, for mechanisms of Unintentional injury: Adverse effects of medical treatment	Animal contact	Drowning	Environmental heat and cold exposure	Exposure to mechanical forces	Falls	Fire, heat, and hot substances	Foreign body	Poisonings	Other unintentional injuries
Afghanistan	26.42	15.76	-40%	-55%	-12%	-47%	-75%	-23%	-27%	-40%	-25%	-44%	-49%
Albania	13.41	8.08	-40%	21%	-68%	-67%	-9%	-56%	11%	-44%	-9%	-2%	-60%
Algeria	19.35	7.80	-60%	-68%	-65%	-70%	-56%	-46%	-44%	-67%	-49%	-59%	-59%
American Samoa	17.66	14.62	-17%	-17%	11%	-15%	-35%	-34%	4%	-13%	1%	-40%	-36%
Andorra	2.76	1.68	-39%	-40%	-4%	-47%	-34%	-41%	-34%	-47%	-37%	-45%	-49%
Angola	11.92	6.44	-46%	-53%	-57%	-49%	-60%	-48%	-22%	-41%	-26%	-50%	-58%
Antigua and Barbuda	15.98	13.24	-17%	-29%	319%	-26%	-18%	7%	19%	-47%	-32%	-81%	-20%
Argentina	12.77	7.95	-38%	-38%	177%	-59%	14%	-19%	-37%	-35%	-39%	-27%	-22%
Armenia	20.43	6.21	-70%	0%	-25%	-80%	-79%	-54%	-73%	-83%	-28%	-78%	-55%
Australia	4.56	2.00	-56%	-16%	-28%	-55%	-53%	-68%	-22%	-70%	-48%	-60%	-80%
Austria	5.51	2.52	-54%	37%	-23%	-65%	-33%	-71%	-54%	-60%	-18%	-29%	-67%
Azerbaijan	15.65	9.66	-38%	-19%	-11%	-54%	-40%	-26%	-37%	-24%	3%	-44%	-6%
Bahamas	13.15	21.46	63%	-19%	232%	-26%	-25%	-6%	6%	-37%	-30%	-74%	-22%
Bahrain	7.51	4.71	-37%	-60%	-49%	-57%	-36%	-5%	-27%	-30%	-25%	-57%	-24%
Bangladesh	13.67	8.25	-40%	-56%	-43%	-44%	-3%	-55%	302%	-44%	90%	-6%	-63%
Barbados	11.38	6.69	-41%	-36%	7%	-44%	-48%	-9%	-14%	-54%	-51%	-85%	-28%
Belarus	21.08	9.92	-53%	-18%	-34%	-63%	-55%	-48%	-27%	-34%	-18%	-59%	-56%
Belgium	4.73	1.96	-59%	-65%	9%	-62%	-38%	-57%	-48%	-59%	21%	-79%	-68%
Belize	14.33	13.88	-3%	-12%	468%	-5%	13%	18%	55%	-33%	-20%	-77%	5%
Benin	8.97	6.75	-25%	-30%	-1%	-36%	-13%	-29%	-17%	-17%	-10%	-26%	-31%
Bermuda	9.41	4.45	-53%	-51%	78%	-53%	-50%	-40%	-29%	-55%	-56%	-90%	-51%
Bhutan	13.85	10.46	-24%	-41%	-31%	-34%	-42%	-31%	47%	-14%	-19%	-26%	-12%
Bolivia	19.63	10.27	-48%	-58%	23%	-54%	-50%	-45%	-27%	-57%	-49%	-62%	-57%
Bosnia and Herzegovina	13.16	4.99	-62%	-80%	7%	-19%	627%	-88%	0%	-24%	8%	-94%	-4%
Botswana	16.37	12.68	-23%	-26%	-23%	-20%	-43%	-35%	-11%	-16%	5%	-21%	-33%
Brazil	14.98	6.97	-53%	-32%	-44%	-59%	-52%	-12%	-53%	-60%	-15%	-50%	-63%
Brunei Darussalam	17.95	6.50	-64%	-44%	-54%	-69%	-63%	-48%	-48%	-61%	-45%	-75%	-74%
Bulgaria	16.71	7.66	-54%	22%	-21%	-54%	-13%	-22%	-40%	-48%	-49%	-70%	-78%
Burkina Faso	7.24	6.71	-7%	-14%	-13%	-10%	-12%	-6%	7%	-2%	3%	-9%	-14%
Burundi	14.79	9.61	-35%	-33%	-37%	-36%	-35%	-25%	-34%	-34%	-32%	-39%	-45%
Cabo Verde	6.48	9.13	41%	-34%	1,216%	89%	257%	-24%	-5%	13%	276%	52%	-30%
Cambodia	24.82	13.20	-47%	-58%	-50%	-55%	-40%	-41%	-17%	-59%	-35%	-51%	-57%
Cameroon	7.45	6.83	-8%	-23%	2%	-19%	-5%	-16%	1%	-1%	12%	-11%	-20%
Canada	5.89	2.60	-56%	-47%	-30%	-52%	-52%	-65%	-30%	-69%	-47%	-51%	-77%
Central African Republic	15.55	11.93	-23%	-30%	-14%	-23%	-29%	-24%	-23%	-22%	-12%	-22%	-32%
Chad	9.19	9.21	0%	-15%	40%	-8%	20%	-11%	11%	8%	12%	0%	-9%
Chile	11.21	4.41	-61%	-58%	-47%	-45%	-71%	-45%	-43%	-73%	-89%	-65%	-60%
China	16.86	8.70	-48%	-78%	-85%	-58%	-84%	-39%	-23%	-67%	-18%	-18%	-15%
Colombia	15.17	5.52	-64%	-41%	-71%	-58%	-70%	-65%	-57%	-77%	-39%	-82%	-76%
Comoros	7.86	7.16	-9%	-28%	-31%	-27%	-28%	-14%	-11%	-18%	6%	-26%	-33%
Congo	9.59	5.14	-46%	-52%	-34%	-49%	-62%	-49%	-36%	-43%	-35%	-47%	-54%
Cook Islands	8.96	3.79	-58%	-38%	-52%	-60%	-45%	-42%	-24%	-43%	-36%	-72%	-46%
Costa Rica	9.83	5.99	-39%	-46%	-8%	-50%	64%	-34%	-8%	-35%	-57%	151%	-14%
Côte d'Ivoire	8.85	6.80	-23%	-31%	-20%	-30%	-10%	-25%	-13%	-22%	-10%	-24%	-28%
Croatia	13.26	2.80	-79%	15%	-19%	-56%	-61%	-55%	-27%	-62%	-35%	-71%	-98%
Cuba	12.20	5.40	-56%	-67%	-40%	-54%	-29%	-60%	-46%	-79%	-68%	-77%	-46%
Cyprus	4.37	2.60	-41%	-38%	-16%	-48%	-49%	-56%	-29%	-42%	-2%	-29%	-45%
Czechia	9.81	3.20	-67%	78%	-38%	-68%	-14%	-63%	-58%	-67%	-55%	-78%	-83%
Denmark	2.63	1.19	-55%	39%	67%	-51%	-4%	-61%	-54%	-50%	4%	-19%	-91%
DPR of Korea	19.01	14.03	-26%	-48%	-42%	-38%	-34%	-5%	-5%	-40%	-3%	-14%	-1%
DR of the Congo	8.55	6.36	-26%	-35%	-17%	-33%	-36%	-16%	-14%	-26%	-7%	-24%	-36%

	Unintentional injuries Deaths/100,000 1990	Unintentional injuries Deaths/100,000 2019	Unintentional injuries % change, 1990 to 2019	**Percentage change in deaths/ 100,000, 1990 to 2019, for mechanisms of Unintentional injury**									
				Adverse effects of medical treatment	Animal contact	Drowning	Environmental heat and cold exposure	Exposure to mechanical forces	Falls	Fire, heat, and hot substances	Foreign body	Poisonings	Other unintentional injuries
Djibouti	7.78	6.80	-13%	-30.8%	12.9%	-15.5%	-32.6%	-16.3%	3.9%	-11.4%	12.7%	-13.3%	-22.8%
Dominica	16.97	12.78	-25%	-21.1%	252.4%	-16.6%	-16.4%	2.6%	5.8%	-25.5%	-27.4%	-76.9%	-3.7%
Dominican Republic	10.16	9.57	-6%	-12.3%	2.3%	-6.6%	-40.2%	9.8%	-0.2%	-24.0%	3.9%	-23.7%	-9.6%
Ecuador	17.03	10.50	-38%	-52.6%	653.8%	-41.9%	17.3%	-46.2%	-19.2%	-37.7%	-33.0%	-51.3%	-49.2%
Egypt	10.42	5.15	-51%	-63.8%	-51.2%	-54.8%	-57.8%	-43.3%	-32.3%	-52.1%	-35.5%	-59.0%	-53.6%
El Salvador	15.90	6.62	-58%	-75.0%	108.9%	-12.9%	-7.4%	-72.4%	-50.6%	-63.6%	-87.0%	-86.5%	-82.9%
Equatorial Guinea	13.28	3.95	-70%	-78.9%	-76.8%	-75.5%	-81.0%	-72.3%	-49.0%	-64.6%	-46.0%	-72.9%	-73.8%
Eritrea	12.08	9.65	-20%	-30.0%	-13.5%	-25.7%	-35.7%	-17.9%	-6.0%	-18.5%	5.4%	-21.0%	-27.6%
Estonia	26.17	5.19	-80%	-66.4%	-20.0%	-85.6%	-78.0%	-68.9%	-70.6%	-78.9%	-64.4%	-91.2%	-89.6%
Eswatini	15.03	16.02	7%	-8.4%	8.5%	3.8%	8.4%	1.9%	33.8%	12.3%	34.6%	-7.2%	-4.2%
Ethiopia	16.50	7.27	-56%	-64.3%	-54.7%	-67.1%	-57.4%	-43.2%	-49.7%	-52.6%	-38.4%	-63.2%	-57.0%
Fiji	18.57	14.67	-21%	-31.9%	-32.0%	-20.7%	-44.9%	-29.4%	-3.5%	-20.9%	2.7%	-45.3%	-39.1%
Finland	4.58	2.07	-55%	-33.3%	-58.2%	-57.3%	24.5%	-54.1%	-54.7%	-59.5%	-34.1%	-66.3%	-87.9%
France	6.08	2.58	-58%	-72.8%	-46.0%	-49.5%	253.5%	-65.2%	-56.9%	-67.8%	-61.6%	-61.8%	-71.3%
Gabon	8.61	5.15	-40%	-51.2%	-31.5%	-48.5%	-53.5%	-39.5%	-33.6%	-33.7%	-22.6%	-40.5%	-46.1%
Gambia	6.82	6.08	-11%	-22.7%	-20.7%	-20.3%	5.0%	-11.0%	2.4%	0.8%	17.5%	-12.2%	-20.5%
Georgia	20.72	11.00	-47%	190.8%	14.2%	-78.1%	-21.7%	20.1%	-15.5%	-64.0%	49.4%	-74.1%	18.5%
Germany	3.86	1.62	-58%	1.6%	-10.9%	-54.7%	-52.7%	-57.9%	-58.8%	-69.3%	-49.2%	-54.6%	-73.0%
Ghana	7.25	5.77	-20%	-24.0%	-26.5%	-26.2%	-30.4%	-17.7%	-0.8%	-16.4%	5.7%	-22.4%	-34.6%
Greece	5.71	2.66	-53%	-29.8%	14.2%	-37.6%	-44.3%	-36.4%	-60.0%	-62.7%	-72.4%	-59.6%	-75.6%
Greenland	17.42	9.71	-44%	-45.8%	-54.3%	-48.6%	-39.7%	-42.7%	-18.7%	-58.2%	-30.7%	-60.9%	-49.5%
Grenada	17.33	12.84	-26%	-39.8%	336.0%	-33.5%	-7.5%	7.0%	26.7%	-47.3%	-21.6%	-87.4%	-17.3%
Guam	10.53	5.88	-44%	-2.5%	23.2%	-49.1%	-65.4%	-65.4%	-19.5%	-18.1%	-15.9%	-69.1%	-50.1%
Guatemala	24.20	17.66	-27%	-74.7%	79.1%	-48.0%	312.6%	153.4%	-30.6%	-64.1%	-58.1%	12.4%	-26.1%
Guinea	9.68	9.00	-7%	-18.8%	20.7%	-17.6%	3.8%	-5.3%	1.2%	0.7%	7.6%	-7.1%	-17.9%
Guinea-Bissau	14.94	9.79	-34%	-39.6%	-20.8%	-44.3%	-21.7%	-33.5%	-28.1%	-27.6%	-22.3%	-36.0%	-39.9%
Guyana	20.99	18.97	-10%	-11.3%	333.0%	12.3%	-28.5%	17.7%	0.8%	-40.0%	-29.8%	-76.7%	-41.7%
Haiti	33.05	19.86	-40%	-39.0%	6.1%	-44.0%	-42.5%	-24.2%	-27.2%	-49.6%	-47.2%	-50.0%	-43.9%
Honduras	20.71	7.54	-64%	-63.2%	-72.5%	-67.8%	-65.0%	-59.8%	-49.5%	-67.2%	-60.5%	-68.9%	-64.8%
Hungary	7.82	2.74	-65%	-45.6%	-33.8%	-66.9%	-40.5%	-58.0%	-67.7%	-71.6%	-48.8%	-73.6%	-68.7%
Iceland	4.78	1.77	-63%	-40.1%	-31.9%	-69.1%	-65.8%	-73.0%	-47.5%	-71.8%	-43.3%	-64.3%	-72.4%
India	21.24	11.54	-46%	-47.7%	-48.8%	-51.1%	-60.1%	-47.6%	-25.2%	-38.4%	-36.2%	-53.9%	-50.0%
Indonesia	10.48	5.51	-47%	-53.5%	-40.8%	-52.7%	8.7%	-50.0%	-32.0%	-43.5%	-62.0%	-14.7%	-62.6%
Iran	70.31	9.50	-86%	-70.0%	-53.9%	-65.3%	-21.7%	-18.3%	-30.0%	-68.8%	-11.9%	-55.1%	-39.1%
Iraq	39.10	19.17	-51%	-52.2%	-72.3%	-54.7%	-86.4%	-46.3%	-49.0%	-51.3%	-35.0%	-48.8%	-56.5%
Ireland	5.08	1.97	-61%	-18.4%	-20.5%	-63.9%	-60.2%	-71.3%	-40.8%	-73.0%	-57.3%	-52.5%	-74.9%
Israel	4.87	1.94	-60%	-74.9%	-12.4%	-64.5%	-36.3%	-73.3%	-35.7%	-48.4%	-45.3%	-56.1%	-59.3%
Italy	4.95	1.82	-63%	-30.3%	-19.1%	-54.3%	-53.8%	-70.5%	-57.9%	-63.0%	-54.9%	-84.0%	-76.8%
Jamaica	3.53	3.56	1%	133.6%	45.1%	-46.1%	34.6%	84.7%	49.7%	-20.6%	-74.2%	-33.7%	-41.5%
Japan	2.99	1.71	-43%	35.8%	-11.5%	-40.8%	-2.4%	-68.3%	-35.7%	-61.0%	-8.4%	-58.1%	-61.3%
Jordan	12.62	5.42	-57%	-65.9%	-47.5%	-58.1%	-59.6%	-65.9%	-41.9%	-57.2%	-38.2%	-65.9%	-59.5%
Kazakhstan	23.20	10.77	-54%	-40.5%	-39.4%	-60.4%	-64.3%	-46.4%	-43.7%	-66.4%	-27.6%	-48.4%	-38.0%
Kenya	6.34	6.01	-5%	-22.0%	5.8%	-9.3%	-7.0%	0.0%	10.1%	-5.3%	19.1%	5.9%	-13.8%
Kiribati	15.21	11.72	-23%	-30.1%	-32.4%	-23.9%	27.6%	-26.3%	-13.1%	-6.6%	-16.8%	-23.0%	-29.8%
Kuwait	6.75	3.01	-55%	-55.7%	-57.6%	-71.9%	-40.2%	-63.5%	-27.5%	-45.3%	-67.9%	-56.1%	-69.2%
Kyrgyzstan	19.64	6.28	-68%	-21.1%	-43.6%	-74.7%	-51.7%	-36.7%	-65.4%	-73.6%	-40.6%	-66.2%	-61.3%
Lao PDR	27.52	12.60	-54%	-61.4%	-54.3%	-61.8%	-61.1%	-48.3%	-32.6%	-62.5%	-48.1%	-67.5%	-42.8%
Latvia	26.62	7.59	-71%	-9.8%	-14.0%	-75.3%	-66.4%	-64.9%	-67.8%	-69.6%	-51.5%	-83.9%	-61.5%
Lebanon	11.28	6.17	-45%	-57.0%	-47.6%	-50.0%	-45.4%	-31.7%	-25.1%	-62.3%	-15.8%	-38.5%	-41.6%
Lesotho	17.34	18.02	4%	-0.8%	9.1%	6.1%	15.6%	-0.3%	36.9%	24.0%	46.5%	4.0%	2.4%
Liberia	8.90	6.50	-27%	-31.5%	-11.3%	-42.5%	-18.1%	-23.7%	-27.1%	17.2%	-17.3%	-28.8%	-39.1%

	Unintentional injuries Deaths/100,000 1990	Unintentional injuries Deaths/100,000 2019	Unintentional injuries % change, 1990 to 2019	Percentage change in deaths/ 100,000, 1990 to 2019, for mechanisms of Unitentional injury									
				Adverse effects of medical treatment	Animal contact	Drowning	Environmental heat and cold exposure	Exposure to mechanical forces	Falls	Fire, heat, and hot substances	Foreign body	Poisonings	Other unintentional injuries
Libya	11.46	7.00	-39%	-52.1%	-44.0%	-50.9%	-40.0%	-27.0%	-26.0%	-46.2%	-12.2%	-29.8%	-39.3%
Lithuania	22.11	7.30	-67%	-3.5%	-52.6%	-74.6%	-43.7%	-59.2%	-57.8%	-75.7%	-24.3%	-52.5%	-72.4%
Luxembourg	7.33	2.56	-65%	-52.2%	-45.7%	-64.1%	-64.1%	-69.6%	-54.6%	-69.0%	-45.0%	-79.6%	-80.4%
Madagascar	13.74	6.69	-51%	-54.8%	-50.8%	-56.5%	-50.4%	-42.1%	-46.3%	-51.6%	-41.1%	-53.6%	-57.3%
Malawi	9.96	6.92	-31%	-43.9%	-39.9%	-37.8%	-36.5%	-26.1%	-16.5%	-31.9%	-15.8%	-36.1%	-43.0%
Malaysia	10.76	6.87	-36%	-50.9%	-41.3%	-35.4%	-39.9%	-39.0%	-23.5%	-39.4%	-19.5%	-45.6%	-45.6%
Maldives	21.47	7.14	-67%	-74.9%	-78.8%	-71.5%	-56.9%	-66.1%	-36.2%	-68.7%	-66.6%	-67.8%	-66.8%
Mali	10.35	7.49	-28%	-38.9%	-32.0%	-35.6%	-39.0%	-25.7%	-9.8%	-26.0%	29.8%	-33.8%	-39.2%
Malta	4.24	2.98	-30%	-27.3%	3.1%	-24.9%	-35.3%	-32.9%	-7.8%	-39.3%	16.6%	-55.3%	-56.8%
Marshall Islands	22.96	18.07	-21%	-33.0%	-47.9%	-17.2%	-50.7%	-38.9%	-12.7%	-17.4%	19.7%	-40.6%	-28.0%
Mauritania	7.67	3.93	-49%	-54.0%	-36.7%	-54.8%	-51.8%	-49.5%	-36.8%	-42.3%	-40.5%	-51.5%	-57.6%
Mauritius	11.88	6.69	-44%	53.2%	-1.5%	-29.9%	-35.8%	-47.5%	40.2%	-79.2%	34.5%	26.3%	-52.7%
Mexico	17.18	6.82	-60%	-37.5%	-69.9%	-63.4%	-74.3%	-68.1%	-55.4%	-59.6%	-31.3%	-64.1%	-62.6%
Micronesia	20.28	19.49	-4%	-27.6%	-14.9%	0.8%	-27.2%	-26.3%	16.4%	-10.7%	31.2%	-39.3%	-3.8%
Monaco	5.63	3.58	-36%	-33.4%	-25.6%	-29.0%	-33.3%	-42.2%	-22.8%	-40.4%	16.4%	-64.0%	-55.4%
Mongolia	22.38	15.89	-29%	-34.3%	73.7%	-19.1%	-44.7%	-36.6%	25.5%	28.0%	84.3%	-73.7%	15.2%
Montenegro	8.00	4.50	-44%	-32.5%	-7.9%	-43.2%	-47.1%	-36.5%	-39.4%	-39.3%	-30.0%	-48.3%	-58.8%
Morocco	15.54	8.74	-44%	-54.5%	-38.2%	-46.6%	-59.7%	-41.5%	-25.1%	-52.5%	-22.0%	-42.3%	-49.5%
Mozambique	9.34	10.49	12%	-30.1%	-17.2%	-22.1%	-18.6%	-3.6%	10.6%	-7.5%	24.4%	-13.0%	-19.1%
Myanmar	30.26	15.69	-48%	-61.9%	-58.1%	-56.7%	-37.7%	-47.9%	-25.1%	-53.4%	-39.4%	-60.2%	-43.2%
Namibia	12.05	10.03	-17%	-27.3%	-25.2%	-17.1%	-35.8%	-18.9%	14.4%	-12.7%	4.0%	-26.6%	-24.6%
Nauru	20.28	20.09	-1%	-27.0%	-7.5%	2.0%	-28.1%	-22.0%	13.6%	-11.5%	36.2%	-26.7%	-0.1%
Nepal	19.64	12.26	-38%	-50.2%	-49.5%	-44.9%	-56.1%	-37.5%	-19.1%	-38.1%	-30.0%	-49.4%	-34.8%
Netherlands	2.22	1.00	-55%	-50.1%	-24.1%	-55.0%	-52.9%	-61.5%	-27.0%	-66.3%	-40.5%	-64.5%	-81.2%
New Zealand	6.92	3.37	-51%	-44.3%	-41.6%	-51.5%	-50.3%	-64.8%	-35.3%	-69.2%	-11.5%	-29.4%	-80.2%
Nicaragua	10.27	6.67	-35%	-53.5%	-26.0%	-27.5%	28.1%	-49.5%	-5.5%	-52.7%	-42.8%	-32.3%	-45.2%
Niger	8.91	6.79	-24%	-28.6%	-22.5%	-30.3%	-34.7%	-16.9%	-8.7%	-19.9%	-22.4%	-25.0%	-30.0%
Nigeria	6.08	4.01	-34%	-39.2%	-33.3%	-36.4%	-42.1%	-40.2%	-5.5%	-27.3%	-14.1%	-40.4%	-46.5%
Niue	14.88	12.71	-15%	-37.9%	-27.2%	-12.0%	-47.4%	-35.9%	-4.3%	-21.5%	31.3%	-36.5%	-16.9%
North Macedonia	10.30	4.04	-61%	-39.8%	-9.7%	-43.8%	-13.9%	-11.4%	41.3%	-38.2%	6.9%	-26.4%	-80.3%
Northern Mariana Islands	15.25	12.06	-21%	-63.0%	-7.1%	-8.1%	-64.4%	-56.2%	-5.8%	-2.1%	50.3%	-59.0%	-46.2%
Norway	5.20	2.13	-59%	-51.9%	22.3%	-66.4%	-46.6%	-69.0%	-53.2%	-66.4%	-49.8%	50.8%	-88.2%
Oman	14.59	8.02	-45%	-58.4%	-30.4%	-48.8%	-61.3%	-55.0%	-22.5%	-37.7%	-21.1%	-40.1%	-36.4%
Pakistan	7.77	7.35	-5%	-1.8%	17.5%	-51.7%	3.5%	-10.3%	33.1%	25.6%	81.3%	1.9%	-6.8%
Palau	31.96	26.35	-18%	-25.2%	7.8%	-20.2%	-19.4%	-19.3%	-3.6%	-22.6%	0.2%	-27.0%	-21.0%
Palestine	10.60	5.30	-50%	-59.8%	-31.5%	-60.7%	-50.4%	-53.0%	-23.6%	-59.6%	8.6%	-62.2%	-61.8%
Panama	14.13	6.44	-54%	-75.0%	-33.6%	-59.6%	-18.7%	-45.4%	-30.7%	-28.6%	-69.2%	-54.5%	-42.6%
Papua New Guinea	11.04	11.11	1%	-24.5%	-12.7%	-5.8%	-4.8%	2.2%	19.5%	7.7%	-9.8%	0.7%	6.6%
Paraguay	9.56	8.53	-11%	-2.9%	233.0%	-12.2%	4.3%	-33.7%	14.5%	-9.7%	-37.2%	-51.8%	-3.2%
Peru	20.87	9.37	-55%	-65.9%	-4.0%	-59.2%	-49.4%	-38.0%	-27.8%	-63.3%	-50.4%	-61.0%	-62.4%
Philippines	18.30	7.86	-57%	-33.3%	-51.9%	-46.2%	-36.9%	-34.5%	-25.7%	-35.8%	-17.3%	-44.4%	-42.2%
Poland	12.66	4.30	-66%	8.3%	-60.9%	-71.8%	-44.5%	-64.0%	-40.3%	-54.5%	-39.4%	-82.8%	-82.2%
Portugal	9.75	2.43	-75%	-29.1%	-43.8%	-77.9%	-68.9%	-84.3%	-71.0%	-75.2%	-37.9%	-79.7%	-86.1%
Puerto Rico	6.91	3.02	-56%	-43.8%	22.9%	-74.3%	-35.8%	37.3%	-9.6%	-51.9%	-31.6%	-94.3%	-34.4%
Qatar	13.84	8.34	-40%	-64.1%	-55.6%	-55.7%	-68.3%	-36.5%	-15.4%	-53.4%	-38.1%	-21.4%	-37.4%
Republic of Korea	16.35	2.16	-87%	-62.2%	-52.5%	-89.7%	-77.4%	-77.5%	-77.2%	-81.6%	-85.7%	-94.6%	-94.0%
Republic of Moldova	21.97	8.67	-61%	-66.1%	-30.9%	-66.1%	-1.0%	-68.0%	-58.7%	-68.6%	-3.4%	-33.4%	-71.1%
Romania	17.79	6.99	-61%	-37.2%	-2.5%	-59.7%	-50.1%	-57.0%	-67.7%	-61.5%	-23.7%	-72.3%	-59.4%
Russian Federation	23.33	11.81	-49%	27.9%	-50.5%	-54.9%	-79.1%	-70.0%	-16.0%	-44.6%	14.3%	-17.6%	-56.6%
Rwanda	12.11	5.88	-51%	-56.3%	-53.8%	-58.2%	-59.4%	-36.2%	-39.6%	-48.5%	-44.1%	-55.8%	-59.0%
Saint Kitts and Nevis	13.89	10.29	-26%	-45.5%	201.2%	-31.4%	-15.9%	5.9%	10.1%	-54.7%	-46.3%	-84.4%	-25.1%

	Unintentional injuries Deaths/100,000 1990	Unintentional injuries Deaths/100,000 2019	Unintentional injuries % change, 1990 to 2019	**Percentage change in deaths/ 100,000, 1990 to 2019, for mechanisms of Unitentional injury**									
				Adverse effects of medical treatment	Animal contact	Drowning	Environmental heat and cold exposure	Exposure to mechanical forces	Falls	Fire, heat, and hot substances	Foreign body	Poisonings	Other unintentional injuries
Saint Lucia	13.04	10.19	-22%	-23%	226%	-25%	-22%	14%	11%	-47%	-36%	-80%	-24%
Samoa	18.47	11.34	-39%	-26%	-35%	-16%	-35%	-34%	-5%	-20%	15%	-45%	-17%
San Marino	2.55	2.00	-22%	-28%	-10%	-7%	-33%	-32%	-7%	-11%	4%	-32%	-42%
Sao Tome and Principe	6.60	5.44	-18%	-21%	-28%	-33%	-3%	-8%	-13%	4%	-1%	-20%	-20%
Saudi Arabia	33.59	13.71	-59%	-62%	-52%	-43%	-51%	-83%	-25%	-30%	-14%	-39%	-63%
Senegal	8.13	6.26	-23%	-30%	-24%	-31%	-28%	-17%	-7%	-20%	-11%	-23%	-33%
Serbia	8.79	2.87	-67%	-68%	-44%	-71%	-68%	-55%	-56%	-63%	-50%	-76%	-76%
Seychelles	16.89	12.60	-25%	-7%	-1%	-16%	34%	-61%	-22%	-48%	-3%	-30%	-45%
Sierra Leone	8.34	7.55	-9%	-13%	20%	-22%	-1%	-12%	-8%	-1%	6%	-8%	-20%
Singapore	4.42	1.73	-61%	-32%	-14%	-69%	-43%	-52%	-50%	-56%	-59%	-73%	-82%
Slovakia	9.54	5.30	-44%	-26%	-59%	-47%	-32%	-46%	-40%	-40%	-24%	-59%	-55%
Slovenia	5.39	2.59	-52%	17%	-36%	-57%	-73%	-51%	-44%	-58%	-34%	-66%	-76%
Solomon Islands	31.31	33.44	7%	-22%	-13%	8%	-24%	-13%	17%	-8%	13%	57%	14%
Somalia	12.27	14.29	17%	10%	33%	13%	8%	25%	19%	12%	28%	15%	2%
South Africa	12.40	6.21	-50%	-57%	-50%	-55%	-45%	-43%	-27%	-49%	-46%	-69%	-55%
South Sudan	6.77	7.34	8%	-23%	-7%	-8%	-18%	-10%	13%	35%	-6%	-16%	-22%
Spain	7.45	1.94	-74%	-55%	-42%	-72%	-71%	-80%	-67%	-78%	-59%	-77%	-90%
Sri Lanka	21.07	10.55	-50%	-54%	-83%	-40%	-62%	-59%	-34%	-50%	-28%	-77%	-56%
St Vincent & the Grenadines	14.22	12.19	-14%	-20%	369%	-18%	-9%	10%	34%	-46%	-16%	-76%	-12%
Sudan	20.09	11.91	-41%	-57%	-45%	-51%	-44%	-24%	-18%	-53%	-17%	-38%	-39%
Suriname	15.04	11.41	-24%	-30%	367%	-24%	-33%	-8%	-3%	-35%	-33%	-77%	-14%
Sweden	3.20	1.63	-49%	-1%	3%	-52%	-19%	-63%	-36%	-52%	-55%	-53%	-51%
Switzerland	6.75	2.08	-69%	-9%	58%	-65%	68%	-82%	-61%	-66%	18%	-20%	-97%
Syrian Arab Republic	10.23	5.04	-51%	-53%	-63%	-53%	-69%	-59%	-29%	-53%	-41%	-41%	-59%
Taiwan (Province of China)	19.10	3.26	-83%	34%	-36%	-85%	-26%	-74%	-57%	-84%	-54%	-93%	-94%
Tajikistan	16.50	10.07	-39%	-24%	-13%	-36%	-13%	-3%	-31%	-52%	-7%	-76%	-13%
Thailand	18.98	11.64	-39%	-51%	-60%	-49%	-26%	-58%	-17%	-22%	109%	-48%	-24%
Timor-Leste	14.07	9.77	-31%	-46%	-46%	-32%	-49%	-34%	-3%	-44%	-8%	-36%	-33%
Togo	8.17	6.54	-20%	-30%	-3%	-31%	-2%	-21%	-14%	-13%	2%	-20%	-30%
Tokelau	10.58	7.81	-26%	-43%	-37%	-26%	-45%	-37%	1%	-25%	9%	-52%	-25%
Tonga	9.65	10.01	4%	-13%	9%	23%	0%	-5%	22%	10%	94%	-2%	0%
Trinidad and Tobago	13.65	6.57	-52%	-14%	210%	-60%	-30%	-43%	-31%	-45%	-36%	-77%	-54%
Tunisia	16.26	8.77	-46%	-57%	-56%	-56%	-44%	-43%	-25%	-52%	-21%	-43%	-45%
Turkey	9.07	4.37	-52%	-66%	-80%	-49%	-60%	-41%	-37%	-44%	-44%	-46%	-72%
Turkmenistan	16.87	10.41	-38%	35%	51%	-67%	989%	75%	-41%	-56%	6%	-36%	-18%
Tuvalu	20.44	14.10	-31%	-50%	-41%	-29%	-43%	-42%	-4%	-39%	-6%	-58%	-30%
Uganda	5.95	5.74	-3%	-21%	-9%	-3%	-20%	-7%	30%	1%	30%	-6%	-19%
Ukraine	20.92	17.18	-18%	62%	30%	-37%	7%	29%	-15%	-18%	67%	-15%	17%
United Arab Emirates	16.34	8.03	-51%	-62%	-64%	-58%	-53%	-44%	-44%	-50%	-45%	-55%	-55%
United Kingdom	4.29	1.77	-59%	19%	16%	-53%	-57%	-66%	-47%	-74%	-50%	-70%	-82%
United Republic of Tanzania	8.83	5.69	-36%	-35%	-35%	-28%	-39%	-32%	-15%	-23%	-17%	-33%	-42%
United States of America	7.04	2.96	-58%	-32%	-14%	-52%	-39%	-74%	-39%	-68%	-43%	-32%	-75%
United States Virgin Islands	14.84	5.95	-60%	-61%	-15%	-60%	-70%	-52%	-54%	-64%	-59%	-78%	-63%
Uruguay	18.70	9.24	-51%	6%	209%	-58%	-15%	-58%	-20%	2%	-37%	-49%	-55%
Uzbekistan	14.77	10.13	-31%	-23%	-1%	-41%	-7%	-11%	-19%	-47%	39%	9%	-33%
Vanuatu	15.62	18.35	17%	-8%	-12%	24%	-3%	-11%	40%	12%	74%	-20%	13%
Venezuela	14.19	9.36	-34%	-68%	18%	-63%	-2%	37%	-49%	-19%	-25%	-26%	-37%
Viet Nam	19.22	11.62	-40%	-42%	-58%	-35%	-54%	-53%	4%	-36%	-27%	-62%	-48%
Yemen	13.56	10.27	-24%	-40%	-38%	-40%	-21%	2%	3%	-34%	-10%	-22%	-23%
Zambia	9.72	7.06	-27%	-44%	-31%	-30%	-38%	-21%	-6%	-26%	-5%	-28%	-40%
Zimbabwe	11.58	18.49	60%	26%	41%	6%	23%	73%	37%	31%	51%	15%	22%

Figure S9: Heatmap of unintentional injury mechanism DALY rates and percentage change 1990-2019 by country, adolescents 10-24 years

	Unintentional injuries DALYs/100,000 1990	Unintentional injuries DALYs/100,000 2019	Unintentional injuries % change, 1990 to 2019	Percentage change in DALYs/ 100,000, 1990 to 2019, for mechanisms of Unintentional injury									
				Adverse effects of medical treatment	Animal contact	Drowning	Environmental heat and cold exposure	Exposure to mechanical forces	Falls	Fire, heat, and hot substances	Foreign body	Poisonings	Other unintentional injuries
Afghanistan	2,122	1,338	-37%	-54.6%	-8.6%	-47.3%	-56.9%	-22.0%	-14.2%	-36.9%	-22.9%	-43.8%	-46.9%
Albania	1,722	1,197	-31%	18.5%	-32.3%	-67.5%	-12.9%	-44.4%	32.5%	-44.3%	-13.3%	-5.4%	-50.2%
Algeria	1,623	744	-54%	-66.7%	-47.4%	-70.4%	-31.9%	-41.5%	-29.0%	-66.8%	-41.6%	-58.8%	-51.3%
American Samoa	1,369	1,207	-12%	-17.2%	6.2%	-15.2%	-32.3%	-32.0%	8.4%	-2.6%	0.2%	-36.4%	-34.8%
Andorra	552	509	-8%	-33.9%	-6.8%	-46.3%	-12.0%	-9.7%	0.6%	-17.4%	-19.3%	-25.1%	-9.5%
Angola	1,045	612	-41%	-52.0%	-54.5%	-49.0%	-50.4%	-39.9%	-13.8%	-42.8%	-26.3%	-49.8%	-50.0%
Antigua and Barbuda	1,472	1,248	-15%	-27.7%	32.5%	-26.3%	-9.1%	6.1%	37.1%	-42.7%	-25.7%	-70.0%	-19.7%
Argentina	1,400	1,005	-28%	-36.8%	-6.2%	-58.7%	-8.5%	-14.0%	-8.5%	-35.5%	-38.5%	-21.6%	-19.8%
Armenia	2,502	813	-67%	0.8%	-20.3%	-79.7%	-73.2%	-43.6%	-47.6%	-68.6%	-27.5%	-62.1%	-48.4%
Australia	1,099	940	-14%	-7.2%	-17.0%	-54.3%	-35.1%	-15.3%	10.3%	-31.0%	-25.2%	-40.8%	-28.3%
Austria	800	589	-26%	37.7%	-16.0%	-63.7%	-17.8%	-47.0%	-14.6%	-28.6%	-11.4%	-19.4%	-27.2%
Azerbaijan	1,568	1,073	-32%	-15.5%	-13.2%	-53.7%	-34.8%	-15.5%	-17.1%	-34.2%	-3.0%	-34.6%	-6.7%
Bahamas	1,215	1,800	48%	-17.5%	31.2%	-25.5%	-11.1%	3.8%	26.6%	-34.9%	-27.7%	-57.0%	-16.4%
Bahrain	742	541	-27%	-57.3%	-31.1%	-57.5%	-25.9%	-6.5%	0.1%	-33.9%	-22.4%	-49.5%	-17.5%
Bangladesh	1,156	755	-35%	-55.2%	-38.5%	-43.2%	-14.0%	-34.2%	116.7%	-42.5%	28.1%	-4.3%	-43.0%
Barbados	1,090	744	-32%	-35.0%	-5.2%	-43.6%	-11.9%	-2.8%	22.7%	-42.2%	-40.3%	-69.9%	-17.9%
Belarus	1,988	1,136	-43%	-16.7%	-18.6%	-63.6%	-48.7%	-22.2%	-16.7%	-37.5%	-17.7%	-49.9%	-34.9%
Belgium	747	589	-21%	-63.1%	-5.7%	-61.6%	-13.5%	-24.3%	5.0%	-40.7%	9.2%	-68.3%	-24.2%
Belize	1,328	1,284	-3%	-12.5%	110.7%	-6.2%	-4.6%	14.3%	49.6%	-24.8%	-19.9%	-69.9%	0.6%
Benin	783	612	-22%	-30.3%	-2.3%	-36.2%	-9.5%	-24.2%	-10.8%	-16.9%	-8.7%	-25.4%	-29.0%
Bermuda	888	581	-35%	-45.0%	-1.3%	-51.7%	-13.2%	-10.5%	14.4%	1.8%	-39.5%	-77.5%	-27.8%
Bhutan	1,162	921	-21%	-40.1%	-29.2%	-33.8%	-27.0%	-30.9%	43.4%	-22.8%	-18.7%	-24.3%	-8.7%
Bolivia	1,819	1,008	-45%	-57.5%	-1.3%	-54.3%	-27.2%	-37.2%	-15.2%	-58.2%	-48.2%	-59.0%	-53.7%
Bosnia and Herzegovina	1,627	999	-39%	-79.0%	-33.6%	-18.8%	156.2%	-54.8%	21.3%	-41.7%	-2.8%	-86.1%	-10.3%
Botswana	1,326	1,053	-21%	-25.7%	-23.2%	-19.8%	-38.4%	-30.8%	-3.4%	-15.4%	-0.4%	-20.7%	-31.6%
Brazil	1,392	739	-47%	-32.4%	-50.3%	-59.4%	-14.1%	-16.2%	-32.0%	-56.3%	-20.8%	-52.0%	-58.6%
Brunei Darussalam	1,657	807	-51%	-41.7%	-26.3%	-68.6%	-30.7%	-24.0%	-28.1%	-55.0%	-27.5%	-63.4%	-43.7%
Bulgaria	1,899	1,166	-39%	20.8%	-17.3%	-53.7%	-17.9%	-10.4%	-19.1%	-39.8%	-41.2%	-48.7%	-67.1%
Burkina Faso	649	606	-7%	-14.4%	-13.5%	-10.9%	-9.3%	-4.5%	6.6%	-3.9%	-0.8%	-9.9%	-13.4%
Burundi	1,317	917	-30%	-32.6%	-33.7%	-36.6%	-31.2%	-20.6%	-15.7%	-30.8%	-25.9%	-38.0%	-42.1%
Cabo Verde	608	782	29%	-32.5%	201.1%	87.2%	81.1%	-18.5%	1.1%	6.2%	59.9%	36.4%	-28.3%
Cambodia	1,970	1,115	-43%	-58.3%	-47.2%	-55.2%	-22.1%	-36.6%	-8.4%	-56.8%	-33.6%	-50.2%	-40.7%
Cameroon	693	616	-11%	-22.6%	1.8%	-19.7%	-5.0%	-13.1%	0.7%	-4.4%	4.8%	-10.8%	-18.7%
Canada	664	413	-38%	-34.6%	-9.6%	-51.0%	-44.4%	-39.9%	-11.7%	-57.6%	-37.3%	-38.6%	-40.2%
Central African Republic	1,274	1,008	-21%	-29.9%	-12.7%	-23.2%	-24.0%	-21.2%	-16.1%	-18.3%	-9.4%	-21.2%	-29.2%
Chad	798	798	0%	-14.2%	34.9%	-8.2%	15.5%	-10.2%	10.1%	5.5%	5.1%	0.2%	-8.9%
Chile	1,244	731	-41%	-51.5%	-30.4%	-45.1%	-61.0%	-21.1%	-4.0%	-65.7%	-82.9%	-41.0%	-35.0%
China	1,363	732	-46%	-75.9%	-75.6%	-58.7%	-64.9%	-33.9%	-24.5%	-51.1%	-35.6%	-17.9%	-18.4%
Colombia	1,600	686	-57%	-38.4%	-57.2%	-57.8%	-43.4%	-52.1%	-36.8%	-69.1%	-35.1%	-67.4%	-67.7%
Comoros	780	706	-9%	-28.1%	-27.7%	-27.8%	-24.5%	-12.6%	2.5%	-20.0%	1.6%	-26.0%	-29.4%
Congo	843	515	-39%	-50.5%	-29.8%	-48.8%	-49.1%	-39.4%	-19.3%	-38.8%	-29.1%	-44.9%	-42.8%
Cook Islands	767	403	-47%	-29.7%	-37.2%	-60.0%	-17.9%	-19.5%	2.4%	-10.0%	-33.2%	-65.1%	-19.9%
Costa Rica	1,061	738	-30%	-45.6%	-16.1%	-50.1%	1.4%	-13.6%	-0.3%	-39.3%	-45.4%	59.0%	-14.0%
Côte d'Ivoire	769	618	-20%	-30.2%	-19.0%	-30.3%	-7.1%	-20.1%	-7.1%	-20.2%	-7.3%	-23.4%	-25.6%
Croatia	1,530	759	-50%	14.9%	-19.2%	-55.3%	-44.6%	-38.4%	30.7%	-32.4%	-21.8%	-45.2%	-88.5%
Cuba	1,154	723	-37%	-60.2%	9.8%	-53.5%	-11.9%	-23.0%	-18.3%	-41.6%	-53.7%	-34.9%	-31.0%
Cyprus	674	587	-13%	-36.2%	-13.8%	-48.2%	-21.2%	-28.4%	15.2%	-31.9%	-7.7%	-22.2%	-18.6%
Czechia	1,312	861	-34%	59.0%	-20.9%	-67.8%	-16.2%	-22.7%	-5.2%	-37.0%	-46.4%	-59.2%	-51.6%
Denmark	550	501	-9%	30.6%	9.5%	-50.4%	-0.8%	-24.6%	19.8%	-28.9%	0.6%	-9.8%	-39.8%
DPR of Korea	1,450	1,064	-27%	-47.9%	-36.2%	-39.1%	-19.4%	-3.1%	-13.2%	-35.5%	-3.1%	-14.8%	-2.1%
DR of the Congo	779	610	-22%	-35.0%	-13.9%	-33.6%	-27.2%	-13.2%	-5.8%	-25.8%	-9.1%	-23.6%	-27.3%

	Unintentional injuries DALYs/100,000 1990	Unintentional injuries DALYs/100,000 2019	Unintentional injuries % change, 1990 to 2019	Percentage change in DALYs/ 100,000, 1990 to 2019, for mechanisms of Unitentional injury									
				Adverse effects of medical treatment	Animal contact	Drowning	Environmental heat and cold exposure	Exposure to mechanical forces	Falls	Fire, heat, and hot substances	Foreign body	Poisonings	Other unintentional injuries
Djibouti	754	670	-11%	-30.1%	8.7%	-15.5%	-27.7%	-13.7%	7.6%	-17.0%	5.4%	-13.4%	-20.8%
Dominica	1,485	1,210	-18%	-20.1%	13.9%	-16.2%	-3.8%	8.4%	24.8%	-18.0%	-23.4%	-74.6%	-3.4%
Dominican Republic	1,048	976	-7%	-12.1%	-17.9%	-7.7%	-15.0%	13.4%	24.0%	-34.6%	-0.2%	-19.0%	-10.0%
Ecuador	1,603	1,014	-37%	-51.0%	66.6%	-42.1%	-18.1%	-37.0%	-9.6%	-47.0%	-33.3%	-46.7%	-44.3%
Egypt	936	539	-42%	-61.0%	-36.7%	-55.4%	-32.7%	-28.5%	-11.5%	-52.8%	-30.5%	-56.3%	-42.0%
El Salvador	1,594	786	-51%	-73.4%	-11.5%	-13.5%	-20.6%	-38.9%	-28.2%	-59.6%	-83.6%	-70.3%	-76.2%
Equatorial Guinea	1,138	425	-63%	-78.1%	-75.0%	-75.4%	-69.1%	-60.3%	-28.6%	-61.1%	-41.9%	-71.2%	-62.8%
Eritrea	1,053	871	-17%	-30.0%	-14.5%	-26.3%	-32.8%	-15.4%	5.2%	-18.0%	1.1%	-21.2%	-26.2%
Estonia	2,394	809	-66%	-63.7%	-14.1%	-85.4%	-63.8%	-42.7%	-48.0%	-72.7%	-59.8%	-76.2%	-57.4%
Eswatini	1,234	1,283	4%	-8.9%	5.6%	2.3%	5.1%	0.1%	22.0%	9.1%	21.5%	-8.0%	-5.8%
Ethiopia	1,467	741	-49%	-64.0%	-51.4%	-67.2%	-50.4%	-36.0%	-29.5%	-47.5%	-31.0%	-62.9%	-52.3%
Fiji	1,464	1,196	-18%	-31.1%	-27.7%	-20.2%	-25.3%	-24.9%	10.2%	-19.3%	3.0%	-41.4%	-35.9%
Finland	838	715	-15%	-26.9%	-30.3%	-56.8%	18.4%	-21.5%	10.1%	-44.9%	-20.7%	-38.5%	-35.0%
France	827	599	-27%	-70.7%	-19.9%	-48.9%	138.9%	-30.4%	-11.2%	-40.1%	-48.3%	-37.4%	-41.2%
Gabon	782	516	-34%	-50.3%	-29.3%	-48.8%	-41.4%	-31.5%	-19.2%	-32.5%	-20.0%	-39.2%	-36.0%
Gambia	625	568	-9%	-22.8%	-17.3%	-21.2%	1.5%	-10.3%	4.7%	-2.5%	8.3%	-12.7%	-17.4%
Georgia	1,933	1,254	-35%	146.3%	-13.9%	-78.0%	-15.2%	9.5%	-2.5%	-52.5%	24.7%	-56.4%	11.7%
Germany	640	518	-19%	2.3%	-12.2%	-54.4%	-23.9%	-19.7%	-0.3%	-43.8%	-35.4%	-25.1%	-30.6%
Ghana	649	544	-16%	-23.7%	-24.2%	-26.7%	-23.4%	-11.8%	3.5%	-15.4%	0.1%	-22.0%	-28.1%
Greece	780	561	-28%	-28.0%	-3.7%	-37.5%	-18.4%	-15.9%	-13.4%	-38.3%	-59.3%	-36.0%	-47.6%
Greenland	1,701	953	-44%	-35.8%	-53.0%	-48.6%	-40.7%	-46.2%	-29.2%	-57.4%	-27.3%	-56.6%	-45.0%
Grenada	1,549	1,240	-20%	-39.1%	46.3%	-33.7%	-7.3%	13.5%	42.2%	-37.9%	-20.4%	-78.7%	-12.7%
Guam	859	546	-36%	-1.6%	4.1%	-48.9%	-34.7%	-54.7%	0.2%	-5.2%	-14.3%	-61.6%	-41.6%
Guatemala	2,348	1,664	-29%	-74.5%	-4.0%	-48.4%	57.6%	95.0%	-21.0%	-61.3%	-58.8%	5.9%	-27.4%
Guinea	849	777	-9%	-18.7%	16.3%	-17.9%	3.1%	-7.2%	0.3%	-3.4%	-0.2%	-7.9%	-18.8%
Guinea-Bissau	1,217	827	-32%	-39.8%	-20.3%	-44.8%	-18.8%	-30.3%	-20.3%	-27.4%	-19.1%	-36.0%	-38.5%
Guyana	1,882	1,702	-10%	-11.5%	160.4%	10.5%	-14.9%	17.4%	10.5%	-33.6%	-29.8%	-65.5%	-38.3%
Haiti	2,915	2,645	-9%	-38.9%	3.5%	-44.2%	-30.6%	-22.7%	-11.1%	-47.6%	-43.2%	-47.7%	-40.9%
Honduras	1,972	929	-53%	-63.1%	-42.6%	-68.2%	-29.4%	-45.8%	-22.3%	-41.6%	-58.8%	-59.5%	-60.2%
Hungary	1,137	820	-28%	-36.7%	-21.0%	-66.5%	-37.6%	-13.8%	14.0%	-41.4%	-37.1%	-45.4%	-38.5%
Iceland	714	525	-27%	-36.2%	-16.4%	-68.8%	-44.1%	-39.3%	1.9%	-43.9%	-24.2%	-49.3%	-32.9%
India	1,765	1,006	-43%	-47.1%	-48.9%	-51.4%	-39.3%	-40.8%	-25.3%	-39.0%	-19.5%	-51.5%	-45.8%
Indonesia	935	577	-38%	-53.5%	-39.4%	-53.2%	-13.7%	-36.5%	-23.0%	-30.1%	-44.1%	-15.0%	-55.1%
Iran	5,376	853	-84%	-69.0%	-44.9%	-65.9%	-14.6%	-20.3%	-29.3%	-68.8%	-12.7%	-55.7%	-34.9%
Iraq	3,106	1,591	-49%	-50.5%	-64.4%	-55.4%	-49.4%	-45.6%	-32.4%	-52.4%	-32.6%	-46.4%	-54.8%
Ireland	752	549	-27%	-15.5%	-22.0%	-63.7%	-22.2%	-42.9%	12.5%	-50.4%	-44.4%	-36.8%	-29.6%
Israel	726	512	-29%	-73.9%	-16.1%	-64.4%	-22.1%	-45.9%	12.8%	-33.7%	-37.8%	-30.7%	-34.5%
Italy	736	441	-40%	-34.5%	-54.0%	-54.2%	-43.6%	-55.0%	-12.6%	-53.1%	-25.2%	-72.8%	-61.4%
Jamaica	581	559	-4%	117.6%	-15.8%	-45.5%	-0.5%	33.5%	36.4%	-17.9%	-61.9%	-19.0%	-19.9%
Japan	569	480	-16%	24.1%	-13.4%	-39.9%	-0.5%	-17.3%	1.5%	-28.3%	-5.6%	-32.1%	-21.0%
Jordan	1,114	575	-48%	-56.0%	-24.4%	-57.5%	-32.4%	-52.9%	-23.2%	-57.6%	-31.8%	-64.5%	-49.9%
Kazakhstan	2,120	1,135	-46%	-37.8%	-29.3%	-60.2%	-57.8%	-36.4%	-30.3%	-60.8%	-27.4%	-44.8%	-26.8%
Kenya	675	641	-5%	-21.6%	4.1%	-10.6%	-4.9%	-0.2%	7.7%	-13.8%	8.1%	4.4%	-8.7%
Kiribati	1,209	978	-19%	-29.8%	-22.2%	-24.0%	16.5%	-21.2%	8.3%	-3.7%	-13.9%	-23.0%	-23.7%
Kuwait	680	429	-37%	-51.3%	-19.5%	-71.2%	-16.6%	-32.9%	-8.1%	-41.8%	-49.2%	-52.5%	-29.2%
Kyrgyzstan	1,853	812	-56%	-15.8%	-18.3%	-74.4%	-39.3%	-15.4%	-47.9%	-55.7%	-33.7%	-55.1%	-48.6%
Lao PDR	2,196	1,050	-52%	-61.7%	-53.7%	-62.4%	-48.9%	-41.9%	-26.0%	-60.8%	-45.2%	-66.6%	-41.2%
Latvia	2,432	957	-61%	-7.4%	-12.5%	-75.4%	-50.5%	-44.7%	-49.1%	-65.7%	-42.1%	-68.2%	-39.9%
Lebanon	1,006	635	-37%	-55.2%	-26.8%	-50.1%	-27.7%	-28.8%	4.7%	-62.2%	-16.6%	-38.0%	-31.8%
Lesotho	1,407	1,433	2%	-1.8%	5.2%	4.5%	11.2%	-1.6%	23.3%	20.4%	27.9%	2.4%	0.4%
Liberia	784	590	-25%	-32.4%	-10.5%	-43.4%	-14.1%	-20.8%	-17.2%	8.2%	-15.1%	-29.5%	-36.9%

	Unintentional injuries	Unintentional injuries	Unintentional injuries	Percentage change in DALYs/ 100,000, 1990 to 2019, for mechanisms of Unitentional injury									
	DALYs/100,000 1990	DALYs/100,000 2019	% change, 1990 to 2019	Adverse effects of medical treatment	Animal contact	Drowning	Environmental heat and cold exposure	Exposure to mechanical forces	Falls	Fire, heat, and hot substances	Foreign body	Poisonings	Other unintentional injuries
Libya	1,011	681	-33%	-50.7%	-25.1%	-51.5%	-20.0%	-24.9%	-9.9%	-46.9%	-11.9%	-30.6%	-32.0%
Lithuania	2,044	958	-53%	-3.1%	-21.1%	-74.5%	-34.6%	-27.4%	-33.6%	-58.6%	-21.2%	-43.6%	-49.4%
Luxembourg	955	592	-38%	-50.3%	-33.9%	-63.5%	-30.0%	-44.0%	-14.7%	-37.2%	-36.2%	-59.2%	-62.9%
Madagascar	1,202	679	-44%	-54.8%	-47.4%	-57.1%	-45.0%	-34.3%	-19.8%	-42.6%	-29.3%	-52.6%	-52.5%
Malawi	896	679	-24%	-43.5%	-33.9%	-38.4%	-31.1%	-19.5%	-2.9%	-26.1%	-12.2%	-35.6%	-37.9%
Malaysia	921	625	-32%	-50.3%	-37.9%	-35.9%	-25.7%	-28.2%	-10.1%	-43.5%	-19.6%	-44.0%	-39.5%
Maldives	1,731	651	-62%	-74.9%	-71.7%	-72.0%	-44.6%	-56.0%	-24.8%	-69.9%	-64.1%	-55.3%	-61.0%
Mali	888	670	-25%	-38.4%	-30.2%	-35.7%	-31.8%	-22.2%	-5.8%	-24.6%	14.6%	-33.2%	-36.8%
Malta	716	675	-6%	-24.7%	-11.9%	-24.5%	-16.7%	-17.1%	18.2%	-15.7%	7.2%	-31.3%	-35.8%
Marshall Islands	1,726	1,409	-18%	-32.5%	-40.2%	-16.4%	-40.9%	-34.3%	1.5%	-13.4%	19.1%	-38.9%	-25.0%
Mauritania	688	409	-41%	-52.8%	-32.7%	-54.4%	-40.5%	-39.1%	-22.0%	-36.3%	-29.2%	-48.4%	-50.5%
Mauritius	978	609	-38%	50.3%	-16.9%	-30.6%	-13.2%	-19.3%	35.8%	-76.9%	22.6%	21.4%	-38.5%
Mexico	1,603	769	-52%	-35.4%	-49.3%	-63.7%	-53.1%	-56.0%	-41.1%	-42.3%	-29.1%	-55.9%	-59.1%
Micronesia	1,575	1,509	-4%	-28.1%	-14.6%	-0.7%	-23.2%	-25.6%	23.1%	-14.1%	26.2%	-39.3%	-5.5%
Monaco	736	635	-14%	-32.9%	-18.8%	-28.7%	-12.9%	-16.2%	14.6%	-18.8%	9.0%	-58.3%	-19.1%
Mongolia	2,056	1,563	-24%	-30.6%	11.2%	-19.8%	-46.1%	-23.9%	27.7%	-6.8%	45.6%	-71.9%	4.2%
Montenegro	1,156	923	-20%	-26.4%	-8.9%	-42.9%	-42.0%	-9.1%	-3.1%	-31.0%	-23.1%	-26.5%	-36.6%
Morocco	1,384	850	-39%	-53.3%	-32.8%	-46.6%	-41.4%	-38.3%	-12.5%	-53.6%	-19.9%	-41.6%	-45.7%
Mozambique	894	961	8%	-30.6%	-19.0%	-23.5%	-15.8%	-3.7%	10.7%	-15.9%	10.4%	-14.8%	-19.2%
Myanmar	2,428	1,468	-40%	-61.7%	-57.7%	-57.2%	-20.9%	-44.9%	-20.0%	-45.2%	-37.3%	-58.9%	-40.9%
Namibia	1,016	866	-15%	-26.7%	-24.3%	-17.4%	-31.8%	-17.0%	15.3%	-13.3%	-2.8%	-25.5%	-23.4%
Nauru	1,525	1,524	0%	-26.9%	-5.3%	1.9%	-22.6%	-19.9%	20.6%	-9.3%	33.0%	-26.1%	-0.3%
Nepal	1,649	1,116	-32%	-50.1%	-42.7%	-45.6%	-44.0%	-25.8%	-16.1%	-34.2%	-22.4%	-49.8%	-30.9%
Netherlands	464	395	-15%	-42.8%	-15.2%	-53.8%	-18.6%	-25.8%	9.6%	-31.3%	-20.8%	-34.8%	-20.2%
New Zealand	1,407	1,136	-19%	-23.6%	-28.9%	-51.6%	-51.1%	-24.8%	14.6%	-50.2%	-8.0%	-24.8%	-34.6%
Nicaragua	1,156	794	-31%	-52.0%	-20.2%	-28.7%	-14.2%	-35.3%	2.9%	-51.7%	-39.8%	-28.0%	-42.1%
Niger	791	621	-21%	-28.5%	-21.3%	-30.5%	-27.7%	-15.7%	-8.3%	-20.6%	-16.9%	-25.1%	-28.5%
Nigeria	584	424	-27%	-38.1%	-30.5%	-36.3%	-30.9%	-32.9%	-4.4%	-25.6%	-9.7%	-39.1%	-38.9%
Niue	1,149	1,034	-10%	-37.1%	-24.7%	-11.6%	-33.3%	-30.1%	12.1%	-14.2%	28.3%	-35.0%	-15.7%
North Macedonia	1,432	883	-38%	-32.3%	-15.0%	-43.5%	-15.3%	-5.4%	36.9%	-38.6%	-5.0%	-9.0%	-74.8%
Northern Mariana Islands	1,180	984	-17%	-62.2%	-2.6%	-6.9%	-27.7%	-48.7%	7.1%	3.5%	46.8%	-48.8%	-42.7%
Norway	773	575	-26%	-37.7%	-5.0%	-66.2%	-41.3%	-39.8%	-0.9%	-47.7%	-27.8%	31.2%	-37.9%
Oman	1,252	783	-37%	-56.3%	-14.2%	-49.8%	-39.0%	-49.7%	-4.0%	-39.2%	-16.7%	-40.2%	-10.2%
Pakistan	693	676	-2%	-1.6%	14.7%	-52.3%	-8.4%	-9.6%	31.0%	11.5%	39.6%	1.3%	-8.1%
Palau	2,394	1,992	-17%	-25.4%	6.0%	-20.6%	-18.5%	-19.2%	0.9%	-22.0%	-0.9%	-27.4%	-20.9%
Palestine	985	592	-40%	-55.8%	-12.4%	-60.7%	-20.2%	-44.5%	-16.0%	-55.7%	1.1%	-48.5%	-44.4%
Panama	1,376	768	-44%	-73.0%	-33.6%	-59.4%	-16.6%	-24.8%	-11.2%	-39.3%	-62.3%	-31.9%	-35.8%
Papua New Guinea	973	993	2%	-24.5%	-12.6%	-6.0%	-6.8%	0.1%	26.6%	3.1%	-6.4%	0.2%	4.5%
Paraguay	1,022	881	-14%	-3.5%	32.3%	-13.2%	-4.1%	-29.4%	-2.4%	-32.7%	-35.6%	-46.7%	-8.2%
Peru	1,838	923	-50%	-64.9%	-11.7%	-59.0%	-35.1%	-30.2%	-6.9%	-58.9%	-51.7%	-56.2%	-56.9%
Philippines	1,489	724	-51%	-33.0%	-47.1%	-46.5%	-29.8%	-19.6%	-15.1%	-32.0%	-15.2%	-35.0%	-38.7%
Poland	1,516	865	-43%	7.3%	-29.8%	-72.0%	-55.8%	-23.7%	-3.3%	-45.4%	-34.4%	-68.7%	-58.6%
Portugal	1,036	440	-58%	-26.6%	-23.5%	-78.1%	-49.0%	-59.2%	-46.7%	-59.4%	-32.4%	-62.0%	-62.9%
Puerto Rico	738	494	-33%	-40.1%	-7.8%	-74.1%	-2.1%	22.3%	14.1%	-8.6%	-21.9%	-86.8%	-26.1%
Qatar	1,243	838	-33%	-58.1%	-22.8%	-56.6%	-38.9%	-32.9%	-4.4%	-54.9%	-24.9%	-22.2%	-28.7%
Republic of Korea	1,591	559	-65%	-56.1%	-18.5%	-89.4%	-59.6%	-29.2%	-43.0%	-66.8%	-71.0%	-85.9%	-60.4%
Republic of Moldova	2,078	1,043	-50%	-63.5%	-12.3%	-65.9%	-6.7%	-38.7%	-33.8%	-57.1%	-5.7%	-32.3%	-61.2%
Romania	2,002	1,078	-46%	-30.8%	-18.4%	-59.4%	-47.3%	-28.5%	-40.1%	-58.9%	-22.9%	-66.9%	-41.7%
Russian Federation	2,232	1,217	-45%	25.9%	-31.6%	-55.2%	-74.7%	-58.0%	-17.6%	-48.0%	7.0%	-20.1%	-48.5%
Rwanda	1,060	600	-43%	-56.1%	-48.2%	-58.5%	-53.2%	-29.4%	-17.7%	-40.1%	-34.1%	-54.5%	-54.6%
Saint Kitts and Nevis	1,250	988	-21%	-42.5%	14.7%	-32.3%	-5.5%	6.5%	22.7%	-45.3%	-37.9%	-66.2%	-21.8%

	Unintentional injuries DALYs/100,000 1990	Unintentional injuries DALYs/100,000 2019	Unintentional injuries % change, 1990 to 2019	Percentage change in DALYs/ 100,000, 1990 to 2019, for mechanisms of Unintentional injury									
				Adverse effects of medical treatment	Animal contact	Drowning	Environmental heat and cold exposure	Exposure to mechanical forces	Falls	Fire, heat, and hot substances	Foreign body	Poisonings	Other unintentional injuries
Saint Lucia	1,228	1,013	-18%	-22.6%	19.0%	-25.6%	-11.4%	15.1%	33.5%	-39.1%	-31.4%	-63.3%	-17.8%
Samoa	1,455	1,018	-30%	-26.3%	-30.0%	-16.2%	-24.2%	-29.2%	10.4%	-15.1%	11.8%	-44.1%	-15.9%
San Marino	532	530	0%	-26.8%	-6.1%	-7.6%	-12.6%	-17.7%	19.5%	-1.1%	1.4%	-26.9%	-20.6%
Sao Tome and Principe	662	548	-17%	-23.3%	-5.4%	-35.7%	-5.4%	-11.1%	-12.3%	-0.4%	-7.6%	-22.5%	-20.7%
Saudi Arabia	2,759	1,330	-52%	-60.8%	-32.0%	-44.0%	-24.6%	-79.5%	-7.9%	-34.1%	-14.8%	-39.2%	-61.2%
Senegal	720	577	-20%	-29.8%	-22.5%	-31.9%	-22.4%	-13.7%	-3.3%	-19.4%	-9.6%	-23.3%	-29.8%
Serbia	1,219	809	-34%	-58.7%	-19.4%	-71.0%	-56.7%	-16.6%	7.9%	-46.3%	-36.2%	-42.0%	-54.0%
Seychelles	1,310	1,009	-23%	-7.0%	-8.0%	-16.3%	8.7%	-49.4%	-11.6%	-44.7%	-4.5%	-28.6%	-38.9%
Sierra Leone	745	689	-8%	-13.1%	14.7%	-22.3%	-0.6%	-13.1%	-9.4%	-5.6%	-0.3%	-8.9%	-22.1%
Singapore	700	491	-30%	-24.8%	-26.2%	-67.2%	-9.0%	-21.0%	-18.2%	-24.4%	-34.7%	-33.8%	-38.3%
Slovakia	1,283	992	-23%	-21.9%	-30.3%	-47.2%	-30.3%	-18.2%	-1.9%	-30.5%	-21.7%	-36.6%	-31.4%
Slovenia	1,028	880	-14%	16.6%	-17.7%	-55.5%	-62.2%	-17.6%	6.7%	-17.9%	-20.6%	-26.2%	-18.6%
Solomon Islands	2,384	2,519	6%	-22.4%	-12.8%	7.9%	-21.6%	-13.0%	18.9%	-10.2%	10.0%	53.1%	11.1%
Somalia	1,066	1,213	14%	9.8%	29.0%	12.1%	7.0%	20.4%	17.8%	3.4%	15.8%	13.3%	0.4%
South Africa	1,043	575	-45%	-56.0%	-43.6%	-56.7%	-36.1%	-38.8%	-14.5%	-49.0%	-33.9%	-67.0%	-50.6%
South Sudan	681	719	6%	-21.6%	-7.7%	-7.0%	-14.4%	-7.9%	8.9%	11.0%	-6.2%	-14.3%	-19.0%
Spain	857	513	-40%	-50.6%	-24.6%	-71.8%	-37.8%	-46.0%	-8.5%	-46.1%	-45.3%	-58.3%	-62.9%
Sri Lanka	1,673	921	-45%	-53.5%	-81.0%	-40.3%	-50.2%	-54.4%	-24.5%	-51.6%	-26.8%	-74.2%	-52.6%
St Vincent & the Grenadines	1,334	1,180	-12%	-18.3%	35.1%	-18.2%	-3.0%	11.2%	39.6%	-38.1%	-17.0%	-55.2%	-9.3%
Sudan	1,616	1,015	-37%	-56.0%	-37.3%	-50.8%	-24.4%	-22.6%	-9.3%	-52.9%	-14.9%	-38.1%	-38.6%
Suriname	1,387	1,115	-20%	-29.6%	104.6%	-23.2%	-9.5%	-2.3%	17.4%	-34.2%	-29.9%	-66.9%	-9.5%
Sweden	629	519	-17%	1.5%	-7.7%	-51.6%	-19.0%	-31.5%	10.5%	-35.2%	-28.7%	-43.6%	-19.9%
Switzerland	985	631	-36%	-5.4%	12.4%	-63.9%	23.4%	-56.0%	-16.6%	-29.3%	11.2%	-7.3%	-64.4%
Syrian Arab Republic	950	588	-38%	-50.7%	-37.2%	-52.5%	-44.0%	-46.7%	2.0%	-52.4%	-36.0%	-38.9%	-45.2%
Taiwan (Province of China)	1,445	302	-79%	30.5%	-30.4%	-84.5%	-12.1%	-63.3%	-51.4%	-80.1%	-48.5%	-90.5%	-92.3%
Tajikistan	1,646	1,108	-33%	-21.7%	7.4%	-36.3%	-5.9%	2.3%	-25.8%	-48.2%	-6.0%	-58.0%	-10.0%
Thailand	1,537	962	-37%	-50.9%	-57.9%	-49.2%	-24.8%	-52.2%	-12.1%	-31.6%	68.1%	-45.4%	-26.8%
Timor-Leste	1,209	847	-30%	-45.9%	-44.5%	-33.0%	-35.3%	-30.5%	1.5%	-48.9%	-11.7%	-34.9%	-32.4%
Togo	725	599	-17%	-29.7%	-2.7%	-31.8%	-1.4%	-17.0%	-9.3%	-14.2%	-0.7%	-20.3%	-26.2%
Tokelau	858	673	-22%	-42.9%	-30.3%	-26.9%	-34.6%	-32.2%	15.9%	-12.9%	5.3%	-49.7%	-23.9%
Tonga	836	858	3%	-12.3%	-1.3%	22.3%	-6.0%	-8.2%	30.2%	-1.0%	70.9%	-3.3%	-2.7%
Trinidad and Tobago	1,269	731	-42%	-14.4%	2.9%	-60.0%	-9.8%	-21.7%	-10.1%	-40.1%	-31.6%	-59.8%	-35.4%
Tunisia	1,371	804	-41%	-55.4%	-38.4%	-56.2%	-25.4%	-42.1%	-9.9%	-52.5%	-19.5%	-42.6%	-34.9%
Turkey	866	513	-41%	-59.7%	-47.9%	-49.1%	-34.5%	-35.2%	-6.8%	-48.8%	-34.7%	-44.9%	-59.8%
Turkmenistan	1,653	1,134	-31%	31.6%	6.3%	-67.0%	328.1%	34.8%	-19.8%	-55.9%	-1.1%	-28.3%	-12.9%
Tuvalu	1,545	1,101	-29%	-50.1%	-33.1%	-30.2%	-35.6%	-38.3%	7.5%	-30.5%	-6.9%	-56.6%	-29.3%
Uganda	611	593	-3%	-20.4%	-9.6%	-2.8%	-15.8%	-5.8%	13.9%	-5.0%	9.6%	-5.7%	-14.9%
Ukraine	2,028	1,648	-19%	56.2%	8.3%	-37.7%	-3.8%	4.2%	-20.4%	-24.6%	42.8%	-13.8%	4.5%
United Arab Emirates	1,405	777	-45%	-60.0%	-53.6%	-57.7%	-28.4%	-40.4%	-32.7%	-48.9%	-39.1%	-48.4%	-46.3%
United Kingdom	710	510	-28%	13.6%	-4.8%	-53.0%	-34.5%	-26.8%	0.0%	-52.8%	-39.9%	-55.8%	-44.0%
United Republic of Tanzania	825	596	-28%	-35.0%	-30.6%	-28.0%	-31.9%	-22.7%	-3.5%	-16.3%	-13.3%	-31.8%	-37.2%
United States of America	840	466	-45%	-22.5%	0.4%	-52.1%	-27.9%	-58.5%	-29.1%	-58.3%	-28.7%	-30.9%	-38.8%
United States Virgin Islands	1,302	673	-48%	-58.9%	-20.1%	-60.6%	-31.3%	-28.5%	-22.5%	-33.7%	-50.7%	-63.9%	-48.9%
Uruguay	1,842	1,091	-41%	5.4%	2.5%	-58.7%	-5.0%	-48.0%	1.0%	-18.5%	-35.4%	-33.0%	-44.6%
Uzbekistan	1,496	1,090	-27%	-19.3%	-9.3%	-41.4%	-12.1%	-2.4%	-13.9%	-45.3%	20.3%	6.4%	-30.4%
Vanuatu	1,281	1,449	13%	-8.6%	-11.8%	22.9%	-4.7%	-11.6%	35.0%	1.0%	64.3%	-19.7%	9.6%
Venezuela	1,488	1,034	-31%	-66.6%	-1.9%	-63.0%	-19.9%	18.8%	-26.5%	-44.9%	-26.5%	-22.3%	-32.6%
Viet Nam	1,558	977	-37%	-41.4%	-52.2%	-35.8%	-38.2%	-46.7%	10.0%	-38.9%	-25.8%	-60.9%	-46.2%
Yemen	1,221	921	-25%	-40.1%	-23.5%	-41.3%	-14.6%	-3.2%	3.0%	-37.8%	-10.8%	-24.0%	-25.9%
Zambia	876	677	-23%	-43.5%	-28.4%	-30.9%	-32.7%	-18.0%	2.5%	-24.7%	-4.7%	-28.7%	-36.9%
Zimbabwe	976	1,475	51%	24.8%	33.6%	5.8%	12.6%	51.6%	21.8%	26.8%	30.1%	13.0%	18.4%

Figure S10: Exposure to forces of nature absolute number, and rate per 100,000 population of DALYs and deaths, annual trends and percentage change, 1990-2019

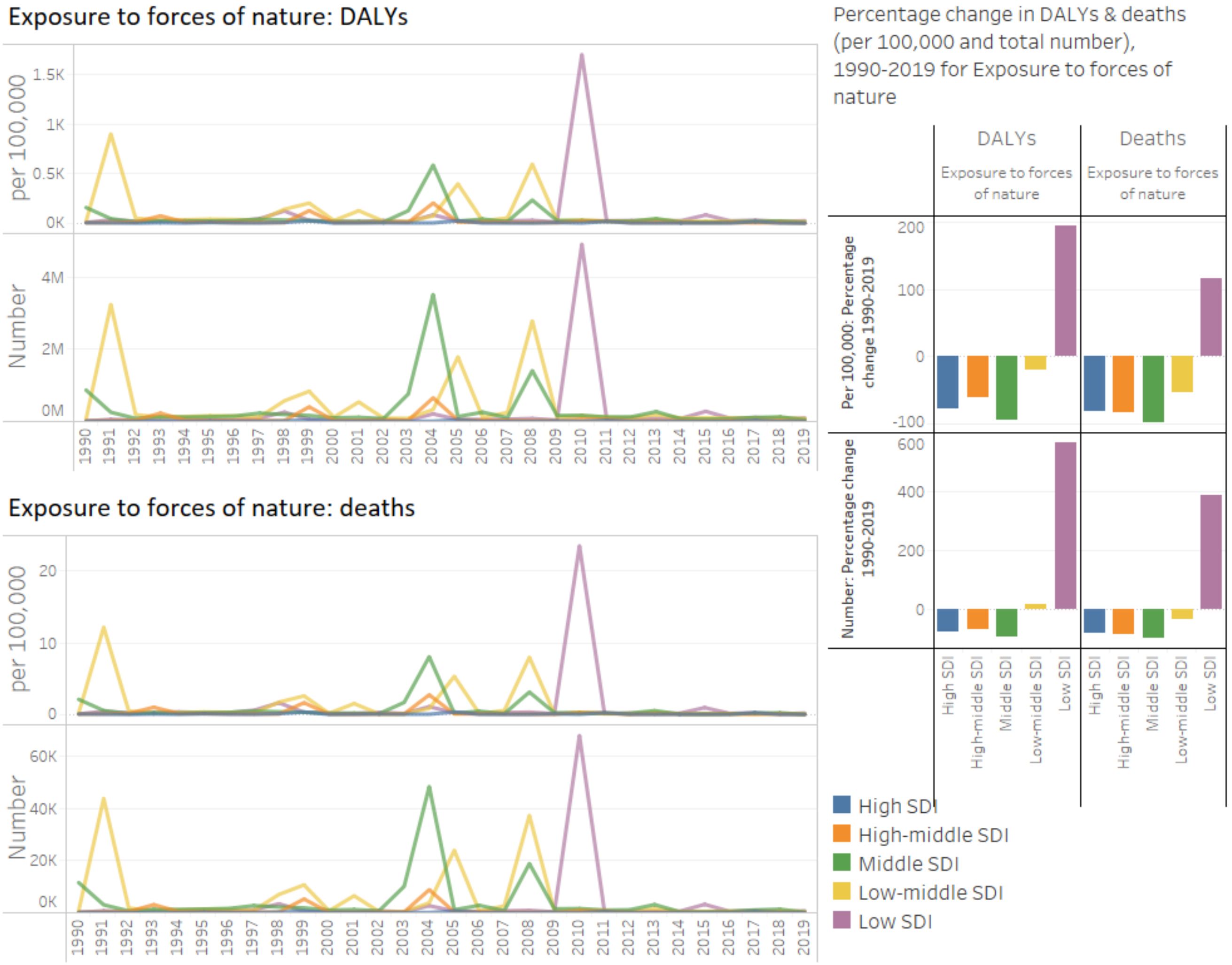

Figure S11: Unintentional injury DALY rates per 100,000 population, 1990 vs 2019 by sex and country (coloured according to SDI), adolescents 10-24 years

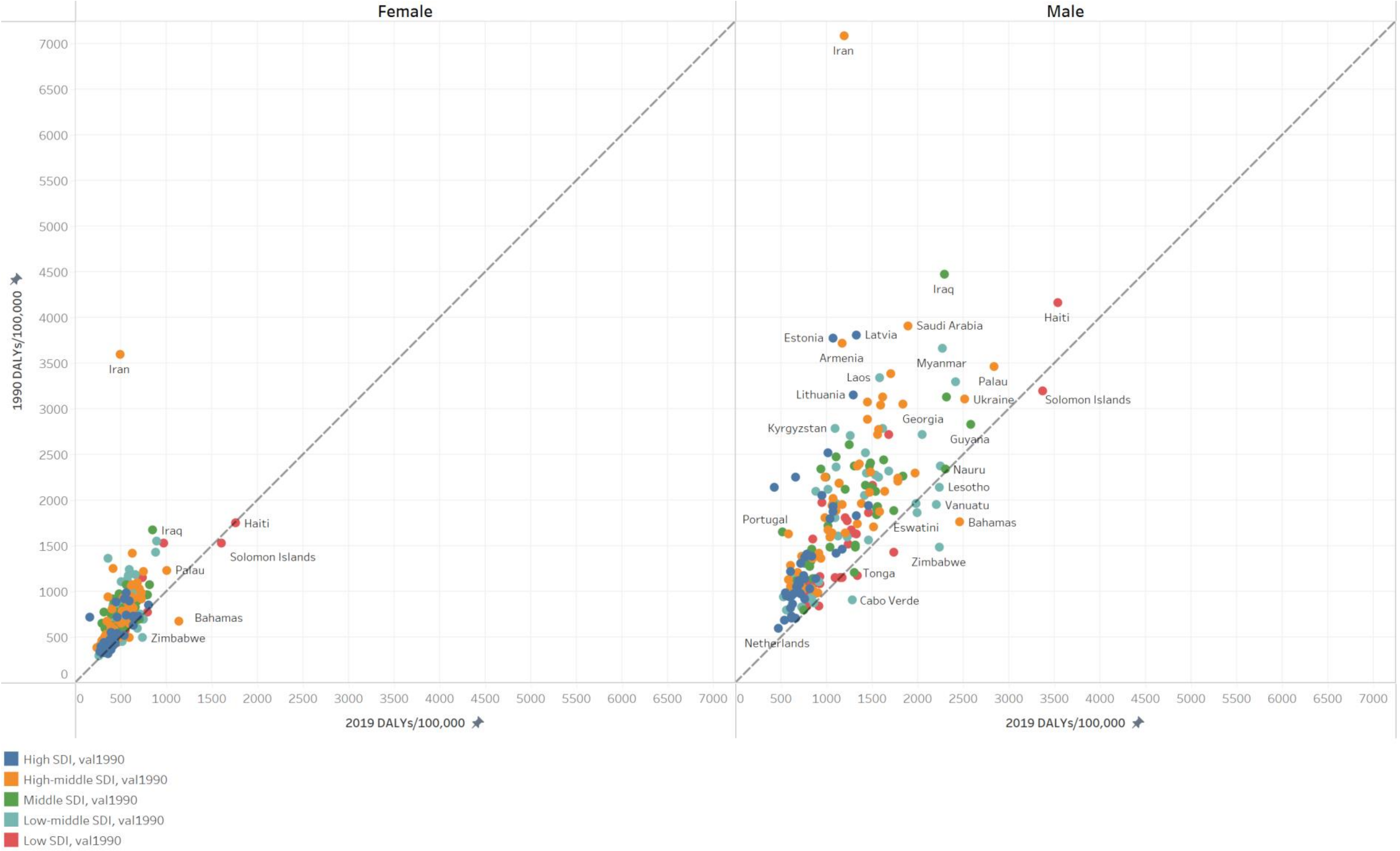

GATHER Checklist

GATHER
Guidelines for Accurate and Transparent Health Estimates Reporting

Item #	Checklist item	Reported on page #
**Objectives and funding**		
1	Define the indicator(s), populations (including age, sex, and geographic entities), and time period(s) for which estimates were made.	5
2	List the funding sources for the work.	3 & 13
**Data Inputs**		
For all data inputs from multiple sources that are synthesized as part of the study:		
3	Describe how the data were identified and how the data were accessed.	4-5
4	Specify the inclusion and exclusion criteria. Identify all ad-hoc exclusions.	4-5
5	Provide information on all included data sources and their main characteristics. For each data source used, report reference information or contact name/institution, population represented, data collection method, year(s) of data collection, sex and age range, diagnostic criteria or measurement method, and sample size, as relevant.	4-5
6	Identify and describe any categories of input data that have potentially important biases (e.g., based on characteristics listed in item 5).	4-5 & 12
For data inputs that contribute to the analysis but were not synthesized as part of the study:		
7	Describe and give sources for any other data inputs.	N/A
For all data inputs:		
8	Provide all data inputs in a file format from which data can be efficiently extracted (e.g., a spreadsheet rather than a PDF), including all relevant meta-data listed in item 5. For any data inputs that cannot be shared because of ethical or legal reasons, such as third-party ownership, provide a contact name or the name of the institution that retains the right to the data.	13
**Data analysis**		
9	Provide a conceptual overview of the data analysis method. A diagram may be helpful.	4-5
10	Provide a detailed description of all steps of the analysis, including mathematical formulae. This description should cover, as relevant, data cleaning, data pre-processing, data adjustments and weighting of data sources, and mathematical or statistical model(s).	4-5
11	Describe how candidate models were evaluated and how the final model(s) were selected.	N/A
12	Provide the results of an evaluation of model performance, if done, as well as the results of any relevant sensitivity analysis.	N/A
13	Describe methods for calculating uncertainty of the estimates. State which sources of uncertainty were, and were not, accounted for in the uncertainty analysis.	4-5
14	State how analytic or statistical source code used to generate estimates can be accessed.	4-5 & 13
**Results and Discussion**		
15	Provide published estimates in a file format from which data can be efficiently extracted.	13
16	Report a quantitative measure of the uncertainty of the estimates (e.g. uncertainty intervals).	6-9
17	Interpret results in light of existing evidence. If updating a previous set of estimates, describe the reasons for changes in estimates.	9-12
18	Discuss limitations of the estimates. Include a discussion of any modelling assumptions or data limitations that affect interpretation of the estimates.	12

This checklist should be used in conjunction with the GATHER statement and Explanation and Elaboration document, found on gather-statement.org

GBD 2019 Adolescent Transport and Unintentional Injuries Collaborators – Authors and Affiliations

Amy E Peden, Patricia Cullen, Kate Louise Francis, Holger Moeller, Margaret M Peden, Pengpeng Ye, Maoyi Tian, Zhiyong Zou, Susan M Sawyer, Amirali Aali, Zeinab Abbasi-Kangevari, Mohsen Abbasi-Kangevari, Michael Abdelmasseh, Meriem Abdoun, Rami Abd-Rabu, Deldar Morad Abdulah, Ame Mehadi Abdurehman, Getachew Abebe, Ayele Mamo Abebe, Aidin Abedi, Hassan Abidi, Richard Gyan Aboagye, Hiwa Abubaker Ali, Eman Abu-Gharbieh, Denberu Eshetie Adane, Tigist Demssew Adane, Isaac Yeboah Addo, Ololade Grace Adewole, Sangeet Adhikari, Mohammad Adnan, Qorinah Estiningtyas Sakilah Adnani, Aanuoluwapo Adeyimika Bachelor Afolabi, Saira Afzal, Muhammad Sohail Afzal, Zahra Babaei Aghdam, Bright Opoku Ahinkorah, Araz Ramazan Ahmad, Tauseef Ahmad, Sajjad Ahmad, Ali Ahmadi, Haroon Ahmed, Muktar Beshir Ahmed, Ali Ahmed, Ayman Ahmed, Jivan Qasim Ahmed, Tarik Ahmed Rashid, Janardhana P Aithala, Budi Aji, Meisam Akhlaghdoust, Fares Alahdab, Fahad Mashhour Alanezi, Astawus Alemayehu, Hanadi Al Hamad, Syed Shujait Ali, Liaqat Ali, Yousef Alimohamadi, Vahid Alipour, Syed Mohamed Aljunid, Louay Almidani, Sami Almustanyir, Khalid A Altirkawi, Nelson J Alvis-Zakzuk, Edward Kwabena Ameyaw, Tarek Tawfik Amin, Mehrdad Amir-Behghadami, Sohrab Amiri, Hoda Amiri, Tadele Fentabil Anagaw, Tudorel Andrei, Catalina Liliana Andrei, Davood Anvari, Sumadi Lukman Anwar, Anayochukwu Edward Anyasodor, Jalal Arabloo, Morteza Arab-Zozani, Asrat Arja, Judie Arulappan, Ashokan Arumugam, Armin Aryannejad, Saeed Asgary, Tahira Ashraf, Seyyed Shamsadin Athari, Alok Atreya, Sameh Attia, Avinash Aujayeb, Atalel Fentahun Awedew, Sina Azadnajafabad, Mohammadreza Azangou-Khyavy, Samad Azari, Amirhossein Azari Jafari, Hosein Azizi, Ahmed Y Azzam, Ashish D Badiye, Nayereh Baghcheghi, Sara Bagherieh, Atif Amin Baig, Shankar M Bakkannavar, Asaminew Birhanu Balta, Maciej Banach, Palash Chandra Banik, Hansi Bansal, Mainak Bardhan, Francesco Barone-Adesi, Amadou Barrow, Azadeh Bashiri, Pritish Baskaran, Saurav Basu, Nebiyou Simegnew Bayileyegn, Abebe Ayalew Bekel, Alehegn Bekele Bekele, Salaheddine Bendak, Isabela M Bensenor, Alemshet Yirga Berhie, Devidas S Bhagat, Akshaya Srikanth Bhagavathula, Pankaj Bhardwaj, Nikha Bhardwaj, Sonu Bhaskar, Ajay Nagesh Bhat, Krittika Bhattacharyya, Zulfiqar A Bhutta, Sadia Bibi, Bagas Suryo Bintoro, Saeid Bitaraf, Belay Boda Abule Bodicha, Archith Boloor, Souad Bouaoud, Julie Brown, Katrin Burkart, Nadeem Shafique Butt, Muhammad Hammad Butt, Luis Alberto Cámera, Julio Cesar Campuzano Rincon, Chao Cao, Andre F Carvalho, Márcia Carvalho, Promit Ananyo Chakraborty, Eeshwar K Chandrasekar, Jung-Chen Chang, Periklis Charalampous, Jaykaran Charan, Vijay Kumar Chattu, Bitew Mekonnen Chekole, Abdulaal Chitheer, Daniel Youngwhan Cho, Hitesh Chopra, Devasahayam J Christopher, Isaac Sunday Chukwu, Natália Cruz-Martins, Omid Dadras, Saad M A Dahlawi, Xiaochen Dai, Giovanni Damiani, Gary L Darmstadt, Reza Darvishi Cheshmeh Soltani, Aso Mohammad Darwesh, Saswati Das, Anna Dastiridou, Sisay Abebe Debela, Amin Dehghan, Getnet Makasha Demeke, Andreas K Demetriades, Solomon Demissie, Fikadu Nugusu Dessalegn, Abebaw Alemayehu Desta, Mostafa Dianatinasab, Nancy Diao, Diana Dias da Silva, Daniel Diaz, Lankamo Ena Digesa, Mengistie Diress, Shirin Djalalinia, Linh Phuong Doan, Milad Dodangeh, Paul Narh Doku, Deepa Dongarwar, Haneil Larson Dsouza, Ebrahim Eini, Michael Ekholuenetale, Temitope Cyrus Ekundayo, Ahmed Elabbas Mustafa Elagali, Mostafa Ahmed Elbahnasawy, Hala Rashad Elhabashy, Muhammed Elhadi, Maysaa El Sayed Zaki, Daniel Berhanie Enyew, Ryenchindorj Erkhembayar, Sharareh Eskandarieh, Farshid Etaee, Adeniyi Francis Fagbamigbe, Pawan Sirwan Faris, Abbas Farmany, Andre Faro, Farshad Farzadfar, Ali Fatehizadeh, Seyed-Mohammad Fereshtehnejad, Abdullah Hamid Feroze, Getahun Fetensa, Bikila Regassa Feyisa, Irina Filip, Florian Fischer, Behzad Foroutan, Masoud Foroutan, Kayode Raphael Fowobaje, Richard Charles Franklin, Takeshi Fukumoto, Peter Andras Gaal, Muktar A Gadanya, Yaseen Galali, Nasrin Galehdar, Balasankar Ganesan, Tushar Garg, Mesfin Gebrehiwot Damtew Gebrehiwot, Yosef Haile Gebremariam, Yibeltal Yismaw Gela, Urge Gerema, Mansour Ghafourifard, Seyyed-Hadi Ghamari, Reza Ghanbari, Mohammad Ghasemi Nour, Maryam Gholamalizadeh, Ali Gholami, Ali Gholamrezanezhad, Sherief Ghozy, Syed Amir Gilani, Tiffany K Gill, Iago Giné-Vázquez, Zeleke Abate Girma, James C Glasbey, Franklin N Glozah, Mahaveer Golechha, Pouya Goleij, Michal Grivna, Habtamu Alganeh Guadie, Damitha Asanga Gunawardane, Yuming Guo, Veer Bala Gupta, Sapna Gupta, Bhawna Gupta, Vivek Kumar Gupta, Arvin Haj-Mirzaian, Rabih Halwani, Randah R Hamadeh, Sajid Hameed, Lolemo Kelbiso Hanfore, Asif Hanif, Arief Hargono, Netanja I Harlianto, Mehdi Harorani, Ahmed I Hasaballah, S M Mahmudul Hasan, Amr Hassan, Soheil Hassanipour, Hadi Hassankhani, Rasmus J Havmoeller, Simon I Hay, Mohammad Heidari, Delia Hendrie, Demisu Zenbaba Heyi, Yuta Hiraike, Ramesh Holla, Nobuyuki Horita, Sheikh Jamal Hossain, Mohammad Bellal Hossain Hossain, Sedighe Hosseini Shabanan, Mehdi Hosseinzadeh, Sorin Hostiuc, Amir Human Hoveidaei, Alexander Kevin Hsiao, Salman Hussain, Amal Hussein, Segun Emmanuel Ibitoye, Olayinka Stephen Ilesanmi, Irena M Ilic, Milena D

Ilic, Behzad Imani, Mustapha Immurana, Leeberk Raja Inbaraj, Sheikh Mohammed Shariful Islam, Rakibul M Islam, Mohammad Mainul Islam, Nahlah Elkudssiah Ismail, Linda Merin J, Haitham Jahrami, Mihajlo Jakovljevic, Manthan Dilipkumar Janodia, Tahereh Javaheri, Sathish Kumar Jayapal, Umesh Umesh Jayarajah, Sudha Jayaraman, Jayakumar Jeganathan, Bedru Jemal, Ravi Prakash Jha, Jost B Jonas, Tamas Joo, Nitin Joseph, Jacek Jerzy Jozwiak, Mikk Jürisson, Ali Kabir, Vidya Kadashetti, Dler Hussein Kadir, Laleh R Kalankesh, Leila R Kalankesh, Rohollah Kalhor, Vineet Kumar Kamal, Rajesh Kamath, Himal Kandel, Rami S Kantar, Neeti Kapoor, Hassan Karami, Ibraheem M Karaye, Samad Karkhah, Patrick DMC Katoto, Joonas H Kauppila, Gbenga A Kayode, Leila Keikavoosi-Arani, Cumali Keskin, Yousef Saleh Khader, Himanshu Khajuria, Mohammad Khammarnia, Ejaz Ahmad Khan, Md Nuruzzaman Khan, Maseer Khan, Yusra H Khan, Imteyaz A Khan, Abbas Khan, Moien AB Khan, Javad Khanali, Moawiah Mohammad Khatatbeh, Hamid Reza Khayat Kashani, Habibolah Khazaie, Jagdish Khubchandani, Zemene Demelash Kifle, Jihee Kim, Yun Jin Kim, Sezer Kisa, Adnan Kisa, Cameron J Kneib, Farzad Kompani, Hamid Reza Koohestani, Parvaiz A Koul, Sindhura Lakshmi Koulmane Laxminarayana, Ai Koyanagi, Kewal Krishan, Vijay Krishnamoorthy, Burcu Kucuk Bicer, Nithin Kumar, Naveen Kumar, Narinder Kumar, Manasi Kumar, Om P Kurmi, Lucie Laflamme, Judit Lám, Iván Landires, Bagher Larijani, Savita Lasrado, Paolo Lauriola, Carlo La Vecchia, Shaun Wen Huey Lee, Yo Han Lee, Sang-woong Lee, Wei-Chen Lee, Samson Mideksa Legesse, Shanshan Li, Stephen S Lim, László Lorenzovici, Amana Ogeto Luke, Farzan Madadizadeh, Áurea M Madureira-Carvalho, Muhammed Magdy Abd El Razek, Soleiman Mahjoub, Ata Mahmoodpoor, Razzagh Mahmoudi, Marzieh Mahmoudimanesh, Azeem Majeed, Alaa Makki, Elaheh Malakan Rad, Mohammad-Reza Malekpour, Ahmad Azam Malik, Tauqeer Hussain Mallhi, Deborah Carvalho Malta, Borhan Mansouri, Mohammad Ali Mansournia, Elezebeth Mathews, Sazan Qadir Maulud, Dennis Mazingi, Entezar Mehrabi Nasab, Oliver Mendoza-Cano, Ritesh G Menezes, Dechasa Adare Mengistu, Alexios-Fotios A Mentis, Atte Meretoja, Mohamed Kamal Mesregah, Tomislav Mestrovic, Ana Carolina Micheletti Gomide Nogueira de Sá, Ted R Miller, Seyed Peyman Mirghaderi, Andreea Mirica, Seyyedmohammadsadeq Mirmoeeni, Erkin M Mirrakhimov, Moonis Mirza, Sanjeev Misra, Prasanna Mithra, Chaitanya Mittal, Madeline E Moberg, Mokhtar Mohammadi, Soheil Mohammadi, Esmaeil Mohammadi, Reza Mohammadpourhodki, Shafiu Mohammed, Teroj Abdulrahman Mohammed, Mohammad Mohseni, Ali H Mokdad, Sara Momtazmanesh, Lorenzo Monasta, Mohammad Ali Moni, Rafael Silveira Moreira, Shane Douglas Morrison, Ebrahim Mostafavi, Haleh Mousavi Isfahani, Sumaira Mubarik, Lorenzo Muccioli, Soumyadeep Mukherjee, Francesk Mulita, Ghulam Mustafa, Ahamarshan Jayaraman Nagarajan, Mukhammad David Naimzada, Vinay Nangia, Hasan Nassereldine, Zuhair S Natto, Biswa Prakash Nayak, Ionut Negoi, Seyed Aria Nejadghaderi, Samata Nepal, Sandhya Neupane Kandel, Nafise Noroozi, Virginia Nuñez-Samudio, Ogochukwu Janet Nzoputam, Chimezie Igwegbe Nzoputam, Chimedsuren Ochir, Julius Nyerere Odhiambo, Oluwakemi Ololade Odukoya, Hassan Okati-Aliabad, Osaretin Christabel Okonji, Andrew T Olagunju, Ahmed Omar Bali, Emad Omer, Adrian Otoiu, Stanislav S Otstavnov, Nikita Otstavnov, Bilcha Oumer, Mayowa O Owolabi, Mahesh P A, Alicia Padron-Monedero, Jagadish Rao Padubidri, Mohammad Taha Pahlevan Fallahy, Songhomitra Panda-Jonas, Seithikurippu R Pandi-Perumal, Shahina Pardhan, Eun-Kee Park, Sangram Kishor Patel, Aslam Ramjan Pathan, Siddhartha Pati, Uttam Paudel, Shrikant Pawar, Paolo Pedersini, Mario F P Peres, Ionela-Roxana Petcu, Michael R Phillips, Julian David Pillay, Zahra Zahid Piracha, Mohsen Poursadeqiyan, Naeimeh Pourtaheri, Ibrahim Qattea, Amir Radfar, Ata Rafiee, Pankaja Raghav Raghav, Fakher Rahim, Muhammad Aziz Rahman, Firman Suryadi Rahman, Mosiur Rahman, Amir Masoud Rahmani, Shayan Rahmani, Sheetal Raj Moolambally, Sheena Ramazanu, Kiana Ramezanzadeh, Juwel Rana, Saleem Muhammad Rana, Chythra R Rao, Sowmya J Rao, Vahid Rashedi, Mohammad-Mahdi Rashidi, Prateek Rastogi, Azad Rasul, Salman Rawaf, David Laith Rawaf, Lal Rawal, Reza Rawassizadeh, Negar Rezaei, Nazila Rezaei, Mohsen Rezaeian, Aziz Rezapour, Abanoub Riad, Muhammad Riaz, Jennifer Rickard, Jefferson Antonio Buendia Rodriguez, Leonardo Roever, Luca Ronfani, Bedanta Roy, Manjula S, Aly M A Saad, Siamak Sabour, Leila Sabzmakan, Basema Saddik, Malihe Sadeghi, Mohammad Reza Saeb, Umar Saeed, Sahar Saeedi Moghaddam, Sher Zaman Safi, Biniyam Sahiledengle, Harihar Sahoo, Mohammad Ali Sahraian, Morteza Saki, Payman Salamati, Sana Salehi, Marwa Rashad Salem, Abdallah M Samy, Juan Sanabria, Milena M Santric-Milicevic, Muhammad Arif Nadeem Saqib, Yaser Sarikhani, Arash Sarveazad, Brijesh Sathian, Maheswar Satpathy, Ganesh Kumar Saya, Ione Jayce Ceola Schneider, David C Schwebel, Hamed Seddighi, Subramanian Senthilkumaran, Allen Seylani, Hosein Shabaninejad, Melika Shafeghat, Pritik A Shah, Saeed Shahabi, Ataollah Shahbandi, Fariba Shahraki-Sanavi, Masood Ali Shaikh, Elaheh Shaker, Mehran Shams-Beyranvand, Mohd Shanawaz, Mohammed Shannawaz, Mequannent Melaku Sharew Sharew, Neeraj Sharma, Bereket Beyene Shashamo, Maryam Shayan, Rahim Ali Sheikhi, Jiabin Shen,

B Suresh Kumar Shetty, Pavanchand H Shetty, Jae Il Shin, Nebiyu Aniley Shitaye, K M Shivakumar, Parnian Shobeiri, Seyed Afshin Shorofi, Sunil Shrestha, Soraya Siabani, Negussie Boti Sidemo, Wudneh Simegn, Ehsan Sinaei, Paramdeep Singh, Robert Sinto, Md Shahjahan Siraj, Valentin Yurievich Skryabin, Anna Aleksandrovna Skryabina, David A Sleet, Chandan S N, Bogdan Socea, Marco Solmi, Yonatan Solomon, Yi Song, Raúl A R C Sousa, Ireneous N Soyiri, Mark A Stokes, Muhammad Suleman, Rizwan Suliankatchi Abdulkader, Jing Sun, Rafael Tabarés-Seisdedos, Seyyed Mohammad Tabatabaei, Mohammad Tabish, Ensiyeh Taheri, Moslem Taheri Soodejani, Mircea Tampa, Ker-Kan Tan, Ingan Ukur Tarigan, Md Tariqujjaman, Nathan Y Tat, Vivian Y Tat, Arash Tavakoli, Belay Negash Tefera, Yibekal Manaye Tefera, Gebremaryam Temesgen, Mohamad-Hani Temsah, Pugazhenthan Thangaraju, Rekha Thapar, Nikhil Kenny Thomas, Jansje Henny Vera Ticoalu, Marius Belmondo Tincho, Amir Tiyuri, Munkhsaikhan Togtmol, Marcos Roberto Tovani-Palone, Mai Thi Ngoc Tran, Sana Ullah, Saif Ullah, Irfan Ullah, Srikanth Umakanthan, Bhaskaran Unnikrishnan, Era Upadhyay, Sahel Valadan Tahbaz, Pascual R Valdez, Tommi Juhani Vasankari, Siavash Vaziri, Massimiliano Veroux, Dominique Vervoort, Francesco S Violante, Vasily Vlassov, Linh Gia Vu, Yasir Waheed, Yanzhong Wang, Yuan-Pang Wang, Cong Wang, Taweewat Wiangkham, Nuwan Darshana Wickramasinghe, Abay Tadesse Woday, Ai-Min Wu, Gahin Abdulraheem Tayib Yahya, Seyed Hossein Yahyazadeh Jabbari, Lin Yang, Sanni Yaya, Arzu Yigit, Vahit Yiğit, Eshetu Yisihak, Naohiro Yonemoto, Yuyi You, Mustafa Z Younis, Chuanhua Yu, Ismaeel Yunusa, Hossein Yusefi, Mazyar Zahir, Sojib Bin Zaman, Iman Zare, Kourosh Zarea, Mikhail Sergeevich Zastrozhin, Zhi-Jiang Zhang, Yunquan Zhang, Arash Ziapour, Sanjay Zodpey, Mohammad Zoladl, George C Patton*, and Rebecca Q Ivers*.

*Jointly supervised the work

Affiliations

School of Public Health and Community Medicine (A E Peden PhD, Prof R Q Ivers PhD), Population Health Department (P Cullen PhD), School of Population Health (H Moeller PhD), The George Institute for Global Health (P Ye MPH, Prof M Tian PhD), Centre for Social Research in Health (I Y Addo PhD), University of New South Wales, Sydney, NSW, Australia; School of Public Health, Medical, and Veterinary Sciences (A E Peden PhD), James Cook University, Townsville, NSW, Australia; Global Women's Health Division (P Cullen PhD), Injury Division (H Moeller PhD), Department of Injury (J Brown PhD), The George Institute for Global Health, Newtown, NSW, Australia; Centre for Adolescent Health (K L Francis MBiostat), Murdoch Childrens Research Institute, Melbourne, VIC, Australia; School of Public Health (M M Peden PhD), Department of Primary Care and Public Health (Prof A Majeed MD, Prof S Rawaf MD), WHO Collaborating Centre for Public Health Education and Training (D L Rawaf MD), Imperial College London, London, UK; TGI-UK (M M Peden PhD), The George Institute for Global Health, London, UK; National Center for Chronic and Noncommunicable Disease Control and Prevention (P Ye MPH), Chinese Center for Disease Control and Prevention, Beijing, China; School of Public Health (Prof M Tian PhD), Harbin Medical University, Harbin, China; Institute of Child and Adolescent Health (Z Zou MD, Y Song PhD), Peking University, Beijing, China; Department of Paediatrics (Prof S M Sawyer MD), University of Melbourne, Parkville, VIC, Australia; Centre for Adolescent Health (Prof S M Sawyer MD), Murdoch Children's Research Institute, Parkville, VIC, Australia; Faculty of Medicine (A Aali MD), E-Learning Center (M Ghasemi Nour MD), Department of Nursing (R Mohammadpourhodki PhD), Department of Medical Informatics (S Tabatabaei PhD), Clinial Research Development Unit (S Tabatabaei PhD), Mashhad University of Medical Sciences, Mashhad, Iran; Health Policy Research Center (A Aali MD, Y Sarikhani PhD, S Shahabi PhD), Health Information Management Deparatment (A Bashiri PhD), Department of Epidemiology (M Dianatinasab MSc), Department of Physical Therapy (E Sinaei MSc), Shiraz University of Medical Sciences, Shiraz, Iran; Social Determinants of Health Research Center (Z Abbasi-Kangevari BSc, M Abbasi-Kangevari MD, M Azangou-Khyavy MD, S Ghamari MD, J Khanali MD, M Rashidi MD), Department of Epidemiology (A Ahmadi PhD, S Sabour PhD), Functional Neurosurgery Research Center and Shohada Tajrish Comprehensive Neurosurgical Center of Excellence (M Akhlaghdoust MD), Research Institute of Dental Sciences (Prof S Asgary MSc), Cancer Research Center (M Gholamalizadeh PhD), Department of Pharmacology (A Haj-Mirzaian MD, K Ramezanzadeh PharmD), Obesity Research Center (A Haj-Mirzaian MD), Department of Neurosurgery (H Khayat Kashani MD), School of Medicine (S Nejadghaderi MD), Student Research Committee (S Rahmani MD), Ophthalmic Research Center (ORC) (M Shayan MD), Shahid Beheshti University of Medical Sciences, Tehran, Iran; Non-Communicable Diseases Research Center (Z

Abbasi-Kangevari BSc, A Aryannejad MD, S Azadnajafabad MD, M Azangou-Khyavy MD, Prof F Farzadfar DSc, S Ghamari MD, J Khanali MD, M Malekpour MD, S Momtazmanesh MD, S Rahmani MD, M Rashidi MD, N Rezaei PhD, N Rezaei MD, S Saeedi Moghaddam MSc), Department of Epidemiology and Biostatistics (Y Alimohamadi PhD, H Azizi PhD, M Mansournia PhD), Experimental Medicine Research Center (A Aryannejad MD), Multiple Sclerosis Research Center (S Eskandarieh PhD, Prof M Sahraian MD), School of Medicine (S Hosseini Shabanan MD, S Mohammadi MD, M Shafeghat MD), Sports Medicine Research Center (A Hoveidaei MD), Children's Medical Center (F Kompani MD), Endocrinology and Metabolism Research Institute (Prof B Larijani FACE, N Rezaei PhD), Department of Pediatrics (Prof E Malakan Rad MD), Students' Scientific Research Center (SSRC) (S Mirghaderi MD), Faculty of Medicine (E Mohammadi MD, E Shaker MD, P Shobeiri MD), Department of Pharmacology (N Noroozi DVM, M Shayan MD, M Zahir MD), Department of Medicine (M Pahlevan Fallahy MD, A Shahbandi), Metabolomics and Genomics Research Center (F Rahim PhD), Sina Trauma and Surgery Research Center (Prof P Salamati MD), Tehran University of Medical Sciences, Tehran, Iran; Department of Surgery (M Abdelmasseh MD, Prof J Sanabria MD), Marshall University, Huntington, WV, USA; Department of Medicine (Prof M Abdoun BMedSc), University of Setif Algeria, Sétif, Algeria; Evidence-Based Practice Center (R Abd-Rabu MD), Department of Radiology (S Ghozy MD), Mayo Clinic, Rochester, MN, USA; Community and Maternity Nursing Unit (D M Abdulah MPH), Department of Pathology and Microbiology (J Q Ahmed MSc), Dental Basic Sciences Department (T A Mohammed MSc), Department of pathology and microbiology (G A Yahya MSc), University of Duhok, Duhok, Iraq; Department of Medical Anatomy (G Abebe MSc, A B Bekele MSc), Department of Clinical Anatomy (A B Balta MSc), Biomedical Sciences Department (B B A Bodicha MSc), Department of Maternal and Reproductive Health (B M Chekole MSc), Department of Anatomy (S Demissie MSc), Department of Comprehensive Nursing (L E Digesa MSc), Department of Public health (Y H Gebremariam MPH, Z A Girma MPH), Department of Nursing (B B Shashamo MSc), School of Public Health (N B Sidemo MPH), Department of Midwifery (G Temesgen MSc, E Yisihak MSc), Arba Minch University, Arba Minch, Ethiopia; Pediatrics Nursing Department (A M Abebe MSc), Debre Berhan University, Debre Berhan, Ethiopia; Department of Neurosurgery (A Abedi MD), Keck School of Medicine (A Abedi MD), Department of Radiology (A Gholamrezanezhad MD), Mark and Mary Stevens Neuroimaging and Informatics Institute (S Salehi MD), University of Southern California, Los Angeles, CA, USA; Laboratory Technology Sciences Department (H Abidi PhD), Department of Nursing (M Zoladl PhD), Yasuj University of Medical Sciences, Yasuj, Iran; Department of Family and Community Health (R G Aboagye MPH), University of Health and Allied Sciences, Hohoe, Ghana; University of Human Development (Prof H Abubaker Ali PhD), Department of Information Technology (A M Darwesh PhD), Department of Computer Science (M Hosseinzadeh PhD), Diplomacy and Public Relations Department (A Omar Bali PhD), University of Human Development, Sulaymaniyah, Iraq; Clinical Sciences Department (E Abu-Gharbieh PhD, Prof R Halwani PhD), Department of Physiotherapy (A Arumugam PhD), College of Medicine (Prof R Halwani PhD), Department of Family and Community Medicine and Behavioral Sciences (A Hussein PhD), Mass Communication Department (A Makki PhD), Sharjah Institute for Medical Research (B Saddik PhD), University of Sharjah, Sharjah, United Arab Emirates; Depatment of Anesthesia and Critical Care (D E Adane MSc), Debre Tabor University, Debre Tabor, Ethiopia; Department of Clinical and Psychosocial Epidemiology (T D Adane MSc), University of Groningen, Groningen, Netherlands; Quality and Systems Performance Unit (I Y Addo PhD), Cancer Institute NSW, Sydney, NSW, Australia; Planning Programming and Linkages (O G Adewole PhD), National Centre for Technology Management, Ile-Ife, Nigeria; Department of Demography and Social Statistics (O G Adewole PhD), Obafemi Awolowo University, Ile-Ife, Nigeria; Biodesign Center for Environmental Health Engineering (S Adhikari MS), Arizona State University, Tempe, AZ, USA; Department of Neonatology (M Adnan MD), Indiana University Health Ball Memorial Hospital, Muncie, IN, USA; Faculty of Medicine (Q E S Adnani PhD), Universitas Padjadjaran (Padjadjaran University), Bandung, Indonesia; Department of Community Medicine (A A B Afolabi MPH, O S Ilesanmi PhD), Department of Epidemiology and Medical Statistics (M Ekholuenetale MSc, A F Fagbamigbe PhD, K R Fowobaje MSc), Faculty of Public Health (M Ekholuenetale MSc), Department of Health Promotion and Education (S E Ibitoye MPH), Department of Medicine (Prof M O Owolabi DrM), University of Ibadan, Ibadan, Nigeria; Department of Community Medicine (Prof S Afzal PhD), King Edward Memorial Hospital, Lahore, Pakistan; Department of Public Health (Prof S Afzal PhD), Public Health Institute, Lahore, Pakistan; Department of Life Sciences (M S Afzal PhD, I Ullah PhD), School of Sciences (M N Saqib PhD), University of Management and Technology, Lahore, Pakistan; Medical Imaging Sciences Research Team (Z B Aghdam MD), Road Traffic Injury Research Center (M Amir-Behghadami MSc), Research Center of Psychiatry and

Behavioral Sciences (H Azizi PhD), Department of Medical Surgical Nursing (M Ghafourifard PhD), School of Nursing and Midwifery (H Hassankhani PhD), School of Management and Medical Informatics (L R Kalankesh PhD), Department of Anesthesiology and Critical Care (Prof A Mahmoodpoor MD), Tabriz University of Medical Sciences, Tabriz, Iran; The Australian Centre for Public and Population Health Research (ACPPHR) (B O Ahinkorah MPH, E K Ameyaw MPhil), School of Health (S Siabani PhD), School of Computing Sciences (Prof J Sun PhD), University of Technology Sydney, Sydney, NSW, Australia; College of Nursing (A R Ahmad PhD), International Relations & Diplomacy, Ranya - Al Sulaimaniyah, Iraq; International Relations & Diplomacy (A R Ahmad PhD), Tishk International University, Erbil, Iraq; Department of Epidemiology and Health Statistics (T Ahmad MS), Southeast University, Nanjing, China; Department of Health and Biological Sciences (S Ahmad PhD), Abasyn University, Peshawar, Pakistan; Department of Epidemiology and Biostatistics (A Ahmadi PhD), Community-Oriented Nursing Midwifery Research Center (M Heidari PhD), Shahrekord University of Medical Sciences, Shahrekord, Iran; Department of Biosciences (H Ahmed PhD), COMSATS Institute of Information Technology, Islamabad, Pakistan; Department of Epidemiology (M B Ahmed MPH), Department of Public Health (U Gerema MSc), Jimma University, Jimma, Ethiopia; Australian Center for Precision Health (M B Ahmed MPH), University of South Australia, Adelaide, SA, Australia; School of Pharmacy (A Ahmed MPhil, S W H Lee PhD), Monash University, Bandar Sunway, Malaysia; Department of Pharmacy (A Ahmed MPhil), Quaid I Azam University Islamabad, Islamabad, Pakistan; Institute of Endemic Diseases (A Ahmed MSc), University of Khartoum, Khartoum, Sudan; Department of Epidemiology and Public Health (A Ahmed MSc), University of Basel, Allschwil, Switzerland; Department of Computer Science and Engineering (T Ahmed Rashid PhD), University of Kurdistan Hewler, Erbil, Iraq; Department of Orthopedics (Prof J P Aithala DNB), Yenepoya Medical College, Mangalore, India; Faculty of Medicine and Public Health (B Aji DrPH), Jenderal Soedirman University, Purwokerto, Indonesia; International Federation of Inventors' Associations, Geneva, Swaziland (M Akhlaghdoust MD); Mayo Evidence-based Practice Center (F Alahdab MSc), Mayo Clinic Foundation for Medical Education and Research, Rochester, MN, USA; Environmental Health Department (S M A Dahlawi PhD), Forensic Medicine Division (Prof R G Menezes MD), Imam Abdulrahman Bin Faisal University, Dammam, Saudi Arabia (F M Alanezi PhD); Department of Public Health (A Alemayehu MPH), Harar Health Science College, Harar, Ethiopia; Department of Public Health (A Alemayehu MPH), Rift Valley University, Harar, Ethiopia; Geriatric and Long Term Care Department (H Al Hamad MD, B Sathian PhD), Rumailah Hospital (H Al Hamad MD), Hamad Medical Corporation, Doha, Qatar; Centre for Biotechnology and Microbiology (S S Ali PhD), University of Swat, Pakistan, Swat, Pakistan; Department of Biological Sciences (L Ali PhD), National University of Medical Sciences (NUMS), Rawalpindi, Pakistan; Pars Advanced and Minimally Invasive Medical Manners Research Center (Y Alimohamadi PhD), Health Management and Economics Research Center (V Alipour PhD, J Arabloo PhD, A Rezapour PhD), Department of Health Economics (V Alipour PhD, H Karami PhD), Hospital Management Research Center (S Azari PhD), School of Medicine (M Dodangeh MD), Minimally Invasive Surgery Research Center (A Kabir MD), Health Services Management (H Mousavi Isfahani PhD), Tehran Institute of Psychiatry (V Rashedi PhD), Colorectal Research Center (A Sarveazad PhD), Department of Health Services Management (H Shabaninejad PhD), Department of Epidemiology and Biostatistics (A Tiyuri MSc), Iran University of Medical Sciences, Tehran, Iran; Department of Health Policy and Management (Prof S M Aljunid PhD), Kuwait University, Kuwait, Kuwait; International Centre for Casemix and Clinical Coding (Prof S M Aljunid PhD), National University of Malaysia, Bandar Tun Razak, Malaysia; Nuffield Department of Primary Care Health Sciences (L Almidani MSc), The George Institute for Global Health (Prof S Yaya PhD), University of Oxford, Oxford, UK; Doheny Image Reading and Research Lab (DIRRL) (L Almidani MSc), University of California Los Angeles, Los Angeles, CA, USA; College of Medicine (S Almustanyir MD), Alfaisal University, Riyadh, Saudi Arabia; Ministry of Health, Riyadh, Saudi Arabia (S Almustanyir MD); Pediatric Intensive Care Unit (K A Altirkawi MD, M Temsah MD), King Saud University, Riyadh, Saudi Arabia; Department of Economic Sciences (N J Alvis-Zakzuk MSc), Universidad de la Costa (University of the Coast), Barranquilla, Colombia; National Health Observatory (N J Alvis-Zakzuk MSc), National Institute of Health, Bogota, Colombia; Public Heath Department (Prof T T Amin MD), Neurophysiology Department (Prof H R Elhabashy MD), Neurology Department (A Hassan MD), Cairo University, Cairo, Egypt; Department of Health Service Management (M Amir-Behghadami MSc), Iranian Center of Excellence in Health Management, Tabriz, Iran; Behavioral Sciences Research Center (S Amiri PhD), Baqiyatallah University of Medical Sciences, Tehran, Iran; Department of Environmental Health (H Amiri PhD), Department of Biostatistics and Epidemiology (M Mahmoudimanesh PhD), Kerman University of Medical Sciences, Kerman, Iran; Department of Health Promotion (T F

Anagaw MPH), Department of Biomedical Science (A A Bekel MSc), School of Health Science (A Y Berhie MSc), Department Health Informatics (H A Guadie MPH), College of Medicine and Health Sciences (N A Shitaye MD), Bahir Dar University, Bahir Dar, Ethiopia; Department of Statistics and Econometrics (Prof T Andrei PhD, A Mirica PhD, A Otoiu PhD, I Petcu PhD), Bucharest University of Economic Studies, Bucharest, Romania; Cardiology Department (C Andrei PhD), Department of Legal Medicine and Bioethics (S Hostiuc PhD), Department of General Surgery (I Negoi PhD, B Socea PhD), Department of Dermatology (M Tampa PhD), Carol Davila University of Medicine and Pharmacy, Bucharest, Romania; Department of Parasitology (D Anvari PhD), Department Medical-Surgical Nursing (S Shorofi PhD), Mazandaran University of Medical Sciences, Sari, Iran; Department of Parasitology (D Anvari PhD), Department of Pharmacology (Prof B Foroutan PhD), Iranshahr University of Medical Sciences, Iranshahr, Iran; Department of Surgery (S Anwar PhD), Gadjah Mada University, Yogyakarta, Indonesia; School of Community Health (A E Anyasodor PhD), Charles Sturt University, Orange, NSW, Australia; Social Determinants of Health Research Center (M Arab-Zozani PhD), Department of Epidemiology and Biostatistics (A Tiyuri MSc), Birjand University of Medical Sciences, Birjand, Iran; National Data Management Center for Health (NDMC) (A Arja MPH), Knowledge Translation Directorate (S M Legesse PhD), Ethiopian Public Health Institute, Addis Ababa, Ethiopia; Department of Maternal and Child Health (J Arulappan DSc), Sultan Qaboos University, Muscat, Oman; Department of Community Medicine and Rehabilitation (A Arumugam PhD), Umeå University, Umea, Sweden; National Agency for Strategic Research in Medical Education (NASRME) (Prof S Asgary MSc), Development of Research and Technology Center (S Djalalinia PhD), Ministry of Health and Medical Education, Tehran, Iran; University Institute of Radiological Sciences and Medical Imaging Technology (T Ashraf MS), Faculty of Allied Health Sciences (Prof S Gilani PhD), University Institute of Public Health (S Hameed MPH, A Hanif PhD, A A Malik PhD), The University of Lahore, Lahore, Pakistan; Department of Immunology (S Athari PhD), Zanjan University of Medical Sciences, Zanjan, Iran; Department of Forensic Medicine (A Atreya MD), Lumbini Medical College, Palpa, Nepal; Oral and Maxillofacial Surgery (S Attia MSc), Justus Liebig University of Giessen, Giessen, Germany; Northumbria HealthCare NHS Foundation Trust (A Aujayeb MBBS), National Health Service (NHS) Scotland, Newcastle upon Tyne, UK; Department of Surgery (A F Awedew MD), Addis Ababa University, Addis Ababa, Ethiopia; School of Medicine (A Azari Jafari MD, S Mirmoeeni MD), Health Information Technology (M Sadeghi PhD), Shahroud University of Medical Sciences, Shahroud, Iran; Faculty of Medicine (A Y Azzam MBBCh), October 6 University, 6th October City, Egypt; Department of Forensic Science (A D Badiye MSc, N Kapoor MSc), Government Institute of Forensic Science, Nagpur, India; Department of Nursing (N Baghcheghi PhD), Saveh University of Medical Sciences, saveh, Iran; School of Medicine (S Bagherieh BSc, A Dehghan MD), Department of Environmental Health Engineering (A Fatehizadeh PhD, E Taheri PhD), Health Services Management (M Mohseni PhD), Isfahan University of Medical Sciences, Isfahan, Iran; Unit of Biochemistry (A A Baig PhD), Universiti Sultan Zainal Abidin (Sultan Zainal Abidin University), Kuala Terengganu, Malaysia; Department of Forensic Medicine and Toxicology (S M Bakkannavar MD), Kasturba Medical College, Mangalore (R Holla MD), Manipal College of Pharmaceutical Sciences (Prof M D Janodia PhD), Prasanna School of Public health (R Kamath MHA), Department of Community Medicine (C R Rao MD), Manipal Academy of Higher Education, Manipal, India; Department of Hypertension (Prof M Banach PhD), Medical University of Lodz, Lodz, Poland; Polish Mothers' Memorial Hospital Research Institute, Lodz, Poland (Prof M Banach PhD); Department of Non-communicable Diseases (P C Banik MPhil), Bangladesh University of Health Sciences, Dhaka, Bangladesh; Department of Forensic Science (H Bansal MSc), Rashtrasant Tukadoji Maharaj Nagpur University, Nagpur, India; Department of Molecular Microbiology and Bacteriology (M Bardhan MD), National Institute of Cholera and Enteric Diseases, Kolkata, India; Department of Molecular Microbiology (M Bardhan MD), Department of Biostatistics (V K Kamal PhD), Indian Council of Medical Research, New Delhi, India; Department of Translational Medicine (F Barone-Adesi PhD), University of Eastern Piedmont, Novara, Italy; Department of Public & Environmental Health (A Barrow MPH), University of The Gambia, Brikama, The Gambia; Epidemiology and Disease Control Unit (A Barrow MPH), Ministry of Health, Kotu, The Gambia; Department of Community Medicine and Family Medicine (P Baskaran MD, P Bhardwaj MD, Prof P R Raghav MD), School of Public Health (P Bhardwaj MD), Department of Anatomy (Prof N Bhardwaj MD), Department of Pharmacology (J Charan MD), Department of Surgical Oncology (Prof S Misra MCh), All India Institute of Medical Sciences, Jodhpur, India; Academics Department (S Basu MD), Indian Institute of Public Health, Gurgaon, India; Department of Surgery (N S Bayileyegn MD), Jimma University, jimma, Ethiopia; Department of Industrial Engineering (S Bendak PhD), Haliç University, Istanbul, Turkey; Department of Internal Medicine (I M Bensenor PhD),

Department of Psychiatry (Prof M F P Peres MD, Y Wang PhD), University of São Paulo, São Paulo, Brazil; Department of Forensic Chemistry (D S Bhagat PhD), Government Institute of Forensic Science, Aurangabad, Aurangabad, India; Department of Social and Clinical Pharmacy (A S Bhagavathula PharmD), Charles University, Hradec Kralova, Czech Republic; Institute of Public Health (A S Bhagavathula PharmD), College of Medicine and Health Sciences (Prof M Grivna PhD), Family Medicine Department (M A Khan MSc), United Arab Emirates University, Al Ain, United Arab Emirates; Neurovascular Imaging Laboratory (S Bhaskar PhD), NSW Brain Clot Bank, Sydney, NSW, Australia; Department of Neurology and Neurophysiology (S Bhaskar PhD), South West Sydney Local Heath District and Liverpool Hospital, Sydney, NSW, Australia; Department of General Medicine (A N Bhat MD, J Jeganathan MD), Department of Internal Medicine (A Boloor MD), Department of Forensic Medicine and Toxicology (H L Dsouza MD, Prof P Rastogi MD, Prof B K Shetty MD), Department of Community Medicine (N Joseph MD, N Kumar MD, P Mithra MD, R Thapar MD), Department of Medicine (S Raj Moolambally MD), Department of Forensic Medicine (P H Shetty MD), Kasturba Medical College (Prof B Unnikrishnan MD), Manipal Academy of Higher Education, Mangalore, India; Department of Statistical and Computational Genomics (K Bhattacharyya MSc), National Institute of Biomedical Genomics, Kalyani, India; Department of Statistics (K Bhattacharyya MSc), University of Calcutta, Kolkata, India; Centre for Global Child Health (Prof Z A Bhutta PhD), University of Toronto, Toronto, ON, Canada; Centre of Excellence in Women & Child Health (Prof Z A Bhutta PhD), Aga Khan University, Karachi, Pakistan; Institute of Soil and Environmental Sciences (S Bibi PhD, S Ullah PhD), University of Agriculture, Faisalabad, Faisalabad, Pakistan; Department of Health Behaviour, Environment and Social Medicine (B Bintoro MD), Center of Health and Behavior and Promotion (B Bintoro MD), Universitas Gadjah Mada (Gadjah Mada University), Sleman, Indonesia; Department of Biostatistics and Epidemiology (Prof S Bitaraf PhD), Department of Orthodontics (E Eini DDS), Department of Microbiology (M Saki PhD), Nursing Care Research Center in Chronic Diseases (K Zarea PhD), Ahvaz Jundishapur University of Medical Sciences, Ahvaz, Iran; Department of Medicine (Prof S Bouaoud MD), Faculty of Medicine University Farhat Abbas, Setif, Algeria; Department of Epidemiology and Preventive Medicine (Prof S Bouaoud MD), University Hospital Saadna Abdenour, Setif, Algeria; Faculty of Medicine (J Brown PhD), University of New South Wales, Kensington, NSW, Australia; Institute for Health Metrics and Evaluation (K Burkart PhD, X Dai PhD, Prof S I Hay FMedSci, Prof S S Lim PhD, T Mestrovic PhD, M E Moberg BS, A H Mokdad PhD, H Nassereldine MD), Department of Health Metrics Sciences, School of Medicine (K Burkart PhD, X Dai PhD, Prof S I Hay FMedSci, Prof S S Lim PhD, A H Mokdad PhD), Department of Neurological Surgery (A H Feroze MD), Division of Plastic Surgery (C J Kneib MD), Department of Anesthesiology & Pain Medicine (V Krishnamoorthy MD), Division of Plastic and Reconstructive Surgery (S D Morrison MD), University of Washington, Seattle, WA, USA; Department of Family and Community Medicine (N S Butt PhD), Rabigh Faculty of Medicine (A A Malik PhD), Department of Dental Public Health (Z S Natto DrPH), King Abdulaziz University, Jeddah, Saudi Arabia; Faculty of Pharmacy (M Butt MS), University of Central Punjab, Lahore, Pakistan; Internal Medicine Department (Prof L A Cámera MD), Hospital Italiano de Buenos Aires (Italian Hospital of Buenos Aires), Buenos Aires, Argentina; Board of Directors (Prof L A Cámera MD), Argentine Society of Medicine, Buenos Aires, Argentina (Prof P R Valdez Med); National Institute of Public Health, Cuernavaca, Mexico (J Campuzano Rincon PhD); School of Medicine (J Campuzano Rincon PhD), University of the Valley of Cuernavaca, Cuernavaca, Mexico; Program in Physical Therapy (C Cao MPH), Washington University in St. Louis, St. Louis, MO, USA; IMPACT Strategic Research Center (the Institute for Mental and Physical Health and Clinical Translation) (A F Carvalho MD), School of Medicine (V Gupta PhD), Deakin University, Geelong, VIC, Australia; Faculty of Health Sciences (M Carvalho PhD), University Fernando Pessoa, Porto, Portugal; Research Unit on Applied Molecular Biosciences (UCIBIO) (M Carvalho PhD), Department of Medicine (Prof N Cruz-Martins PhD), Laboratory of Toxicology (Prof D Dias da Silva PhD), Associated Laboratory for Green Chemistry (LAQV) (Á M Madureira-Carvalho PhD), University of Porto, Porto, Portugal; School of Population and Public Health (P A Chakraborty MPH), University of British Columbia, Vancouver, BC, Canada; Department of Anesthesiology and Perioperative Medicine (E K Chandrasekar MD), University of Rochester, Rochester, NY, USA; College of Medicine (J Chang PhD), National Taiwan University, Taipei, Taiwan; Department of Nursing (J Chang PhD), National Taiwan University Hospital, Taipei, Taiwan; Public Health Epidemiology (P Charalampous MSc), Erasmus University Medical Center, Rotterdam, Netherlands; Department of Community Medicine (V Chattu MD), Datta Meghe Institute of Medical Sciences, Sawangi, India; Saveetha Medical College (V Chattu MD), Saveetha Medical College and Hospitals (S R Pandi-Perumal MSc), Saveetha University, Chennai, India; Iraq Field Epidemiology Training Program (I-FETP) (A Chitheer MD),

Ministry of Health, Baghdad, Iraq; Division of Plastic Surgery (D Y Cho MD), Children's Hospital of Philadelphia, Philadelphia, PA, USA; Department of Pharmacy (H Chopra MPH), Chitkara University, Rajpura, India; Department of Pulmonary Medicine (Prof D J Christopher MD), Christian Medical College and Hospital (CMC), Vellore, India; Department of Surgery (I S Chukwu BMedSc), Federal Medical Centre, Umuahia, Nigeria; Health Sciences Department (Prof N Cruz-Martins PhD), Institute of Research and Advanced Training in Health Sciences and Technologies (CESPU), Famalicão, Portugal; School of Public Health (O Dadras DrPH), Walailak University, Thai Buri, Thailand; Department of Global Public Health and Primary Care (O Dadras DrPH), University of Bergen, Bergen, Norway; IRCCS Istituto Ortopedico Galeazzi (Galeazzi Orthopedic Institute IRCCS) (G Damiani MD), Department of Clinical Sciences and Community Health (Prof C La Vecchia MD), University of Milan, Milan, Italy; Department of Dermatology (G Damiani MD), Department of Neonatology (I Qattea MD), Department of Nutrition and Preventive Medicine (Prof J Sanabria MD), Case Western Reserve University, Cleveland, OH, USA; Department of Pediatrics (Prof G L Darmstadt MD), Stanford University, Stanford, CA, USA; Department of Environmental Health (R Darvishi Cheshmeh Soltani PhD), Department of Nursing (M Harorani MSc), Arak University of Medical Sciences, Arak, Iran; Department of Biochemistry (S Das MD), Ministry of Health and Welfare, New Delhi, India; 2nd University Ophthalmology Department (A Dastiridou MD), Aristotle University of Thessaloniki, Thessaloniki, Greece; Ophthalmology Department (A Dastiridou MD), Medical School (F Mulita MD), University of Thessaly, Larissa, Greece; School of Public Health (S Debela MPH), Salale University, Fiche, Ethiopia; College of Health Sciences (G M Demeke MPH), Debre Berhan University, Debere Berhan, Ethiopia; Department of Public Health (G M Demeke MPH), University of South Africa, Tswane, South Africa; Department of Neurosurgery (A K Demetriades MD), University of Edinburgh, Edinburgh, UK; Department of Neurosurgery (A K Demetriades MD), National Health Service (NHS) Scotland, Edinburgh, UK; Department of Public Health (F N Dessalegn MPH, D Z Heyi MPH, B Sahiledengle MPH), Madda Walabu University, Robe, Ethiopia; Department of Surgical Nursing (A A Desta MSc), Department of Human Physiology (M Diress MSc, Y Gela MSc), Department of Pharmacology (Z D Kifle MSc), Institute of Public Health (M M S Sharew MPH), Department of Social and Administrative Pharmacy (W Simegn MSc), University of Gondar, Gondar, Ethiopia; Department of Epidemiology (M Dianatinasab MSc), Care and Public Health Research Institute (CAPHRI) (R Kamath MHA), Maastricht University, Maastricht, Netherlands; Department of Environmental Health (N Diao DSc), Department of Health Policy and Oral Epidemiology (Z S Natto DrPH), Harvard University, Boston, MA, USA; Center of Complexity Sciences (Prof D Diaz PhD), National Autonomous University of Mexico, Mexico City, Mexico; Faculty of Veterinary Medicine and Zootechnics (Prof D Diaz PhD), Autonomous University of Sinaloa, Culiacán Rosales, Mexico; Institute for Global Health Innovations (L P Doan MSc, L G Vu MSc), Faculty of Medicine (L P Doan MSc, L G Vu MSc), Institute of Research and Development (M Hosseinzadeh PhD), Duy Tan University, Da Nang, Vietnam; School of Nursing and Midwifery (P N Doku PhD), University of Cape Coast, Cape Coast, Ghana; Center of Excellence in Health Equity, Training and Research (D Dongarwar MS), Baylor College of Medicine, Houston, TX, USA; Department of Forensic Medicine and Toxicology (H L Dsouza MD), Kasturba Medical College Mangalore, Mangalore, India; Department of Biological Sciences (T C Ekundayo PhD), University of Medical Sciences, Ondo, Ondo, Nigeria; Geospatial Health and Development (A E M Elagali PhD), Telethon Kids Institute, Perth, WA, Australia; Department of Zoology (A E M Elagali PhD), Omdurman Islamic University, Khartoum, Sudan; Microbiology Department (M A Elbahnasawy PhD), Department of Zoology and Entomology (A I Hasaballah PhD), Al Azhar University, Cairo, Egypt; Faculty of Medicine (M Elhadi MD), University of Tripoli, Tripoli, Libya; Department of Clinical Pathology (Prof M El Sayed Zaki PhD), Mansoura University, Mansoura, Egypt; Deppartment of Health informatics (D B Enyew MSc), Department of Environmental Health Science (D Mengistu MSc), Department of Public Health and Health Policy (B N Tefera MPH), Haramaya University, Harar, Ethiopia; Department of International Cyber Education (R Erkhembayar MD, Prof C Ochir PhD), Mongolian National University of Medical Sciences, Ulaanbaatar, Mongolia; Department of Internal Medicine (F Etaee MD), Department of Genetics (S Pawar PhD), Yale University, New Haven, CT, USA; Population and Behavioural Sciences Division (A F Fagbamigbe PhD), University of St Andrews, St Andrews, UK; Department of Biology (P S Faris PhD, S Q Maulud PhD), Food Technology Department (Y Galali ResM), Salahaddin University-Erbil, Erbil, Iraq; Department of Biology (P S Faris PhD), Department of Nutrition and dietetics (Y Galali ResM), Cihan University-Erbil, Erbil, Iraq; Department of Health Sciences (A Farmany PhD), Hamadan University of Medical Sciences, Hamadan, Iran; Department of Psychology (Prof A Faro PhD), Federal University of Sergipe, São Cristóvão, Brazil; Department of Neurobiology (S Fereshtehnejad PhD), Department of Molecular Medicine and

Surgery (J H Kauppila MD), Department of Global Public Health (Prof L Laflamme PhD), Karolinska Institute, Stockholm, Sweden; Division of Neurology (S Fereshtehnejad PhD), Department of Neurosciences (M Solmi MD), School of International Development and Global Studies (Prof S Yaya PhD), University of Ottawa, Ottawa, ON, Canada; Human Biology Division (A H Feroze MD), Fred Hutchinson Cancer Research Center, Seattle, WA, USA; Department of Nursing (G Fetensa MSc), Institute of Health sciences (B R Feyisa MPH), Wollega University, Nekemte, Ethiopia; Psychiatry Department (I Filip MD), Kaiser Permanente, Fontana, CA, USA; School of Health Sciences (I Filip MD), A.T. Still University, Mesa, AZ, USA; Institute of Public Health (F Fischer PhD), Charité Universitätsmedizin Berlin (Charité Medical University Berlin), Berlin, Germany; Department of Medical Parasitology (M Foroutan PhD), Faculty of Medicine (M Foroutan PhD), Abadan University of Medical Sciences, Abadan, Iran; Child Survival Unit (K R Fowobaje MSc), Centre for African Newborn Health and Nutrition, Ibadan, Nigeria; School of Public Health, Medical, and Veterinary Sciences (R C Franklin PhD), James Cook University, Douglas, QLD, Australia; Department of Dermatology (T Fukumoto PhD), Kobe University, Kobe, Japan; Health Services Management Training Centre (P A Gaal PhD, T Joo MSc), Faculty of Health and Public Administration (J Lám PhD), Semmelweis University, Budapest, Hungary; Department of Applied Social Sciences (P A Gaal PhD), Sapientia Hungarian University of Transylvania, Târgu-Mureş, Romania; Community Medicine Department (M A Gadanya FMCPH), Bayero University, Kano, Kano, Nigeria; Department of Community Medicine (M A Gadanya FMCPH), Aminu Kano Teaching Hospital, Kano, Nigeria; Department of Surgical Technology (N Galehdar PhD), Lorestan University of Medical Sciences, Khorramabad, Iran; Neurorehabilitation and Robotics Laboratory (B Ganesan PhD), The Chinese University of Hong Kong, Hong Kong, China; Department of Occupational Therapy (B Ganesan PhD), Mahatma Gandhi Occupational Therapy College, Jaipur, India; Department of Radiology (T Garg MBBS), King Edward Memorial Hospital, Mumbai, India; Department of Environmental Health (M G D Gebrehiwot PhD), Wollo University, Dessie, Ethiopia; Department of Environmental Health Engineering (R Ghanbari PhD), Institute for Prevention of Non-communicable Diseases (R Kalhor PhD), Health Services Management Department (R Kalhor PhD), Department of Food Hygiene and Safety (Prof R Mahmoudi PhD), Qazvin University of Medical Sciences, Qazvin, Iran; Department of Epidemiology and Biostatistics (A Gholami PhD), Non-Communicable Diseases Research Center (A Gholami PhD), Neyshabur University of Medical Sciences, Neyshabur, Iran; Afro-Asian Institute, Lahore, Pakistan (Prof S Gilani PhD); Adelaide Medical School (T K Gill PhD), University of Adelaide, Adelaide, SA, Australia; Research, Innovation and Teaching Unit (I Giné-Vázquez PhD), Biomedical Research Networking Center for Mental Health Network (CIBERSAM) (A Koyanagi MD), San Juan de Dios Sanitary Park, Sant Boi de Llobregat, Spain; Carlos III Health Institute (Prof R Tabarés-Seisdedos PhD), Biomedical Research Networking Center for Mental Health Network (CiberSAM), Madrid, Spain (I Giné-Vázquez PhD); NIHR Global Health Research Unit on Global Surgery (J C Glasbey MSc), University of Birmingham, Birmingham, UK; Department of Social and Behavioural Sciences (F N Glozah PhD), University of Ghana, Accra, Ghana; Health Systems and Policy Research (M Golechha PhD), Indian Institute of Public Health, Gandhinagar, India; Department of Genetics (P Goleij MSc), Sana Institute of Higher Education, Sari, Iran; Department of Public Health and Preventive Medicine (Prof M Grivna PhD), Charles University, Prague, Czech Republic; Department of Community Medicine (D A Gunawardane MD), University of Peradeniya, Kandy, Sri Lanka; Department of Epidemiology and Preventive Medicine (Prof Y Guo PhD, R M Islam PhD), School of Public Health and Preventive Medicine (S Li PhD), The School of Clinical Sciences at Monash Health (S Zaman MPH), Monash University, Melbourne, VIC, Australia; Department of Epidemiology (Prof Y Guo PhD), Binzhou Medical University, Yantai City, China; Toxicology Department (S Gupta MSc), Shriram Institute for Industrial Research, Delhi, Delhi, India; Department of Public Health (B Gupta PhD), Torrens University, Melbourne, VIC, Australia; Department of Clinical Medicine (Prof V K Gupta PhD), Macquarie University, Sydney, NSW, Australia; Department of Family and Community Medicine (Prof R R Hamadeh PhD), College of Medicine and Medical Sciences (H Jahrami PhD), Arabian Gulf University, Manama, Bahrain; Department of Epidemiology (A Hargono DMD), Department of Public Health (F S Rahman DrPH), Universitas Airlangga (Airlangga University), Surabaya, Indonesia; Faculty of Medicine (N I Harlianto BSc), Julius Centre for Health Sciences and Primary Care (G A Kayode PhD), Utrecht University, Utrecht, Netherlands; Department of Radiology (N I Harlianto BSc), University Medical Center Utrecht, Utrecht, Netherlands; Department of Public Health (S Hasan PhD), German University Bangladesh, Dhaka, Bangladesh; Daffodil International University, Dhaka, Bangladesh (S Hasan PhD); Gastrointestinal and Liver Diseases Research Center (S Hassanipour PhD), Caspian Digestive Disease Research Center (S Hassanipour PhD), Department of Medical-Surgical Nursing (S Karkhah MSc), Guilan University of Medical

Sciences, Rasht, Iran; Independent Consultant, Tabriz, Iran (H Hassankhani PhD); Skaane University Hospital (R J Havmoeller PhD), Skaane County Council, Malmoe, Sweden; School of Public Health (D Hendrie PhD, T R Miller PhD), Curtin University, Perth, WA, Australia; Division for Health Service Promotion (Y Hiraike PhD), University of Tokyo, Tokyo, Japan; Department of Pulmonology (N Horita PhD), Yokohama City University, Yokohama, Japan; National Human Genome Research Institute (NHGRI) (N Horita PhD), National Institutes of Health, Bethesda, MD, USA; Maternal and Child Health Division (S J Hossain MPH, M Siraj MSc, S Zaman MPH), Nutrition and Clinical Services Division (M Tariqujjaman MSc), International Centre for Diarrhoeal Disease Research, Bangladesh, Dhaka, Bangladesh; Department of Population Sciences (Prof M B H Hossain PhD, Prof M Islam PhD), University of Dhaka, Dhaka, Bangladesh; Clinical Legal Medicine Department (S Hostiuc PhD), National Institute of Legal Medicine Mina Minovici, Bucharest, Romania; Outpatient Rehabilitation (A K Hsiao DPT), Southcoast Health: Tobey Hospital, Wareham, MA, USA; Czech National Centre for Evidence-Based Healthcare and Knowledge Translation (S Hussain PhD), Institute of Biostatistics and Analyses (S Hussain PhD), Department of Public Health (A Riad DDS), Czech National Centre for Evidence-based Healthcare and Knowledge Translation (A Riad DDS), Masaryk University, Brno, Czech Republic; Department of Community Medicine (O S Ilesanmi PhD), Department of Medicine (Prof M O Owolabi DrM), University College Hospital, Ibadan, Ibadan, Nigeria; Faculty of Medicine (I M Ilic PhD, Prof M M Santric-Milicevic PhD), School of Public Health and Health Management (Prof M M Santric-Milicevic PhD), University of Belgrade, Belgrade, Serbia; Department of Epidemiology (Prof M D Ilic PhD), University of Kragujevac, Kragujevac, Serbia; Institute of Health Research (M Immurana PhD), University of Health and Allied Sciences, Ho, Ghana; Division of Community Health and Family Medicine (L R Inbaraj MD), Bangalore Baptist Hospital, Bangalore, India; Institute for Physical Activity and Nutrition (S Islam PhD), Department of Psychology (M A Stokes PhD), Deakin University, Burwood, VIC, Australia; Sydney Medical School (S Islam PhD), Save Sight Institute (H Kandel PhD, Y You PhD), University of Sydney, Sydney, NSW, Australia; Department of Clinical Pharmacy (Prof N Ismail PhD), MAHSA University, Bandar Saujana Putra, Malaysia; Department of Orthodontics & Dentofacial Orthopedics (L J BDS), Dr. D. Y. Patil University, Pune, India; Ministry of Health, Manama, Bahrain (H Jahrami PhD); Institute of Advanced Manufacturing Technologies (Prof M Jakovljevic PhD), Peter the Great St. Petersburg Polytechnic University, St. Petersburg, Russia; Institute of Comparative Economic Studies (Prof M Jakovljevic PhD), Hosei University, Tokyo, Japan; Health Informatic Lab (T Javaheri PhD), Department of Computer Science (R Rawassizadeh PhD), Boston University, Boston, MA, USA; Centre of Studies and Research (S Jayapal PhD), Ministry of Health, Muscat, Oman; Postgraduate Institute of Medicine (U U Jayarajah MD), University of Colombo, Colombo, Sri Lanka; Department of Surgery (U U Jayarajah MD), National Hospital, Colombo, Sri Lanka; Department of Surgery (Prof S Jayaraman MD), University of Utah, Salt Lake City, VA, USA; Center for Global Surgery (Prof S Jayaraman MD), Department of Family and Preventive Medicine (O O Odukoya MSc), University of Utah, Salt Lake City, UT, USA; Department of Anesthesiology (B Jemal MSc), Dilla University, Dilla, Ethiopia; Department of Community Medicine (R P Jha MSc), Dr. Baba Saheb Ambedkar Medical College & Hospital, Delhi, India; Department of Community Medicine (R P Jha MSc), Banaras Hindu University, Varanasi, India; Institute of Molecular and Clinical Ophthalmology Basel, Basel, Switzerland (Prof J B Jonas MD); Department of Ophthalmology (Prof J B Jonas MD), Heidelberg University, Mannheim, Germany; Department of Family Medicine and Public Health (J J Jozwiak PhD), University of Opole, Opole, Poland; Institute of Family Medicine and Public Health (M Jürisson PhD), University of Tartu, Tartu, Estonia; Department of Oral Pathology and Microbiology, Forensic Odontology (V Kadashetti MDS), Krishna institute of Medical Sciences Deemed to be University, Karad, India; Department of Statistics (D H Kadir PhD), Salahaddin University, Erbil, Iraq; Social Determinants of Health Research Center (L R Kalankesh PhD), Gonanad University of Medical Sciences, Gonabad, Iran; Division of Epidemiology and Biostatistics (V K Kamal PhD), National Institute of Epidemiology, Chennai, India, India; Sydney Eye Hospital (H Kandel PhD), South Eastern Sydney Local Health District, Sydney, NSW, Australia; Department of Surgery (R S Kantar MD), University of Maryland, Baltimore, MD, USA; Cleft Lip and Palate Surgery (R S Kantar MD), Global Smile Foundation, Norwood, MA, USA; Healthcare Network (H Karami PhD), Shiraz University of Medical Sciences, Farashband, Iran; School of Health Professions and Human Services (I M Karaye MD), Hofstra University, Hempstead, NY, USA; Centre for Tropical Diseases and Global Health (P D Katoto PhD), Catholic University of Bukavu, Bukavu, Democratic Republic of the Congo; Department of Global Health (P D Katoto PhD), Stellenbosch University, Cape Town, South Africa; Surgery Research Unit (J H Kauppila MD), University of Oulu, Oulu, Finland; International Research Center of Excellence (G A Kayode PhD), Institute of Human Virology

Nigeria, Abuja, Nigeria; Department of Healthcare Services Management (L Keikavoosi-Arani PhD), Non-Communicable Diseases Research Center (L Sabzmakan PhD), School of Medicine (M Shams-Beyranvand MSc), Alborz University of Medical Sciences, Karaj, Iran; Vocational Higher School of Healthcare Studies (Prof C Keskin PhD), Mardin Artuklu University, Mardin, Turkey; Department of Public Health (Prof Y S Khader PhD), Jordan University of Science and Technology, Irbid, Jordan; Amity Institute of Forensic Sciences (H Khajuria PhD, B P Nayak PhD), Amity University, Noida, India; Health Promotion Research Center (M Khammarnia PhD, H Okati-Aliabad PhD, F Shahraki-Sanavi PhD), Zahedan University of Medical Sciences, Zahedan, Iran; Department of Epidemiology and Biostatistics (E A Khan MPH), Department of Public Health (Z Z Piracha PhD), Health Services Academy, Islamabad, Pakistan; Department of Population Science (M Khan PhD), Jatiya Kabi Kazi Nazrul Islam University, Mymensingh, Bangladesh; Epidemiology Department (M Khan MD), Health Education and Promotion (M Shanawaz MD), Jazan University, Jazan, Saudi Arabia; Clinical Pharmacy (Y H Khan PhD, T Mallhi PhD), Jouf University, Sakaka, Saudi Arabia; Department of Pediatrics (I A Khan MD), Rutgers University, New Brunswick, NJ, USA; Department of Bioinformatics and Biostatistics (A Khan PhD), Shanghai Mental Health Center (Prof M R Phillips MD), Shanghai Jiao Tong University, Shanghai, China; Primary Care Department (M A Khan MSc), NHS North West London, London, UK; Basic Medical Sciences (M M Khatatbeh PhD), Yarmouk University, Irbid, Jordan; Department of Public Health (Prof J Khubchandani PhD), New Mexico State University, Las Cruces, NM, USA; Department of Preventive Medicine (J Kim MSc), Korea University, Seoul, South Korea; School of Traditional Chinese Medicine (Y Kim PhD), Xiamen University Malaysia, Sepang, Malaysia; Department of Nursing and Health Promotion (S Kisa PhD), Oslo Metropolitan University, Oslo, Norway; School of Health Sciences (Prof A Kisa PhD), Kristiania University College, Oslo, Norway; Department of Global Community Health and Behavioral Sciences (Prof A Kisa PhD), Tulane University, New Orleans, LA, USA; Social Determinants of Health Research Center (H Koohestani PhD), Saveh University of Medical Sciences, Saveh, Iran; Department of Internal and Pulmonary Medicine (Prof P A Koul MD), Sheri Kashmir Institute of Medical Sciences, Srinagar, India; Kasturba Medical College, Udupi, India (S Koulmane Laxminarayana MD); Catalan Institution for Research and Advanced Studies (ICREA), Barcelona, Spain (A Koyanagi MD); Department of Anthropology (Prof K Krishan PhD), Panjab University, Chandigarh, India; Department of Anesthesiology (V Krishnamoorthy MD), Duke University, Durham, NC, USA; Faculty of Medicine (B Kucuk Bicer PhD), Gazi University, Ankara, Turkey; Amity Institute of Biotechnology (N Kumar PhD, E Upadhyay PhD), Amity University Rajasthan, Jaipur, India; Department of Orthopaedics (Prof N Kumar MS), Medanta Hospital, Lucknow, India; Department of Psychiatry (M Kumar PhD), University of Nairobi, Nairobi, Kenya; Division of Psychology and Language Sciences (M Kumar PhD), University College London, London, UK; Faculty of Health and Life Sciences (O P Kurmi PhD), Coventry University, Coventry, UK; Department of Medicine (O P Kurmi PhD), Department of Psychiatry and Behavioural Neurosciences (A T Olagunju MD), McMaster University, Hamilton, ON, Canada; Institute for Social and Health Sciences (Prof L Laflamme PhD), University of South Africa, Pretoria, South Africa; NEVES Society for Patient Safety (J Lám PhD), NEVES Society for Patient Safety, Budapest, Hungary; Unit of Genetics and Public Health (Prof I Landires MD), Unit of Microbiology and Public Health (V Nuñez-Samudio PhD), Institute of Medical Sciences, Las Tablas, Panama; Department of Public Health (V Nuñez-Samudio PhD), Ministry of Health, Herrera, Panama (Prof I Landires MD); Department of Otorhinolaryngology (S Lasrado MS), Father Muller Medical College, Mangalore, India; International Society Doctors for the Environment, Arezzo, Italy (P Lauriola MD); School of Pharmacy (S W H Lee PhD), Taylor's University Lakeside Campus, Subang Jaya, Malaysia; Graduate School of Public Health (Y Lee PhD), Ajou University, Suwon-si, South Korea; Pattern Recognition and Machine Learning Lab (Prof S Lee PhD), Gachon University, Seongnam, South Korea; The Office of Health Policy & Legislative Affairs (W Lee PhD), Department of Pathology (V Y Tat BS), University of Texas, Galveston, TX, USA; Department of Health Economics (L Lorenzovici MSc), Syreon Research Romania, Targu Mures, Romania; Department of Doctoral Studies (L Lorenzovici MSc), George Emil Palade University of Medicine, Tirgu Mures, Romania; Department of Public Health (A O Luke MPH), Rift Valley University, Addis Ababa, Ethiopia; Department of Biostatistics and Epidemiology (F Madadizadeh PhD), Yazd University of Medical Sciences, Yazd, Iran; Instituto de Investigação e Formação Avançada em Ciências e Tecnologias da Saúde (Institute for Research and Advanced Training in Health Sciences and Technologies) (Á M Madureira-Carvalho PhD), Instituto Universitário de Ciências da Saúde (University Institute of Health Sciences), Gandra, Portugal; Ophthalmology Department (M Magdy Abd El Razek MSc), Ministry of Health & Population, Aswan, Egypt; Cellular and Molecular Biology Research Center (Prof S Mahjoub PhD), Department of Clinical Biochemistry (Prof S Mahjoub PhD), Babol University of Medical

Sciences, Babol, Iran; Department of Maternal and Child Nursing and Public Health (Prof D C Malta PhD, Prof A C Micheletti Gomide Nogueira de Sá MSc), Federal University of Minas Gerais, Belo Horizonte, Brazil; Substance Abuse Prevention Research Center (B Mansouri PhD), Department of Health Education and Health Promotion (S Siabani PhD, A Ziapour PhD), Department of Infectious Disease (Prof S Vaziri MD), Kermanshah University of Medical Sciences, Kermanshah, Iran; Department of Public Health and Community Medicine (E Mathews PhD), Central University of Kerala, Kasaragod, India; Nuffield Department of Surgical Sciences (D Mazingi MSc), Oxford University, Oxford, UK; Department of Surgical sciences (D Mazingi MSc), University of Zimbabwe, Harare, Zimbabwe; Tehran Heart Center (E Mehrabi Nasab MD), School of Medicine (S Momtazmanesh MD), Tehran, Iran; Civil Engineering Faculty (Prof O Mendoza-Cano PhD), University of Colima, Coquimatlán City, Mexico; University Research Institute (A A Mentis MD), National and Kapodistrian University of Athens, Athens, Greece; Neurology Unit (A Meretoja MD), Helsinki University Hospital, Helsinki, Finland; School of Health Sciences (A Meretoja MD), Department of Paediatrics (Prof G C Patton MD), University of Melbourne, Melbourne, VIC, Australia; Faculty of Medicine (M K Mesregah MD), Menoufia University, Shebin El-Kom, Egypt; University Centre Varazdin (T Mestrovic PhD), University North, Varazdin, Croatia; Pacific Institute for Research & Evaluation, Calverton, MD, USA (T R Miller PhD); Internal Medicine Programme (Prof E M Mirrakhimov PhD), Kyrgyz State Medical Academy, Bishkek, Kyrgyzstan; Department of Atherosclerosis and Coronary Heart Disease (Prof E M Mirrakhimov PhD), National Center of Cardiology and Internal Disease, Bishkek, Kyrgyzstan; Department of Hospital Administration (M Mirza MD), Department of Radiodiagnosis (P Singh MD), All India Institute of Medical Sciences, Bathinda, India; Department of Forensic Medicine and Toxicology (C Mittal MD), Dr. B. C. Roy Multi-Specialty Medical Research Centre, IIT Kharagpur, Kharagpur, India; Department of Information Technology (M Mohammadi PhD), Lebanese French University, Erbil, Iraq; Department of Epidemiology (S Nejadghaderi MD, E Shaker MD), Department of International Studies (P Shobeiri MD), Non-Communicable Diseases Research Center (NCDRC), Tehran, Iran (E Mohammadi MD); Health Systems and Policy Research Unit (S Mohammed PhD), Ahmadu Bello University, Zaria, Nigeria; Department of Health Care Management (S Mohammed PhD), Technical University of Berlin, Berlin, Germany; Health Services Management (M Mohseni PhD), Iran University of Medical Sciences, Iran, Iran; Clinical Epidemiology and Public Health Research Unit (L Monasta DSc, L Ronfani PhD), Burlo Garofolo Institute for Maternal and Child Health, Trieste, Italy; Faculty of Health and Behavioural Sciences (M Moni PhD), The University of Queensland, Brisbane, QLD, Australia; Department of Public Health (Prof R S Moreira PhD), Oswaldo Cruz Foundation, Recife, Brazil; Department of Public Health (Prof R S Moreira PhD), Federal University of Pernambuco, Recife, Brazil; Department of Medicine (E Mostafavi PhD), Stanford Cardiovascular Institute (E Mostafavi PhD), Stanford University, Palo Alto, CA, USA; Department of Epidemiology and Biostatistics (S Mubarik MS, Prof C Yu PhD), School of Medicine (Z Zhang PhD), Wuhan University, Wuhan, China; Department of Biomedical and Neuromotor Sciences (L Muccioli MD), Department of Medical and Surgical Sciences (Prof F S Violante MD), University of Bologna, Bologna, Italy; Health and Physical Education (S Mukherjee PhD), Rhode Island College, Providence, RI, USA; Department of Surgery (F Mulita MD), General University Hospital of Patras, Patras, Greece; Department of Pediatric Medicine (Prof G Mustafa MD), The Children's Hospital & The Institute of Child Health, Multan, Pakistan; Department of Pediatrics & Pediatric Pulmonology (Prof G Mustafa MD), Institute of Mother & Child Care, Multan, Pakistan; Research and Analytics Department (A J Nagarajan MTech), Initiative for Financing Health and Human Development, Chennai, India; Department of Research and Analytics (A J Nagarajan MTech), Bioinsilico Technologies, Chennai, India; Laboratory of Public Health Indicators Analysis and Health Digitalization (M Naimzada MD, S S Otstavnov PhD, N Otstavnov BA), Moscow Institute of Physics and Technology, Dolgoprudny, Russia; Experimental Surgery and Oncology Laboratory (M Naimzada MD), Kursk State Medical University, Kursk, Russia; Suraj Eye Institute, Nagpur, India (V Nangia MD); Department of General Surgery (I Negoi PhD), Emergency Hospital of Bucharest, Bucharest, Romania; Department of Community Medicine (S Nepal MD), Kathmandu University, Palpa, Nepal; Estia Health Blakehurst (S Neupane Kandel BSN), Estia Health, Sydney, NSW, Australia; Department of Physiology (O J Nzoputam PhD), Center of Excellence in Reproductive Health Innovation (CERHI) (C I Nzoputam MPH), University of Benin, Edo, Nigeria; Department of Physiology (O J Nzoputam PhD), Benson Idahosa University, Benin City, Nigeria; Advisory Board (Prof C Ochir PhD), Ministry of Health, Ulaanbaatar, Mongolia; Discipline of Public Health Medicine (J N Odhiambo MSc), University of KwaZulu-Natal, Durban, South Africa; Department of Management Science and Technology (J N Odhiambo MSc), Technical University of Kenya, Nairobi, Kenya; Department of Community Health and Primary Care (O O Odukoya MSc),

University of Lagos, Idi Araba, Nigeria; School of Pharmacy (O C Okonji MSc), University of the Western Cape, Cape Town, South Africa; Department of Psychiatry (A T Olagunju MD), University of Lagos, Lagos, Nigeria; Mass Communication Department (E Omer PhD), Ajman University, Dubai, United Arab Emirates; Department of Project Management (S S Otstavnov PhD), Department of Health Care Administration and Economics (Prof V Vlassov MD), National Research University Higher School of Economics, Moscow, Russia; Department of Midwifery (B Oumer MPH), Arba Minch College of Health Science, Arba Minch, Ethiopia; Department of Respiratory Medicine (Prof M P A DNB), Jagadguru Sri Shivarathreeswara Academy of Health Education and Research, Mysore, India; National School of Public Health (A Padron-Monedero PhD), Institute of Health Carlos III, Madrid, Spain; Department of Forensic Medicine and Toxicology (J Padubidri MD), Kasturba Medical College, Mangalore, Mangalore, India; Privatpraxis, Heidelberg, Germany (S Panda-Jonas MD); Vision and Eye Research Institute (Prof S Pardhan PhD), Anglia Ruskin University, Cambridge, UK; Department of Medical Humanities and Social Medicine (Prof E Park PhD), Kosin University, Busan, South Korea; Department of Poverty, Gender and Youth (S K Patel PhD), Population Council, New Delhi, India; Department of Pharmacology (A R Pathan PhD, M Tabish MPharm), Shaqra University, Shaqra, Saudi Arabia; Research Consultancy (A R Pathan PhD), Author Gate Publications, Malegaon, India; Skills Innovation and Academic Network (SIAN) Institute (S Pati PhD), Association for Biodiversity Conservation and Research (ABC), Odisha, India; Research Section (U Paudel PhD), Nepal Health Research Council, Kathmandu, Nepal; Faculty of Humanities and Social Sciences (U Paudel PhD), Tribhuvan University, Kathmandu, Nepal; Clinical Research Department (P Pedersini MSc), IRCCS Fondazione Don Carlo Gnocchi, Milan, Italy; International Institute for Educational Planning (IIEP) (Prof M F P Peres MD), Albert Einstein Hospital, São Paulo, Brazil; Department of Psychiatry (Prof M R Phillips MD), Columbia University, New York, NY, USA; Basic Medical Sciences Department (J D Pillay PhD), Durban University of Technology, Durban, South Africa; Department of Occupational Health and Safety Engineering (M Poursadeqiyan PhD), Ardabil University of Medical Science, Ardabil, Iran; Non-Communicable Diseases Research Center (N Pourtaheri PhD), Bam University of Medical Sciences, Bam, Iran; College of Medicine (A Radfar MD), University of Central Florida, Orlando, FL, USA; Department of Medicine (A Rafiee MSc), University of Alberta, Edmonton, AB, Canada; School of Nursing and Healthcare Professions (M Rahman PhD), Federation University Australia, Berwick, VIC, Australia; School of Nursing and Midwifery (M Rahman PhD), La Trobe University, Melbourne, VIC, Australia; Department of Population Science and Human Resource Development (M Rahman DrPH), University of Rajshahi, Rajshahi, Bangladesh; Future Technology Research Center (A Rahmani PhD), National Yunlin University of Science and Technology, Yunlin, Taiwan; Alice Lee Centre of Nursing Studies (S Ramazanu PhD), Department of Surgery (K Tan PhD), National University of Singapore, Singapore, Singapore; Department of Epidemiology, Biostatistics and Occupational Health (J Rana MPH), McGill University, Montreal, QC, Canada; Research and Innovation Division (J Rana MPH), South Asian Institute for Social Transformation (SAIST), Dhaka, Bangladesh; Department of Oral Pathology (S Rao MDS), Sharavathi Dental College and Hospital, Shimogga, India; Department of Geography (A Rasul PhD), Soran University, Soran, Iraq; Academic Public Health England (Prof S Rawaf MD), Public Health England, London, UK; University College London Hospitals, London, UK (D L Rawaf MD); School of Health, Medical and Applied Sciences (L Rawal PhD), CQ University, Sydney, NSW, Australia; Department of Epidemiology and Biostatistics (Prof M Rezaeian PhD), Rafsanjan University of Medical Sciences, Rafsanjan, Iran; Department of Pharmacy (M Riaz PhD), Shaheed Benazir Bhutto University Sheringal Pakistan, Dir Upper, Pakistan; Department of Surgery (J Rickard MD), University of Minnesota, Minneapolis, MN, USA; Department of Surgery (J Rickard MD), University Teaching Hospital of Kigali, Kigali, Rwanda; Deparment of Pharmacology and Toxicology (Prof J A B Rodriguez PhD), University of Antioquia, Medellin, Colombia; Department of Clinical Research (L Roever PhD), Federal University of Uberlândia, Uberlândia, Brazil; Faculty of Medicine (B Roy PhD), Quest International University Perak, Ipoh, Malaysia; Oral and Maxillofacial Surgery (M S MDS, C S N PhD), Jagadguru Sri Shivarathreeswara University, Mysore, India; Cardiovascular Department (Prof A M A Saad MD), Zagazig University- Egypt, Zagazig, Egypt; Health Information Management (M Sadeghi PhD), Semnan University of Medical Sciences, Semnan, Iran; Independent Consultant, Gdansk, Poland (Prof M R Saeb PhD); Research and Development (Prof U Saeed PhD), Islamabad Diagnostic Center Pakistan, Islamabad, Pakistan; Biological Production Division (Prof U Saeed PhD), National Institute of Health, Islamabad, Pakistan; Interdisciplinary Research Centre in Biomedical Materials (S Z Safi PhD), COMSATS Institute of Information Technology, Lahore, Pakistan; Department of Development Studies (H Sahoo PhD), International Institute for Population Sciences, Mumbai, India; Public Health and Community Medicine

Department (M R Salem MD), Cairo University, Giza, Egypt; Department of Entomology (A M Samy PhD), Ain Shams University, Cairo, Egypt; Research Development Coordination Section (M N Saqib PhD), Pakistan Health Research Council, Islamabad, Pakistan; Department of Public Health (Y Sarikhani PhD), Jahrom University of Medical Sciences, Jahrom, Iran; Faculty of Health & Social Sciences (B Sathian PhD), Bournemouth University, Bournemouth, UK; UGC Centre of Advanced Study in Psychology (M Satpathy PhD), Utkal University, Bhubaneswar, India; Udyam-Global Association for Sustainable Development, Bhubaneswar, India (M Satpathy PhD); Department of Preventive and Social Medicine (G Saya MD), Jawaharlal Institute of Postgraduate Medical Education and Research, Puducherry, India; Department of Health Sciences (I J C Schneider PhD), Federal University of Santa Catarina, Araranguá, Brazil; Department of Psychology (D C Schwebel PhD), University of Alabama at Birmingham, Birmingham, AL, USA; Department of Governance and Innovation (H Seddighi PhD), University of Groningen, Leeuwarden, Netherlands; Department of Social Welfare (H Seddighi PhD), University of Social Welfare and Rehabilitation Sciences, Tehran, Iran; Emergency Department (S Senthilkumaran MD), Manian Medical Centre, Erode, India; National Heart, Lung, and Blood Institute (A Seylani BS), National Institute of Health, Rockville, MD, USA; Population Health Sciences Institute (H Shabaninejad PhD), Newcastle University, Newcastle Upon Tyne, UK; Infectious Diseases and Microbiology (P A Shah MBBS), Rajiv Gandhi University of Health Sciences, Bangalore, India; Department of HepatoPancreatoBiliary Surgery and Liver Transplant (P A Shah MBBS), HealthCare Global Limited Cancer Care Hospital, Bangalore, India; Independent Consultant, Karachi, Pakistan (M A Shaikh MD); Symbiosis Medical College for Women (M Shannawaz PhD), Symbiosis International University, Pune, India; Department of Public Health (N Sharma MPH), Indian Institute of Health Management Research University, Jaipur, India; Department of Health in Disasters and Emergencies (R Sheikhi BHlthSci), Iran University of Medical Sciences, Shahrekord, Iran; Psychology Department (J Shen PhD), University of Massachusetts Lowell, Boston, MA, USA; College of Medicine (Prof J Shin MD), Yonsei University, Seoul, South Korea; Public Health Dentistry Department (Prof K M Shivakumar PhD), Krishna Institute of Medical Sciences Deemed to be University, Karad, India; Nursing and Health Sciences (S Shorofi PhD), Flinders University, Adelaide, SA, Australia; School of Pharmacy (S Shrestha PharmD), Monash University, Selangor Darul Ehsan, Malaysia; Department of Internal Medicine (R Sinto MD), University of Indonesia, Jakarta Pusat, Indonesia; Department of Internal Medicine (R Sinto MD), Dr. Cipto Mangunkusumo National Hospital, Jakarta Pusat, Indonesia; Department No.16 (V Y Skryabin MD), Moscow Research and Practical Centre on Addictions, Moscow, Russia; Department of Infectious Diseases and Epidemiology (A A Skryabina MD), Pirogov Russian National Research Medical University, Moscow, Russia; The Bizzell Group, Atlanta, GA, USA (Prof D A Sleet PhD); Rollins School of Public Health (Prof D A Sleet PhD), Emory University, Atlanta, GA, USA; Department of Surgery (B Socea PhD), "Sf. Pantelimon" Emergency Clinical Hospital Bucharest, Bucharest, Romania; Department of Nursing (Y Solomon MSc), Dire Dawa University, Dire Dawa, Ethiopia; Directive Board (R A Sousa FEAOO), Association of Licensed Optometry Professionals, Linda-a-Velha, Portugal; Hull York Medical School (I N Soyiri PhD), University of Hull, Hull City, UK; Center for Biotechnology and Microbiology (M Suleman PhD), University of Swat, Mingora, Swat, Pakistan; School of Life Sciences (M Suleman PhD), Xiamen University, Xiamen, China; National Institute of Epidemiology (R Suliankatchi Abdulkader MD), Indian Council of Medical Research, Chennai, India; School of Medicine (Prof J Sun PhD), Griffith University, Gold Coast, QLD, Australia; Department of Medicine (Prof R Tabarés-Seisdedos PhD), University of Valencia, Valencia, Spain; Department of Biostatistics and Epidemiology (M Taheri Soodejani PhD), Shahid Sadoughi University of Medical Sciences, Yazd, Iran; Department of Dermato-Venereology (M Tampa PhD), Dr. Victor Babes Clinical Hospital of Infectious Diseases and Tropical Diseases, Bucharest, Romania; Research and Development Center for Humanities and Health Management (I U Tarigan PhD), National Institute of Health Research & Development, Jakarta, Indonesia; Department of Economics (N Y Tat MS), Rice University, Houston, TX, USA; Research and Innovation (N Y Tat MS), Enventure Medical Innovation, Houston, TX, USA; Engineering Systems and Environment (A Tavakoli PhD), University of Virginia, Charlottesville, VA, USA; Department of Public Health (Y M Tefera MPH), Dire Dawa university, Dire Dawa, Ethiopia; Department of Pharmacology (P Thangaraju MD), All India Institute of Medical Sciences, Raipur, India; Department of Gastroenterology (N K Thomas MD), PSG Institute of Medical Sciences and Research, Coimbatore, India; Faculty of Public Health (J H V Ticoalu MPH), Universitas Sam Ratulangi, Manado, Indonesia; Department of Pathology (M B Tincho PhD), University of Cape Town, Cape Town, South Africa; Department of Biochemistry and Molecular Biology (M B Tincho PhD), University of Buea, Buea, Cameroon; General Department of Surgery (M Togtmol MD), National Center of Traumatology and Orthopedics,

Ulaanbaatar, Mongolia; Mongolian Burns Association, Ulaanbaatar, Mongolia (M Togtmol MD); Department of Pathology and Legal Medicine (M R Tovani-Palone PhD), University of São Paulo, Ribeirão Preto, Brazil; Modestum LTD, London, UK (M R Tovani-Palone PhD); School of Public Health and Social Work (M T N Tran PhD), Queensland University of Technology, Brisbane, QLD, Australia; Health informatic Department (M T N Tran PhD), Hanoi Medical University, Ha Noi, Vietnam; Department of Zoology (S Ullah PhD), Division of Science and Technology (S Ullah PhD), University of Education, Lahore, Lahore, Pakistan; Department of Paraclinical Sciences (S Umakanthan MD), The University of the West Indies, St. Augustine, Trinidad and Tobago; Clinical Cancer Research Center (S Valadan Tahbaz PhD, S Yahyazadeh Jabbari MD), Milad General Hospital, Tehran, Iran; Department of Microbiology (S Valadan Tahbaz PhD), Faculty of Medicine (M Zahir MD), Islamic Azad University, Tehran, Iran; Velez Sarsfield Hospital, Buenos Aires, Argentina (Prof P R Valdez Med); UKK Institute, Tampere, Finland (Prof T J Vasankari MD); Faculty of Medicine and Health Technology (Prof T J Vasankari MD), Tampere University, Tampere, Finland; Department of Medical and Surgical Sciences and Advanced Technologies (Prof M Veroux PhD), University of Catania, Catania, Italy; Health Policy and Management (D Vervoort MD), Johns Hopkins University, Baltimore, MD, USA; Occupational Health Unit (Prof F S Violante MD), Sant'Orsola Malpighi Hospital, Bologna, Italy; Foundation University Medical College (Prof Y Waheed PhD), Foundation University Islamabad, Islamabad, Pakistan; School of Population Health and Environmental Sciences (Y Wang PhD), King's College London, London, UK; Department of Medicine (C Wang MPH), Vanderbilt University, Nashville, TN, USA; Department of Physical Therapy (T Wiangkham PhD), Naresuan University, Phitsanulok, Thailand; Department of Community Medicine (N D Wickramasinghe MD), Rajarata University of Sri Lanka, Anuradhapura, Sri Lanka; Department of Public Health (A T Woday MPH), Samara University, Samara, Ethiopia; Department of Orthopaedics (Prof A Wu MD), Wenzhou Medical University, Wenzhou, China; Cancer Epidemiology and Prevention Research (L Yang PhD), Alberta Health Services, Calgary, BC, Canada; Department of Oncology (L Yang PhD), University of Calgary, Calgary, AB, Canada; Department of Health Management (A Yigit PhD, V Yiğit PhD), Süleyman Demirel Üniversitesi (Süleyman Demirel University), Isparta, Turkey; Department of Neuropsychopharmacology (N Yonemoto PhD), National Center of Neurology and Psychiatry, Kodaira, Japan; Department of Public Health (N Yonemoto PhD), Juntendo University, Tokyo, Japan; Macquarie Medical School (Y You PhD), Macquarie University, Macquarie University, NSW, Australia; Department of Health Policy and Management (Prof M Z Younis PhD), Jackson State University, Jackson, MS, USA; School of Medicine (Prof M Z Younis PhD), Tsinghua University, Beijing, China; Department of Clinical Pharmacy and Outcomes Sciences (I Yunusa PhD), University of South Carolina, Columbia, SC, USA; Department of internal medicine (H Yusefi MSc), Qom University of Medical Sciences, Qom, Iran; Research and Development Department (I Zare BSc), Sina Medical Biochemistry Technologies, Shiraz, Iran; Department of Bioengineering and Therapeutic Sciences (Prof M S Zastrozhin PhD), University of California San Francisco, San Francisco, CA, USA; Addictology Department (Prof M S Zastrozhin PhD), Russian Medical Academy of Continuous Professional Education, Moscow, Russia; School of Public Health (Y Zhang PhD), Hubei Province Key Laboratory of Occupational Hazard Identification and Control (Y Zhang PhD), Wuhan University of Science and Technology, Wuhan, China; Indian Institute of Public Health (Prof S Zodpey PhD), Public Health Foundation of India, Gurugram, India; Population Health Theme (Prof G C Patton MD), Murdoch Children's Research Institute, Melbourne, VIC, Australia.

Authors' Contributions

Providing data or critical feedback on data sources

Meriem Abdoun, Rami Abd-Rabu, Deldar Abdulah, Ame Abdurehman, Hassan Abidi, Hiwa Abubaker Ali, Tigist Adane, Sangeet Adhikari, Qorinah Adnani, Muhammad Afzal, Saira Afzal, Bright Ahinkorah, Sajjad Ahmad, Tauseef Ahmad, Ali Ahmadi, Ayman Ahmed, Muktar Ahmed, Tarik Ahmed Rashid, Janardhana Aithala, Budi Aji, Hanadi Al Hamad, Fares Alahdab, Fahad Alanezi, Astawus Alemayehu, Liaqat Ali, Syed Ali, Sami Almustanyir, Khalid Altirkawi, Nelson Alvis-Zakzuk, Mehrdad Amir-Behghadami, Hoda Amiri, Catalina Liliana Andrei, Tudorel Andrei, Davood Anvari, Sumadi Lukman Anwar, Jalal Arabloo, Morteza Arab-Zozani, Judie Arulappan, Seyyed Shamsadin Athari, Alok Atreya, Samad Azari, Ahmed Azzam, Ashish Badiye, Sara Bagherieh, Atif Baig, Maciej Banach, Palash Banik, Mainak Bardhan, Amadou Barrow, Pritish Baskaran, Alehegn Bekele, Akshaya Bhagavathula, Sonu Bhaskar, Zulfiqar Bhutta, Bagas Bintoro, Belay Bodicha, Souad Bouaoud, Katrin Burkart, Muhammad Hammad Butt, Luis Cámera, Julio Cesar Campuzano Rincon, Periklis Charalampous, Vijay Kumar Chattu, Bitew Chekole, Abdulaal Chitheer, Daniel Cho, Devasahayam Christopher, Natália Cruz-Martins, Xiaochen Dai, Giovanni Damiani, Aso Darwesh, Saswati Das, Getnet Demeke, Andreas Demetriades, Fikadu Dessalegn, Mostafa Dianatinasab, Nancy Diao, Diana Dias Da Silva, Milad Dodangeh, Paul Doku, Haneil Dsouza, Ebrahim Eini, Michael Ekholuenetale, Maysaa El Sayed Zaki, Ryenchindorj Erkhembayar, Adeniyi Fagbamigbe, Farshad Farzadfar, Ali Fatehizadeh, Seyed-Mohammad Fereshtehnejad, Getahun Fetensa, Richard Franklin, Takeshi Fukumoto, Peter Gaal, Muktar Gadanya, Yaseen Galali, Tushar Garg, Vidhi Garg, Mansour Ghafourifard, Sherief Ghozy, Sherief Ghozy, James Glasbey, Mahaveer Golechha, Pouya Goleij, Sapna Gupta, Veer Bala Gupta, Vivek Gupta, Arvin Haj-Mirzaian, Rabih Halwani, Lolemo Hanfore, Netanja Harlianto, Soheil Hassanipour, Hadi Hassankhani, Rasmus Havmoeller, Simon Hay, Nobuyuki Horita, Mehdi Hosseinzadeh, Amir Human Hoveidaei, Alexander Hsiao, Salman Hussain, Amal Hussein, Segun Ibitoye, Behzad Imani, Mohammad Mainul Islam, Sheikh Mohammed Shariful Islam, Nahlah Elkudssiah Ismail, Linda Merin J, Haitham Jahrami, Mihajlo Jakovljevic, Tahereh Javaheri, Sathish Kumar Jayapal, Sudha Jayaraman, Jost B Jonas, Tamas Joo, Jacek Jozwiak, Lam Judit, Mikk Jürisson, Vidya Kadashetti, Dler Kadir, Leila Kalankesh, Rohollah Kalhor, Rajesh Kamath, Himal Kandel, Rami Kantar, Neeti Kapoor, Hassan Karami, Gbenga Kayode, Cumali Keskin, Yousef Khader, Himanshu Khajuria, Mohammad Khammarnia, Abbas Khan, Imteyaz Khan, Maseer Khan, Md Nuruzzaman Khan, Yusra Khan, Jihee Kim, Yun Jin Kim, Adnan Kisa, Sezer Kisa, Parvaiz Koul, Sindhura Lakshmi Koulmane Laxminarayana, Kewal Krishan, Vijay Krishnamoorthy, Savita Lasrado, Sang-Woong Lee, Shaun Lee, Samson Legesse, Stephen Lim, Farzan Madadizadeh, Muhammed Magdy Abd El Razek, Ata Mahmoodpoor, Razzagh Mahmoudi, Alaa Makki, Elaheh Malakan Rad, Tauqeer Hussain Mallhi, Deborah Malta, Borhan Mansouri, Mohammad Ali Mansournia, Entezar Mehrabi Nasab, Ritesh Menezes, Dechasa Adare Mengistu, Atte Meretoja, Mohamed Mesregah, Ted Miller, Erkin Mirrakhimov, Prasanna Mithra, Madeline Moberg, Mokhtar Mohammadi, Reza Mohammadpourhodki, Shafiu Mohammed, Ali H Mokdad, Sara Momtazmanesh, Lorenzo Monasta, Saeideh Moosavi, Ebrahim Mostafavi, Sumaira Mubarik, Francesk Mulita, Ghulam Mustafa, Ahamarshan Nagarajan, Shumaila Nargus, Zuhair Natto, Biswa Nayak, Ionut Negoi, Samata Nepal, Sandhya Neupane Kandel, Chimezie Nzoputam, Ogochukwu Nzoputam, Chimedsuren Ochir, Julius Odhiambo, Andrew Olagunju, Emad Omer, Adrian Otoiu, Bilcha Oumer, Mayowa Owolabi, Mahesh P A, Alicia Padron-Monedero, Amy Peden, Jagadish Rao Padubidri, Tamas Palicz, Songhomitra Panda-Jonas, Shahina Pardhan, Sangram Patel, Aslam Pathan, Shrikant Pawar, Linh Phuong Doan, Julian Pillay, Zahra Piracha, Naeimeh Pourtaheri, Pankaja Raghav, Fakher Rahim, Firman Rahman, Amir Masoud Rahmani, Shayan Rahmani, Sheena Ramazanu, Kiana Ramezanzadeh, Juwel Rana, Saleem Rana, Chythra Rao, Sowmya J Rao, Prateek Rastogi, David L Rawaf, Salman Rawaf, Lal Rawal, Reza Rawassizadeh, Leonardo Roever, Luca Ronfani, Aly Saad, Siamak Sabour, Basema Saddik, Umar Saeed, Sher Safi, Harihar Sahoo, Marwa Salem, Abdallah Samy, Milena Santric-Milicevic, Arash Sarveazad, Brijesh Sathian, Maheswar Satpathy, Ganesh Kumar Saya, Ione Schneider, David Schwebel, Allen Seylani, Melika Shafeghat, Pritik Shah, Fariba Shahraki-Sanavi, Masood Shaikh, Mohd Shanawaz, Mohammed Shannawaz, Rahim Ali Sheikhi, Jiabin Shen, B Suresh Shetty, Jae Il Shin, K Shivakumar, Parnian Shobeiri, Sunil Shrestha, Apurva Shrigiriwar, Soraya Siabani, Negussie Sidemo, Wudneh Simegn, Tanya Singh, Valentin Skryabin, Anna Skryabina, Ireneous Soyiri, Muhammad Suleman, Rizwan Suliankatchi Abdulkader, Miklos Szocska, Rafael Tabarés-Seisdedos, Ensiyeh Taheri, Mircea Tampa, Ker-Kan Tan, Belay Tefera, Yibekal Tefera, Marius Tincho, Amir Tiyuri, Munkhsaikhan Togtmol, Marcos Tovani-Palone, Mai Tran, Irfan Ullah, Saif Ullah, Sana Ullah, Srikanth Umakanthan, Bhaskaran Unnikrishnan, Era Upadhyay, Sahel Valadan

Tahbaz, Pascual Valdez, Tommi Vasankari, Vasily Vlassov, Linh Vu, Yasir Waheed, Taweewat Wiangkham, Hossein Yahyazadeh, Sanni Yaya, Vahit Yiäÿit, Eshetu Yisihak, Naohiro Yonemoto, Mustafa Younis, Chuanhua Yu, Ismaeel Yunusa, Iman Zare, Zhi-Jiang Zhang, Arash Ziapour, Mohammad Zoladl, Zhiyong Zou.

Developing methods or computational machinery

Hiwa Abubaker Ali, Tigist Adane, Saira Afzal, Araz Ahmad, Ali Ahmadi, Ali Ahmed, Muktar Ahmed, Tarik Ahmed Rashid, Meisam Akhlaghdoust, Liaqat Ali, Hoda Amiri, Ahmed Azzam, Akshaya Bhagavathula, Belay Bodicha, Xiaochen Dai, Aso Darwesh, Mostafa Dianatinasab, Ali Fatehizadeh, Kate Francis, Sherief Ghozy, Bhawna Gupta, Sheikh Hossain, Mehdi Hosseinzadeh, Tahereh Javaheri, Hassan Karami, Samad Karkhah, Cumali Keskin, Mohammad Khammarnia, Abbas Khan, Adnan Kisa, Sang-Woong Lee, Razzagh Mahmoudi, Alaa Makki, Borhan Mansouri, Moonis Mirza, Mokhtar Mohammadi, Ali H Mokdad, Francesk Mulita, Shumaila Nargus, Ahmed Omar Bali, Emad Omer, Bilcha Oumer, Amy Peden, Firman Rahman, Amir Masoud Rahmani, Saleem Rana, Reza Rawassizadeh, Jefferson Rodriguez, Umar Saeed, Abdallah Samy, Maheswar Satpathy, Ganesh Kumar Saya, Fariba Shahraki-Sanavi, Mohammed Shannawaz, Negussie Sidemo, Ensiyeh Taheri, Belay Tefera, Mai Tran, Taweewat Wiangkham, Hossein Yusefi, Kourosh Zarea, Arash Ziapour.

Providing critical feedback on methods or results

Amirali Aali, Michael Abdelmasseh, Meriem Abdoun, Rami Abd-Rabu, Deldar Abdulah, Ame Abdurehman, Hassan Abidi, Richard Aboagye, Hiwa Abubaker Ali, Denberu Adane, Tigist Adane, Isaac Addo, Ololade Adewole, Sangeet Adhikari, Mohammad Adnan, Qorinah Adnani, Aanuoluwapo Afolabi, Muhammad Afzal, Saira Afzal, Bright Ahinkorah, Araz Ahmad, Sajjad Ahmad, Tauseef Ahmad, Ali Ahmadi, Ali Ahmed, Ayman Ahmed, Haroon Ahmed, Jivan Ahmed, Muktar Ahmed, Tarik Ahmed Rashid, Janardhana Aithala, Budi Aji, Meisam Akhlaghdoust, Hanadi Al Hamad, Fares Alahdab, Fahad Alanezi, Astawus Alemayehu, Liaqat Ali, Syed Ali, Yousef Alimohamadi, Vahid Alipour, Syed Aljunid, Louay Almidani, Sami Almustanyir, Khalid Altirkawi, Nelson Alvis-Zakzuk, Edward Ameyaw, Tarek Amin, Mehrdad Amir-Behghadami, Hoda Amiri, Sohrab Amiri, Tadele Anagaw, Catalina Liliana Andrei, Tudorel Andrei, Davood Anvari, Sumadi Lukman Anwar, Anayochukwu Anyasodor, Jalal Arabloo, Morteza Arab-Zozani, Judie Arulappan, Ashokan Arumugam, Armin Aryannejad, Tahira Ashraf, Seyyed Shamsadin Athari, Alok Atreya, Sameh Attia, Avinash Aujayeb, Sina Azadnajafabad, Mohammadreza Azangou-Khyavy, Samad Azari, Amirhossein Azari Jafari, Hosein Azizi, Ahmed Azzam, Ashish Badiye, Nayereh Baghcheghi, Sara Bagherieh, Atif Baig, Shankar Bakkannavar, Maciej Banach, Palash Banik, Hansi Bansal, Mainak Bardhan, Amadou Barrow, Pritish Baskaran, Saurav Basu, Nebiyou Bayileyegn, Alehegn Bekele, Salaheddine Bendak, Alemshet Berhie, Devidas Bhagat, Akshaya Bhagavathula, Nikha Bhardwaj, Pankaj Bhardwaj, Sonu Bhaskar, Ajay Bhat, Krittika Bhattacharyya, Zulfiqar Bhutta, Sadia Bibi, Bagas Bintoro, Belay Bodicha, Archith Boloor, Souad Bouaoud, Julie Brown, Katrin Burkart, Muhammad Hammad Butt, Nadeem Shafique Butt, Luis Cámera, Chao Cao, Andre Carvalho, Márcia Carvalho, Promit Chakraborty, Eeshwar Chandrasekar, Jung-Chen Chang, Periklis Charalampous, Jaykaran Charan, Vijay Kumar Chattu, Bitew Chekole, Abdulaal Chitheer, Daniel Cho, Devasahayam Christopher, Isaac Chukwu, Natália Cruz-Martins, Omid Dadras, Saad Dahlawi, Xiaochen Dai, Giovanni Damiani, Reza Darvishi Cheshmeh Soltani, Aso Darwesh, Saswati Das, Anna Dastiridou, Sisay Abebe Debela, Getnet Demeke, Andreas Demetriades, Solomon Demissie, Fikadu Dessalegn, Abebaw Desta, Mostafa Dianatinasab, Nancy Diao, Diana Dias Da Silva, Daniel Diaz, Lankamo Digesa, Mengistie Diress, Shirin Djalalinia, Milad Dodangeh, Deepa Dongarwar, Haneil Dsouza, Ebrahim Eini, Michael Ekholuenetale, Temitope Ekundayo, Maysaa El Sayed Zaki, Ahmed Elagali, Hala Elhabashy, Muhammed Elhadi, Daniel Enyew, Ryenchindorj Erkhembayar, Sharareh Eskandarieh, Farshid Etaee, Adeniyi Fagbamigbe, Pawan Faris, Andre Faro, Farshad Farzadfar, Ali Fatehizadeh, Seyed-Mohammad Fereshtehnejad, Abdullah Feroze, Getahun Fetensa, Bikila Feyisa, Irina Filip, Florian Fischer, Masoud Foroutan, Kayode Fowobaje, Kate Francis, Richard Franklin, Takeshi Fukumoto, Peter Gaal, Muktar Gadanya, Yaseen Galali, Nasrin Galehdar, Balasankar Ganesan, Tushar Garg, Vidhi Garg, Mesfin Gebrehiwot, Yosef Gebremariam, Mansour Ghafourifard, Reza Ghanbari, Mohammad Ghasemi Nour, Maryam Gholamalizadeh, Ali Gholami, Sherief Ghozy, Sherief Ghozy, Syed Amir Gilani, Tiffany Gill, Zeleke Girma, James Glasbey, Franklin Glozah, Mahaveer Golechha, Michal Grivna, Habtamu Guadie, Damitha Gunawardane, Yuming Guo, Bhawna Gupta, Sapna Gupta, Veer Bala Gupta, Vivek Gupta, Arvin Haj-Mirzaian, Rabih Halwani, Randah Hamadeh, Lolemo Hanfore, Asif Hanif, Arief Hargono, Netanja Harlianto, Mehdi Harorani, Ahmed Hasaballah, S M

Mahmudul Hasan, Soheil Hassanipour, Hadi Hassankhani, Rasmus Havmoeller, Simon Hay, Mohammad Heidari, Delia Hendrie, Demisu Heyi, Yuta Hiraike, Ramesh Holla, Nobuyuki Horita, Mohammad Hossain, Sheikh Hossain, Sedighe Hosseini Shabanan, Mehdi Hosseinzadeh, Amir Human Hoveidaei, Alexander Hsiao, Salman Hussain, Amal Hussein, Segun Ibitoye, Olayinka Ilesanmi, Irena Ilic, Milena Ilic, Mustapha Immurana, Leeberk Inbaraj, Mohammad Mainul Islam, Rakibul Islam, Sheikh Mohammed Shariful Islam, Nahlah Elkudssiah Ismail, Linda Merin J, Haitham Jahrami, Mihajlo Jakovljevic, Tahereh Javaheri, Sathish Kumar Jayapal, Umesh Jayarajah, Sudha Jayaraman, Jayakumar Jeganathan, Ravi Jha, Jost B Jonas, Tamas Joo, Nitin Joseph, Jacek Jozwiak, Lam Judit, Mikk Jürisson, Vidya Kadashetti, Dler Kadir, Laleh Kalankesh, Leila Kalankesh, Rohollah Kalhor, Vineet Kamal, Rajesh Kamath, Himal Kandel, Rami Kantar, Neeti Kapoor, Hassan Karami, Ibraheem Karaye, Samad Karkhah, Patrick Katoto, Joonas Kauppila, Gbenga Kayode, Leila Keikavoosi-Arani, Cumali Keskin, Yousef Khader, Himanshu Khajuria, Mohammad Khammarnia, Abbas Khan, Ejaz Khan, Imteyaz Khan, Maseer Khan, Md Nuruzzaman Khan, Moien Khan, Yusra Khan, Javad Khanali, Moawiah Khatatbeh, Hamid Reza Khayat Kashani, Habibolah Khazaie, Fatemeh Khoshpasand, Jagdish Khubchandani, Zemene Kifle, Jihee Kim, Yun Jin Kim, Adnan Kisa, Sezer Kisa, Cameron Kneib, Hamid Reza Koohestani, Sindhura Lakshmi Koulmane Laxminarayana, Ai Koyanagi, Kewal Krishan, Vijay Krishnamoorthy, Burcu Kucuk Bicer, Dr Nithin Kumar, Narinder Kumar, Naveen Kumar, Om Kurmi, Carlo La Vecchia, Lucie Laflamme, Bagher Larijani, Savita Lasrado, Paolo Lauriola, Sang-Woong Lee, Shaun Lee, Wei-Chen Lee, Yo Han Lee, Samson Legesse, Shanshan Li, Stephen Lim, László Lorenzovici, Amana Luke, Farzan Madadizadeh, Muhammed Magdy Abd El Razek, Ata Mahmoodpoor, Razzagh Mahmoudi, Marzieh Mahmoudimanesh, Azeem Majeed, Alaa Makki, Elaheh Malakan Rad, Mohammad-Reza Malekpour, Ahmad Malik, Tauqeer Hussain Mallhi, Deborah Malta, Zahra Mansour Kiaee, Borhan Mansouri, Mohammad Ali Mansournia, Elezebeth Mathews, Sazan Maulud, Dennis Mazingi, Entezar Mehrabi Nasab, Oliver Mendoza-Cano, Ritesh Menezes, Dechasa Adare Mengistu, Alexios-Fotios Mentis, Atte Meretoja, Mohamed Mesregah, Tomislav Mestrovic, Ana Micheletti Gomide Nogueira De Sá, Ted Miller, Seyed Peyman Mirghaderi, Andreea Mirica, Seyyedmohammadsadeq Mirmoeeni, Moonis Mirza, Sanjeev Misra, Prasanna Mithra, Chaitanya Mittal, Madeline Moberg, Javad Moghadasi, Mokhtar Mohammadi, Soheil Mohammadi, Reza Mohammadpourhodki, Shafiu Mohammed, Teroj Mohammed, Mohammad Mohseni, Ali H Mokdad, Sara Momtazmanesh, Lorenzo Monasta, Mohammad Ali Moni, Saeideh Moosavi, Rafael Moreira, Shane Morrison, Ebrahim Mostafavi, Haleh Mousavi Isfahani, Sumaira Mubarik, Soumyadeep Mukherjee, Francesk Mulita, Ghulam Mustafa, Ahamarshan Nagarajan, Mukhammad David Naimzada, Vinay Nangia, Shumaila Nargus, Zuhair Natto, Biswa Nayak, Ionut Negoi, Seyed Aria Nejadghaderi, Samata Nepal, Sandhya Neupane Kandel, Nafise Noroozi, Chimezie Nzoputam, Ogochukwu Nzoputam, Chimedsuren Ochir, Julius Odhiambo, Hassan Okati-Aliabad, Osaretin Okonji, Andrew Olagunju, Ahmed Omar Bali, Emad Omer, Adrian Otoiu, Nikita Otstavnov, Stanislav Otstavnov, Bilcha Oumer, Mayowa Owolabi, Mahesh P A, Jagadish Rao Padubidri, Mohammad Taha Pahlevan Fallahy, Tamas Palicz, Songhomitra Panda-Jonas, Seithikurippu Pandi-Perumal, Shahina Pardhan, Eun-Kee Park, Sangram Patel, Aslam Pathan, Siddhartha Pati, Uttam Paudel, Shrikant Pawar, Amy Peden, Paolo Pedersini, Mario Peres, Ionela-Roxana Petcu, Michael Phillips, Linh Phuong Doan, Julian Pillay, Zahra Piracha, Mohsen Poursadeqiyan, Ibrahim Qattea, Amir Radfar, Pankaja Raghav, Fakher Rahim, Firman Rahman, Mosiur Rahman, Muhammad Aziz Rahman, Amir Masoud Rahmani, Shayan Rahmani, Sheena Ramazanu, Kiana Ramezanzadeh, Juwel Rana, Saleem Rana, Chythra Rao, Sowmya J Rao, Vahid Rashedi, Mohammad-Mahdi Rashidi, Prateek Rastogi, Azad Rasul, David L Rawaf, Salman Rawaf, Lal Rawal, Reza Rawassizadeh, Negar Rezaei, Mohsen Rezaeian, Aziz Rezapour, Sahba Rezazadeh-Khadem, Abanoub Riad, Jennifer Rickard, Jefferson Rodriguez, Leonardo Roever, Luca Ronfani, Chandan S N, Aly Saad, Siamak Sabour, Leila Sabzmakan, Basema Saddik, Malihe Sadeghi, Mohammad Saeb, Umar Saeed, Sahar Saeedi Moghaddam, Sher Safi, Biniyam Sahiledengle, Harihar Sahoo, Mohammad Ali Sahraian, Morteza Saki, Sana Salehi, Marwa Salem, Abdallah Samy, Juan Sanabria, Milena Santric-Milicevic, Muhammad Arif Saqib, Yaser Sarikhani, Arash Sarveazad, Brijesh Sathian, Maheswar Satpathy, Ganesh Kumar Saya, David Schwebel, Subramanian Senthilkumaran, Allen Seylani, Hosein Shabaninejad, Pritik Shah, Saeed Shahabi, Ataollah Shahbandi, Fariba Shahraki-Sanavi, Masood Shaikh, Elaheh Shaker, Mehran Shams-Beyranvand, Mohd Shanawaz, Mohammed Shannawaz, Mequannent Sharew, Neeraj Sharma, Maryam Shayan, Rahim Ali Sheikhi, Rahim Ali Sheikhi, Jiabin Shen, Pavanchand Shetty, Jae Il Shin, K Shivakumar, Parnian Shobeiri, Seyed Afshin Shorofi, Sunil Shrestha, Apurva Shrigiriwar, Soraya Siabani, Negussie Sidemo, Wudneh Simegn, Ehsan Sinaei, Paramdeep Singh, Tanya Singh, Robert Sinto, Md Shahjahan Siraj, Valentin Skryabin, Anna Skryabina, Bogdan Socea, Marco Solmi, Yonatan Solomon, Yi Song, Raúl Sousa, Ireneous Soyiri, Mark

Stokes, Muhammad Suleman, Rizwan Suliankatchi Abdulkader, Jing Sun, Miklos Szocska, Dr Rekha T, Rafael Tabarés-Seisdedos, Seyyed Mohammad Tabatabaei, Mohammad Tabish, Ensiyeh Taheri, Moslem Taheri Soodejani, Mircea Tampa, Ker-Kan Tan, Ingan Tarigan, Md Tariqujjaman, Arash Tavakoli, Belay Tefera, Yibekal Tefera, Gebremaryam Temesgen, Mohamad-Hani Temsah, Pugazhenthan Thangaraju, Nikhil Thomas, Jansje Ticoalu, Marius Tincho, Amir Tiyuri, Munkhsaikhan Togtmol, Marcos Tovani-Palone, Mai Tran, Irfan Ullah, Saif Ullah, Sana Ullah, Srikanth Umakanthan, Bhaskaran Unnikrishnan, Era Upadhyay, Sahel Valadan Tahbaz, Pascual Valdez, Siavash Vaziri, Massimiliano Veroux, Dominique Vervoort, Francesco Violante, Linh Vu, Yasir Waheed, Cong Wang, Yanzhong Wang, Taweewat Wiangkham, Nuwan Wickramasinghe, Abay Woday, Ai-Min Wu, Gahin Yahya, Hossein Yahyazadeh, Sanni Yaya, Vahit Yiäÿit, Arzu Yigit, Eshetu Yisihak, Naohiro Yonemoto, Yuyi You, Mustafa Younis, Chuanhua Yu, Ismaeel Yunusa, Hossein Yusefi, Mazyar Zahir, Sojib Bin Zaman, Kourosh Zarea, Mikhail Zastrozhin, Yunquan Zhang, Zhi-Jiang Zhang, Arash Ziapour, Sanjay Zodpey, Mohammad Zoladl, Zhiyong Zou.

Drafting the work or revising is critically for important intellectual content

Amirali Aali, Mohsen Abbasi-Kangevari, Zeinab Abbasi-Kangevari, Michael Abdelmasseh, Meriem Abdoun, Rami Abd-Rabu, Ame Abdurehman, Ayele Abebe, Getachew Abebe, Aidin Abedi, Hassan Abidi, Eman Abu-Gharbieh, Denberu Adane, Isaac Addo, Mohammad Adnan, Qorinah Adnani, Aanuoluwapo Afolabi, Muhammad Afzal, Saira Afzal, Zahra Aghdam, Bright Ahinkorah, Ali Ahmadi, Ali Ahmed, Ayman Ahmed, Jivan Ahmed, Muktar Ahmed, Janardhana Aithala, Fares Alahdab, Liaqat Ali, Syed Ali, Sami Almustanyir, Nelson Alvis-Zakzuk, Edward Ameyaw, Tarek Amin, Mehrdad Amir-Behghadami, Sohrab Amiri, Tadele Anagaw, Catalina Liliana Andrei, Tudorel Andrei, Anayochukwu Anyasodor, Jalal Arabloo, Morteza Arab-Zozani, Asrat Arja, Judie Arulappan, Ashokan Arumugam, Saeed Asgary, Sameh Attia, Avinash Aujayeb, Atalel Awedew, Sina Azadnajafabad, Mohammadreza Azangou-Khyavy, Amirhossein Azari Jafari, Ahmed Azzam, Ashish Badiye, Sara Bagherieh, Atif Baig, Asaminew Balta, Maciej Banach, Palash Banik, Hansi Bansal, Mainak Bardhan, Francesco Barone-Adesi, Amadou Barrow, Azadeh Bashiri, Pritish Baskaran, Saurav Basu, Abebe Bekel, Alehegn Bekele, Salaheddine Bendak, Isabela Bensenor, Devidas Bhagat, Akshaya Bhagavathula, Sonu Bhaskar, Krittika Bhattacharyya, Sadia Bibi, Saeid Bitaraf, Belay Bodicha, Souad Bouaoud, Julie Brown, Muhammad Hammad Butt, Nadeem Shafique Butt, Chao Cao, Andre Carvalho, Márcia Carvalho, Eeshwar Chandrasekar, Vijay Kumar Chattu, Bitew Chekole, Daniel Cho, Hitesh Chopra, Devasahayam Christopher, Natália Cruz-Martins, Giovanni Damiani, Gary Darmstadt, Saswati Das, Anna Dastiridou, Amin Dehghan, Andreas Demetriades, Solomon Demissie, Fikadu Dessalegn, Diana Dias Da Silva, Daniel Diaz, Milad Dodangeh, Paul Doku, Deepa Dongarwar, Haneil Dsouza, Ebrahim Eini, Temitope Ekundayo, Maysaa El Sayed Zaki, Ahmed Elagali, Mostafa Elbahnasawy, Muhammed Elhadi, Sharareh Eskandarieh, Farshid Etaee, Adeniyi Fagbamigbe, Abbas Farmany, Andre Faro, Ali Fatehizadeh, Seyed-Mohammad Fereshtehnejad, Abdullah Feroze, Bikila Feyisa, Irina Filip, Florian Fischer, Behzad Foroutan, Masoud Foroutan, Kate Francis, Takeshi Fukumoto, Peter Gaal, Muktar Gadanya, Nasrin Galehdar, Balasankar Ganesan, Tushar Garg, Vidhi Garg, Mesfin Gebrehiwot, Yibeltal Yismaw Gela, Urge Gerema, Mansour Ghafourifard, Seyyed-Hadi Ghamari, Mohammad Ghasemi Nour, Ali Gholamrezanezhad, Sherief Ghozy, Sherief Ghozy, Tiffany Gill, Iago Giné-Vázquez, James Glasbey, Franklin Glozah, Michal Grivna, Habtamu Guadie, Damitha Gunawardane, Bhawna Gupta, Sapna Gupta, Veer Bala Gupta, Vivek Gupta, Arvin Haj-Mirzaian, Rabih Halwani, Randah Hamadeh, Sajid Hameed, Lolemo Hanfore, Netanja Harlianto, Ahmed Hasaballah, S. M. Mahmudul Hasan, Amr Hassan, Rasmus Havmoeller, Simon Hay, Demisu Heyi, Yuta Hiraike, Ramesh Holla, Nobuyuki Horita, Mohammad Hossain, Sheikh Hossain, Sedighe Hosseini Shabanan, Sorin Hostiuc, Amir Human Hoveidaei, Salman Hussain, Amal Hussein, Segun Ibitoye, Olayinka Ilesanmi, Irena Ilic, Milena Ilic, Mustapha Immurana, Mohammad Mainul Islam, Rakibul Islam, Sheikh Mohammed Shariful Islam, Nahlah Elkudssiah Ismail, Linda Merin J, Haitham Jahrami, Mihajlo Jakovljevic, Manthan Janodia, Sathish Kumar Jayapal, Umesh Jayarajah, Sudha Jayaraman, Jayakumar Jeganathan, Bedru Jemal, Ravi Jha, Jost B Jonas, Tamas Joo, Nitin Joseph, Jacek Jozwiak, Lam Judit, Mikk Jürisson, Ali Kabir, Vidya Kadashetti, Dler Kadir, Leila Kalankesh, Vineet Kamal, Himal Kandel, Rami Kantar, Neeti Kapoor, Hassan Karami, Samad Karkhah, Patrick Katoto, Joonas Kauppila, Gbenga Kayode, Cumali Keskin, Yousef Khader, Himanshu Khajuria, Mohammad Khammarnia, Abbas Khan, Ejaz Khan, Imteyaz Khan, Maseer Khan, Md Nuruzzaman Khan, Moien Khan, Yusra Khan, Javad Khanali, Moawiah Khatatbeh, Hamid Reza Khayat Kashani, Jagdish Khubchandani, Yun Jin Kim, Adnan Kisa, Sezer Kisa, Cameron Kneib, Farzad Kompani, Parvaiz Koul, Sindhura Lakshmi Koulmane Laxminarayana, Ai Koyanagi, Kewal Krishan, Burcu Kucuk Bicer, Manasi Kumar, Narinder Kumar, Naveen Kumar, Om Kurmi, Carlo La Vecchia, Lucie

Laflamme, Iván Landires, Bagher Larijani, Savita Lasrado, Paolo Lauriola, Yo Han Lee, Samson Legesse, Áurea Madureira-Carvalho, Muhammed Magdy Abd El Razek, Soleiman Mahjoub, Ata Mahmoodpoor, Razzagh Mahmoudi, Azeem Majeed, Elaheh Malakan Rad, Mohammad-Reza Malekpour, Ahmad Malik, Tauqeer Hussain Mallhi, Borhan Mansouri, Elezebeth Mathews, Sazan Maulud, Dennis Mazingi, Oliver Mendoza-Cano, Ritesh Menezes, Dechasa Adare Mengistu, Alexios-Fotios Mentis, Atte Meretoja, Mohamed Mesregah, Tomislav Mestrovic, Ana Micheletti Gomide Nogueira De Sá, Ted Miller, Seyed Peyman Mirghaderi, Seyyedmohammadsadeq Mirmoeeni, Moonis Mirza, Chaitanya Mittal, Madeline Moberg, Esmaeil Mohammadi, Soheil Mohammadi, Shafiu Mohammed, Teroj Mohammed, Ali H Mokdad, Sara Momtazmanesh, Mohammad Ali Moni, Rafael Moreira, Shane Morrison, Lorenzo Muccioli, Soumyadeep Mukherjee, Francesk Mulita, Ghulam Mustafa, Ahamarshan Nagarajan, Mukhammad David Naimzada, Vinay Nangia, Shumaila Nargus, Hasan Nassereldine, Zuhair Natto, Biswa Nayak, Ionut Negoi, Seyed Aria Nejadghaderi, Sandhya Neupane Kandel, Virginia Nuñez-Samudio, Chimezie Nzoputam, Ogochukwu Nzoputam, Oluwakemi Odukoya, Osaretin Okonji, Andrew Olagunju, Adrian Otoiu, Nikita Otstavnov, Stanislav Otstavnov, Bilcha Oumer, Mayowa Owolabi, Mahesh P A, Alicia Padron-Monedero, Jagadish Rao Padubidri, Mohammad Taha Pahlevan Fallahy, Tamas Palicz, Songhomitra Panda-Jonas, Shahina Pardhan, Siddhartha Pati, Uttam Paudel, Shrikant Pawar, Amy Peden, Paolo Pedersini, Mario Peres, Ionela-Roxana Petcu, Michael Phillips, Linh Phuong Doan, Julian Pillay, Zahra Piracha, Naeimeh Pourtaheri, Ibrahim Qattea, Amir Radfar, Ata Rafiee, Pankaja Raghav, Fakher Rahim, Shayan Rahmani, Sheetal Raj Moolambally, Sheena Ramazanu, Kiana Ramezanzadeh, Saleem Rana, Chythra Rao, Sowmya J Rao, Vahid Rashedi, David L Rawaf, Salman Rawaf, Lal Rawal, Nazila Rezaei, Sahba Rezazadeh-Khadem, Abanoub Riad, Muhammad Riaz, Jennifer Rickard, Jefferson Rodriguez, Leonardo Roever, Bedanta Roy, Manjula S, Chandan S N, Aly Saad, Leila Sabzmakan, Basema Saddik, Malihe Sadeghi, Umar Saeed, Sahar Saeedi Moghaddam, Sher Safi, Biniyam Sahiledengle, Harihar Sahoo, Mohammad Ali Sahraian, Morteza Saki, Payman Salamati, Marwa Salem, Abdallah Samy, Juan Sanabria, Milena Santric-Milicevic, Yaser Sarikhani, Maheswar Satpathy, Ganesh Kumar Saya, Ione Schneider, David Schwebel, Hamed Seddighi, Allen Seylani, Pritik Shah, Saeed Shahabi, Fariba Shahraki-Sanavi, Elaheh Shaker, Mehran Shams-Beyranvand, Mohd Shanawaz, Mequannent Sharew, Neeraj Sharma, Bereket Shashamo, Jiabin Shen, Pavanchand Shetty, Nebiyu Shitaye, K Shivakumar, Parnian Shobeiri, Seyed Afshin Shorofi, Sunil Shrestha, Apurva Shrigiriwar, Negussie Sidemo, Wudneh Simegn, Paramdeep Singh, Tanya Singh, Robert Sinto, Valentin Skryabin, Anna Skryabina, David Sleet, Bogdan Socea, Marco Solmi, Yonatan Solomon, Raúl Sousa, Mark Stokes, Muhammad Suleman, Miklos Szocska, Ensiyeh Taheri, Mircea Tampa, Ker-Kan Tan, Nathan Tat, Vivian Tat, Arash Tavakoli, Belay Tefera, Yibekal Tefera, Gebremaryam Temesgen, Mohamad-Hani Temsah, Pugazhenthan Thangaraju, Jansje Ticoalu, Marius Tincho, Marcos Tovani-Palone, Mai Tran, Irfan Ullah, Saif Ullah, Srikanth Umakanthan, Bhaskaran Unnikrishnan, Era Upadhyay, Sahel Valadan Tahbaz, Tommi Vasankari, Massimiliano Veroux, Dominique Vervoort, Francesco Violante, Vasily Vlassov, Linh Vu, Yanzhong Wang, Yuan-Pang Wang, Taweewat Wiangkham, Nuwan Wickramasinghe, Abay Woday, Ai-Min Wu, Gahin Yahya, Hossein Yahyazadeh, Lin Yang, Sanni Yaya, Vahit Yiäÿit, Arzu Yigit, Naohiro Yonemoto, Hossein Yusefi, Mazyar Zahir, Sojib Bin Zaman, Iman Zare, Mikhail Zastrozhin, Arash Ziapour, Mohammad Zoladl, Zhiyong Zou.

Managing the estimation or publications process

Kate Francis, Simon I Hay, Ali H Mokdad, Amy Peden.

Author Acknowledgements and Declarations of Interest

Acknowledgements

S Afzal acknowledge the moral and intellectual support of the Department of Community Medicine and Epidemiology at King Edward Medical University Lahore. S Aljunid would like to acknowledge Department of Health Policy and Management, College of Public Health, Kuwait University for the approval and support to participate in this research project. M Carvalho acknowledges the support from FCT in the scope of the project UIDP/04378/2020 and UIDB/04378/2020 of UCIBIO and the project LA/P/0140/2020 of i4HB. D Dias da Silva acknowledges national funds from FCT - Fundação para a Ciência e a Tecnologia, I.P., in the scope of the project UIDP/04378/2022 and UIDB/04378/2022 of the Research Unit on Applied Molecular Biosciences - UCIBIO and the project LA/P/0140/2022 of the Associate Laboratory Institute for Health and Bioeconomy - i4HB." T Ekundayo thanks the African-German Network of Excellence in Science (AGNES), the Federal Ministry of Education and Research (BMBF) and the Alexander von Humboldt Foundation (AvH) for financial support. I Giné-Vázquez is supported by CIBER -Consorcio Centro de Investigación Biomédica en Red- (CB 2021), Instituto de Salud Carlos III, Ministerio de Ciencia e Innovación. J Glasbey is support by a NIHR Doctoral Research Fellowship (NIHR300175). V K Gupta and V B Gupta acknowledge funding support from National Health and Medical Research Council (NHMRC), Australia. S Hussain was supported from Operational Programme Research, Development and Education "Project, Postdoc2MUNI (No.CZ.02.2.69/0.0/0.0/18_053/0016952). S Islam acknowledges support from the National Heart Foundation of Australia and NHMRC. The Serbian part of this GBD related contribution was co-financed through the Grant OI175014 of the Ministry of Education Science and Technological Development of the Republic of Serbia. M N Khan acknowledges the support of Jatiya Kabi Kazi Nazrul Islam University, Bangladesh. S Koulmane Laxminarayana Institutional support provided by Manipal Academy of Higher Education is gratefully acknowledged. K Krishan is supported by the UGC Centre of Advanced Study (Phase II), awarded to the Department of Anthropology, Panjab University, Chandigarh, India. "I Landires is a member of the Sistema Nacional de Investigación (SNI), which is supported by Panamaâ€™s Secretaría Nacional de Ciencia, Tecnología e Innovación (SENACYT). " Elezebeth Mathews is supported by a Clinical and Public Health Early Career Fellowship (grant number IA/CPHE/17/1/503345) from the DBT India Alliance/Wellcome Trust Department of Biotechnology, India Alliance (2018–2023). L Monasta and L Ronfani acknowledge support from the Italian Ministry of Health at the Institute for Maternal and Child Health IRCCS Burlo Garofolo, Trieste - Italy (RC 34/2017). V Nuñez-Samudio is a member of the Sistema Nacional de Investigación (SNI), which is supported by Panama's Secretaría Nacional de Ciencia, Tecnología e Innovación (SENACYT). J R Padubidri acknowledges Kasturba Medical College, Mangalore, Manipal Academy of Higher Education, Manipal, India for their constant support in Research. M R Phillips was supported in part by a grant from the Global Alliance for Chronic Diseases- National Natural Science Foundation of China (NSFC. No. 81761128031). U Saeed and Z Piracha acknowledge the International Center of Medical Sciences Research Islamabad (44000) Pakistan. A M Samy acknowledges support from Ain Shams University and the Egyptian Fulbright Mission Program. "B S Shetty and P Shetty acknowledge Kasturba Medical College, Mangalore, Manipal Academy of Higher Education, Manipal for all the support. D A Sleet is partially supported by The Bizzell Group, LLC. S Ullah acknowledges the support from Institute of Soil and Environmental Sciences, University of Agriculture, Faisalabad-Pakistan. S B Zaman acknowledges a scholarship from the Australian Government Research Training Program (RTP) in support of his academic career.

Declarations of Interest

S Afzal is a member of the Institutional Ethical Review Committee in King Edward Medical University Lahore, Pakistan and is a fellow of Faculty of Public Health England and fellow of College of Physicians and Surgeons Pakistan and a member of the Pakistan Association of Medical Editors. SMM Bhaskar is the Board Director of the Rotary Club of Sydney and the International Rotary Fellowship of Health Professionals (UK), the chair and amanger of the Global Health and Migration Hub Community, Global Health Hub Germany, and the district chair of Rotary District 9675 Diversity, Equity, & Inclusion. A K Demetriades reports lecture honorarium from Stryker and Integra and is an unpaid invited faculty on courses for European Association of Neurosurgical Societies (EANS), AO Spine, and the Global Neuro Foundation. A K Demetriades als reports non-salaried roles as the president of EANS and a board member of

the Global Neuro Foundation. R Franklin reports voluntary board member positions at Kidsafe Queensland, Australia and Northern Territory, Farmsafe Australia, and Royal Life Saving Society Australia. SMS Islam reports fellowships from the National Heart Foundation of Australia and National Health and Medical Research Council (NHMRC). J Jozwiak reports personal fees from Teva, Amgen, Synexus, Boehringer Ingelheim, Zentiva, and Sanofi. K Krishan reports non-financial support from the UGC Centre of Advanced Study, CAS II, Department of Anthropology, Panjab University, Chandigarh, India. A-F Mentis reports funding from MilkSafe: A novel pipeline to enrich formula milk using omics technologies', a research co-financed by the European Regional Development Fund of the European Union and Greek national funds through the Operational Program Competitiveness, Entrepreneurship and Innovation, under the call RESEARCH - CREATE - INNOVATE (project code: T2EDK-02 222), as well as from ELIDEK (Hellenic Foundation for Research and Innovation, MIMS-860), stocks from "Vinellas. GR", and a paid position as a scientific officer for the BGI group. S R Pandi-Perumal receives royalties from Springer for academic books edited. A Radfar reports other support from Avicenna Medical and Clinical Research Institute. S Shrestha reports a research merit scholarship from the School of Pharmacy, Monash University, Malaysia. M Solmi reports honoraria and consulting fees from Angelini, Lundbeck, and Otsuka. E Upadhyay reports published patents "A system and method of reusable filters for anti-pollution mask" and "A system and method for electricity generation through crop stubble by using microbial fuel cells" and filed patents "A system for disposed personal protection equipment (PPE) into biofuel through pyrolysis and method" and "A novel herbal pharmaceutical aid for formulation of gel and method thereof". All outside the submitted work.
